# Huisgen Cycloaddition of Azidoazulenes: Synthesis, Structural and Optical Properties of 2- and 6-(1,2,3-Triazol-1-yl)azulenes

**DOI:** 10.3390/molecules31020221

**Published:** 2026-01-08

**Authors:** Taku Shoji, Miku Yoshida, Masayuki Iwabuchi, Mitsuki Furuhata, Shigeki Mori, Tetsuo Okujima, Ikumi Uchiyama, Ryuta Sekiguchi, Shunji Ito

**Affiliations:** 1 Department of Chemical Biology and Applied Chemistry, College of Engineering, Nihon University, Koriyama 963-8642, Fukushima, Japan; 2 Advanced Research Support Center, Ehime University, Matsuyama 790-8577, Ehime, Japan; 3 Graduate School of Science and Engineering, Ehime University, Matsuyama 790-8577, Ehime, Japan; 4 Graduate School of Science and Technology, Hirosaki University, Hirosaki 036-8561, Aomori, Japan

**Keywords:** azulene, azido, triazole, Huisgen [3 + 2] cycloaddition, single crystal X-ray analysis

## Abstract

We developed an efficient and modular route to 2- and 6-(1,2,3-triazol-1-yl)azulenes to expand the synthetic accessibility and functional scope of azulene-based π-systems with stimulus-responsive photophysics. Readily accessible 2- and 6-azidoazulenes, prepared in excellent yields via S_N_Ar reactions of haloazulenes, were subjected to Cu(I)-catalyzed Huisgen [3 + 2] cycloaddition with a broad range of terminal alkynes to afford the corresponding triazolylazulenes in good to high yields, followed by acid-mediated decarboxylation and Staudinger reduction to enable further diversification to 2-azulenyltriazoles and a 6-aminoazulene derivative. Single-crystal X-ray diffraction analysis revealed substitution-position-dependent torsional arrangements and variations in π-conjugation between the azulene and triazole units. Photophysical characterization by UV/Vis absorption and fluorescence spectroscopy showed pronounced halochromism under acidic conditions, and selected derivatives displayed substantially enhanced fluorescence quantum yields. Overall, these results establish the azulene–1,2,3-triazole motif as a versatile building block for designing optoelectronic π-systems with acid-responsive emission properties.

## 1. Introduction

**Figure 1:**
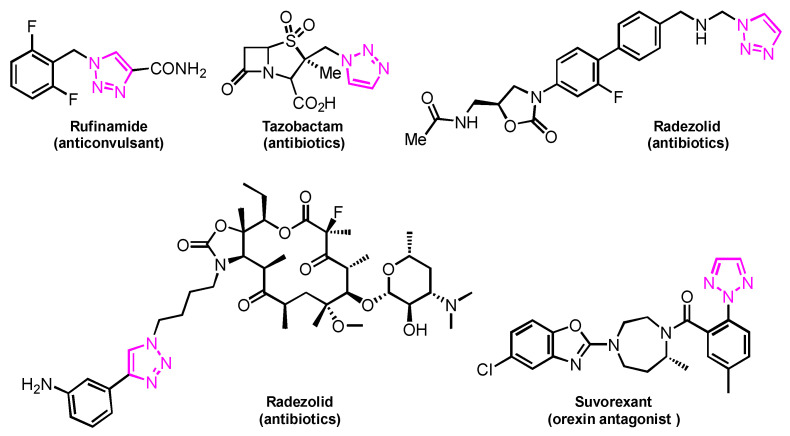
Pharmaceuticals containing a 1,2,3-triazole substructure.

1,2,3-Triazoles are frequently encountered as structural motifs in pharmaceuticals, such as Rufinamide [1], Tazobactam [2], Solithromycin [3], and others [4,5], owing to their ability to function as bioisosteres of imidazoles and various carboxylic acid derivatives (Figure 1). In addition, 1,2,3-triazoles have attracted considerable attention as ligands for metal coordination [6,7,8] and as functional components for electrolyte applications such as ionic liquids [9,10]. Among the established synthetic methods, the most widely employed approach to the 1,2,3-triazoles is the Huisgen [3 + 2] dipolar cycloaddition between azides and alkynes. In the early development of this reaction, azides and alkynes were thermally activated to promote the cycloaddition reaction [11,12]. Subsequently, Sharpless introduced the concept of “click chemistry” for the Huisgen cycloaddition, now extensively utilized in the construction of pharmaceuticals, biological probes, and functional organic materials because of its high efficiency, broad functional-group tolerance, and operational simplicity [13]. The Huisgen reaction—classified as a click transformation—enables the rapid formation of the 1,2,3-triazole derivatives via the Cu(I)-catalyzed [3 + 2] cycloaddition of azides with terminal alkynes [14,15,16].

Previously, Nozoe, Horino and Toda reported that, in the course of investigating the reactivity of 5-*N*-(1,2,3-triazol-1-yl)tropolone, the reaction of this ether derivative with malononitrile or ethyl cyanoacetate afforded the corresponding 6-(1,2,3-triazol-1-yl)azulenes [17,18]. In 2014, Emrick and co-workers also reported the synthesis of a 2-(1,2,3-triazol-1-yl)azulene derivative as a monomer precursor for functional polymer development [19]. This compound was obtained via a copper(I) iodide (CuI)-catalyzed Huisgen cycloaddition between 2-trimethylsilylethynylazulene and the corresponding azide. However, despite the fact that azidoazulenes themselves are relatively accessible, their reactivity in the Huisgen cycloadditions with alkynes has not been systematically explored, and no examples of such transformations have been disclosed to date.

In the present study, as part of our ongoing investigation into the reactivity of diethyl 2- and 6-azidoazulene-1,3-dicarboxyltes, we explored the CuI-catalyzed Huisgen cycloaddition with a range of alkynes, establishing an efficient synthetic route to 2- and 6-(1,2,3-triazol-1-yl)azulene drerivatives. In addition, we developed a convenient protocol for the preparation of 6-aminoazulene derivative via the Staudinger reaction of the corresponding 6-azidoazulene derivative. The synthesis of 2-(1,2,3-triazol-1-yl)azulenes without the ester substituents was also investigated, and optical measurements revealed that several of these derivatives exhibit relatively strong fluorescence with relatively high quantum yields. Furthermore, the detailed molecular structures of the 2- and 6-(1,2,3-triazol-1-yl)azulenes were established by single-crystal X-ray diffraction analysis.

## 2. Results and Discussion

**Scheme 1:**
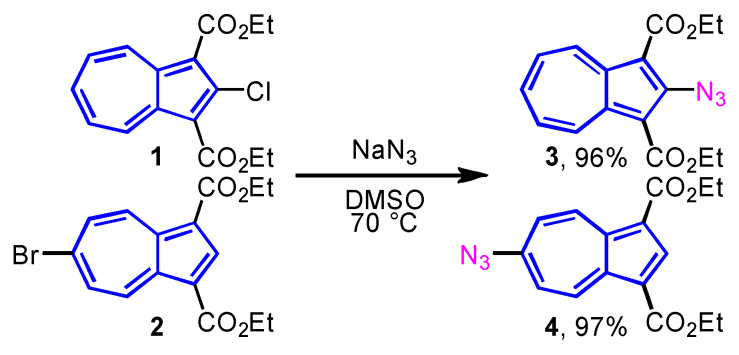
Preparation of 2- and 6-azidoazulenes **3** and **4**.

**Synthesis**: Prior to the synthesis of azulene-substituted triazoles, azidoazulenes were prepared from 2-chloro and 6-bromoazulene derivatives **1** and **2** bearing two-ethoxycarbonyl functions at the 1,3-positions of the azulene ring. Since the synthesis of 6-azidoazulene derivative **4** was previously reported by us, a similar approach was adopted for the preparation of 2-azidoazulene derivative **3** [20]. Thus, the preparation of 2- and 6-azidoazulene derivatives **3** and **4 [21]** were accomplished in 96% and 97% yields, respectively, by the aromatic nucleophilic substitution (S_N_Ar) reaction of **1** and **2** with sodium azide (NaN_3_) in dimethyl sulfoxide (DMSO) at 70 °C (Scheme 1).

The Huisgen cycloaddition of **3** with various alkynes in the presence of a CuI catalyst furnished 2-(1,2,3-triazol-1-yl)azulenes **5a**–**5g**, bearing an aromatic or an alkyl substituent at the 4-position, in good to excellent yields (Table 1). The electronic nature of the substituents at the *para*-position of the arylalkynes exerted little influence on the reaction outcome, and **5a**–**5c** were obtained in almost comparable yields (entries 1–3). The reaction of 1-ethynylnaphthalene afforded **5d** in 74% yield, similar to those of phenylacetylenes (entry 4). In contrast, electron-rich alkynes such as 1-ethynylazulene and ethynylferrocene [22] gave the corresponding cycloadducts **5e** and **5f** in 88% and 85% yields, respectively, indicating enhanced reactivity under these conditions (entries 5 and 6). Furthermore, the cycloaddition of **3** with an alkyl-substituted alkyne, 1-hexyne, also proceeded smoothly to afford **5g** in 72% yield (entry 7).

Similarly, 6-azidoazulene derivative **4** also underwent smooth Huisgen cycloaddition with a range of alkynes to afford the corresponding 6-azulenyltriazoles **6a**–**6g** in good yields (Table 2). Reactions of **4** with benzene-substituted alkynes in the presence of catalytic amount of CuI provided **6a** (84%), **6b** (86%), and **6c** (69%) (entries 1–3). The reaction of **4** with 1-ethynylnaphthalene, followed by chromatographic purification on silica gel, furnished **6d** in 82% yield (entry 4). Furthermore, the reactions of **4** with 1-ethynylazulene and ethynylferrocene afforded di(azulenyl)triazole **6e** (76%) and ferrocenyl derivative **6f** (74%), respectively (entries 5 and 6). The cycloaddition of **4** with 1-hexyne also proceeded efficiently to give *n*-butyl-substituted triazole **6g** in 65% yield (entry 7).

As summarized in Table 1 and Table 2, the Huisgen cycloaddition of both azides **3** and **4** with diverse alkynes proceeded efficiently to furnish the corresponding 1,2,3-triazoles in consistently high yields, demonstrating that **3** and **4** possess high intrinsic reactivity toward the [3 + 2] dipolar cycloaddition.

The decarboxylation reactions of **5a**–**5g** were investigated under acidic conditions. In general, 100% phosphoric acid (H_3_PO_4_) is widely employed for the decarboxylation of ester groups in azulene derivatives. However, owing to the limited solubility of **5a**–**5g** in 100% H_3_PO_4_, methanesulfonic acid (MeSO_3_H) was selected as an alternative reaction medium [23]. This modification was expected to improve the solubility of the substrates and thereby facilitate a more efficient decarboxylation process. In practice, the decarboxylation reaction of **5a**–**5g** proceeded smoothly to afford the corresponding products **7a**–**7g** in excellent yields (88–95%) in most cases (Table 3). Notably, in the case of **5e**, decarboxylation also occurred at the ester group on the substituted 1-azulenyl moiety, affording the fully decarboxylated product **7e’** in 89% yield (entry 5). In contrast, the ferrocene-substituted derivative **5f** underwent significant decomposition under the reaction conditions, and no isolable product could be obtained (entry 6).

**Scheme 2:**
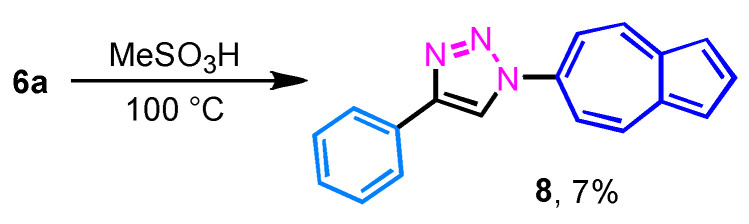
Decarboxylation of **6a** to 6-(4-phenyl-1,2,3-triazol-1-yl)azulene **8**.

Similarly, the decarboxylation of **6a** was also examined under the conditions comparable to those used for the decarboxylation of **5a**–**5g**. However, unlike **5a**–**5g**, the reaction of **6a** was accompanied by extensive decomposition, and the desired product **8** was isolated in only 7% yield (Scheme 2). This result indicates that the 6-substituted derivative is less stable than the corresponding 2-substituted analogues under the decarboxylation conditions.

The Staudinger reaction is a well-established method for the conversion of azides into primary amines under relatively mild conditions [24,25,26,27]. In this transformation, azides react with phosphines to afford iminophosphorane intermediates, which can subsequently be hydrolyzed to yield the corresponding primary amines. Although the synthesis of 6-aminoazulene derivative **10** via the reduction of **4** was previously reported by us [17], that method required the use of Raney-Ni—a highly reactive reducing agent. To develop a more convenient and safer alternative, we explored the Staudinger reaction as a method for converting **4** into **10**.

**Scheme 3:**
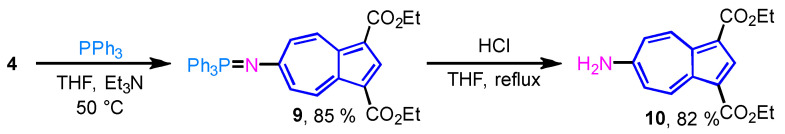
Transformation to 6-aminoazulene derivative **10** via Staudinger reaction.

The reaction of **4** with triphenylphosphine (PPh_3_) proceeded smoothly to afford the corresponding iminophosphorane **9** in 85% yield. The detailed structure of **9** was confirmed by single-crystal X-ray diffraction analysis, as described later. Subsequent acidic hydrolysis of **9** with aqueous HCl in THF furnished the desired 6-aminoazulene derivative **10** in 82% yield (Scheme 3). This two-step sequence provides a safer and more convenient alternative for the synthesis of the 6-aminoazulene derivative compared with the previously reported Raney-Ni reduction protocol.

**Spectroscopic properties:** The 2- and 6-(1,2,3-triazol-1-yl)azulenes obtained in this study were fully characterized based on their spectroscopic data, as summarized in Section 3. The ^1^H NMR spectral assignments of the reported compounds were confirmed by COSY experiments (see Supplementary Materials). The high-resolution mass spectra (HRMS) of the newly prepared compounds, recorded using ESI-TOF or MALDI-TOF ionization, displayed the expected molecular ion peaks, consistent with the proposed molecular formulas. These spectroscopic results collectively support the assigned structures of the synthesized products.

**Figure 2:**
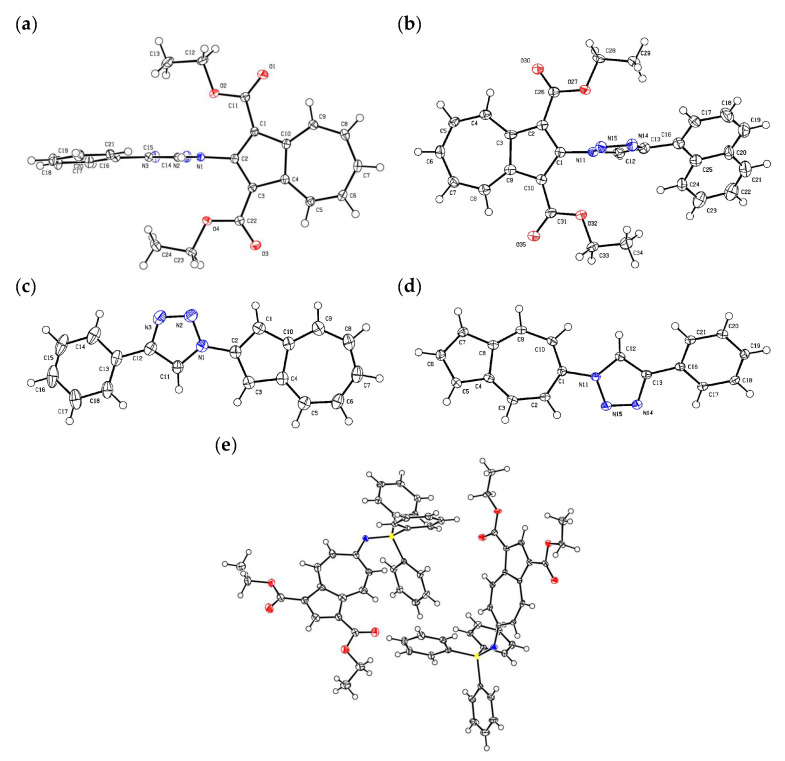
ORTEP drawing of (**a**) **5a** (CCDC 2087438), (**b**) **5d** (CCDC 2447293), (**c**) **7a** (CCDC 2502616), (**d**) **8** (CCDC 2447292), and (**e**) **9** (CCDC 2088856); ellipsoids are drawn at the 50% probability level.

Since suitable single crystals for X-ray crystallographic analysis were obtained for **5a**, **5d**, **8**, and **9**, their molecular structures were elucidated as summarized in Figure 2. In compounds **5a** and **5d**, the dihedral angle between the azulene and triazole rings was determined to be 85.69° (**5a**) and 85.98° (**5d**), indicating that the two rings are nearly orthogonal. These large torsions are attributed to steric repulsion between the triazole moiety and the two ester substituents substituted at the 1,3-positions of the azulene ring, which reduces the overall planarity of the π-system. In contrast, the dihedral angles between the triazole and the pendant phenyl and 1-naphtyl groups were found to be 8.02° (**5a**) and 36.55° (**5d**), signifying a coplanarity as a result of reduced steric hindrance.

Upon decarboxylation, the steric environment changed markedly. In the decarboxylated derivative **7a**, the dihedral angle between the azulene and triazole rings was significantly reduced to 8.13°. In contrast, **8** exhibited larger dihedral angles of 33.23° (azulene–triazole) and 18.87° (triazole–phenyl). The increased torsion between the azulene and triazole rings in **8** is plausibly attributed to steric repulsion between the hydrogen atoms on the seven-membered ring of the azulene moiety and the adjacent triazole ring.

A partially planar conformation was also observed in the crystal structure of **9**, in which the dihedral angle between the azulene framework and the iminophosphorane moiety was only 0.41°, suggesting efficient conjugation between these two π-systems.

To investigate the effects of the substitution position on the triazole ring and the substituents on the introduced aryl groups on the optical properties, UV/Vis absorption spectra of compounds **5**–**8** were measured. The maximum absorption wavelengths (λ_max_) and the molar absorption coefficients (log ε) for compounds **5** and **6** in dichloromethane (CH_2_Cl_2_) as well as **7** in 10% CF_3_CO_2_H (TFA)/CH_2_Cl_2_ are summarized in Table 4 and Table 5, respectively.

Overall, the absorption maxima of **6a**–**6g** were red-shifted in the visible region compared with those of **5a**–**5g** (Table 4). Compound **5b** (λ_max_ = 524 nm), bearing an electron-donating *p*-anilino group, exhibited a slightly red-shifted absorption maximum relative to **5a** (λ_max_ = 513 nm). In contrast, **5c** (λ_max_ = 512 nm), containing an electron-withdrawing *p*-nitrophenyl group, displayed an absorption maximum nearly identical to that of **5a**. For 1-naphthyl derivative **5d** (λ_max_ = 508 nm), which possesses a π-extended aromatic ring, the absorption maximum also showed little deviation from that of phenyl analogue **5a**. This behavior is likely attributable to the near-orthogonal orientation of the azulene and triazole rings, as revealed by single-crystal X-ray structural analysis, where steric hindrance from the two ester substituents minimizes π-conjugation overlap between the two systems. Compound **5e** (λ_max_ = 522 nm) exhibited a prominent absorption band in the visible region with approximately twice the molar absorption coefficient of **5a**, a feature reasonably ascribed to the overlap of π–π* transitions of the two azulene units. Meanwhile, the absorption maxima of **5f** (λ_max_ = 517 nm) and **5g** (λ_max_ = 514 nm) were similar to that of **5a**, indicating that the absorption characteristics are governed predominantly by the 1,3-diethoxycarbonylazulene core, with only minor electronic contributions from the substituents.

Conversely, despite the relatively high planarity observed in the X-ray crystallographic structures of **6a**–**6g**, neither the electronic effects nor the conjugation extension of the aryl substituents on the triazole ring were reflected in their absorption maxima. This behavior may be attributed to the weak electronic influence of substituents at the 6-position of the azulene ring, where electronic communication with the π-system is comparatively limited.

The UV/Vis spectra of **7a**–**7g** in CH_2_Cl_2_ are also measured (see Section 3). All compounds exhibited two distinct absorption bands in the range of λ_max_ = 540–585 nm, accompanied by a weak shoulder around 630–655 nm. These absorptions can be assigned to the π–π* transitions derived from the azulene ring. Notably, even for **7b** (λ_max_ = 593 nm) and **7c** (λ_max_ = 598 nm), bearing electron-donating and electron-withdrawing substituents, respectively, the absorption maxima appeared at nearly identical wavelengths. These observations suggest that both conjugation and substituent effects are electronically decoupled by the triazole spacer, preventing efficient transmission of the substituent influence to the azulene core. Since compound **8** (λ_max_ = 593 nm) exhibited an absorption maximum similar to those of **7a**–**7g**, the substitution position of the triazole ring on the azulene framework may not significantly influence its electronic properties.

Azulene derivatives are well known for exhibiting a color change dependent on pH, so called halochromism, under acidic conditions [27,28,29]. We have also found that several azulene derivatives not only induce halochromism but also display remarkable fluorescence under acidic conditions [30,31,32]. Since a series of **7** might also exhibit halochromism, UV/Vis absorption and fluorescence spectra were also measured under acidic conditions. The maximum absorption (λ_max_) and fluorescence (λ_Flu_) wavelengths, and fluorescence quantum yields (Φ_Flu_) in 10% TFA/CH_2_Cl_2_ are presented in Table 5.

All compounds exhibited distinct spectral changes in both band shape and absorption intensity under the acidic conditions. In CH_2_Cl_2_, **7a**–**7g** showed absorption bands at around 540–585 nm, accompanied by weak shoulders at near 630–655 nm. In contrast, when measured in 10% TFA/CH_2_Cl_2_, a new strong absorption band emerged at approximately 410–420 nm, while the absorption intensity in the longer-wavelength visible region (550–600 nm) increased slightly. These pronounced spectral changes could be attributed to the protonation of the azulene core or triazole ring by TFA, which perturbs the π-electronic structure and changes electronic character. Regarding the substituent effects, both **7a** and **7b** exhibited the emergence of a pronounced new absorption band at approximately 410–421 nm, whereas the position of the long-wavelength visible band remained nearly identical to that observed under neutral conditions. In sharp contrast, **7c** displayed distinct new absorption bands at λ_max_ = 412 nm and 472 nm, together with a pronounced enhancement of the absorption intensity in the visible region. In 10% TFA/CH_2_Cl_2_, the absorption maximum of **8** (λ_max_ = 619 nm) showed a slight red shift compared with those of **7a**–**7g**.

**Figure 3:**
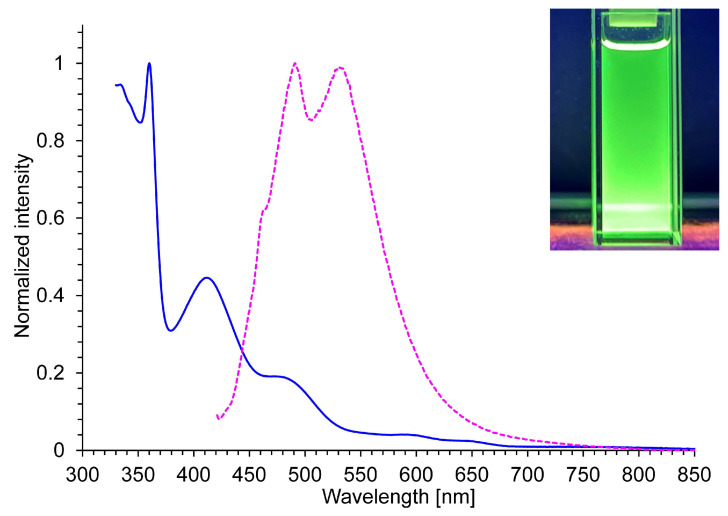
UV/Vis (blue line) and fluorescent spectra (λ_ex_ = 405 nm, pink dotted-line) of **7c** in 10% TFA/CH_2_Cl_2_; Photograph: **7c** in 10% TFA/CH_2_Cl_2_ under the UV-light irradiation at λ_ex_ = 365 nm.

As is typical for azulene derivatives, **7a**–**7g** and **8** exhibited exceedingly weak fluorescence in CH_2_Cl_2_, presumably arising from the S_2_→S_0_ transition. However, the fluorescence spectra displayed only faint, poorly resolved emission features, and the corresponding fluorescence quantum yields were below the instrumental detection limit. By contrast, in 10% TFA/CH_2_Cl_2_, **7a**–**7g** and **8** showed visually discernible fluorescence. The maximum fluorescence wavelength (λ_Flu_) and fluorescence quantum yields (Φ_Flu_) of **7** in 10% TFA/CH_2_Cl_2_ are also summarized in Table 5. All compounds exhibited multiple emission peaks within the range of λ_Flu_ = 470–540 nm. However, the Φ_Flu_ values varied substantially depending on the aryl substituents. Compounds **7a** (λ_Flu_ = 475, 489, and 540 nm), **7b** (λ_Flu_ = 491 and 534 nm), and **7d** (λ_Flu_ = 463, 489, and 529 nm) showed extremely weak fluorescence (Φ_Flu_ < 1%), whereas **7e**, despite its low overall efficiency (Φ_Flu_ = 2.3%), displayed clear fluorescence at λ_Flu_ = 472 and 523 nm. In contrast, **7c** (λ_Flu_ = 492 and 533 nm) and **7g** (λ_Flu_ = 472 and 530 nm) exhibited markedly enhanced Φ_Flu_ values of 27.0% and 10.8%, respectively (Figure 3).

**Figure 4:**
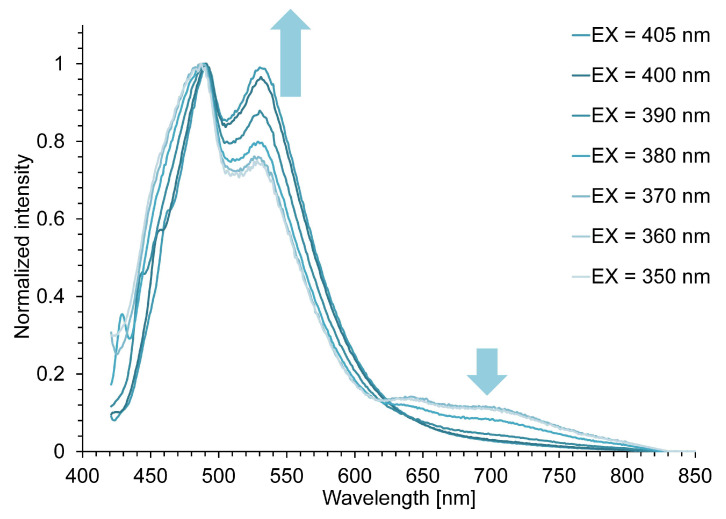
Fluorescence spectra of **7c** in 10% TFA/CH_2_Cl_2_ under different excitation wavelengths.

To investigate these emission behaviors, the fluorescence spectra of **7c** in 10% TFA/CH_2_Cl_2_ were measured while varying the excitation wavelength from 350 nm to 405 nm (Figure 4). If the intensity ratio between the shorter and longer-wavelength emissions changes upon varying the excitation wavelength, it can be considered that the fluorescence originates from multiple emissive species. In practice, the fluorescence spectra of **7c** showed a decrease in the near-infrared emission band and a simultaneous increase in the fluorescence intensity at λ_Flu_ = 533 nm as the excitation wavelength was shifted to longer wavelengths. This excitation-wavelength dependence of the fluorescence spectra indicates that the fluorescence of **7c** arises from multiple emissive species.

**Figure 5:**
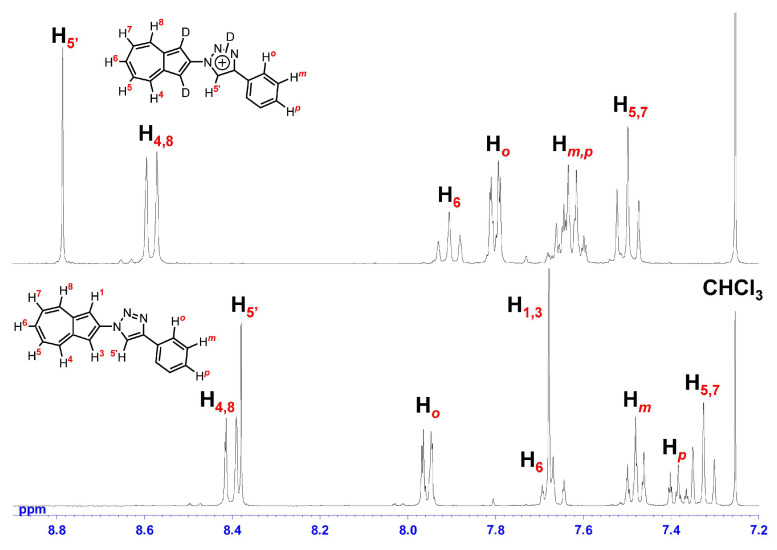
^1^H NMR spectra (400 MHz) of **7a** in CDCl_3_ (bottom) and 10% CF_3_CO_2_D/CDCl_3_ (top).

**Figure 6:**
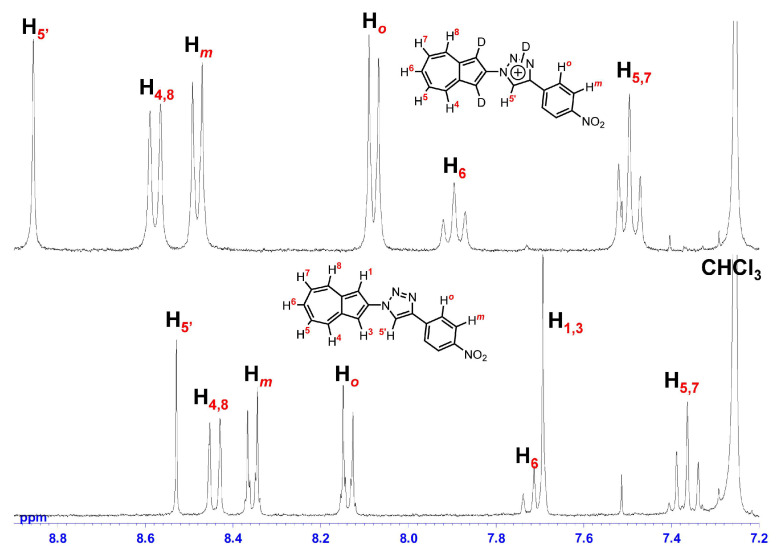
^1^H NMR spectra (400 MHz) of **7c** in CDCl_3_ (bottom) and 10% CF_3_CO_2_D/CDCl_3_ (top).

To elucidate the structure of the species formed in acidic medium, the ^1^H NMR spectra of **7a** and **7c** were recorded in 10% CF_3_CO_2_D/CDCl_3_ (Figure 5 and Figure 6). Formation of azulenium ion in acidic media induces a pronounced downfield shift of proton signals on the seven-membered ring [33,34,35], and the protonation at the five-membered ring disrupts the inherent symmetry of the azulene skeleton. In the present case, the azulene proton signals for **7a** and **7c** were observed while retaining symmetry even in 10% CF_3_CO_2_D/CDCl_3_. This indicates that protonation preferentially occurs at the triazole nitrogen rather than at the azulene ring. The proton signals of **7a** and **7c** in 10% CF_3_CO_2_D/CDCl_3_ were shifted downfield overall relative to those in CDCl_3_, which can be attributed to the increased electron-withdrawing nature by the protonation of the triazole ring such as the structure in Figure 5 and Figure 6. Notably, the H_1_ and H_3_ protons of both **7a** and **7c** disappeared due to proton-deuterium exchange, suggesting transient formation of azulenium ion intermediates whose contribution is not dominant on the ^1^H NMR timescale. Accordingly, the differences in Φ_Flu_ observed across the series are plausibly governed by the electronic states of the protonated species and/or the generation of the transient azulenium ion intermediates. Accordingly, emission maxima observed in the fluorescence spectra may originate from the coexistence of two chemically distinct emissive species, namely, triazolium and azulenium ions in equilibrium, or from an excited-state intramolecular proton transfer (ESIPT) process from the triazole moiety to the azulene unit [36,37,38]. Moreover, the higher Φ_Flu_ of **7c** may be rationalized by a greater contribution of the azulenium ion, stemming from either an equilibrium shift or an ESIPT, facilitated by destabilization of the triazolium cation induced by the strongly electron-withdrawing p-nitrophenyl substituent.

## 3. Materials and Methods

**General:** Melting points were determined with a Yanagimoto MPS3 micromelting apparatus. UV/Vis spectra were measured with JASCO V-670 spectrophotometer. The fluorescence spectra and fluorescence quantum yield were measured with JASCO FP-8350 and Hamamatsu C11347 absolute photoluminescence quantum yield spectrometer, respectively. The UV/Vis spectra, fluorescence spectra, and fluorescence quantum yields were measured at room temperature. ^1^H and ^13^C NMR spectra were recorded with a JEOL ECA500 at 500 MHz (^1^H NMR) and 125 MHz (^13^C NMR) or a JEOL ECZ400 at 400 MHz (^1^H NMR) and 100 MHz (^13^C NMR), respectively. The HRMS data were obtained with a Waters Xevo G2-XS QTof (ESI-TOF) or a Bruker Daltonics autoflex maX (MALDI-TOF).

**Diethyl 2-azidoazulene-1,3-dicarboxylate (3):** A mixture of 3-chloro-1,3-diethoxycarbonylazulene (**1**) (1.54 g, 5.02 mmol) and sodium azide (503 mg, 7.72 mmol) in DMSO (30 mL) was heated at 70 °C in an oil bath for 4 h under an Ar atmosphere. The reaction mixture was poured into water and extracted with AcOEt. The organic layer was washed with water and brine, dried over Na_2_SO_4_, and concentrated under reduced pressure to afford the product **3** (1.53 g, 97%) as a red oil. IR (ATR): ν_max_ = 2998 (w), 2979 (w), 2110 (m), 1694 (s), 1676 (m), 1592 (w), 1533 (w), 1498 (w), 1434 (s), 1411 (m), 1382 (w), 1296 (m), 1211 (s), 1180 (s), 1113 (w), 1093 (w), 1069 (m), 1056 (m), 1031 (m), 966 (w), 883 (w), 848 (w), 824 (w), 789 (m), 768 (w), 737 (w), 674 (w) cm^−1^; ^1^H NMR (500 MHz, CDCl_3_): δ_H_ = 9.45 (dd, *J* = 9.7, 1.1 Hz, 2H), 7.84–7.80 (m, 1H), 7.68 (t, *J* = 9.9 Hz, 2H), 4.49 (q, *J* = 7.1 Hz, 4H), 1.47 (t, *J* = 7.2 Hz, 6H) ppm; ^13^C NMR (125 MHz, CDCl_3_): δ_C_ = 164.32, 149.20, 142.52, 138.97, 137.12, 131.41, 109.42, 60.70, 14.57 ppm; HRMS (ESI-TOF, Positive): calcd for [C_16_H_15_N_3_O_4_ + H]^+^ 314.1135, found: 314.1147.

**Diethyl 6-azidoazulene-1,3-dicarboxylate (4):** A solution of **2** (358 mg, 1.02 mmol) and sodium azide (170 mg, 2.61 mmol) in DMSO (30 mL) was heated at 70 °C in an oil bath for 2 h under an Ar atmosphere. The reaction mixture was poured into water and extracted with ethyl acetate. The combined organic layers were washed with water, dried over Na_2_SO_4_, and concentrated under reduced pressure. The residue was purified by column chromatography on silica gel using ethyl acetate as eluent to afford **4** (304 mg, 95%) as a yellow solid. M.p. 150–152 °C (lit. 150.5–152 °C); ^1^H NMR (500 MHz, CDCl_3_): δ_H_ = 9.65 (dd, *J* = 9.9, 1.3 Hz, 2H), 8.68 (s, 1H), 7.32 (dt, *J* = 11.1, 1.1 Hz, 2H), 4.42 (q, *J* = 7.1 Hz, 4H), 1.44 (t, *J* = 7.2 Hz, 6H) ppm; ^13^C NMR (125 MHz, CDCl_3_): δ_C_ = 165.02, 152.43, 141.75, 140.99, 138.41, 120.99, 117.69, 60.25, 14.63 ppm; HRMS (ESI-TOF, Positive): calcd for [C_16_H_15_N_3_O_4_ + H]^+^ 314.1135; found: 314.1147.

**Diethyl 2-(4-phenyl-1*H*-1,2,3-triazol-1-yl)azulene-1,3-dicarboxylate (5a):** To a degassed solution of **3** (158 mg, 0.50 mmol) and ethynylbenzene (115 mg, 1.13 mmol) in THF (2.5 mL) and triethylamine (2.5 mL) was added CuI (11 mg, 0.06 mmol). The mixture was heated at 50 °C in an oil bath for 17 h under an Ar atmosphere. The reaction mixture was poured into a 10% NH_4_Cl solution and extracted with toluene. The combined organic layers were washed with brine, dried over Na_2_SO_4_, and concentrated under reduced pressure. The residue was purified by column chromatography on silica gel using toluene as eluent to afford **5a** (160 mg, 77%) as a purple solid. M.p. 140–143 °C; IR (ATR): ν_max_ = 3143 (w), 2978 (w), 1678 (s), 1510 (w), 1479 (w), 1459 (w), 1431 (s), 1414 (m), 1385 (m), 1354 (w), 1322 (w), 1299 (w), 1256 (m), 1214 (m), 1180 (s), 1154 (m), 1112 (w), 1084 (w), 1060 (m), 1042 (m), 1025 (m), 881 (w), 826 (w), 792 (m), 761 (m), 741 (w), 718 (w), 690 (m), 656 (w) cm^−1^; UV/Vis (CH_2_Cl_2_): λ_max_ (log ε) = 236 (4.62), 272 (4.47), 291 sh (4.61), 302 (4.73), 361 (4.01), 495 sh (2.99), 513 (3.00), 570 sh (2.60) nm; ^1^H NMR (500 MHz, CDCl_3_): δ_H_ = 9.96–9.94 (m, 2H), 8.17–8.12 (m, 2H), 7.95–7.89 (m, 4H), 7.49–7.46 (m, 2H), 7.39–7.36 (m, 1H), 4.18–4.14 (m, 4H), 1.01 (t, *J* = 7.2 Hz, 6H) ppm; ^13^C NMR (125 MHz, CDCl_3_): δ_C_ = 163.63, 146.61, 143.65, 142.87, 141.84, 141.65, 131.94, 130.82, 129.04, 128.16, 125.88, 123.38, 113.15, 60.67, 13.93 ppm; HRMS (ESI-TOF, Positive): calcd for [C_24_H_21_N_3_O_4_ + H]^+^ 416.1605; found: 416.1608.

**Diethyl 2-[4-(4-aminophenyl)-1*H*-1,2,3-triazol-1-yl]azulene-1,3-dicarboxylate (5b):** To a degassed solution of **3** (158 mg, 0.50 mmol) and 4-ethynylaniline (103 mg, 0.88 mmol) in THF (2.5 mL) and triethylamine (2.5 mL) was added CuI (12 mg, 0.06 mmol). The mixture was heated at 50 °C in an oil bath for 17 h under an Ar atmosphere. The reaction mixture was poured into a 10% NH_4_Cl solution and extracted with toluene. The combined organic layers were washed with brine, dried over Na_2_SO_4_, and concentrated under reduced pressure. The residue was purified by column chromatography on silica gel using toluene/AcOEt as eluent to afford **5b** (162 mg, 75%) as a red solid. M.p. 199–202 °C; IR (ATR): ν_max_ = 3415 (w), 3347 (w), 2977 (w), 1692 (m), 1674 (m), 1643 (w), 1613 (m), 1563 (w), 1513 (m), 1489 (m), 1474 (m), 1460 (m), 1432 (s), 1386 (m), 1352 (w), 1332 (w), 1293 (w), 1252 (s), 1223 (m), 1212 (m), 1189 (s), 1141 (m), 1117 (w), 1080 (m), 1057 (m), 1038 (s), 888 (w), 865 (w), 847 (w), 829 (m), 816 (m), 792 (m), 764 (w), 730 (m), 712 (w), 700 (w), 683 (m), 655 (w), cm^−1^; UV/Vis (CH_2_Cl_2_): λ_max_ (log ε) = 236 (4.48), 272 (4.57), 294 (4.69), 333 sh (4.00), 362 (3.97), 499 (3.00), 524 sh (2.98), 574 (2.57) nm; ^1^H NMR (500 MHz, CDCl_3_): δ_H_ = 9.92 (d, *J* = 9.7 Hz, 2H), 8.13 (t, *J* = 9.9 Hz, 1H), 7.97 (s, 1H), 7.90 (t, *J* = 10.2 Hz, 2H), 7.73 (d, *J* = 8.6 Hz, 2H), 6.78 (d, *J* = 8.6 Hz, 2H), 4.15 (q, *J* = 7.1 Hz, 4H), 3.79 (s, 2H), 1.02 (t, *J* = 7.2 Hz, 6H) ppm; ^13^C NMR (125 MHz, CDCl_3_): δ_C_ = 163.72, 146.94, 146.52, 143.90, 142.72, 141.70, 141.66, 131.86, 127.12, 122.16, 121.32, 115.45, 113.17, 60.67, 13.94 ppm; HRMS (ESI-TOF, Positive): calcd for [C_24_H_22_N_4_O_4_ + H]^+^ 431.1714; found: 431.1733.

**Diethyl 2-[4-(4-nitrophenyl)-1*H*-1,2,3-triazol-1-yl]azulene-1,3-dicarboxylate (5c):** To a degassed solution of **3** (158 mg, 0.50 mmol) and 4-ethynylnitrobenzene (147 mg, 1.00 mmol) in THF (2.5 mL) and triethylamine (2.5 mL) was added CuI (11 mg, 0.06 mmol). The mixture was heated at 50 °C in an oil bath for 17 h under an Ar atmosphere. The reaction mixture was poured into a 10% NH_4_Cl solution and extracted with toluene. The combined organic layers were washed with brine, dried over Na_2_SO_4_, and concentrated under reduced pressure. The residue was purified by column chromatography on silica gel using toluene/AcOEt as eluent to afford **5c** (178 mg, 77%) as a red solid. M.p. 222–225 °C; IR (ATR): ν_max_ = 3139 (w), 3111 (w), 2988 (w), 1703 (m), 1684 (m), 1604 (w), 1591 (w), 1512 (s), 1473 (w), 1436 (s), 1385 (w), 1341 (s), 1313 (w), 1286 (w), 1254 (m), 1215 (m), 1183 (m), 1144 (w), 1110 (w), 1077 (w), 1054 (w), 1040 (m), 967 (w), 887 (w), 853 (m), 795 (m), 768 (w), 757 (w), 745 (w), 728 (w), 711 (w), 688 (w), 680 (w), 661 (w) cm^−1^; UV/Vis (CH_2_Cl_2_): λ_max_ (log ε) = 232 (4.46), 273 (4.31), 303 (4.57), 335 sh (4.40), 365 sh (4.13), 493 sh (2.98), 512 (3.00) nm; ^1^H NMR (500 MHz, CDCl_3_): δ_H_ = 9.96 (d, *J* = 10.0 Hz, 2H), 8.35 (d, *J* = 8.6 Hz, 2H), 8.28 (s, 1H), 8.24–8.16 (m, 2H), 8.12 (d, *J* = 8.9 Hz, 2H), 7.95 (t, *J* = 10.0 Hz, 2H), 7.69 (d, *J* = 8.9 Hz, 1H), 4.17 (q, *J* = 7.1 Hz, 4H), 1.01 (t, *J* = 7.0 Hz, 6H) ppm; ^13^C NMR (125 MHz, CDCl_3_): δ_C_ = 163.42, 147.45, 144.44, 143.23, 142.16, 141.57, 133.50, 132.16, 126.18, 124.90, 124.61, 123.86, 113.06, 60.73, 13.96 ppm; HRMS (ESI-TOF, Positive): calcd for [C_24_H_20_N_4_O_6_ + H]^+^ 461.1456; found: 461.1480.

**Diethyl 2-[4-(naphthalen-1-yl)-1*H*-1,2,3-triazol-1-yl]azulene-1,3-dicarboxylate (5d):** To a degassed solution of **3** (158 mg, 0.50 mmol) and 1-ethynylnaphthalene (158 mg, 1.04 mmol) in THF (2.5 mL) and triethylamine (2.5 mL) was added CuI (11 mg, 0.06 mmol). The mixture was heated at 50 °C in an oil bath for 17 h under an Ar atmosphere. The reaction mixture was poured into a 10% NH_4_Cl solution and extracted with toluene. The combined organic layers were washed with brine, dried over Na_2_SO_4_, and concentrated under reduced pressure. The residue was purified by column chromatography on silica gel using toluene/AcOEt as eluent to afford **5d** (171 mg, 74%) as an orange solid. M.p. 174–176 °C; IR (ATR): ν_max_ = 3135 (w), 3063 (w), 2998 (w), 2978 (w), 1693 (m), 1679 (s), 1591 (w), 1506 (m), 1434 (s), 1414 (m), 1397 (m), 1384 (m), 1354 (w), 1317 (w), 1298 (w), 1265 (m), 1254 (m), 1215 (s), 1180 (s), 1152 (m), 1116 (w), 1062 (m), 1049 (m), 1033 (w), 998 (w), 943 (w), 885 (w), 839 (w), 800 (s), 793 (s), 776 (s), 744 (m), 715 (w), 687 (w), 669 (w), 660 (w) cm^−1^; UV/Vis (CH_2_Cl_2_): λ_max_ (log ε) = 273 (4.40), 294 sh (4.61), 304 (4.74), 362 (3.94), 493 sh (2.99), 508 (3.00) nm; ^1^H NMR (500 MHz, CDCl_3_): δ_H_ = 9.99 (d, *J* = 9.7 Hz, 2H), 8.59 (dd, *J* = 6.6, 2.9 Hz, 1H), 8.21 (s, 1H), 8.17 (t, *J* = 9.9 Hz, 1H), 7.96–7.90 (m, 5H), 7.61–7.53 (m, 3H), 4.21 (q, *J* = 7.2 Hz, 4H), 1.07 (t, *J* = 7.2 Hz, 6H) ppm; ^13^C NMR (125 MHz, CDCl_3_): δ_C_ = 163.71, 145.49, 142.93, 141.92, 141.76, 134.12, 132.01, 131.21, 128.94, 128.70, 128.15, 127.22, 126.71, 126.18, 126.12, 125.65, 125.42, 113.23, 60.75, 14.10 ppm; HRMS (ESI-TOF, Positive): calcd for [C_28_H_23_N_3_O_4_ + H]^+^ 466.1761; found: 466.1747.

**Diethyl 2-[4-(5-isopropyl-3-methoxycarbonylazulen-1-yl)-1*H*-1,2,3-triazol-1-yl]azulene-1,3-dicarboxylate (5e):** To a degassed solution of **3** (122 mg, 0.39 mmol) and 1-ethynyl-5-isopropyl-3-methoxycarbonylazulene (192 mg, 0.76 mmol) in THF (2 mL) and triethylamine (2 mL) was added CuI (11 mg, 0.06 mmol). The mixture was heated at 50 °C in an oil bath for 17 h under an Ar atmosphere. The reaction mixture was poured into a 10% NH_4_Cl solution and extracted with toluene. The combined organic layers were washed with brine, dried over Na_2_SO_4_, and concentrated under reduced pressure. The residue was purified by column chromatography on silica gel using toluene as eluent to afford **5e** (193 mg, 88%) as a brown solid. M.p. 208–211 °C; IR (ATR): ν_max_ = 2974 (w), 1685 (s), 1592 (w), 1518 (m), 1434 (s), 1386 (m), 1362 (w), 1321 (w), 1275 (w), 1191 (s), 1170 (m), 1136 (m), 1099 (w), 1053 (m), 1027 (m), 937 (w), 902 (m), 879 (w), 792 (m), 776 (m), 740 (w), 719 (w), 710 (w), 687 (w), 659 (w) cm^−1^; UV/Vis (CH_2_Cl_2_): λ_max_ (log ε) = 237 (4.71), 273 (4.65), 305 (4.97), 318 sh (4.71), 365 (4.20), 388 (4.10), 403 sh (4.03), 522 (3.18), 570 (3.03) nm; ^1^H NMR (500 MHz, CDCl_3_): δ_H_ = 9.98–9.95 (m, 2H), 9.82 (d, *J* = 2.0 Hz, 1H), 9.62 (d, *J* = 9.5 Hz, 1H), 8.63 (s, 1H), 8.20 (s, 1H), 8.15 (t, *J* = 9.7 Hz, 1H), 7.92 (t, *J* = 10.2 Hz, 2H), 7.83 (d, *J* = 10.6 Hz, 1H), 7.56 (t, *J* = 10.0 Hz, 1H), 4.17 (q, *J* = 7.2 Hz, 4H), 3.98 (s, 3H), 3.26 (t, *J* = 6.9 Hz, 1H), 1.45 (d, *J* = 6.9 Hz, 6H), 1.01–0.99 (m, 6H) ppm; ^13^C NMR (125 MHz, CDCl_3_): δ_C_ = 165.90, 163.70, 149.64, 143.86, 143.59, 142.83, 142.26, 141.81, 141.71, 139.95, 139.28, 138.79, 138.23, 137.32, 131.93, 127.85, 124.17, 117.09, 114.97, 113.23, 60.67, 51.20, 39.19, 24.74, 13.98 ppm; HRMS (ESI-TOF, Positive): calcd for [C_33_H_31_N_3_O_6_ + H]^+^ 566.2286, found: 566.2260.

**Diethyl 2-[4-ferrocenyl-1*H*-1,2,3-triazol-1-yl]azulene-1,3-dicarboxylate (5f):** To a degassed solution of **3** (161 mg, 0.51 mmol) and ethynylferrocene (221 mg, 1.05 mmol) in THF (2.5 mL) and triethylamine (2.5 mL) was added CuI (12 mg, 0.06 mmol). The mixture was heated at 50 °C in an oil bath for 17 h under an Ar atmosphere. The reaction mixture was poured into a 10% NH_4_Cl solution and extracted with toluene. The combined organic layers were washed with brine, dried over Na_2_SO_4_, and concentrated under reduced pressure. The residue was purified by column chromatography on silica gel using toluene/AcOEt as eluent to afford **5f** (226 mg, 85%) as a red solid. M.p. 191–194 °C; IR (ATR): ν_max_ = 2987 (w), 1686 (s), 1592 (w), 1525 (w), 1507 (w), 1457 (w), 1434 (s), 1385 (m), 1335 (w), 1319 (w), 1299 (w), 1252 (m), 1239 (w), 1214 (s), 1186 (s), 1154 (w), 1099 (m), 1061 (m), 1036 (s), 974 (w), 877 (m), 825 (m), 781 (w), 762 (w), 742 (w), 725 (w), 714 (w), 703 (w), 683 (w), 669 (w), 654 (w) cm^−1^; UV/Vis (CH_2_Cl_2_): λ_max_ (log ε) = 273 (4.47), 291 sh (4.59), 302 (4.71), 362 (4.00), 490 (3.30), 517 sh (3.26) nm; ^1^H NMR (500 MHz, CDCl_3_): δ_H_ = 9.89 (d, *J* = 9.7 Hz, 2H), 8.13 (t, *J* = 9.9 Hz, 1H), 7.89 (t, *J* = 10.2 Hz, 2H), 7.83 (s, 1H), 4.78 (t, *J* = 1.7 Hz, 2H), 4.34 (t, *J* = 1.9 Hz, 2H), 4.21 (q, *J* = 7.2 Hz, 4H), 4.17 (s, 5H), 1.14 (t, *J* = 7.2 Hz, 6H) ppm; ^13^C NMR (125 MHz, CDCl_3_): δ_C_ = 163.68, 145.31, 143.67, 142.66, 141.62, 141.53, 131.79, 123.01, 113.15, 75.86, 69.66, 68.71, 67.04, 60.69, 14.20 ppm; HRMS (ESI-TOF, Positive): calcd for [C_28_H_25_FeN_3_O_4_ + H]^+^ 524.1267, found: 524.1282; calcd: [C_28_H_25_FeN_3_O_4_]^+^ 523.1189, found: 523.1216.

**Diethyl 2-(4-butyl-1*H*-1,2,3-triazol-1-yl)azulene-1,3-dicarboxylate (5g):** To a degassed solution of **3** (157 mg, 0.50 mmol) and 1-hexyne (84 mg, 1.02 mmol) in THF (2.5 mL) and triethylamine (2.5 mL) was added CuI (10 mg, 0.05 mmol). The mixture was heated at 50 °C in an oil bath for 17 h under an Ar atmosphere. The reaction mixture was poured into a 10% NH_4_Cl solution and extracted with toluene. The combined organic layers were washed with brine, dried over Na_2_SO_4_, and concentrated under reduced pressure. The residue was purified by column chromatography on silica gel using toluene/AcOEt as eluent to afford **5g** (142 mg, 72%) as a red solid. M.p. 99–101 °C; IR (ATR): ν_max_ = 3144 (w), 2983 (w), 2954 (w), 2931 (w), 2855 (w), 1678 (s), 1591 (w), 1508 (m), 1459 (w), 1433 (s), 1415 (m), 1383 (m), 1355 (w), 1317 (w), 1298 (w), 1254 (m), 1221 (s), 1182 (s), 1132 (w), 1111 (w), 1059 (m), 1044 (s), 1027 (w), 880 (w), 820 (w), 793 (m), 743 (w), 716 (w), 687 (w), 669 (w), 660 (w) cm^−1^; UV/Vis (CH_2_Cl_2_): λ_max_ (log ε) = 273 (4.47), 292 sh (4.61), 303 (4.73), 363 (3.94), 495 sh (2.99), 514 (3.00) nm; ^1^H NMR (500 MHz, CDCl_3_): δ_H_ = 9.89 (dd, *J* = 10.7, 1.0 Hz, 2H), 8.11 (t, *J* = 9.7 Hz, 1H), 7.88 (dd, *J* = 10.7, 9.9 Hz, 2H), 7.62 (s, 1H), 4.16 (q, *J* = 7.2 Hz, 4H), 2.86 (t, *J* = 7.7 Hz, 2H), 1.80–1.74 (m, 2H), 1.49 (q, *J* = 7.4 Hz, 2H), 1.07 (t, *J* = 7.2 Hz, 6H), 0.98 (t, *J* = 7.3 Hz, 3H) ppm; ^13^C NMR (125 MHz, CDCl_3_): δ_C_ = 163.73, 146.86, 144.16, 142.58, 141.56, 131.76, 124.29, 113.18, 60.56, 31.91, 25.38, 22.52, 13.96, 13.92 ppm; HRMS (ESI-TOF, Positive): calcd for [C_22_H_25_N_3_O_4_ + H]^+^ 396.1918; found: 396.1927.

**Diethyl 6-(4-phenyl-1*H*-1,2,3-triazol-1-yl)azulene-1,3-dicarboxylate (6a):** To a degassed solution of **4** (269 mg, 0.86 mmol) and ethynylbenzene (182 mg, 1.78 mmol) in THF (4 mL) and triethylamine (4 mL) was added CuI (17 mg, 0.09 mmol). The mixture was heated at 50 °C in an oil bath for 18 h under an Ar atmosphere. The reaction mixture was poured into a 10% NH_4_Cl solution and extracted with toluene. The combined organic layers were washed with brine, dried over Na_2_SO_4_, and concentrated under reduced pressure. The residue was purified by column chromatography on silica gel using CH_2_Cl_2_/ethyl acetate as eluent to afford **6a** (299 mg, 84%) as a purple solid. M.p. 177–180 °C; IR (ATR): ν_max_ = 3116 (w), 3092 (w), 2976 (w), 2930 (w), 1692 (s), 1592 (w), 1550 (w), 1522 (w), 1480 (w), 1467 (w), 1437 (s), 1406 (w), 1393 (w), 1382 (w), 1362 (w), 1302 (w), 1237 (w), 1199 (s), 1173 (m), 1092 (w), 1080 (w), 1047 (w), 1036 (s), 988 (m), 909 (w), 867 (w), 842 (m), 820 (w), 766 (s), 708 (w), 691 (m), 671 (w), 656 (w) cm^−1^; UV/Vis (CH_2_Cl_2_): λ_max_ (log ε) = 239 (4.80), 265 sh (4.51), 309 (4.77), 336 (4.67), 374 sh (4.42), 522 (3.00), 565 sh (2.87), 623 (2.32) nm; ^1^H NMR (500 MHz, CDCl_3_): δ_H_ = 9.76 (d, *J* = 11.2 Hz, 2H), 8.90 (s, 1H), 7.93 (s, 1H), 7.77 (dd, *J* = 10.0, 1.1 Hz, 2H), 7.41–7.33 (m, 3H), 7.22–7.21 (m, 2H), 4.44 (q, *J* = 7.1 Hz, 4H), 1.45 (t, *J* = 7.2 Hz, 6H) ppm; ^13^C NMR (125 MHz, CDCl_3_): δ_C_ = 164.71, 145.14, 144.92, 142.96, 138.62, 137.91, 134.02, 129.92, 129.40, 128.78, 127.40, 126.15, 118.26, 60.52, 14.61 ppm; HRMS (ESI-TOF, Positive): calcd for [C_24_H_21_N_3_O_4_ + H]^+^ 416.1605, found: 416.1608.

**Diethyl 6-[4-(4-aminophenyl)-1*H*-1,2,3-triazol-1-yl]azulene-1,3-dicarboxylate (6b):** To a degassed solution of **4** (162 mg, 0.52 mmol) and 4-ethynylaniline (120 mg, 1.02 mmol) in THF (3 mL) and triethylamine (3 mL) was added CuI (11 mg, 0.06 mmol). The mixture was stirred at 50 °C for 17 h under an Ar atmosphere. The reaction mixture was poured into a 10% NH_4_Cl solution and extracted with toluene. The combined organic layers were washed with brine, dried over Na_2_SO_4_, and concentrated under reduced pressure. The residue was purified by column chromatography on silica gel using CH_2_Cl_2_/AcOEt as eluent to afford **6b** (192 mg, 86%) as a purple solid. M.p. 223–227 °C; IR (ATR): ν_max_ = 3432 (w), 3348 (w), 3095 (w), 2979 (w), 1701 (m), 1673 (m), 1633 (w), 1612 (w), 1589 (w), 1549 (w), 1498 (w), 1436 (s), 1406 (w), 1387 (m), 1298 (w), 1239 (w), 1200 (s), 1179 (m), 1091 (m), 1035 (s), 990 (m), 907 (w), 871 (w), 850 (w), 828 (m), 765 (m), 720 (w), 670 (w), 655 (w) cm^−1^; UV/Vis (CH_2_Cl_2_): λ_max_ (log ε) = 239 (4.32), 270 (4.30), 315 (4.50), 347 (3.89), 378 (3.90), 523 sh (2.78), 565 sh (2.62), 624 sh (2.13) nm; ^1^H NMR (500 MHz, DMSO-*d*_6_): δ_H_ = 9.74 (d, *J* = 11.2 Hz, 2H), 9.04 (s, 1H), 8.65 (s, 1H), 8.50 (d, *J* = 11.2 Hz, 2H), 7.63 (d, *J* = 8.3 Hz, 2H), 6.71 (d, *J* = 8.0 Hz, 2H), 4.98 (s, 2H), 4.41 (q, *J* = 7.2 Hz, 4H), 1.40 (t, *J* = 7.0 Hz, 6H) ppm; Low solubility of compound humped the measurement of ^13^C NMR; HRMS (ESI-TOF, Positive): calcd for [C_24_H_22_N_4_O_4_ + H]^+^ 431.1714, found: 431.1733.

**Diethyl 6-[4-(4-nitrophenyl)-1*H*-1,2,3-triazol-1-yl]azulene-1,3-dicarboxylate (6c):** To a degassed solution of 4 (159 mg, 0.51 mmol) and 4-ethynylnitrobenzene (81 mg, 0.55 mmol) in THF (3 mL) and triethylamine (3 mL) was added CuI (10 mg, 0.05 mmol). The mixture was heated at 50 °C in an oil bath for 17 h under an Ar atmosphere. The reaction mixture was poured into a 10% NH_4_Cl solution and extracted with toluene. The combined organic layers were washed with brine, dried over Na_2_SO_4_, and concentrated under reduced pressure. The residue was purified by column chromatography on silica gel using CH_2_Cl_2_/AcOEt as eluent to afford 6c (162 mg, 69%) as a purple solid. M.p. 258–262 °C; IR (ATR): ν_max_ = 3162 (w), 2979 (w), 1688 (m), 1606 (w), 1588 (w), 1550 (w), 1518 (m), 1429 (s), 1406 (w), 1389 (w), 1344 (m), 1246 (w), 1201 (s), 1110 (w), 1095 (w), 1080 (w), 1043 (m), 1019 (m), 996 (w), 963 (w), 912 (w), 868 (w), 855 (w), 810 (w), 768 (w), 755 (w), 708 (w), 690 (w), 657 (w) cm^−1^; UV/Vis (CH_2_Cl_2_): λ_max_ (log ε) = 237 (4.52), 268 (4.26), 333 (4.69), 374 sh (4.26), 523 (2.83), 570 sh (2.68), 630 sh (2.17) nm; ^1^H NMR (500 MHz, DMSO-*d*_6_): δ_H_ = 9.77 (dd, *J* = 10.0, 1.1 Hz, 2H), 9.57 (d, *J* = 2.0 Hz, 1H), 8.69 (s, 1H), 8.50 (dd, *J* = 11.3, 0.7 Hz, 2H), 8.33 (d, *J* = 8.9 Hz, 2H), 8.21 (dd, *J* = 6.9, 2.0 Hz, 2H), 4.41 (q, *J* = 7.1 Hz, 4H), 1.40 (t, *J* = 7.2 Hz, 6H) ppm; ^13^C NMR (125 MHz, DMSO-*d*_6_): δ_C_ = 164.27, 148.14, 146.76, 145.51, 143.52, 142.05, 138.79, 136.79, 127.11, 124.78, 123.68, 123.61, 118.35, 60.58, 14.75 ppm; HRMS (ESI-TOF, Positive): calcd for [C_24_H_20_N_4_O_6_ + H]^+^ 461.1456, found: 461.1436.

**Diethyl 6-[4-(naphthalen-1-yl)-1*H*-1,2,3-triazol-1-yl]azulene-1,3-dicarboxylate (6d):** To a degassed solution of 4 (143 mg, 0.46 mmol) and 1-ethynylnaphthalene (140 mg, 0.92 mmol) in THF (2.5 mL) and triethylamine (2.5 mL) was added CuI (10 mg, 0.05 mmol). The mixture was heated at 50 °C in an oil bath for 17 h under an Ar atmosphere. The reaction mixture was poured into a 10% NH_4_Cl solution and extracted with toluene. The combined organic layers were washed with brine, dried over Na_2_SO_4_, and concentrated under reduced pressure. The residue was purified by column chromatography on silica gel using CH_2_Cl_2_ as eluent to afford 6d (175 mg, 82%) as a purple solid. M.p. 179–182 °C; IR (ATR): ν_max_ = 3132 (w), 3060 (w), 2979 (w), 1685 (s), 1587 (w), 1551 (w), 1518 (w), 1435 (s), 1378 (w), 1350 (w), 1201 (s), 1128 (w), 1109 (w), 1086 (w), 1046 (m), 1021 (w), 993 (m), 912 (w), 874 (w), 859 (w), 797 (m), 769 (m), 742 (w), 714 (w), 674 (w), 662 (w), cm^−1^; UV/Vis (CH_2_Cl_2_): λ_max_ (log ε) = 270 (4.27), 316 (4.74), 358 sh (4.30), 375 sh (4.23), 522 (3.00), 565 (2.87), 625 sh (2.29) nm; ^1^H NMR (500 MHz, CDCl_3_): δ_H_ = 9.93 (dd, *J* = 10.0, 1.4 Hz, 2H), 8.89 (s, 1H), 8.46–8.43 (m, 2H), 8.30 (dd, *J* = 9.9, 1.3 Hz, 2H), 7.96–7.93 (m, 2H), 7.83 (dd, *J* = 6.9, 1.1 Hz, 1H), 7.60–7.54 (m, 3H), 4.47 (q, *J* = 7.2 Hz, 4H), 1.48 (t, *J* = 7.2 Hz, 6H) ppm; ^13^C NMR (125 MHz, CDCl_3_): δ_C_ = 164.76, 148.77, 145.05, 144.43, 142.43, 138.61, 134.04, 131.16, 129.74, 128.72, 127.69, 127.11, 127.04, 126.34, 125.46, 125.28, 122.62, 121.68, 118.43, 60.54, 14.63 ppm; HRMS (ESI-TOF, Positive): calcd for [C_28_H_23_N_3_O_4_ + H]^+^ 466.1761, found: 466.1747.

**Diethyl 6-{4-[5-isopropyl-3-(methoxycarbonyl)azulen-1-yl]-1*H*-1,2,3-triazol-1-yl}azulene-1,3-dicarboxylate (6e):** To a degassed solution of **4** (53 mg, 0.17 mmol) and 1-ethynyl-5-isopropyl-3-methoxycarbonylazulene (86 mg, 0.34 mmol) in THF (1 mL) and triethylamine (1 mL) was added CuI (3 mg, 0.016 mmol). The mixture was heated at 50 °C in an oil bath for 17 h under an Ar atmosphere. The reaction mixture was poured into a 10% NH_4_Cl solution and extracted with toluene. The combined organic layers were washed with brine, dried over Na_2_SO_4_, and concentrated under reduced pressure. The residue was purified by column chromatography on silica gel using toluene as eluent to afford **6e** (73 mg, 76%) as a purple solid. M.p. 170–174 °C; IR (ATR): ν_max_ = 2978 (w), 1686 (s), 1587 (w), 1571 (w), 1547 (w), 1522 (w), 1434 (s), 1421 (m), 1404 (m), 1239 (m), 1207 (s), 1171 (m), 1119 (m), 1082 (w), 1063 (w), 1041 (m), 1028 (m), 983 (m), 940 (w), 908 (w), 876 (m), 857 (m), 817 (w), 777 (m), 768 (w), 739 (w), 705 (w), 676 (w), 662 (w) cm^−1^; UV/Vis (CH_2_Cl_2_): λ_max_ (log ε) = 237 (4.79), 270 (4.51), 324 (4.98), 364 sh (4.41), 381 (4.12), 560 sh (2.85), 613 sh (2.52) nm; ^1^H NMR (500 MHz, CDCl_3_): δ_H_ = 9.92 (d, *J* = 11.2 Hz, 2H), 9.83 (d, *J* = 1.7 Hz, 1H), 9.55 (d, *J* = 9.7 Hz, 1H), 8.88 (s, 1H), 8.64 (s, 1H), 8.46 (s, 1H), 8.29 (dd, *J* = 10.0, 1.4 Hz, 2H), 7.85 (d, *J* = 10.3 Hz, 1H), 7.60 (t, *J* = 10.0 Hz, 1H), 4.47 (q, *J* = 7.2 Hz, 4H), 3.99 (s, 3H), 3.27 (t, *J* = 6.9 Hz, 1H), 1.48 (t, *J* = 7.2 Hz, 6H), 1.45 (d, *J* = 6.9 Hz, 6H) ppm; ^13^C NMR (125 MHz, CDCl_3_): δ_C_ = 165.74, 164.79, 150.27, 146.83, 145.09, 144.28, 142.50, 142.38, 140.16, 139.61, 139.12, 138.60, 138.54, 137.32, 128.34, 122.46, 119.03, 118.35, 115.58, 115.23, 60.52, 51.28, 39.21, 24.73, 14.65 ppm; HRMS (ESI-TOF, Positive): calcd for [C_33_H_31_N_3_O_6_ + H]^+^ 566.2286, found: 566.2260.

**Diethyl 6-(4-ferrocenyl-1*H*-1,2,3-triazol-1-yl)azulene-1,3-dicarboxylate (6f):** To a degassed solution of 4 (158 mg, 0.50 mmol) and ethynylferrocene (210 mg, 1.00 mmol) in THF (2.5 mL) and triethylamine (2.5 mL) was added CuI (10 mg, 0.05 mmol). The mixture was heated at 50 °C in an oil bath for 17 h under an Ar atmosphere. The reaction mixture was poured into a 10% NH_4_Cl solution and extracted with toluene. The combined organic layers were washed with brine, dried over Na_2_SO_4_, and concentrated under reduced pressure. The residue was purified by column chromatography on silica gel using CH_2_Cl_2_ as eluent to afford **6f** (194 mg, 74%) as a red solid. M.p. 247–250 °C; IR (ATR): ν_max_ = 3119 (w), 2975 (w), 1691 (s), 1598 (w), 1550 (w), 1436 (s), 1409 (w), 1390 (m), 1352 (w), 1306 (w), 1237 (m), 1194 (s), 1118 (w), 1078 (w), 1043 (m), 1036 (s), 1020 (m), 988 (m), 870 (m), 840 (m), 820 (m), 764 (m), 716 (w), 670 (w), 658 (w) cm^−1^; UV/Vis (CH_2_Cl_2_): λ_max_ (log ε) = 240 (4.41), 266 sh (4.11), 309 (4.38), 336 (4.27), 374 sh (4.03), 501 (2.99), 565 sh (2.73) nm; ^1^H NMR (500 MHz, CDCl_3_): δ_H_ = 9.90 (d, *J* = 11.5 Hz, 2H), 8.87 (s, 1H), 8.23 (d, *J* = 11.5 Hz, 2H), 8.08 (s, 1H), 4.83 (t, *J* = 1.7 Hz, 2H), 4.46 (q, *J* = 7.2 Hz, 4H), 4.39 (t, *J* = 1.9 Hz, 2H), 4.15 (s, 4H), 1.47 (t, *J* = 7.2 Hz, 6H) ppm; ^13^C NMR (125 MHz, CDCl_3_): δ_C_ = 164.79, 149.20, 145.03, 144.19, 142.30, 138.60, 122.27, 118.30, 117.32, 74.15, 69.80, 69.30, 67.04, 60.49, 14.63 ppm; HRMS (ESI-TOF, Positive): calcd for [C_28_H_25_FeN_3_O_4_ + H]^+^ 524.1267, found: 524.1282; [C_28_H_25_FeN_3_O_4_]^+^ 523.1189, found: 523.1216.

**Diethyl 6-(4-butyl-1*H*-1,2,3-triazol-1-yl)azulene-1,3-dicarboxylate (6g):** To a degassed solution of **4** (158 mg, 0.50 mmol) and 1-hexyne (84 mg, 1.02 mmol) in THF (5 mL) and triethylamine (5 mL) was added CuI (15 mg, 0.08 mmol). The mixture was heated at 50 °C in an oil bath for 17 h under an Ar atmosphere. The reaction mixture was poured into a 10% NH_4_Cl solution and extracted with toluene. The combined organic layers were washed with brine, dried over Na_2_SO_4_, and concentrated under reduced pressure. The residue was purified by column chromatography on silica gel using toluene as eluent to afford **6g** (129 mg, 65%) as a purple solid. M.p. 172–173 °C; IR (ATR): ν_max_ = 3125 (w), 2960 (w), 2931 (w), 2872 (w), 1693 (s), 1590 (w), 1549 (w), 1519 (w), 1479 (w), 1436 (s), 1409 (w), 1394 (w), 1381 (w), 1352 (w), 1325 (w), 1308 (w), 1236 (w), 1198 (s), 1164 (m), 1077 (m), 1039 (s), 1020 (w), 994 (m), 932 (w), 910 (w), 869 (w), 845 (w), 804 (w), 763 (m), 701 (w), 659 (w) cm^−1^; UV/Vis (CH_2_Cl_2_): λ_max_ (log ε) = 237 (4.24), 270 (3.96), 324 (4.43), 362 sh (3.88), 381 sh (3.57), 519 (2.73), 561 sh (2.53), 616 sh (1.93) nm; ^1^H NMR (500 MHz, CDCl_3_): δ_H_ = 9.87 (dd, *J* = 10.0, 1.4 Hz, 2H), 8.85 (s, 1H), 8.17 (dd, *J* = 10.0, 1.4 Hz, 2H), 7.92 (s, 1H), 4.45 (q, *J* = 7.2 Hz, 4H), 2.85 (t, *J* = 7.7 Hz, 2H), 1.79–1.73 (m, 2H), 1.48–1.44 (m, 8H), 0.98 (t, *J* = 7.4 Hz, 3H) ppm; ^13^C NMR (125 MHz, CDCl_3_): δ_C_ = 164.78, 150.46, 145.32, 144.14, 142.31, 138.58, 122.50, 119.97, 118.20, 60.47, 31.45, 25.46, 22.43, 14.62, 13.91 ppm; HRMS (ESI-TOF, Positive): calcd for [C_22_H_25_N_3_O_4_ + H]^+^ 396.1918, found: 396.1927.

**1-(Azulen-2-yl)-4-phenyl-1*H*-1,2,3-triazole (7a):** A solution of **5a** (104 mg, 0.25 mmol) in methanesulfonic acid (3 mL) was heated at 90 °C in an oil bath for 2 h. The reaction mixture was poured into water. The precipitate was collected by filtration to afford **7a** (63 mg, 93%) as a purple solid. M.p. 228–231 °C; IR (ATR): ν_max_ = 3142 (m), 3115 (w), 3084 (w), 3055 (w), 3023 (w), 1575 (m), 1536 (m), 1522 (s), 1477 (m), 1452 (m), 1421 (m), 1399 (m), 1299 (w), 1230 (m), 1223 (m), 1213 (m), 1172 (w), 1155 (w), 1071 (w), 1040 (m), 1016 (m), 959 (w), 915 (w), 899 (m), 810 (m), 760 (m), 736 (m), 706 (w), 692 (m), 660 (w), 632 (w) cm^−1^; UV/Vis (CH_2_Cl_2_): λ_max_ (log ε) = 239 (4.33), 285 (4.75), 301 (4.64), 344 (3.85), 373 (4.17), 549 (2.65), 569 (2.63), 582 (2.64), 640 sh (2.24) nm; ^1^H NMR (400 MHz, CDCl_3_): δ_H_ = 8.40 (d, *J* = 9.2 Hz, 2H), 8.38 (s, 1H), 7.96 (dd, *J* = 8.3, 1.3 Hz, 2H), 7.70–7.64 (m, 3H), 7.50–7.46 (m, 2H), 7.41–7.36 (m, 1H), 7.33 (t, *J* = 9.9 Hz, 2H) ppm; ^13^C NMR (100 MHz, CDCl_3_): δ_C_ = 148.44, 142.64, 140.04, 137.76, 137.62, 130.24, 129.03, 128.57, 126.01, 125.33, 117.96, 106.28 ppm; HRMS (ESI-TOF, Positive): calcd for [C_18_H_13_N_3_ + H]^+^ 272.1182; found: 272.1210.

**4-[1-(Azulen-2-yl)-1*H*-1,2,3-triazol-4-yl]aniline (7b):** A solution of **5b** (108 mg, 0.25 mmol) in methanesulfonic acid (3 mL) was heated at 90 °C in an oil bath for 2 h. The reaction mixture was poured into water. The precipitate was collected by filtration to afford **7b** (63 mg, 88%) as a purple solid. M.p. 237–239 °C; IR (ATR): ν_max_ = 3464 (w), 3333 (s), 3210 (w), 1625 (s), 1612 (m), 1579 (w), 1535 (w), 1519 (m), 1489 (s), 1441 (w), 1417 (m), 1397 (w), 1296 (m), 1230 (m), 1218 (w), 1202 (w), 1184 (w), 1169 (w), 1128 (w), 1041 (m), 1020 (w), 958 (w), 946 (w), 901 (w), 833 (m), 801 (s), 789 (s), 769 (w), 729 (m), 700 (w), 657 (w), 630 (w) cm^−1^; UV/Vis (CH_2_Cl_2_): λ_max_ (log ε) = 237 (4.30), 292 (4.93), 380 (4.16), 510 sh (2.55), 547 (2.69), 582 (2.66), 638 sh (2.30) nm; ^1^H NMR (400 MHz, CDCl_3_): δ_H_ = 8.40–8.38 (m, 2H), 8.24 (s, 1H), 7.76–7.74 (m, 2H), 7.68–7.63 (m, 3H), 7.32 (t, *J* = 9.8 Hz, 2H), 6.79–6.77 (m, 2H), 3.81 (s, 2H) ppm; ^13^C NMR (100 MHz, CDCl_3_): δ_C_ = 148.77, 146.85, 142.85, 140.05, 137.54, 137.42, 127.26, 125.25, 120.67, 116.58, 115.37, 106.24 ppm; HRMS (ESI-TOF, Positive): [C_18_H_14_N_4_ + H]^+^ 287.1291; found: 287.1290.

**1-(Azulen-2-yl)-4-(4-nitrophenyl)-1*H*-1,2,3-triazole (7c):** A solution of **5c** (115 mg, 0.25 mmol) in methanesulfonic acid (3 mL) was heated at 90 °C in an oil bath for 2 h. The reaction mixture was poured into water. The precipitate was collected by filtration to afford **7c** (75 mg, 95%) as a purple solid. M.p. 321–323 °C; IR (ATR): ν_max_ = 3134 (w), 3102 (w), 3053 (w), 1604 (s), 1579 (w), 1536 (m), 1508 (s), 1477 (s), 1453 (m), 1415 (m), 1398 (m), 1376 (w), 1337 (s), 1315 (s), 1296 (s), 1272 (m), 1236 (s), 1222 (s), 1208 (s), 1199 (s), 1164 (w), 1109 (s), 1032 (m), 1016 (s), 1006 (s), 962 (m), 899 (w), 860 (s), 852 (m), 836 (m), 793 (s), 769 (m), 752 (s), 742 (s), 703 (m), 688 (m), 652 (w), 627 (m) cm^−1^; UV/Vis (CH_2_Cl_2_): λ_max_ (log ε) = 299 (4.65), 357 (4.35), 373 (4.45), 549 (2.70), 582 (2.68), 637 sh (2.31) nm; ^1^H NMR (400 MHz, CDCl_3_): δ_H_ = 8.53 (s, 1H), 8.44 (d, *J* = 9.2 Hz, 2H), 8.36 (dd, *J* = 7.0, 2.0 Hz, 2H), 8.14 (dd, *J* = 6.9, 2.0 Hz, 2H), 7.74–7.69 (m, 3H), 7.36 (t, *J* = 9.9 Hz, 2H) ppm; Low solubility of compound hampered the measurement of ^13^C NMR; HRMS (ESI-TOF, Positive): [C_18_H_12_N_4_O_2_ + H]^+^ 317.1033; found: 317.1062.

**1-(Azulen-2-yl)-4-(naphthalen-1-yl)-1*H*-1,2,3-triazole (7d):** A solution of **5d** (116 mg, 0.25 mmol) in methanesulfonic acid (3 mL) was heated at 90 °C in an oil bath for 2 h. The reaction mixture was poured into water, and the resulting solid was collected by filtration to afford **7d** (73 mg, 91%) as a purple solid. M.p. 159–161 °C; IR (ATR): ν_max_ = 3115 (m), 3045 (m), 3016 (w), 1581 (m), 1536 (m), 1503 (s), 1454 (m), 1392 (m), 1338 (w), 1298 (w), 1277 (w), 1258 (w), 1237 (m), 1218 (m), 1201 (m), 1163 (w), 1105 (w), 1034 (m), 1010 (m), 955 (w), 900 (w), 861 (w), 836 (m), 813 (m), 792 (s), 769 (s), 731 (s), 716 (m), 658 (w), 637 (w), 625 (m) cm^−1^; UV/Vis (CH_2_Cl_2_): λ_max_ (log ε) = 289 (4.80), 340 (3.90), 358 (4.02), 372 (4.18), 549 (2.66), 582 (2.65), 639 sh (2.27) nm; ^1^H NMR (400 MHz, CDCl_3_): δ_H_ = 8.49–8.46 (m, 1H), 8.45–8.42 (m, 3H), 7.95–7.93 (m, 2H), 7.85 (dd, *J* = 7.1, 1.2 Hz, 1H), 7.74 (s, 2H), 7.69 (t, *J* = 9.9 Hz, 1H), 7.60–7.55 (m, 3H), 7.35 (t, *J* = 9.9 Hz, 2H) ppm; ^13^C NMR (100 MHz, CDCl_3_): δ_C_ = 147.62, 142.63, 140.10, 137.82, 137.70, 134.00, 131.26, 129.30, 128.62, 127.69, 127.52, 126.89, 126.20, 125.50, 125.36, 121.05, 106.34 ppm, one signal is overlapped with other signals; HRMS (ESI-TOF, Positive): [C_22_H_15_N_3_ + H]^+^ 322.1339; found: 322.1357.

**1-(Azulen-2-yl)-4-(azulen-1-yl)-1*H*-1,2,3-triazole (7e):** A solution of **5e** (113 mg, 0.20 mmol) in methanesulfonic acid (5 mL) was heated at 90 °C in an oil bath for 2 h. The reaction mixture was poured into water, and the resulting solid was collected by filtration to afford **7e** (65 mg, 89%) as a purple solid. M.p. 252–254 °C; IR (ATR): ν_max_ = 3136 (m), 3112 (m), 3084 (m), 3045 (m), 3023 (m), 3004 (m), 2958 (s), 2922 (m), 2884 (m), 2865 (m), 1580 (m), 1562 (m), 1536 (m), 1522 (s), 1502 (m), 1484 (m), 1453 (m), 1428 (m), 1394 (s), 1363 (m), 1325 (w), 1295 (m), 1261 (m), 1247 (w), 1220 (m), 1176 (m), 1161 (w), 1106 (m), 1070 (w), 1054 (w), 1040 (m), 1004 (m), 978 (w), 955 (w), 943 (w), 919 (w), 908 (w), 900 (m), 886 (w), 870 (w), 859 (w), 820 (m), 802 (s), 770 (s), 730 (m), 700 (m), 632 (m) cm^−1^; UV/Vis (CH_2_Cl_2_): λ_max_ (log ε) = 299 (4.89), 341 (4.12), 386 (4.22), 550 (3.15), 585 (3.15), 655 sh (2.93) nm; ^1^H NMR (400 MHz, CDCl_3_): δ_H_ = 9.28 (d, *J* = 9.9 Hz, 1H), 8.40 (d, *J* = 10.2 Hz, 3H), 8.33 (d, *J* = 1.7 Hz, 1H), 8.21 (d, *J* = 4.0 Hz, 1H), 7.72 (s, 2H), 7.67–7.59 (m, 2H), 7.37–7.28 (m, 4H), 3.12 (t, *J* = 6.9 Hz, 1H), 1.40 (d, *J* = 6.9 Hz, 6H) ppm; ^13^C NMR (100 MHz, CDCl_3_): δ_C_ = 146.43, 144.27, 143.21, 142.83, 140.33, 137.39, 137.32, 136.06, 135.62, 135.23, 125.24, 124.15, 117.97, 117.45, 117.28, 106.52, 38.55, 24.53 ppm; HRMS (ESI-TOF, Positive): [C_25_H_21_N_3_ + H]^+^ 364.1808; found: 364.1826.

**1-(Azulen-2-yl)-4-butyl-1*H*-1,2,3-triazole (7g):** A solution of **5g** (79 mg, 0.20 mmol) in methanesulfonic acid (5 mL) was heated at 90 °C in an oil bath for 3 h. The reaction mixture was poured into water, and the resulting solid was collected by filtration to afford **7g** (45 mg, 90%) as a purple solid. M.p. 97–100 °C; IR (ATR): ν_max_ = 3142 (m), 3115 (m), 3084 (w), 3055 (w), 3023 (w), 1575 (m), 1536 (s), 1522 (s), 1477 (m), 1452 (m), 1421 (m), 1399 (s), 1299 (w), 1230 (m), 1223 (m), 1213 (m), 1172 (m), 1155 (w), 1071 (w), 1040 (m), 1016 (s), 959 (w), 915 (w), 899 (m), 810 (s), 760 (m), 736 (m), 706 (w), 692 (s), 660 (w), 632 (m) cm^−1^; UV/Vis (CH_2_Cl_2_): λ_max_ (log ε) = 239 (4.21), 290 (4.78), 298 (4.77), 340 (3.82), 354 (3.94), 370 (4.10), 548 sh (2.65), 581 (2.63), 635 sh (2.28) nm; ^1^H NMR (400 MHz, CDCl_3_): δ_H_ = 8.36 (d, *J* = 9.1 Hz, 2H), 7.89 (s, 1H), 7.66–7.60 (m, 3H), 7.29 (t, *J* = 10.1 Hz, 2H), 2.83 (t, *J* = 7.5 Hz, 2H), 1.74 (quint, *J* = 7.5 Hz, 2H), 1.44 (sextet, *J* = 7.5 Hz, 2H), 0.96 (t, *J* = 7.5 Hz, 3H) ppm; ^13^C NMR (100 MHz, CDCl_3_): δ_C_ = 149.30, 143.06, 140.07, 137.43, 137.32, 125.19, 119.34, 106.21, 31.55, 25.45, 22.42, 13.95 ppm; HRMS (ESI-TOF, Positive): [C_16_H_17_N_3_ + H]^+^ 252.1495; found: 252.1497.

**1-(Azulen-2-yl)-4-(azulen-1-yl)-1*H*-1,2,3-triazole (8):** A solution of **5e** (208 mg, 0.50 mmol) in methanesulfonic acid (5 mL) was heated at 90 °C in an oil bath for 1 h. The reaction mixture was poured into water, and the resulting solid was collected by filtration. The crude solid was dissolved in CH_2_Cl_2_ and purified by column chromatography on silica gel using CH_2_Cl_2_ as eluent to afford **8** (9 mg, 7%) as a purple solid. M.p. 228–231 °C; IR (ATR): ν_max_ = 3125 (m), 3093 (w), 3080 (w), 3058 (w), 1574 (m), 1551 (m), 1477 (m), 1454 (m), 1422 (m), 1398 (m), 1379 (m), 1346 (m), 1308 (m), 1286 (m), 1239 (m), 1211 (m), 1197 (m), 1161 (m), 1085 (m), 1073 (m), 1050 (m), 1032 (m), 1019 (m), 977 (m), 965 (m), 916 (m), 905 (m), 837 (m), 821 (m), 792 (m), 764 (s), 740 (s), 706 (m), 689 (s), 662 (m), 644 (m) cm^−1^; ^1^H NMR (400 MHz, CDCl_3_): δ_H_ = 8.46 (d, *J* = 10.3 Hz, 2H), 8.30 (d, *J* = 0.6 Hz, 1H), 8.00 (t, *J* = 3.8 Hz, 1H), 7.95–7.93 (m, 2H), 7.65 (d, *J* = 10.6 Hz, 2H), 7.54 (d, *J* = 3.7 Hz, 2H), 7.48 (t, *J* = 7.7 Hz, 2H), 7.41–7.37 (m, 1H) ppm; ^13^C NMR (100 MHz, CDCl_3_): δ_C_ = 148.81, 142.82, 139.18, 138.78, 135.46, 130.11, 129.09, 128.71, 125.99, 120.74, 119.10, 116.23 ppm; HRMS (MALDI-TOF, Positive): calcd for [C_18_H_13_N_3_ + H]^+^ 272.1182, found: 272.1175.

**Diethyl 6-[(triphenyl-λ_5_-phosphaneylidene)amino]azulene-1,3-dicarboxylate (9):** To a degassed solution of **4** (605 mg, 1.93 mmol) in THF (10 mL) was added triphenylphosphane (1.02 g, 3.89 mmol). The mixture was heated at 50 °C in an oil bath for 17 h under an Ar atmosphere. The generated solid was filtered and washed with EtOH and hexane to afford **9** (908 mg, 86%) as a yellow solid. M.p. 175–178 °C; IR (ATR): ν_max_ = 2980 (w), 1686 (w), 1666 (m), 1579 (w), 1562 (w), 1535 (w), 1479 (w), 1459 (w), 1425 (s), 1368 (w), 1323 (s), 1241 (w), 1196 (s), 1142 (w), 1106 (m), 1094 (m), 1047 (m), 999 (w), 952 (s), 889 (s), 868 (w), 846 (w), 828 (w), 806 (w), 761 (w), 721 (m), 693 (m), 669 (w), 655 (w) cm^−1^; ^1^H NMR (500 MHz, CDCl_3_): δ_H_ = 9.11 (d, *J* = 10.9 Hz, 2H), 8.22 (s, 1H), 7.77–7.73 (m, 6H), 7.62–7.59 (m, 3H), 7.53–7.50 (m, 6H), 7.14 (d, *J* = 11.7 Hz, 2H), 4.33 (q, *J* = 7.1 Hz, 4H), 1.38 (t, *J* = 7.2 Hz, 6H) ppm; ^13^C NMR (125 MHz, CDCl_3_): δ_C_ = 168.86, 168.84, 166.07, 137.43, 137.12, 134.80, 132.90, 132.88, 132.68, 132.60, 129.33, 129.23, 128.45, 127.84, 127.68, 127.65, 114.58, 59.34, 14.71 ppm; HRMS (ESI-TOF, Positive): calcd for [C_34_H_30_NO_4_P + H]^+^ 548.1985; found: 548.1978.

**Diethyl 6-aminoazulene-1,3-dicarboxylate (10):** To a solution of **9** (551 mg, 1.01 mmol) in THF (20 mL) was added hydrochloric acid (2 mL). The mixture was heated at reflux temperature in an oil bath for 17 h. The reaction mixture was poured into water and neutralized with K_2_CO_3_. The resulting solid was collected by filtration and washed with EtOH and hexane to afford **10** (235 mg, 81%) as a yellow solid. M.p. 252–254 °C (lit. 236–237 °C); ^1^H NMR (500 MHz, CDCl_3_): δ_H_ = 9.41 (d, *J* = 11.2 Hz, 2H), 8.34 (s, 1H), 6.87 (d, *J* = 11.5 Hz, 2H), 5.05 (s, 2H), 4.38 (q, *J* = 7.1 Hz, 4H), 1.42 (t, *J* = 7.2 Hz, 6H) ppm; ^13^C NMR (125 MHz, CDCl_3_): δ_C_ = 165.78, 159.38, 139.96, 137.49, 136.51, 132.26, 128.62, 116.50, 77.10, 59.70, 14.70 ppm; HRMS (ESI-TOF, Positive): calcd for [C_16_H_17_NO_4_ + H]^+^ 288.1230; found: 288.1253.

## 4. Conclusions

In summary, we have demonstrated that both 2- and 6-azidoazulenes serve as highly efficient dipoles for Cu(I)-catalyzed Huisgen cycloaddition with terminal alkynes, enabling rapid access to structurally diverse 1,2,3-triazole-functionalized azulenes. Subsequent transformations, including acid-mediated decarboxylation and the Staudinger reaction, further expanded the synthetic utility of these intermediates to furnish 2-azulenyltriazoles and 6-aminoazulene derivative.

Single-crystal X-ray diffraction analysis revealed that steric congestion around the azulene 1,3-positions enforces near-orthogonality between the azulene and triazole rings, whereas partial planarization is achieved in decarboxylated derivatives, providing direct structural insight into the control of π-conjugation within these scaffolds. UV/Vis and fluorescence measurements of a series of **7** disclosed pronounced effects of protonation, leading to distinct spectral changes and, in some cases, fluorescence with markedly enhanced quantum yields.

Taken together, these results establish azidoazulenes as versatile building blocks for modular construction of triazole-based π-extended azulenes, and reveal that fine control of substitution pattern, conformation, and protonation state provides an effective design handle for tuning their optical responses. We anticipate that these findings will contribute to the future development of azulene-based functional chromophores and electro-/photoresponsive organic materials.

## Figures and Tables

**Table 1:** Huisgen cycloaddition of 2-azidoazulene **3**.

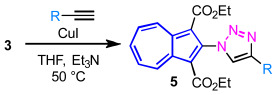				
Entry	Substrate	R	Product	Yield [%] ^1^
1	**3a**	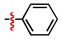	**5a**	77
2	**3b**	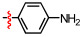	**5b**	75
3	**3c**	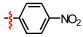	**5c**	77
4	**3d**	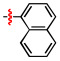	**5d**	74
5	**3e**	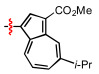	**5e**	88
6	**3f**	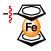	**5f**	85
7	**3g**	*n*-Bu	**5g**	72

^1^ Isolated yield.

**Table 2:** Huisgen cycloaddition of 6-azidoazulene derivative **4**.

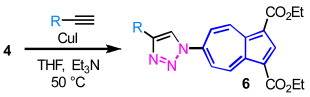				
Entry	Substrate	R	Product	Yield [%] ^1^
1	**4a**	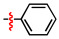	**6a**	84
2	**4b**	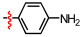	**6b**	86
3	**4c**	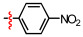	**6c**	69
4	**4d**	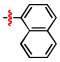	**6d**	82
5	**4e**	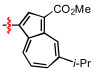	**6e**	76
6	**4f**	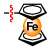	**6f**	74
7	**4g**	*n*-Bu	**6g**	65

^1^ Isolated yield.

**Table 3:** Decarboxylation of **5** to 2-(1,2,3-triazol-1-yl)azulenes **7**.

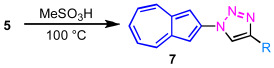				
Entry	Substrate	Product	R	Yield [%] ^1^
1	**5a**	**7a**	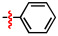	93
2	**5b**	**7b**	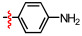	88
3	**5c**	**7c**	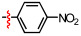	95
4	**5d**	**7d**	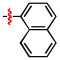	91
5	**5e**	**7e’**	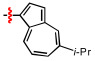	89
6	**5f**	**7f**	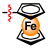	Decomp.
7	**5g**	**7g**	*n*-Bu	90

^1^ Isolated yield.

**Table 4:** The maximum absorption wavelength (λ_max_) and molar absorption coefficient (log ε) of **5** and **6** in CH_2_Cl_2_.

Sample	λ_max_ [nm] (log ε)
**5a**	495 sh (2.99), 513 (3.00), 570 sh (2.60)
**5b**	499 (3.00), 524 sh (2.98), 574 (2.57)
**5c**	493 sh (2.98), 512 (3.00)
**5d**	493 sh (2.99), 508 (3.00)
**5e**	403 sh (4.03), 522 (3.18), 570 (3.03)
**5f**	490 (3.30), 517 sh (3.26)
**5g**	495 sh (2.99), 514 (3.00)
**6a**	522 (3.00), 565 sh (2.87), 623 (2.32)
**6b**	523 sh (2.78), 565 sh (2.62), 624 sh (2.13)
**6c**	523 (2.83), 570 sh (2.68), 630 sh (2.17)
**6d**	522 (3.00), 565 (2.87), 625 sh (2.29)
**6e**	560 sh (2.85), 613 sh (2.52)
**6f**	501 (2.99), 565 sh (2.73)
**6g**	519 (2.73), 561 sh (2.53), 616 sh (1.93)

**Table 5:** The maximum absorption wavelength (λ_max_), molar absorption coefficient (log ε), maximum fluorescence wavelength (λ_Flu_), and fluorescence quantum yield (Φ_Flu_) of **7** and **8** in 10% TFA/CH_2_Cl_2_.

Sample	λ_max_ [nm] (log ε) in 10% TFA/CH_2_Cl_2_.	λ_Flu_ [nm]	Φ_Flu_ [%]
**7a**	421 (2.96), 558 (2.93), 597 (2.96), 651 sh (2.72)	475, 489, 540	<1
**7b**	410 (3.51), 554 (2.84), 593 (2.85), 651 sh (2.59)	491, 534	<1
**7c**	412 (3.84), 472 (3.47), 598 (2.79), 649 sh (2.57)	492, 533	27.0
**7d**	548 (2.89), 594 (2.92), 645 sh (2.66)	463, 489, 529	<1
**7e**	558 (3.12), 593 (3.10), 651 sh (2.81)	472, 523	2.3
**7g**	555 (2.87), 596 (2.90), 651 sh (2.65)	472, 530	10.8
**8**	580 sh (2.65), 619 (2.74), 676 sh (2.64), 754 sh (2.19)	476, 490, 539	<1

## Data Availability

Data is available from the corresponding author.

## Supplementary Materials

The following supporting information can be downloaded at: https://www.mdpi.com/article/10.3390/molecules31020221/s1, Figures S1–S114: ^1^H, ^13^C NMR, UV/Vis and fluorescent spectra of new compounds; Table S1: Selected crystal data of 5a, 5d, 7a, 8, and 9.
