# Supplementary material for: Huisgen Cycloaddition of Azidoazulenes: Synthesis, Structural and Optical Properties of 2- and 6-(1,2,3-Triazol-1-yl)azulenes
Source: Molecules. 2026 Jan 8;31(2):221. doi: 10.3390/molecules31020221 (PMC12843792; doi:10.3390/molecules31020221)
Supplement: Supplementary file 1 [file molecules-31-00221-s001.zip › molecules-4069052-supplementary.pdf]

Supporting Information

Huisgen cycloaddition of azidoazulenes: Synthesis, structural and optical properties of 2- and 6-(1,2,3-triazol-1-yl)azulenes

Taku Shoji[1,*], Miku Yoshida[1], Masayuki Iwabuchi[1], Mitsuki Furuhata[1], Shigeki Mori[2], Tetsuo Okujima[3], Ikumi Uchiyama[4], Ryuta Sekiguchi[4] and Shunji Ito[4]

[1] Department of Chemical Biology and Applied Chemistry, College of Engineering, Nihon University, Koriyama 963-8642, Japan. E-mail: shoji.taku@nihon-u.ac.jp

[2] Advanced Research Support Center, Ehime University, Matsuyama 790-8577, Japan.

[3] Graduate School of Science and Engineering, Ehime University, Matsuyama 790-8577, Japan.

[4] Graduate School of Science and Technology, Hirosaki University, Hirosaki 036-8561, Japan.

➢ ***Contents***

1. Copies of 1H NMR, $^{13}C\{^{1}H\}$ NMR, COSY and HRMS of reported compounds. (Figures S1−S83). S1−S42

2. UV/Vis and fluorescent spectra of reported compounds (Figures S84−S109). S43−S56

3. ORTEP diagrams of reported compounds (Figures S110−S114) S56−S61

1. Copies of ^{1}H NMR, ^{13}C{^{1}H} NMR, COSY and HRMS of reported compounds.

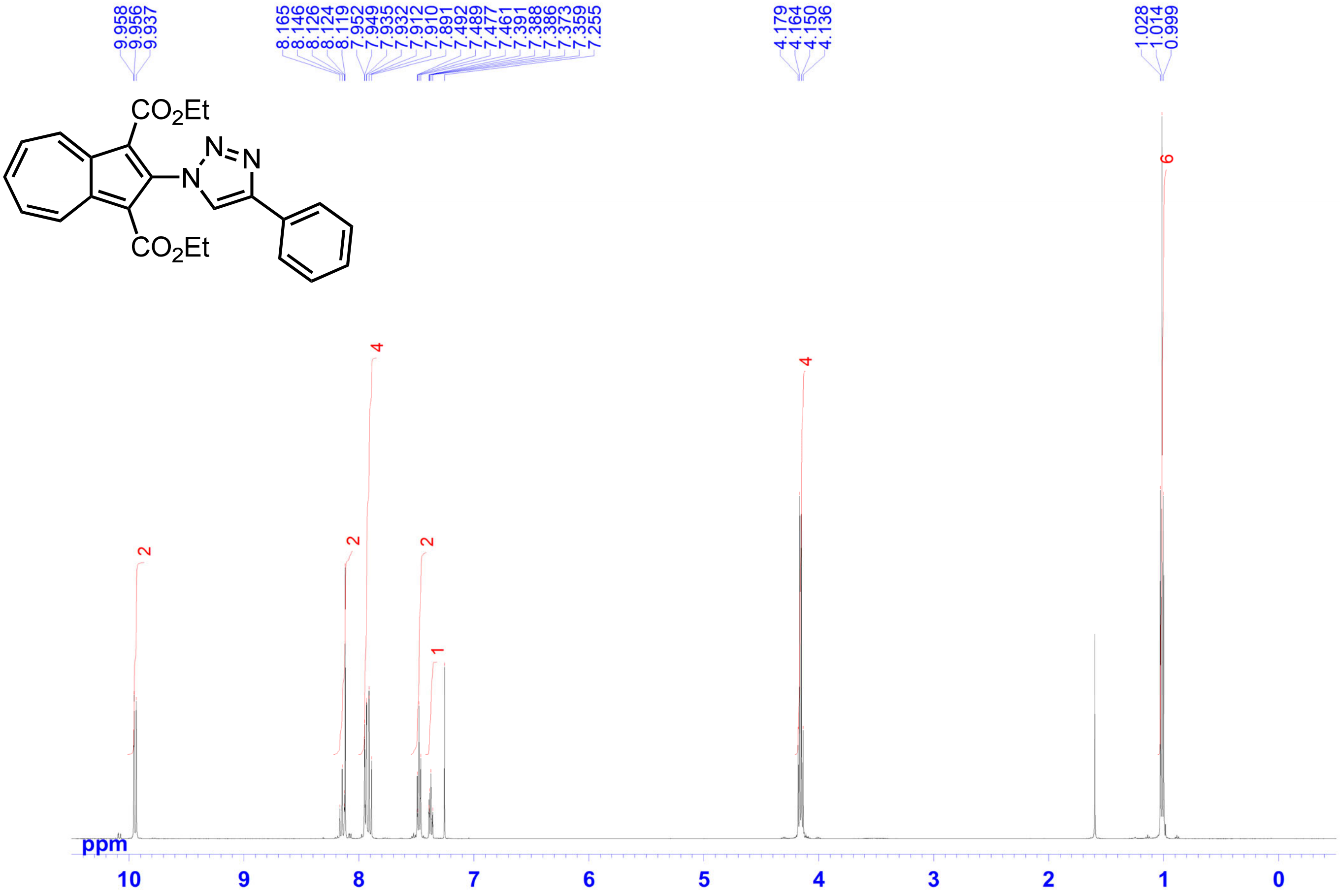

Figure S1. ^{1}H NMR spectrum of **5a** in $CDCl_3$ (500 MHz).

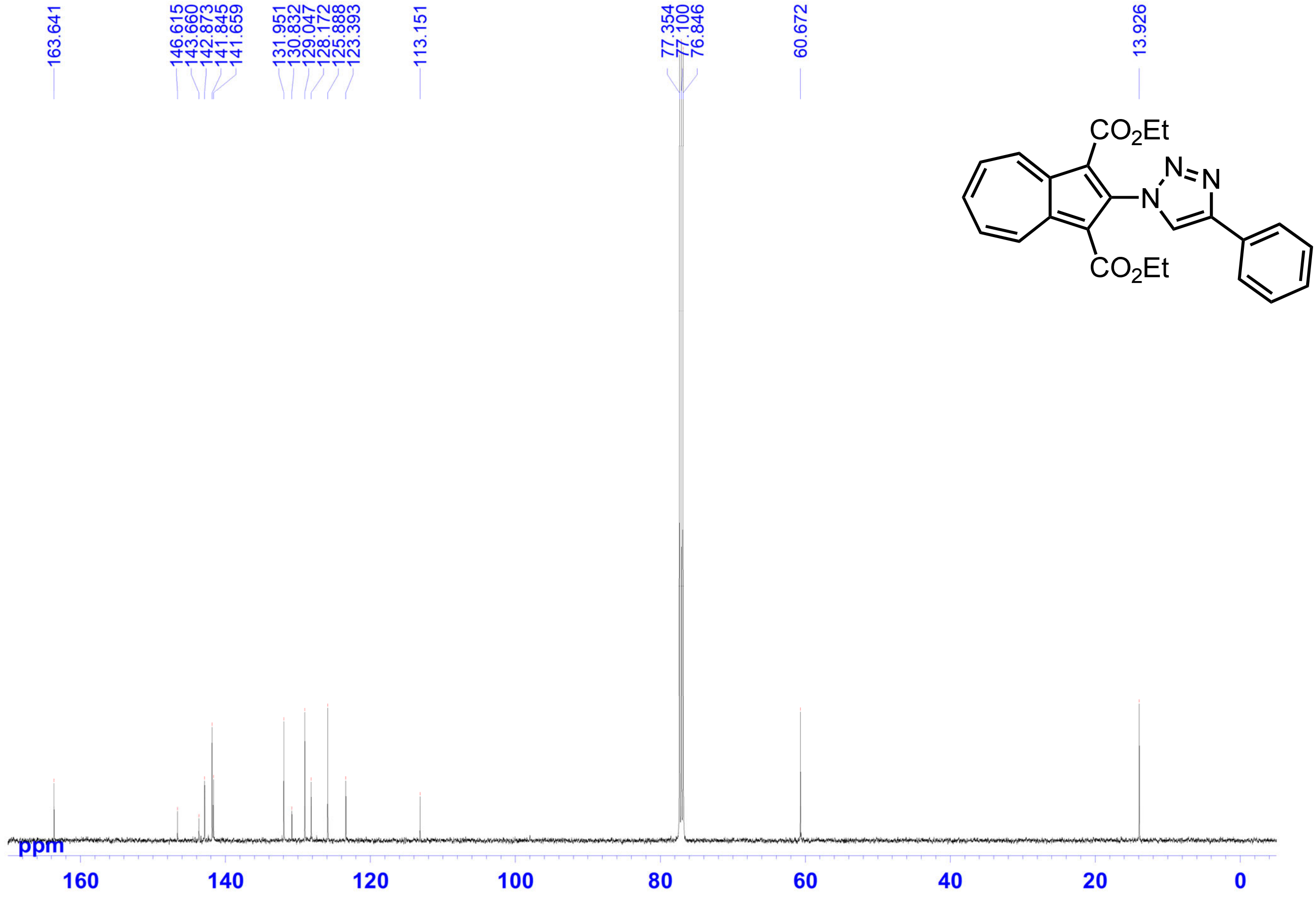

Figure S2. ^{13}C{^{1}H} NMR spectrum of **5a** in $CDCl_3$ (126 MHz).

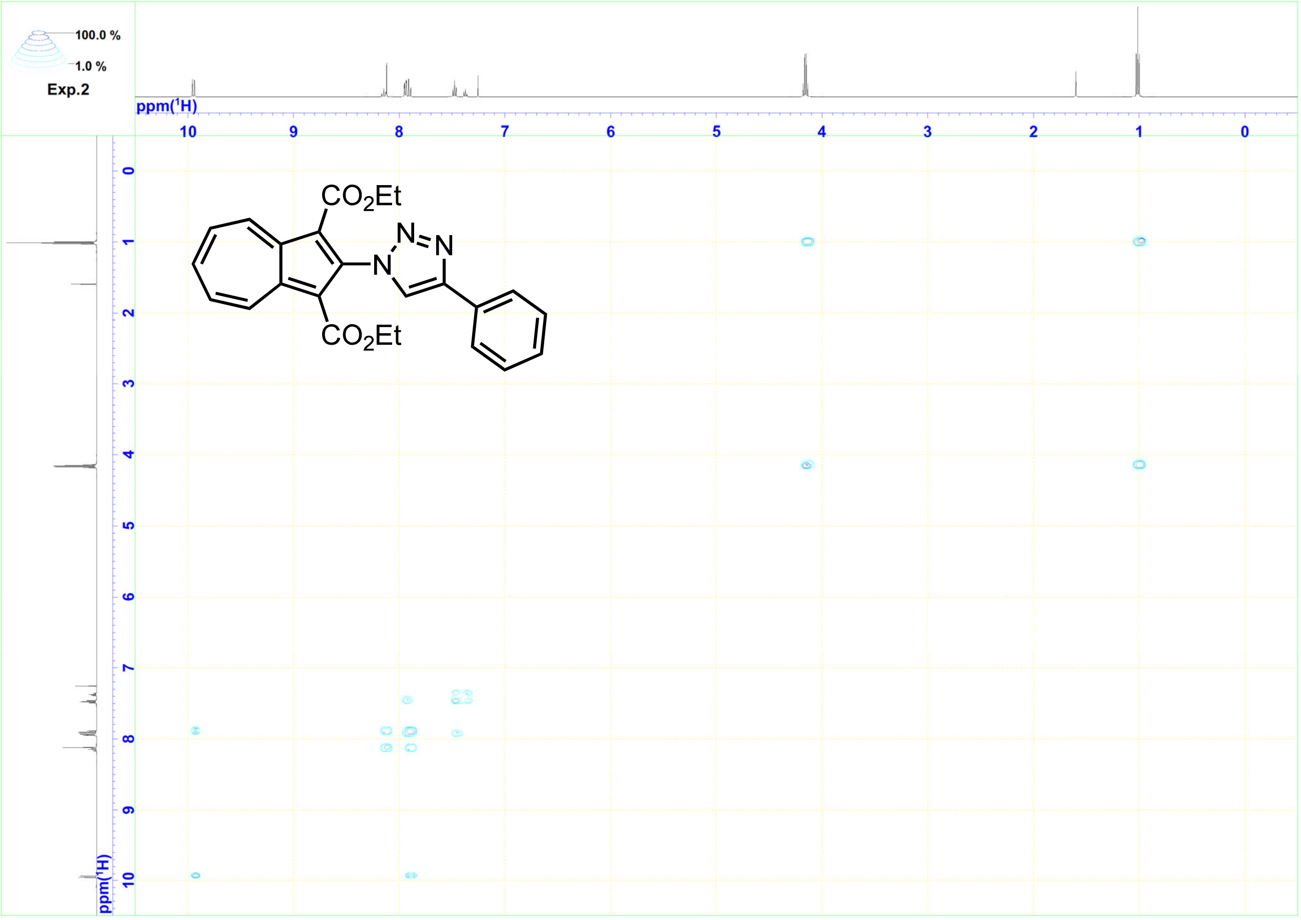

Figure S3. COSY spectrum of **5a** in CDCl3 (500 MHz).

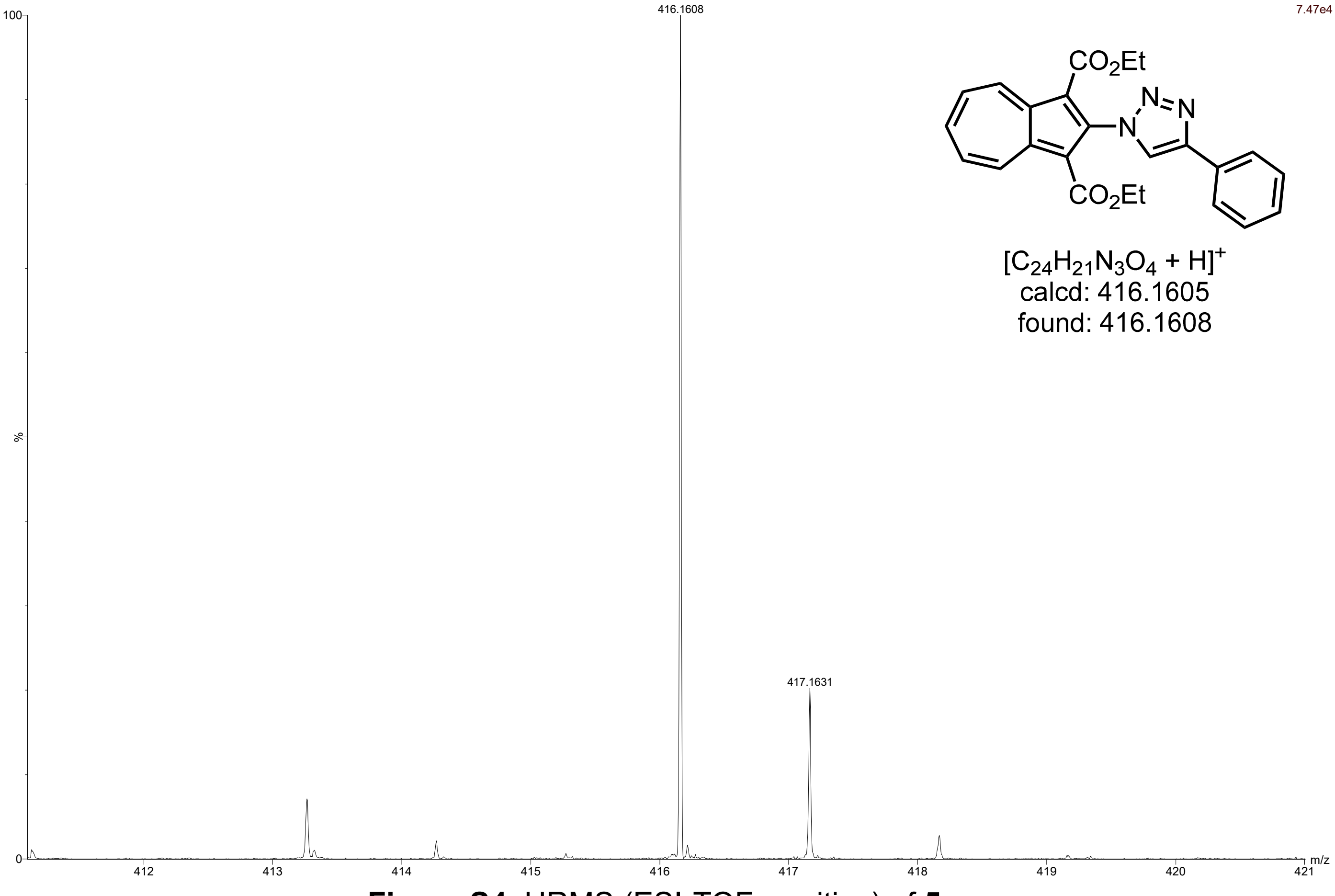

Figure S4. HRMS (ESI-TOF, positive) of **5a**.

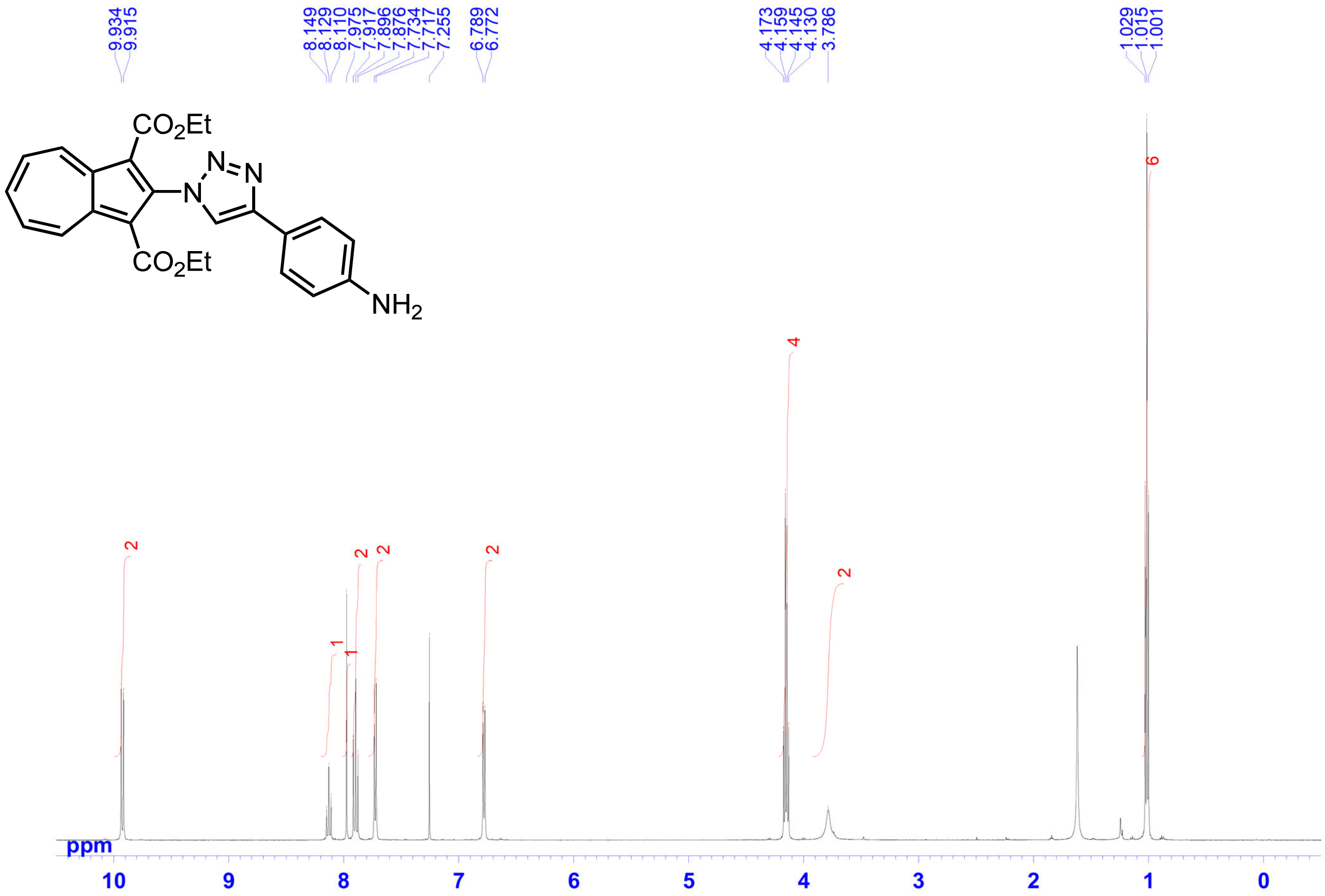

Figure S5. ^{1}H NMR spectrum of **5b** in $CDCl_3$ (500 MHz).

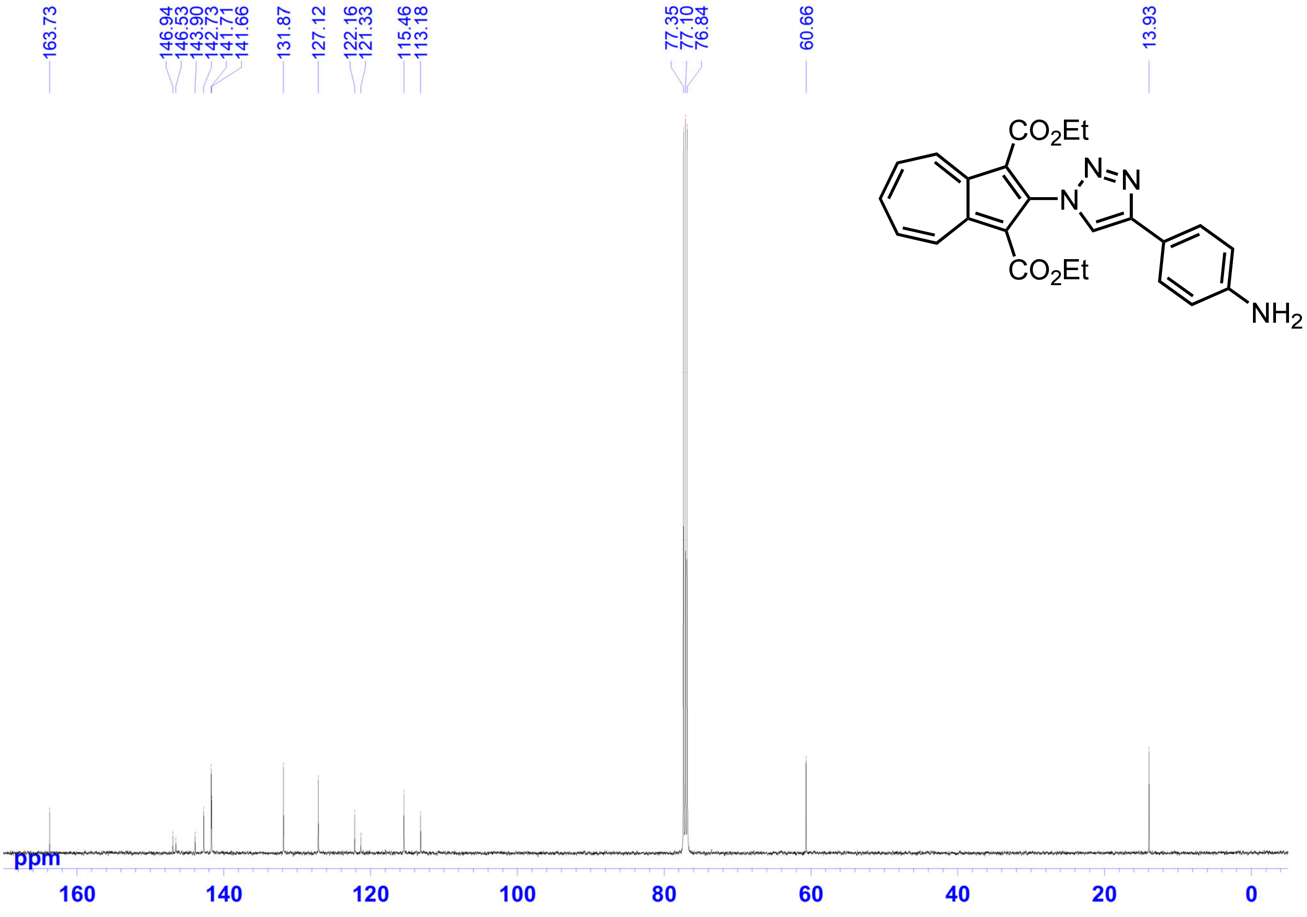

Figure S6. $^{13}C\{^{1}H\}$ NMR spectrum of **5b** in $CDCl_3$ (126 MHz).

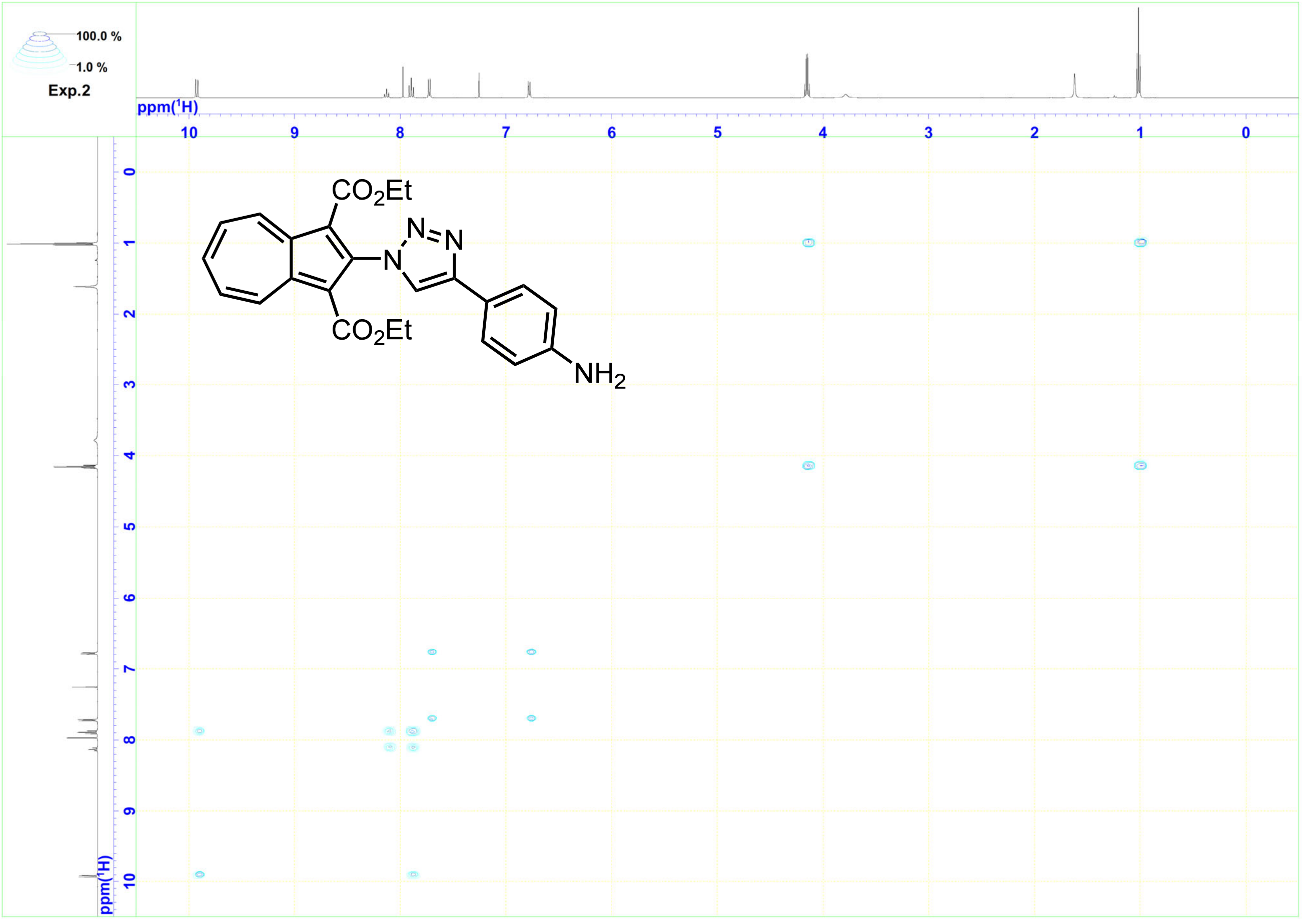

Figure S7. COSY spectrum of **5b** in $CDCl_3$ (500 MHz).

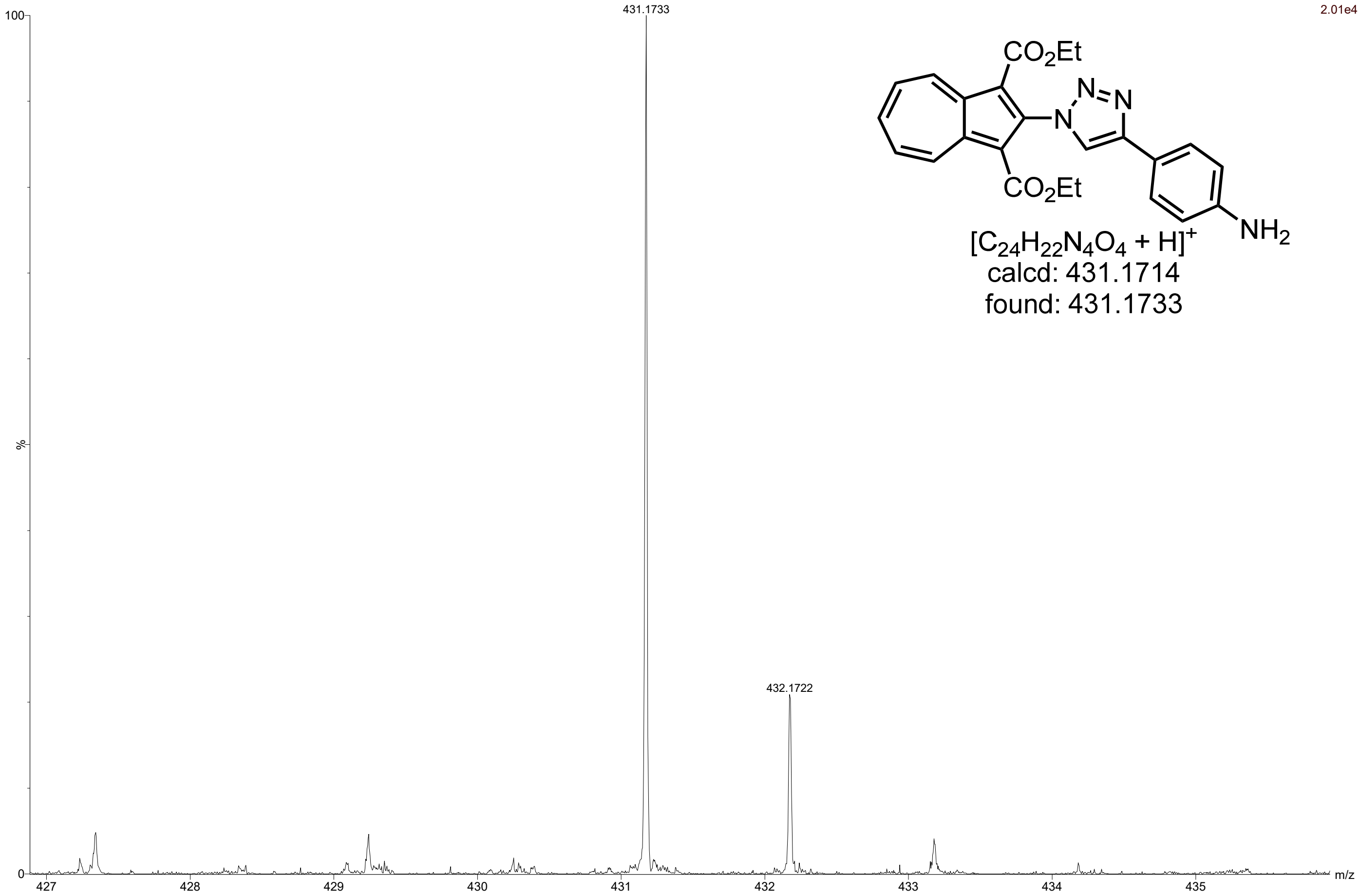

Figure S8. HRMS (ESI-TOF, positive) of **5b**.

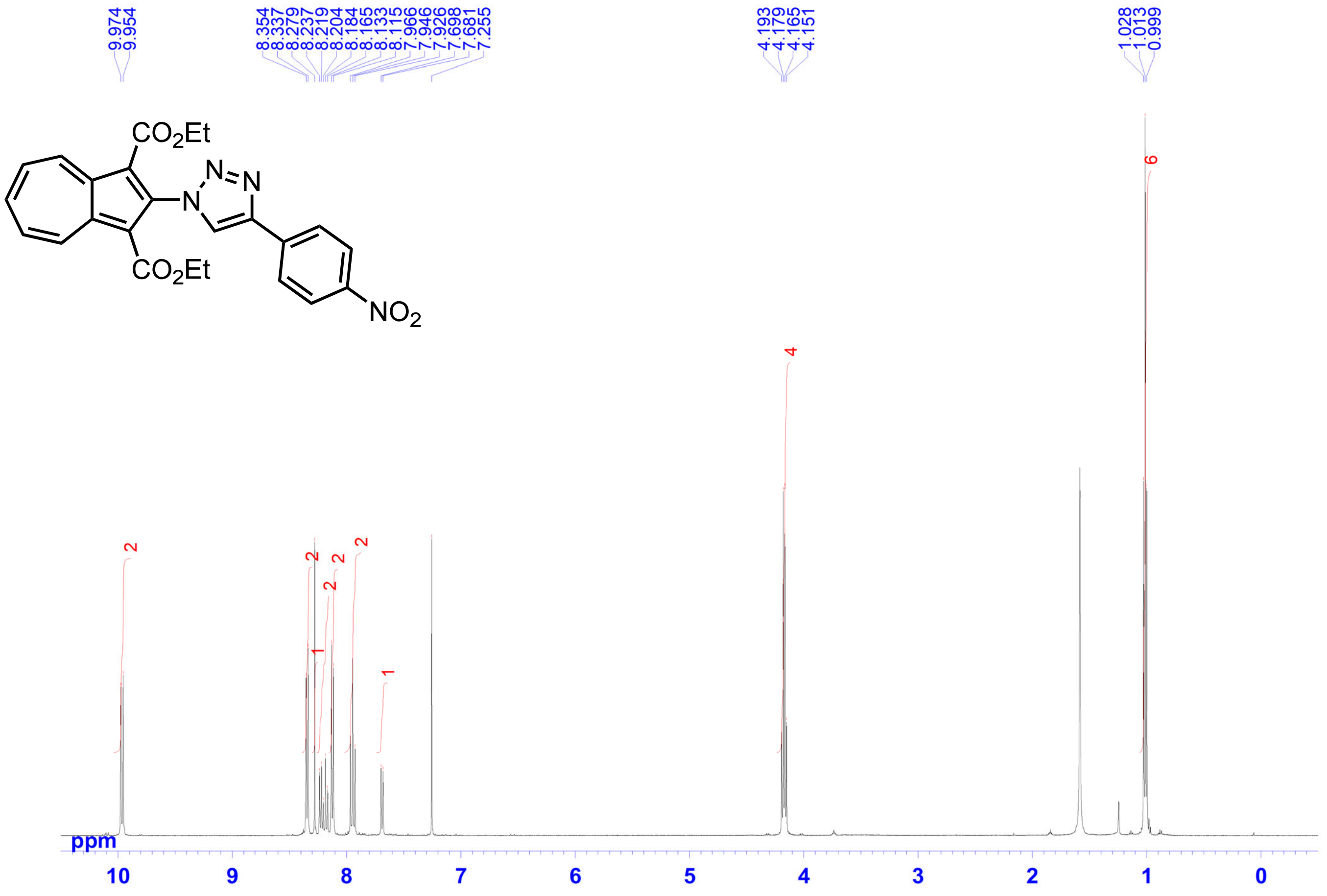

Figure S9. ^{1}H NMR spectrum of **5c** in $CDCl_3$ (500 MHz).

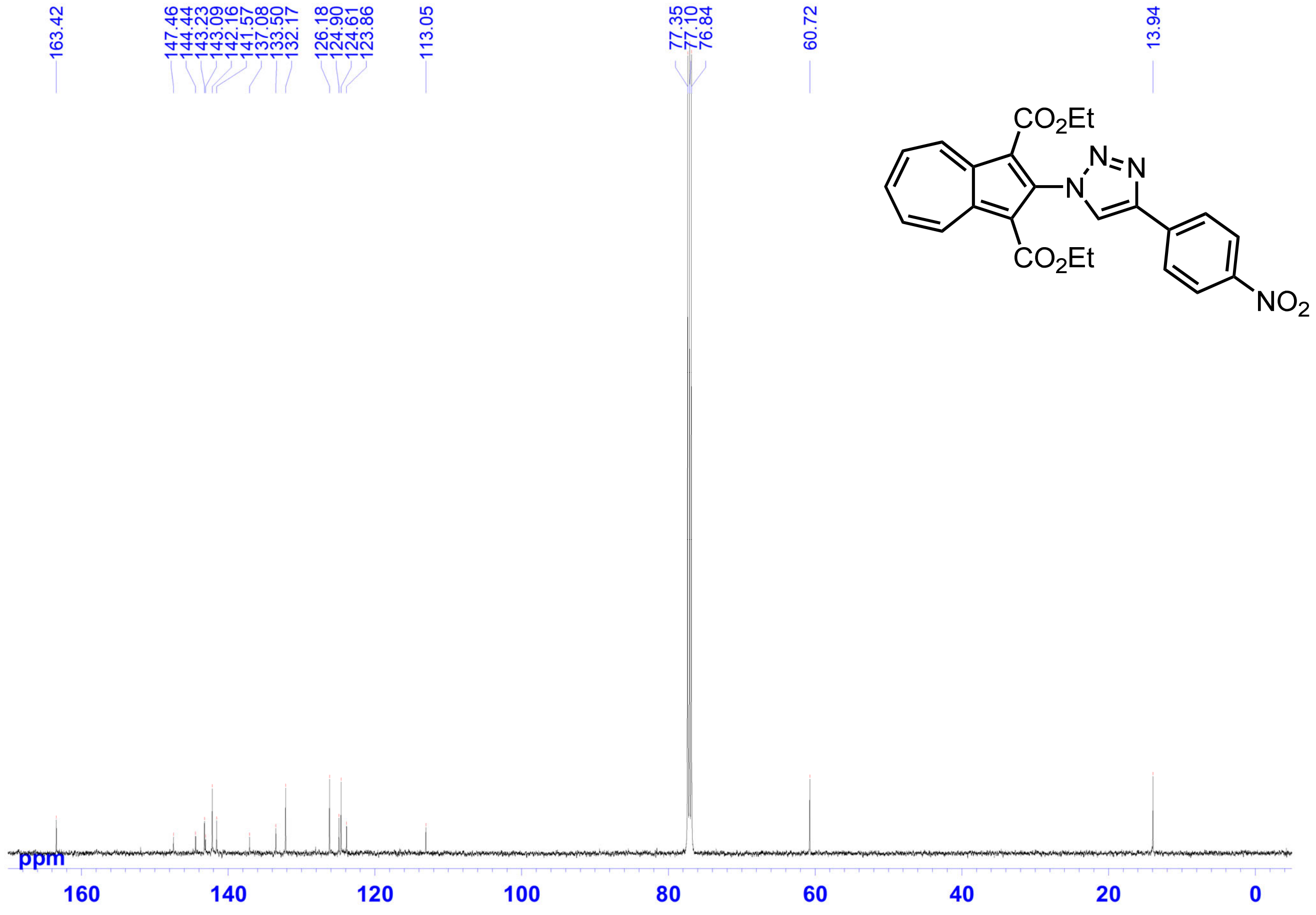

Figure S10. $^{13}C\{^{1}H\}$ NMR spectrum of **5c** in $CDCl_3$ (126 MHz).

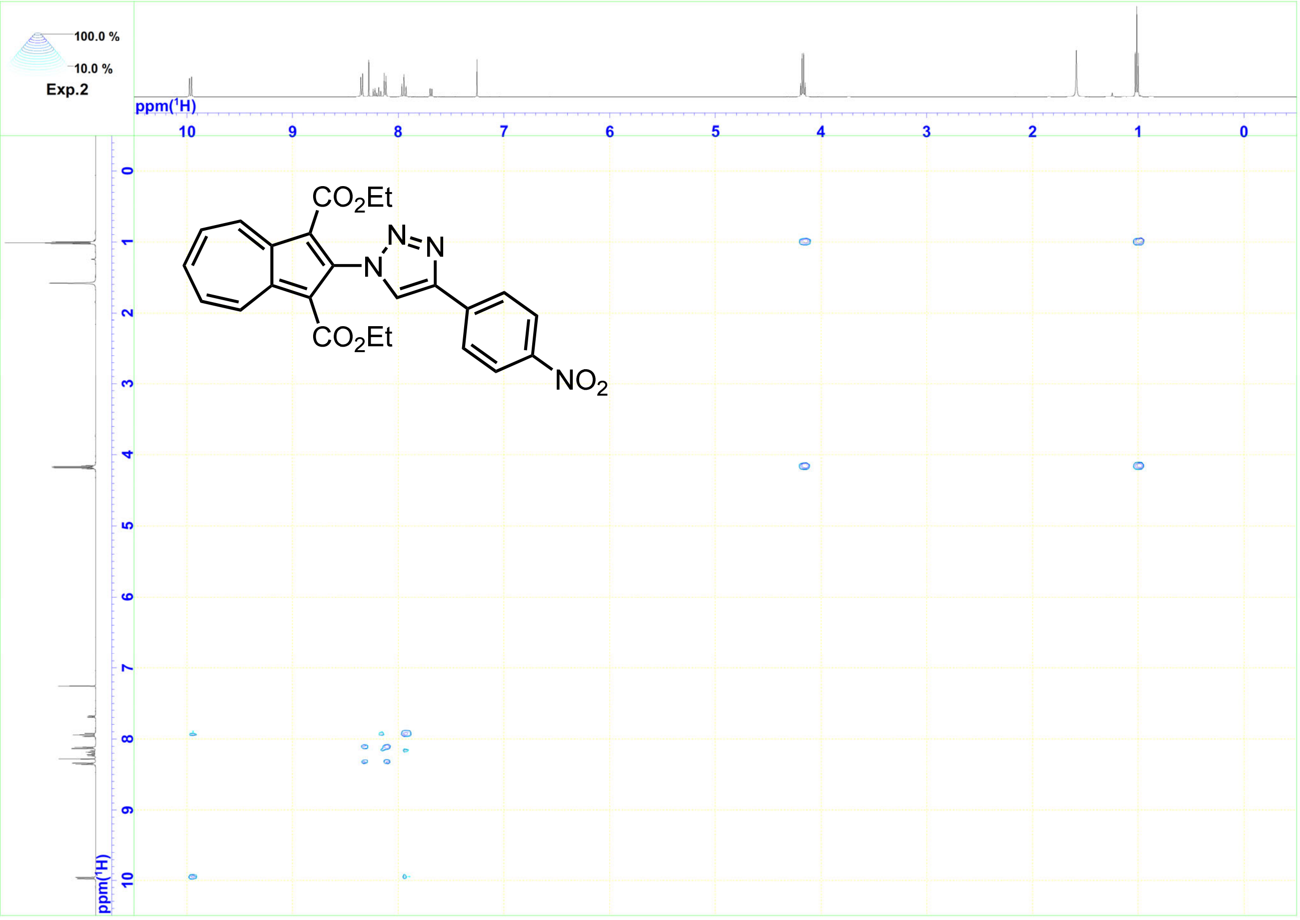

Figure S11. COSY spectrum of **5c** in $CDCl_3$ (500 MHz).

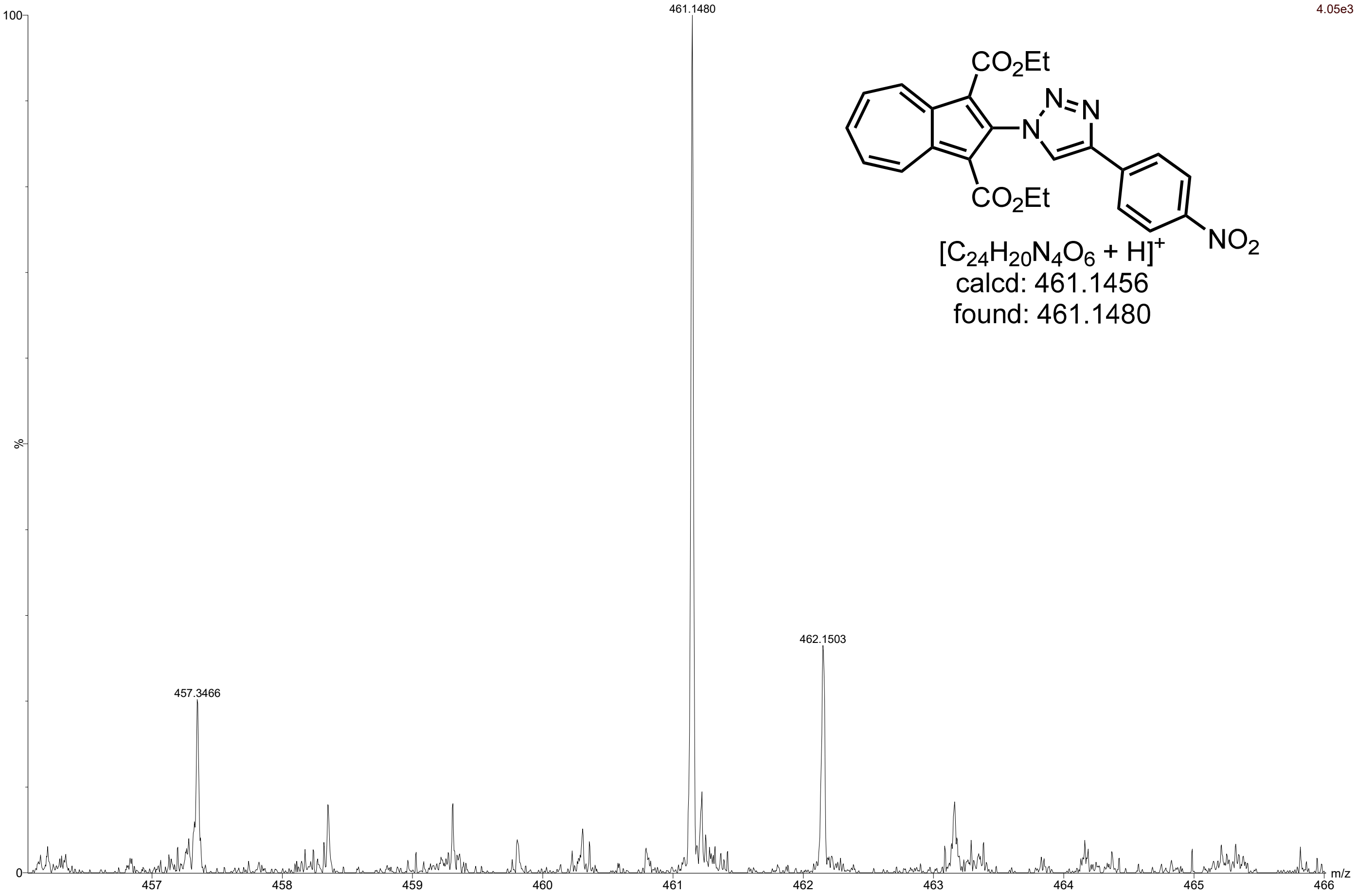

Figure S12. HRMS (ESI-TOF, positive) of **5c**.

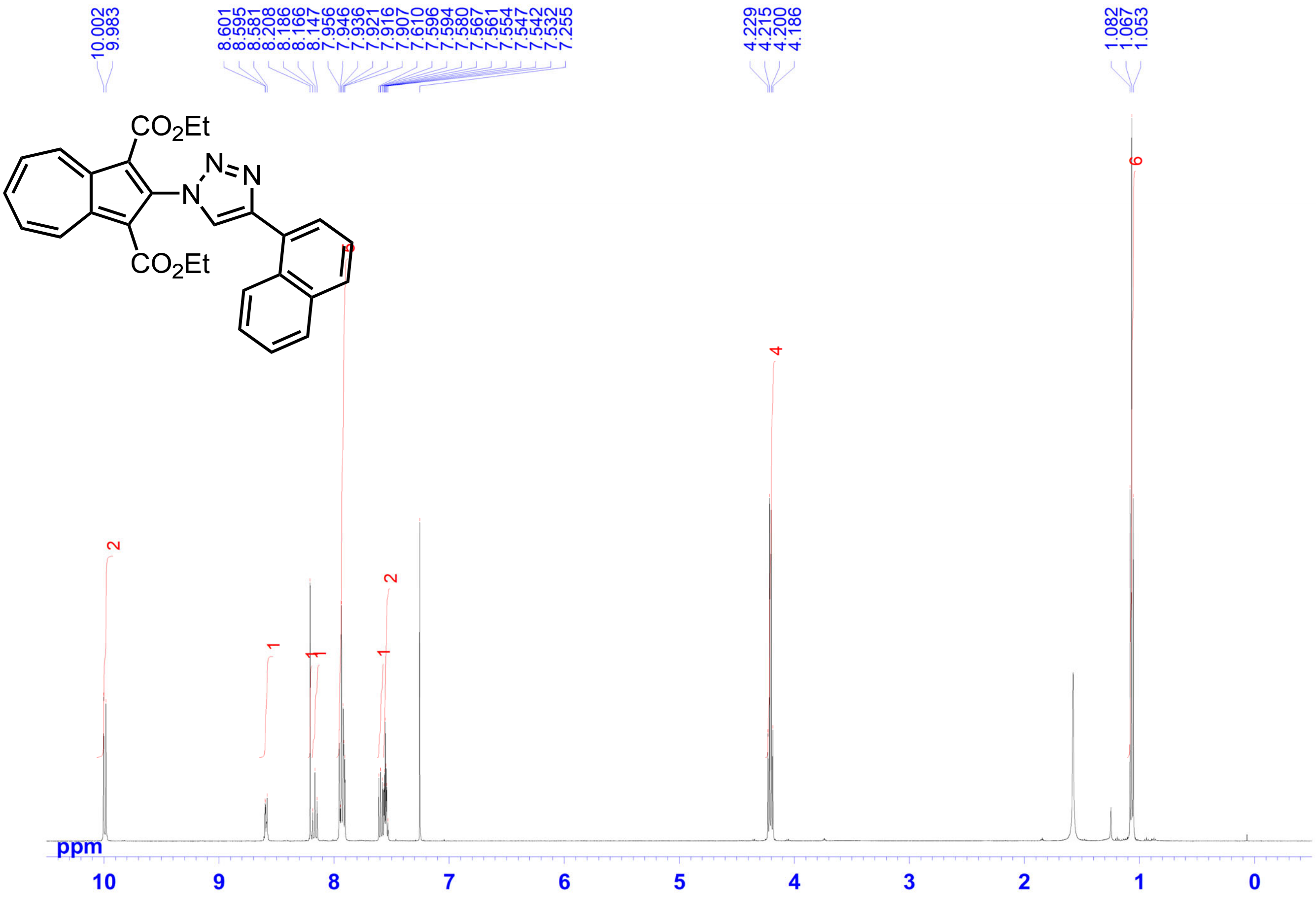

Figure S13. ^{1}H NMR spectrum of **5d** in $CDCl_3$ (500 MHz).

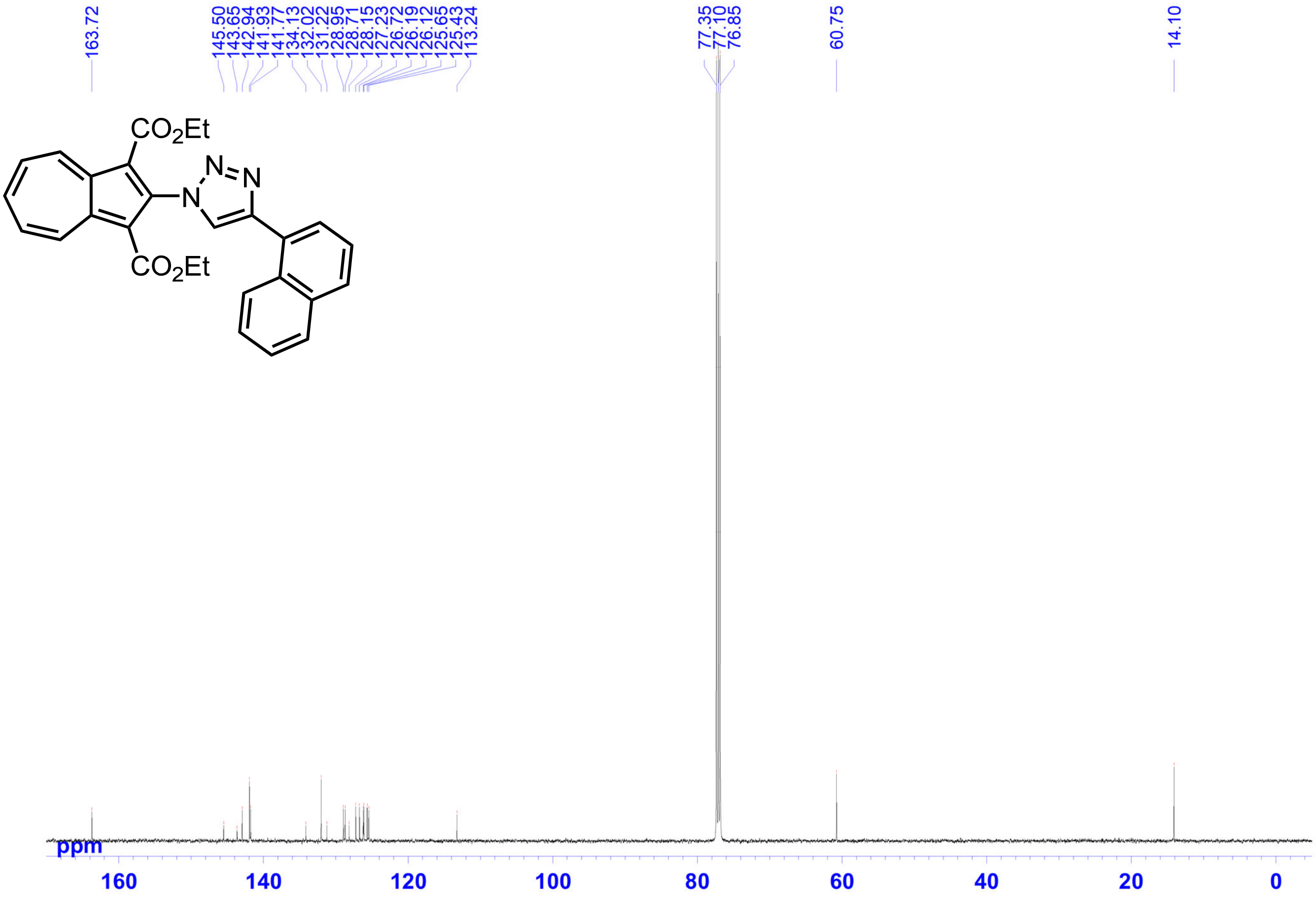

Figure S14. $^{13}C\{^1H\}$ NMR spectrum of **5d** in $CDCl_3$ (126 MHz).

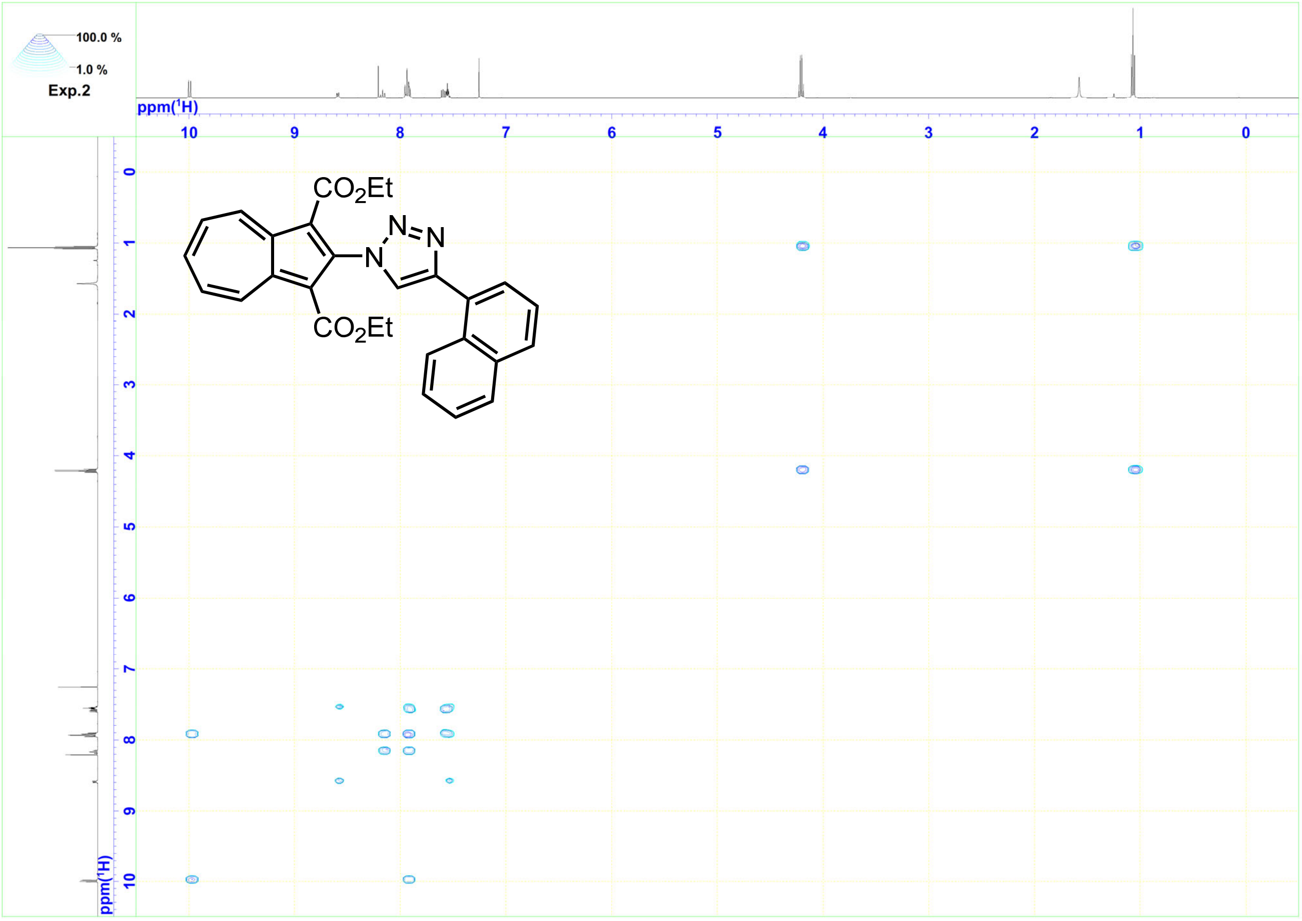

Figure S15. COSY spectrum of **5d** in $CDCl_3$ (500 MHz).

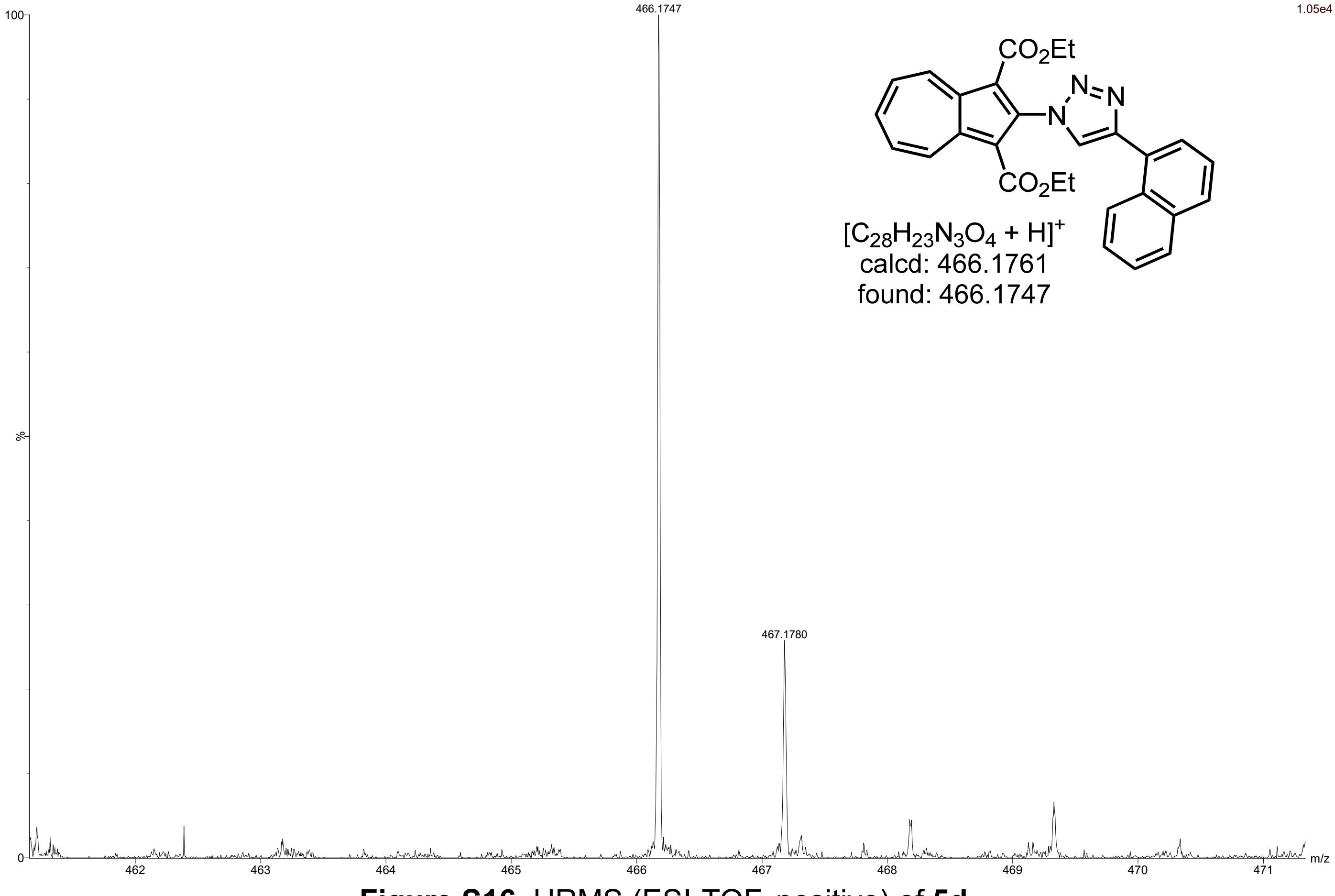

Figure S16. HRMS (ESI-TOF, positive) of **5d**.

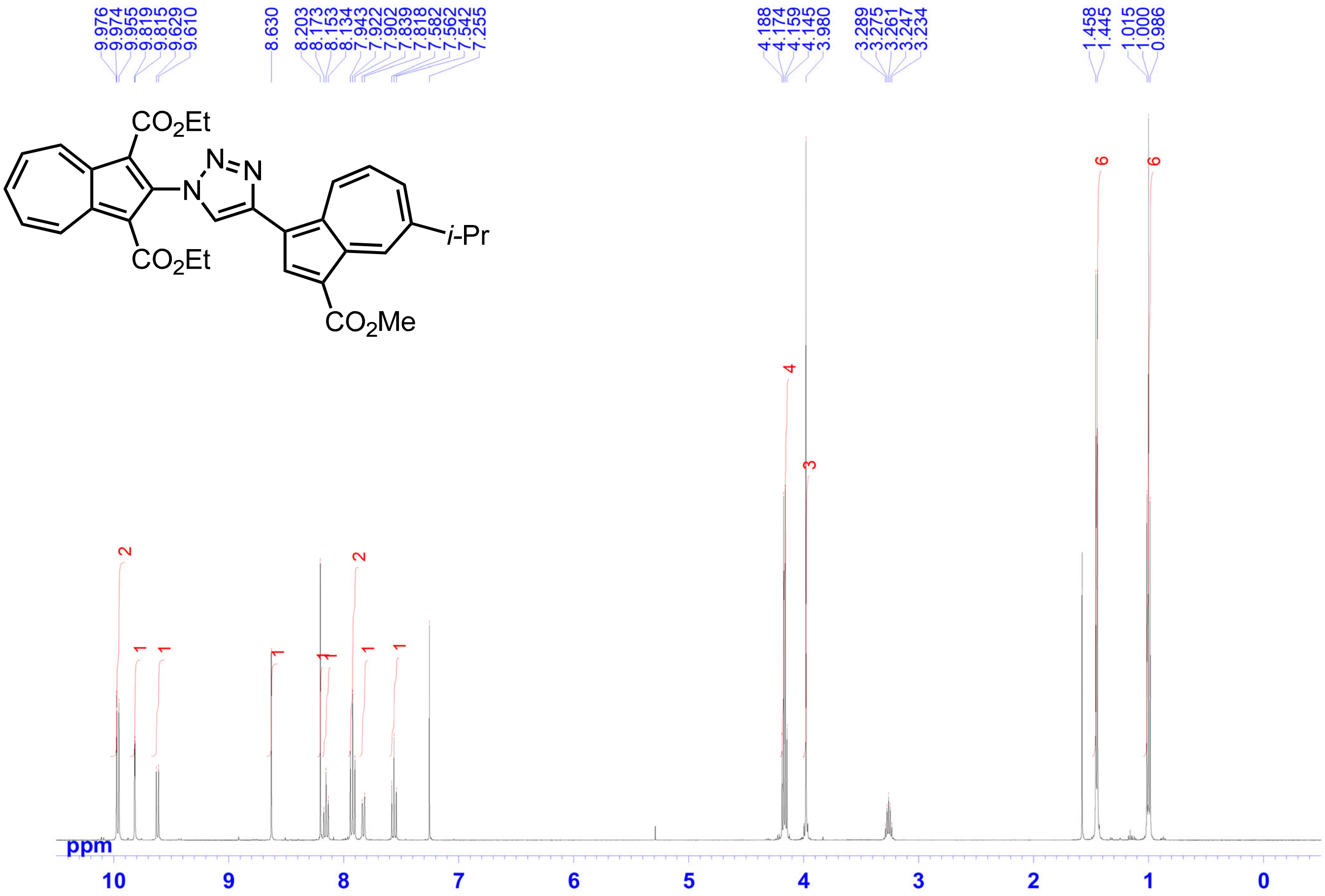

Figure S17. ^{1}H NMR spectrum of **5e** in $CDCl_3$ (500 MHz).

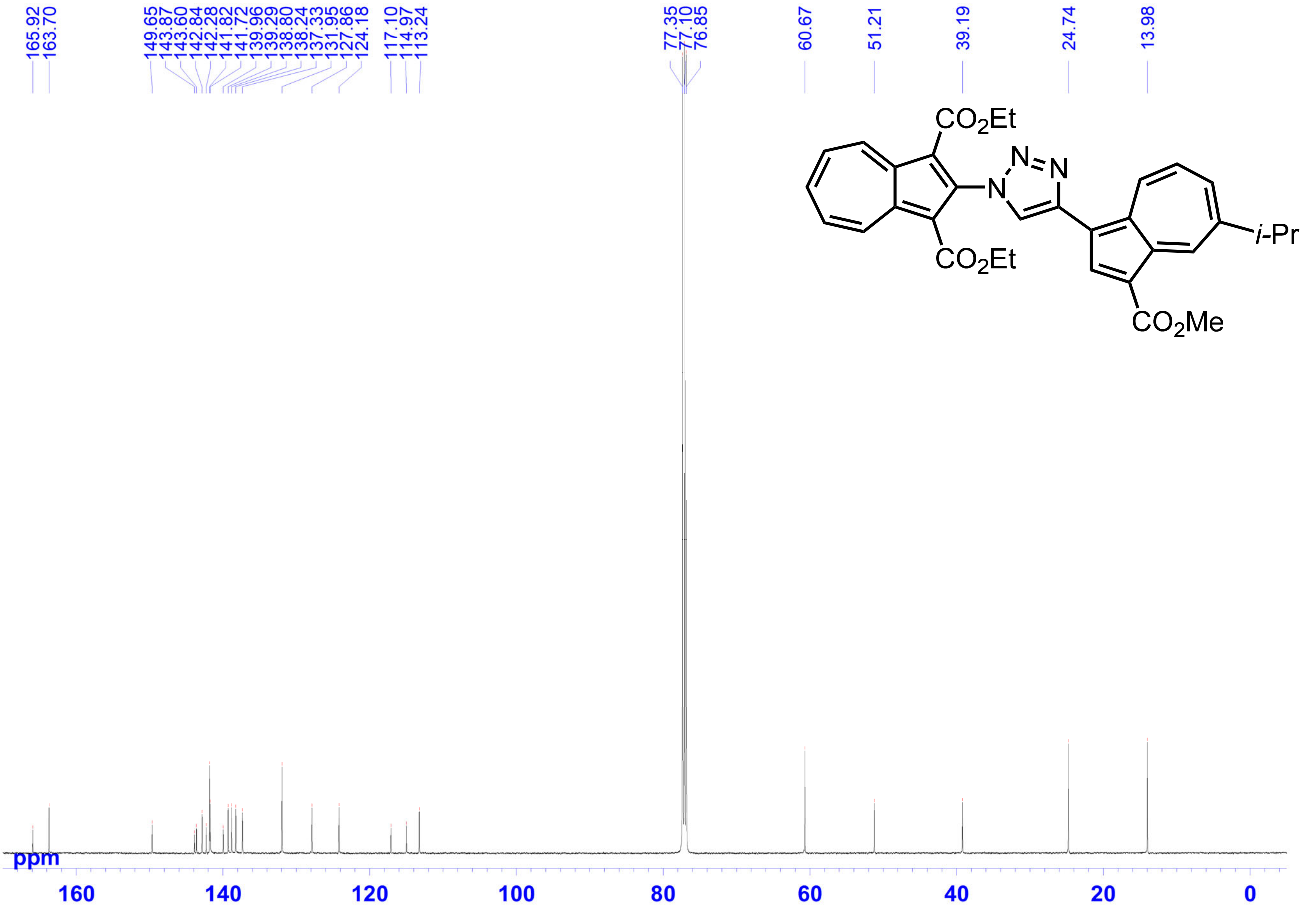

Figure S18. $^{13}C\{^1H\}$ NMR spectrum of **5e** in $CDCl_3$ (126 MHz).

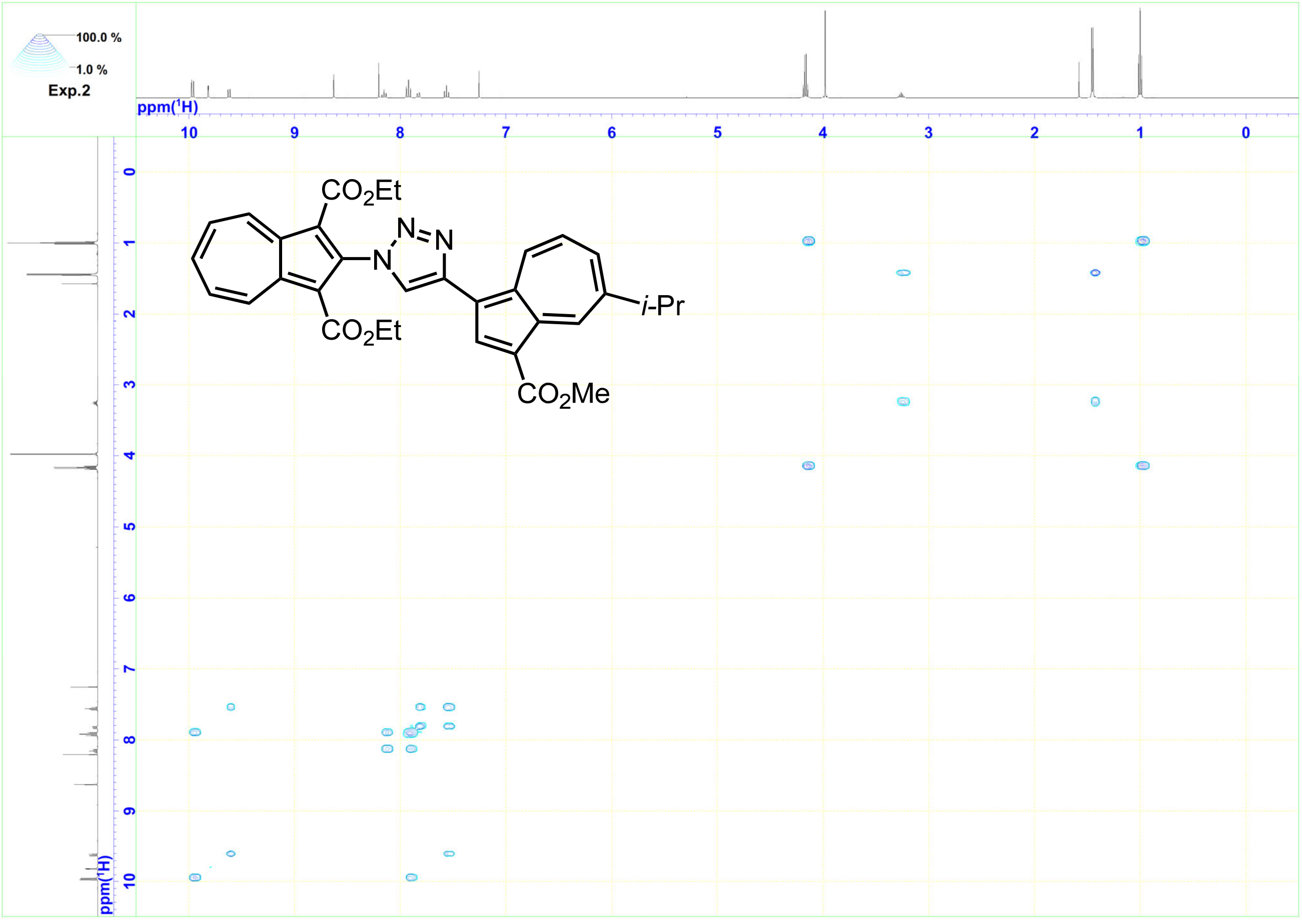

Figure S19. COSY spectrum of **5e** in $CDCl_3$ (500 MHz).

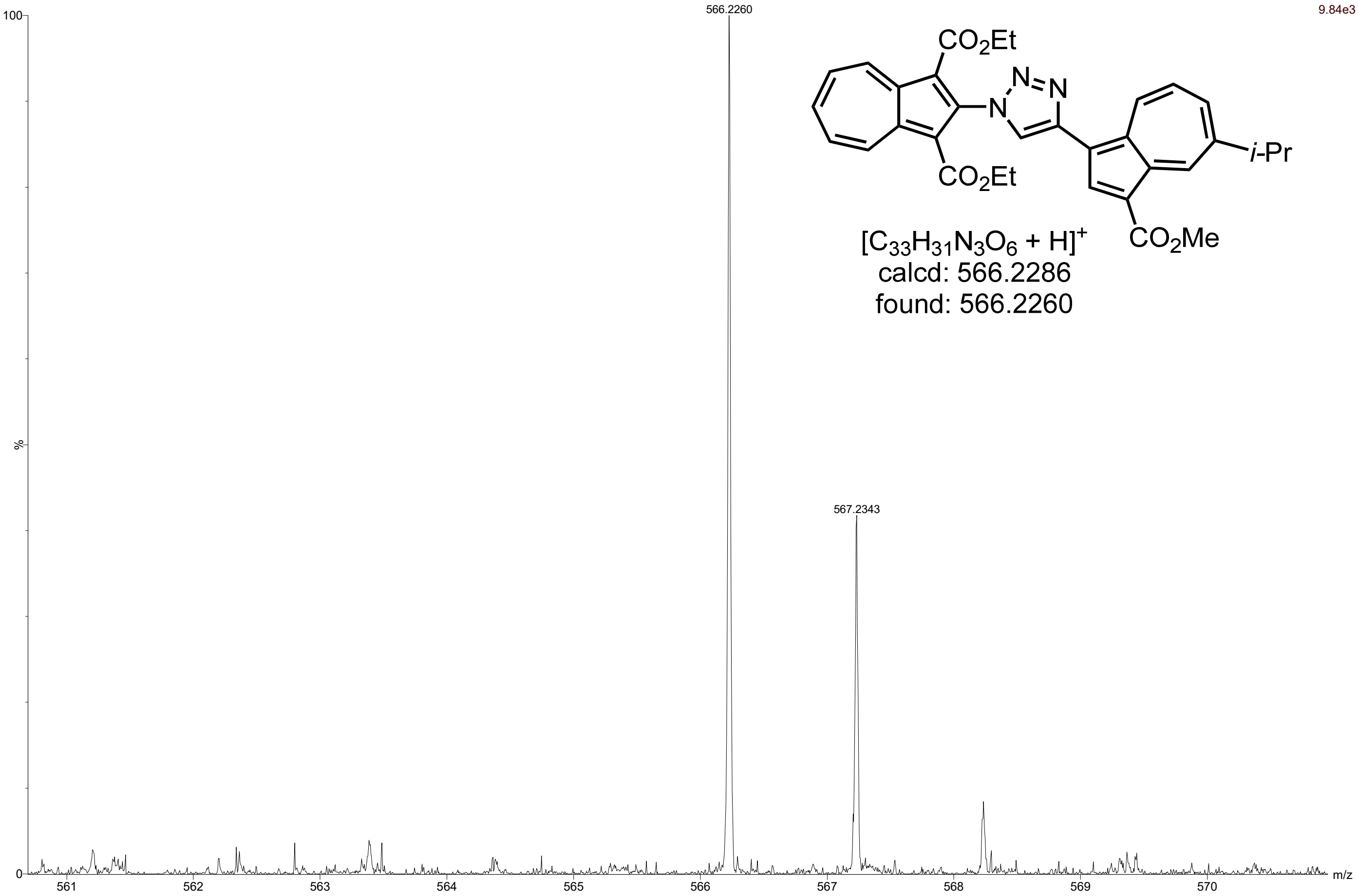

Figure S20. HRMS (ESI-TOF, positive) of **5e**.

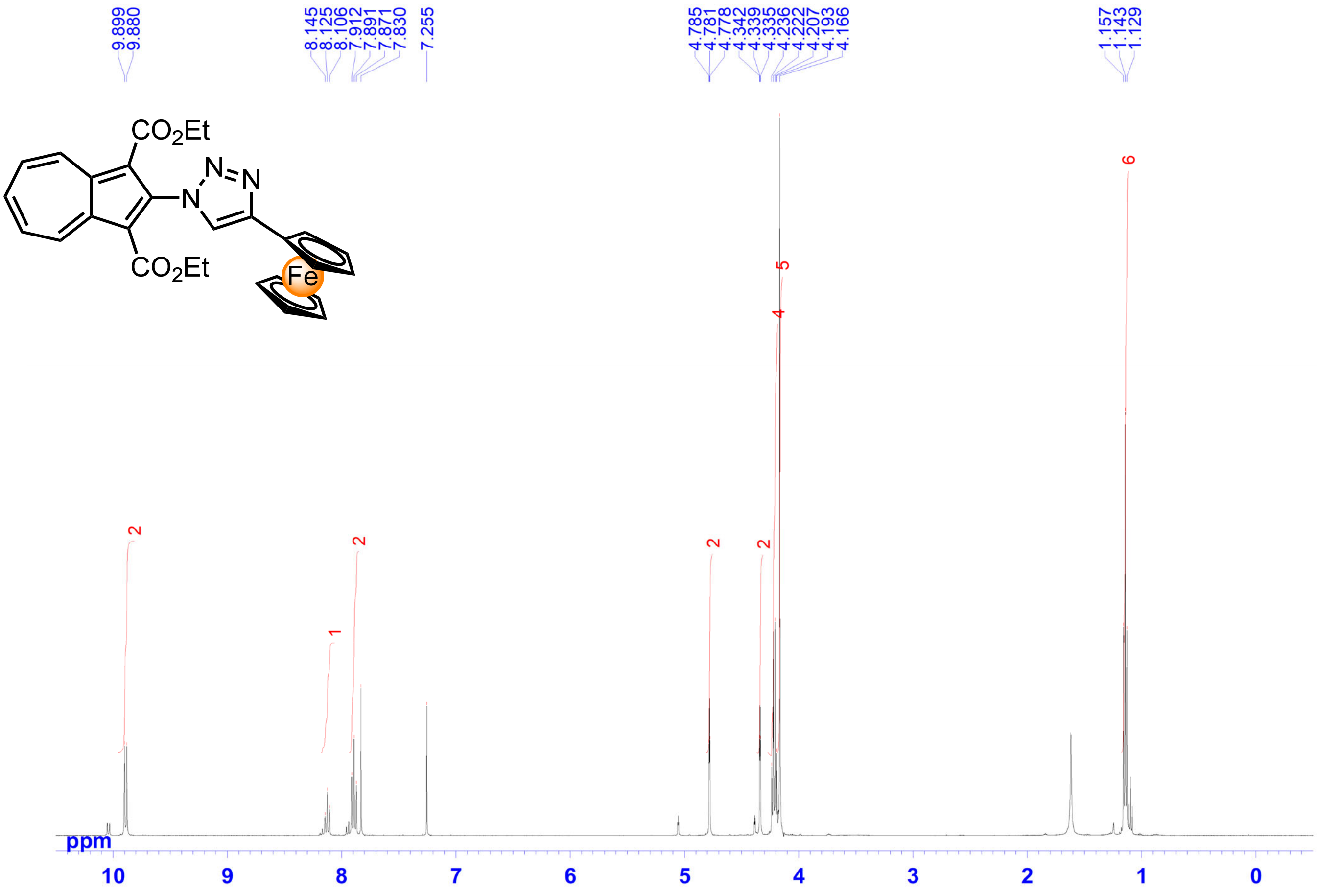

Figure S21. ^{1}H NMR spectrum of **5f** in $CDCl_3$ (500 MHz).

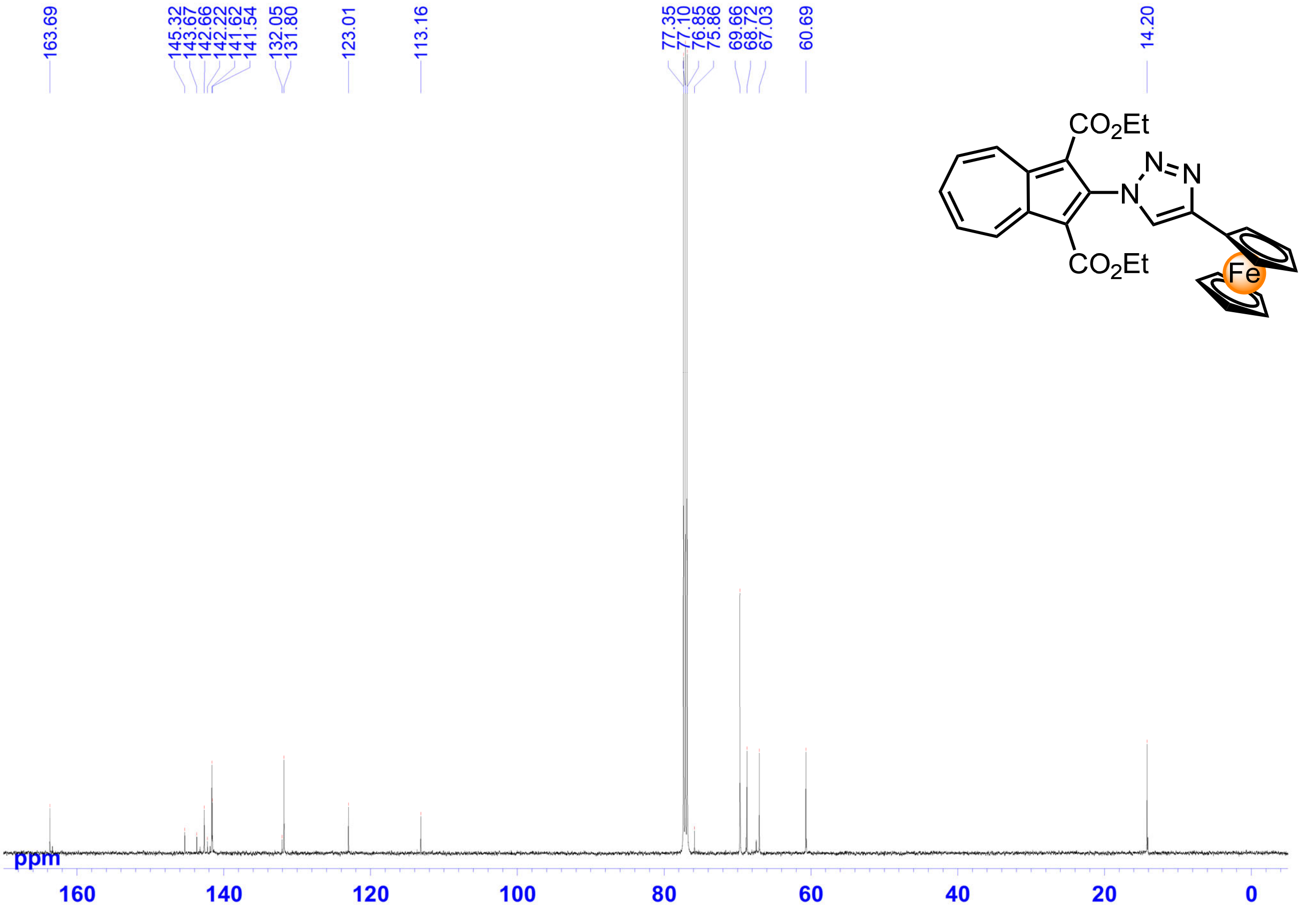

Figure S22. $^{13}C\{^1H\}$ NMR spectrum of **5f** in $CDCl_3$ (126 MHz).

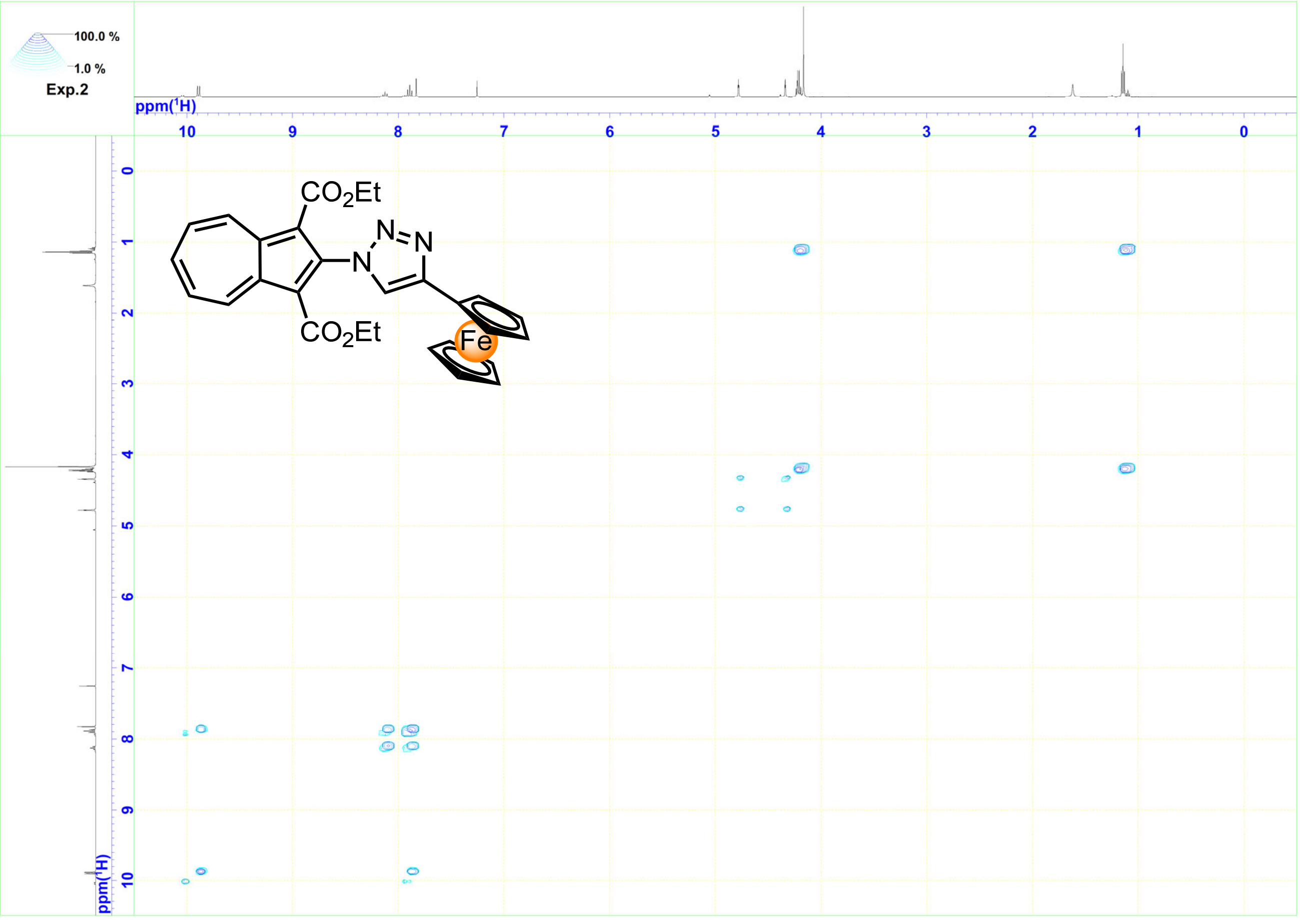

Figure S23. COSY spectrum of **5f** in $CDCl_3$ (500 MHz).

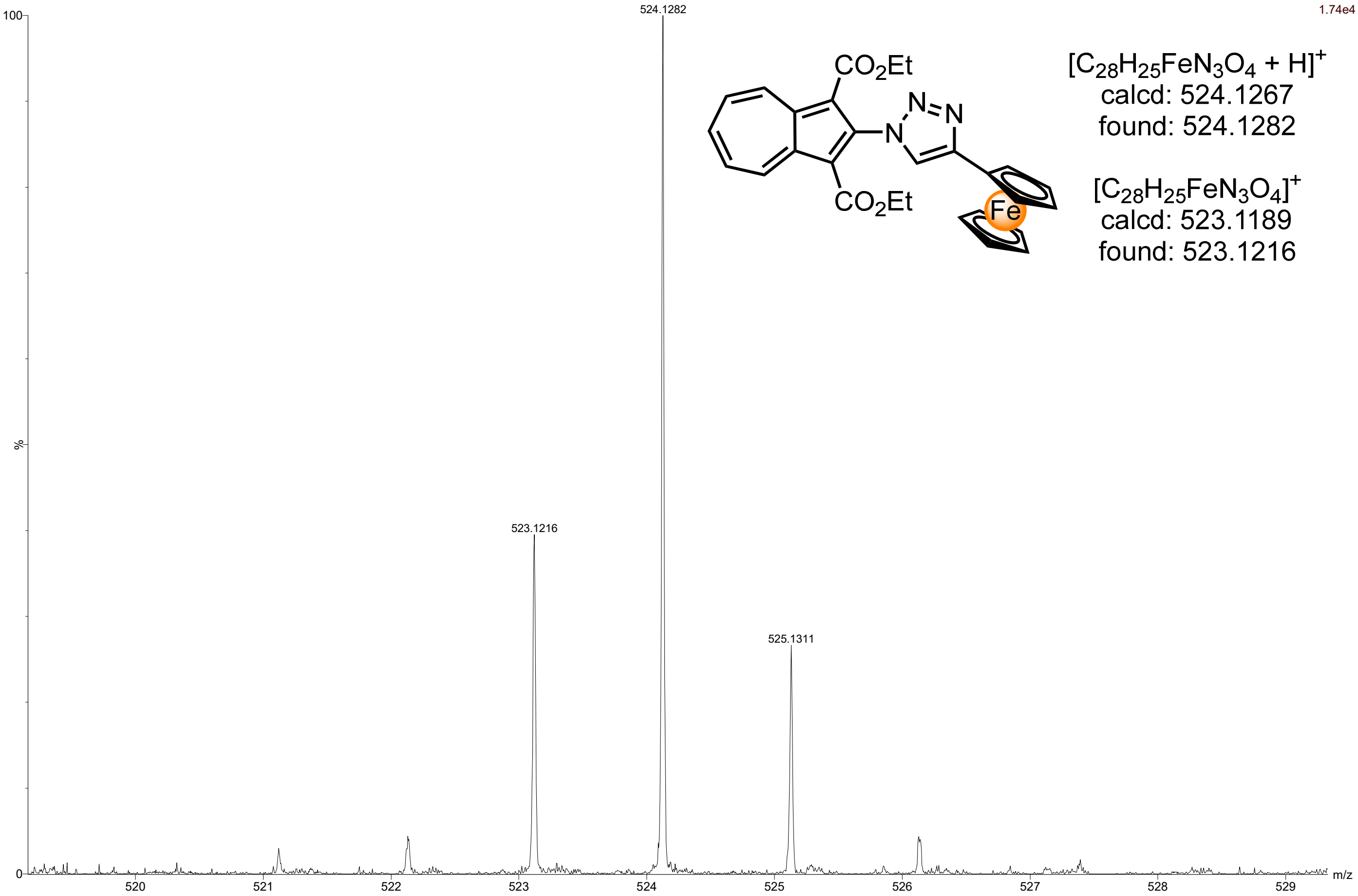

Figure S24. HRMS (ESI-TOF, positive) of **5f**.

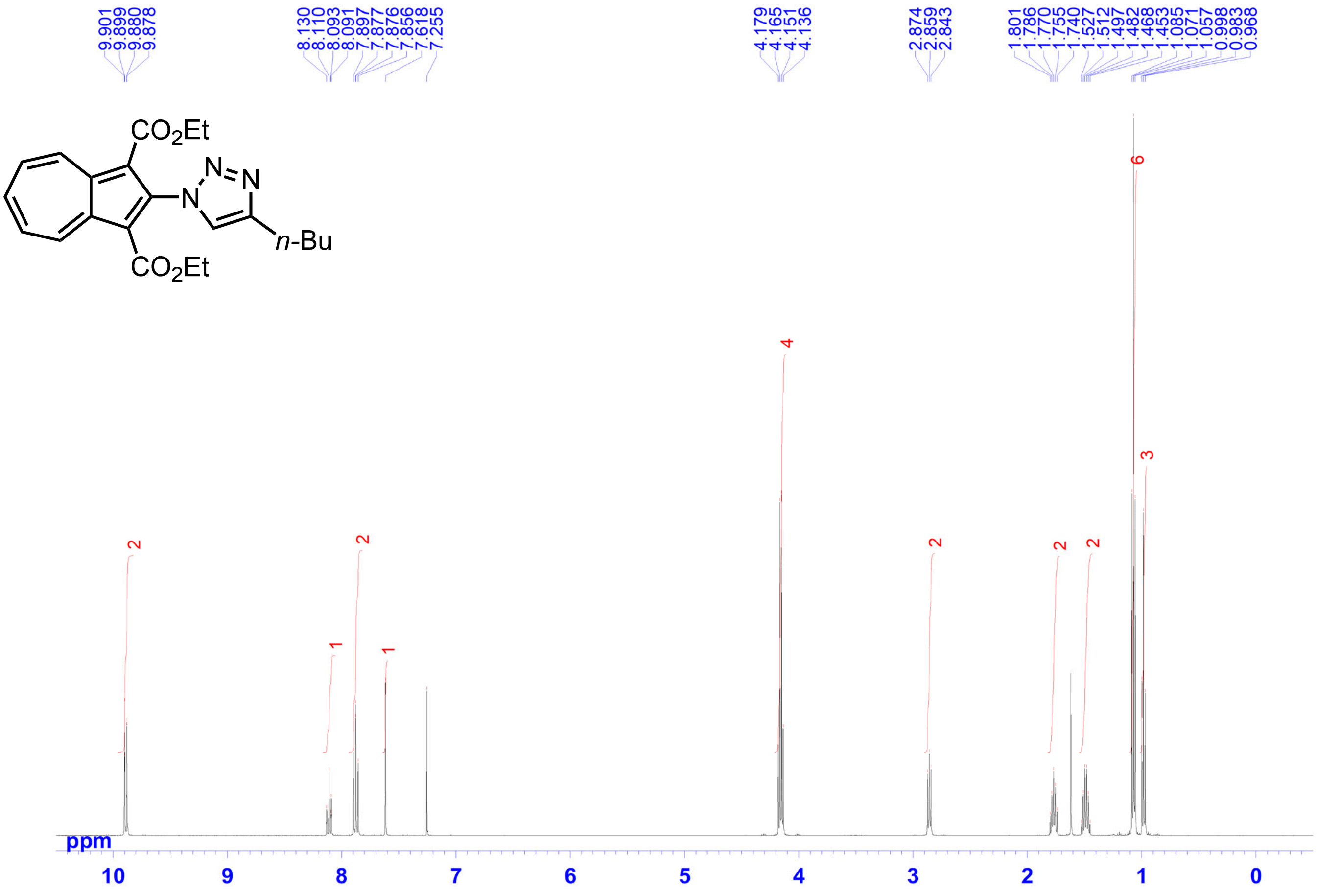

Figure S25. ^{1}H NMR spectrum of **5g** in $CDCl_3$ (500 MHz).

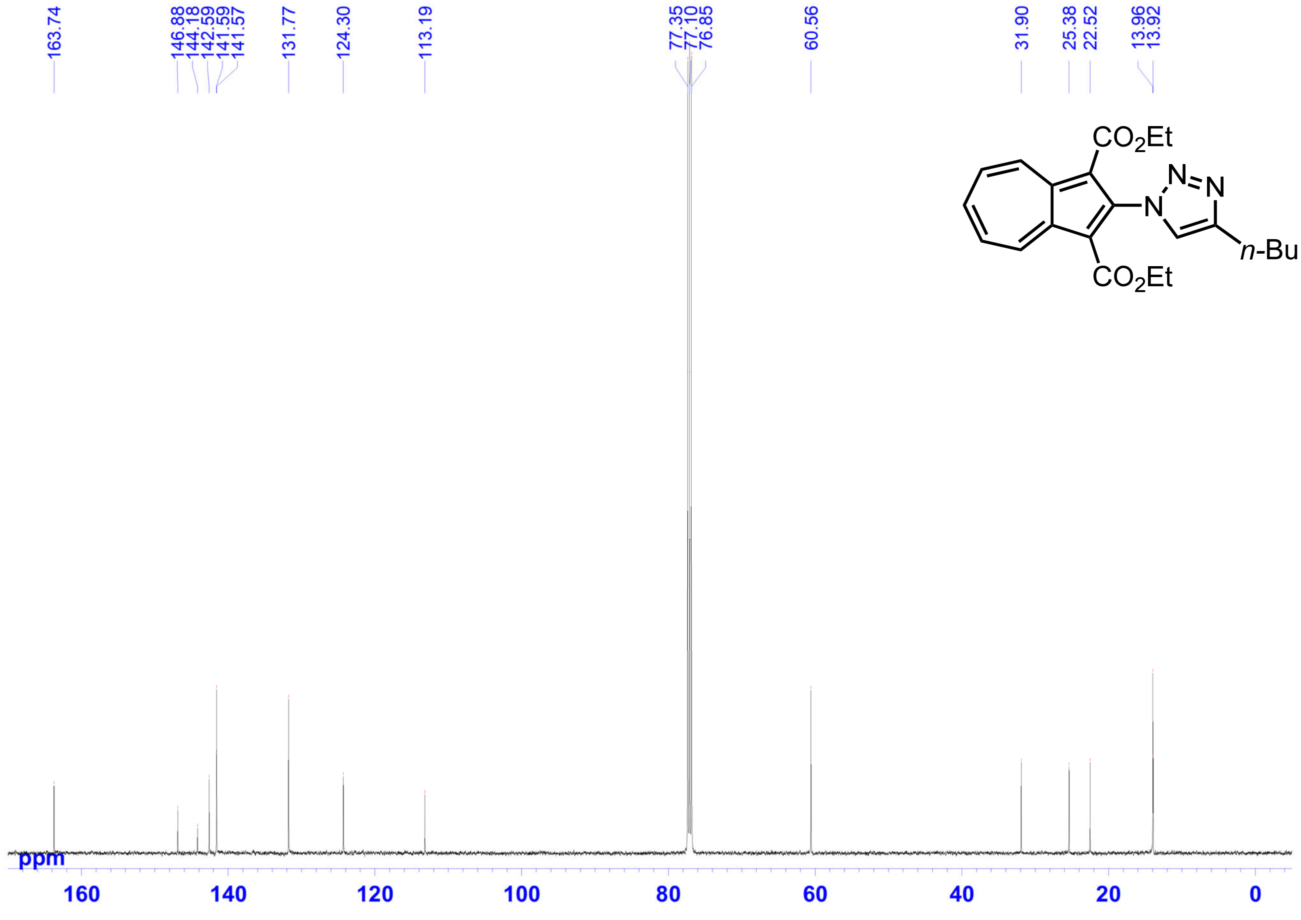

Figure S26. $^{13}C\{^{1}H\}$ NMR spectrum of **5g** in $CDCl_3$ (126 MHz).

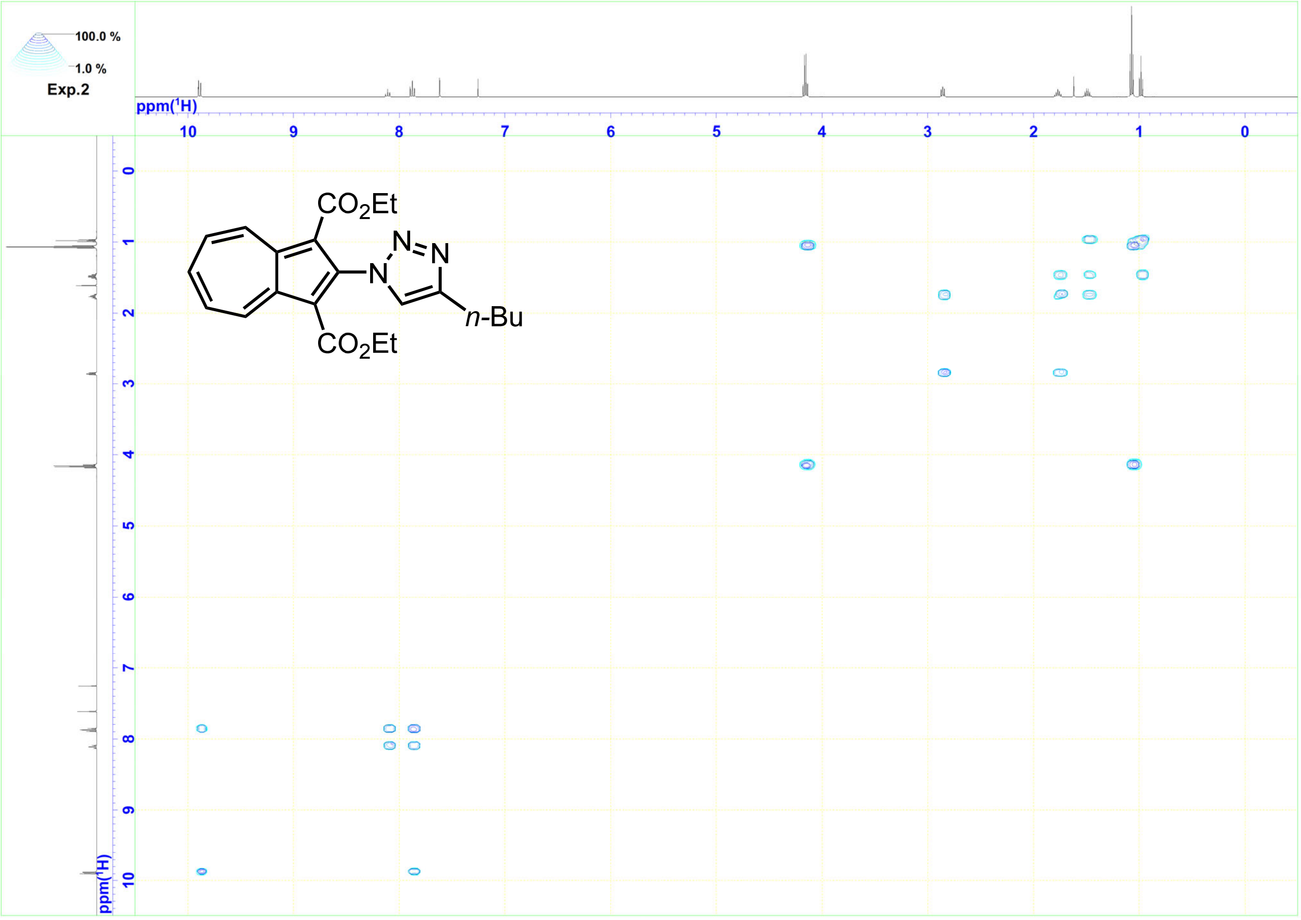

Figure S27. COSY spectrum of **5g** in $CDCl_3$ (500 MHz).

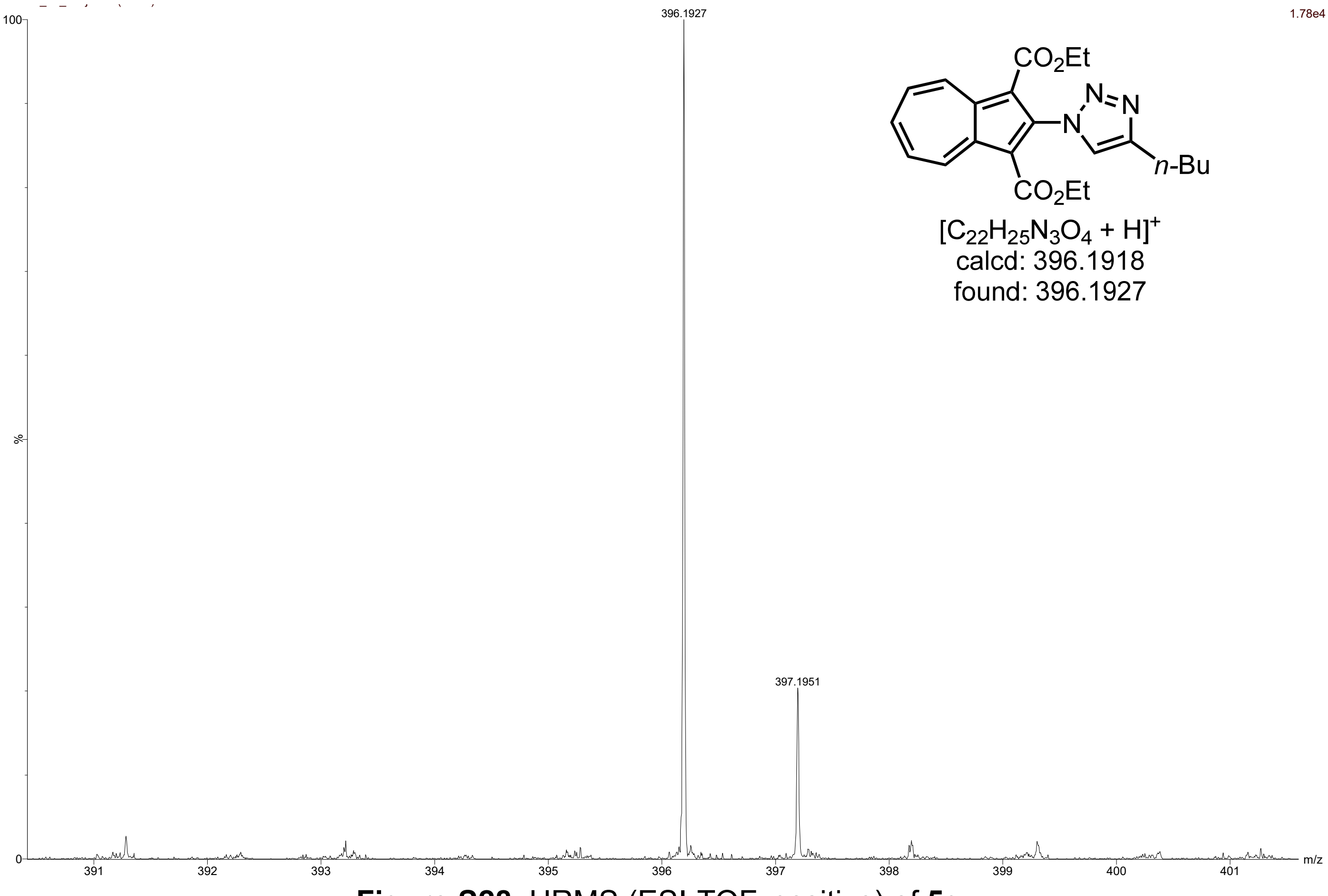

Figure S28. HRMS (ESI-TOF, positive) of **5g**.

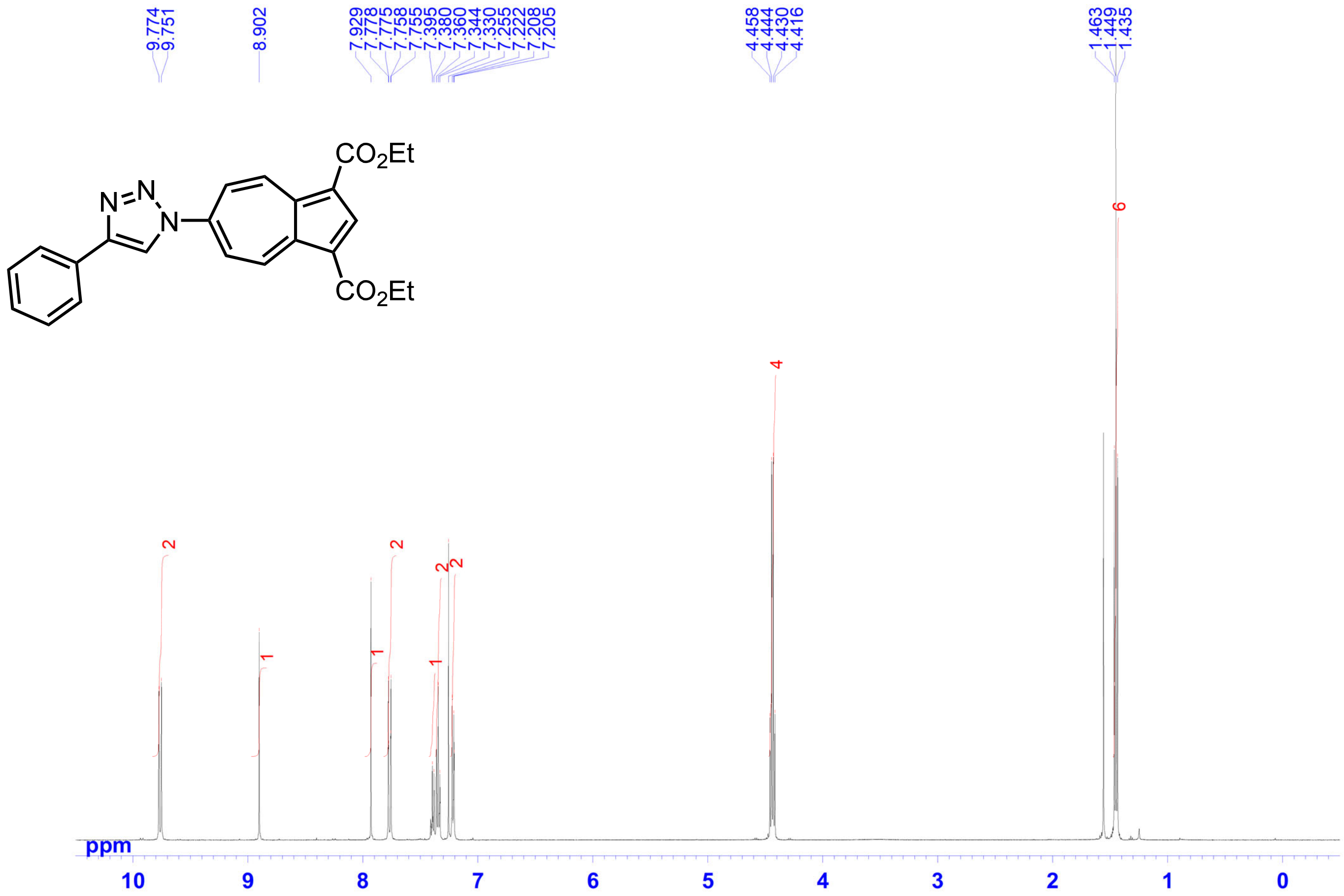

Figure S29. ^{1}H NMR spectrum of **6a** in $CDCl_3$ (500 MHz).

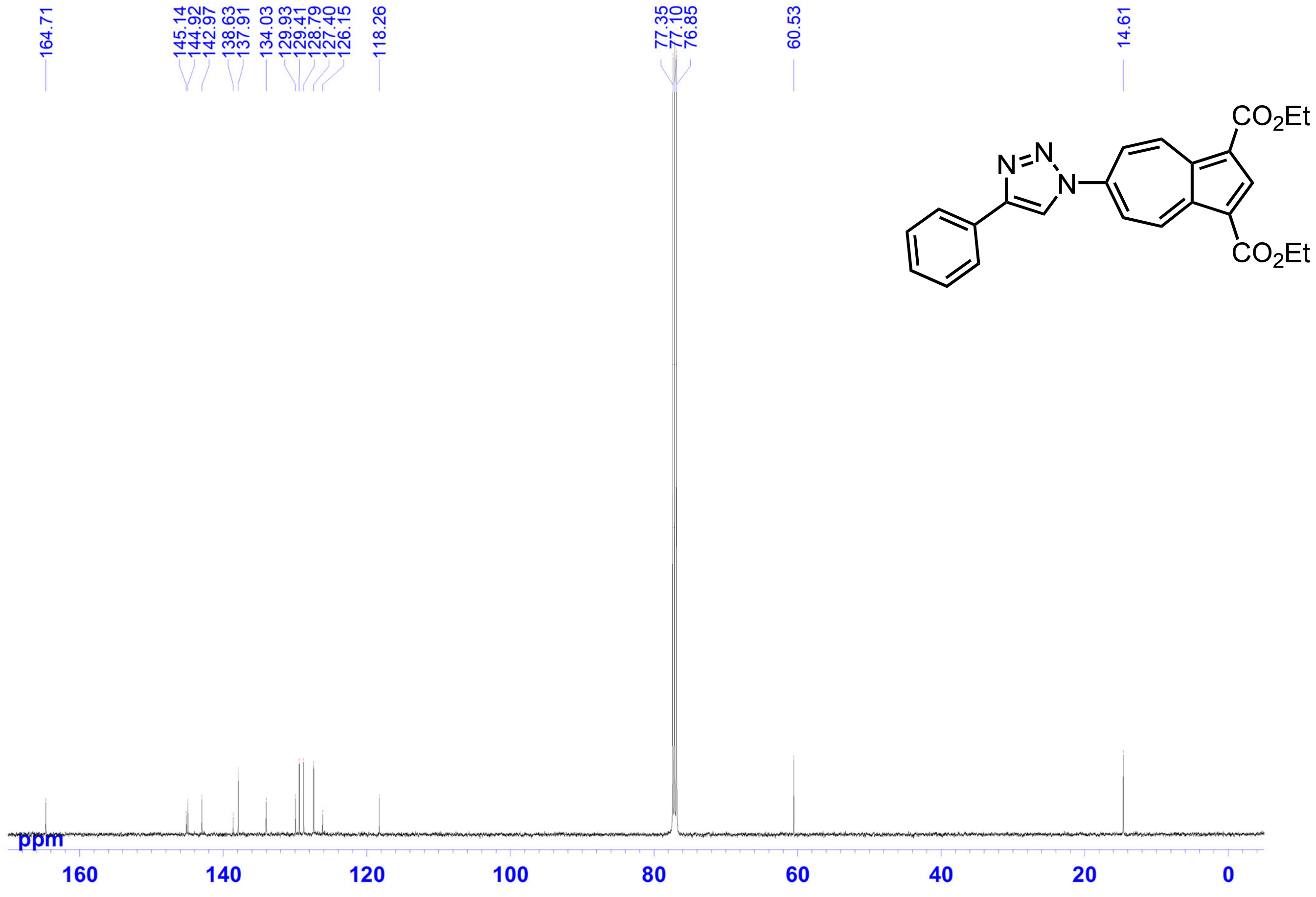

Figure S30. $^{13}C\{^{1}H\}$ NMR spectrum of **6a** in $CDCl_3$ (126 MHz).

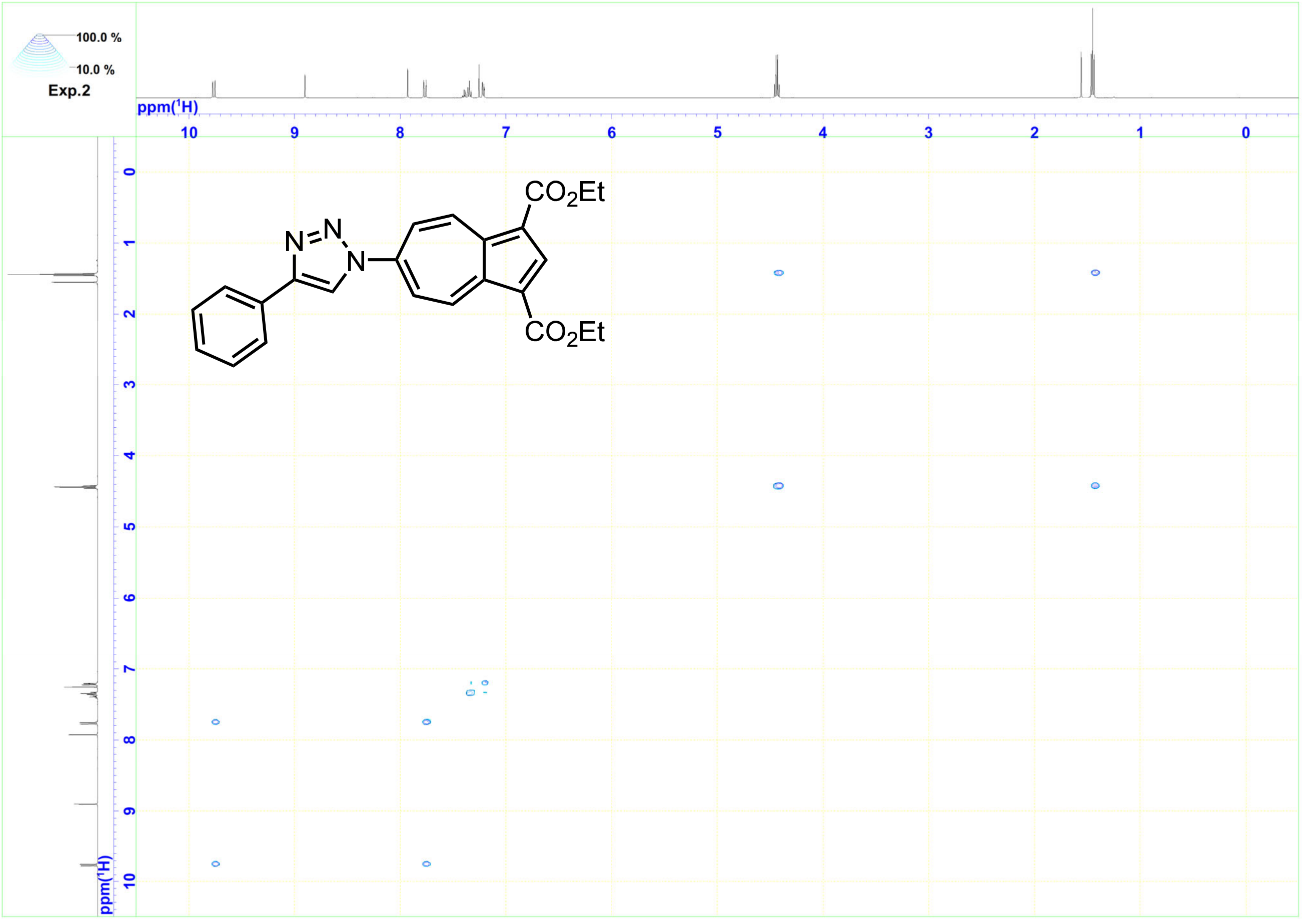

Figure S31. COSY spectrum of **6a** in CDCl3 (500 MHz).

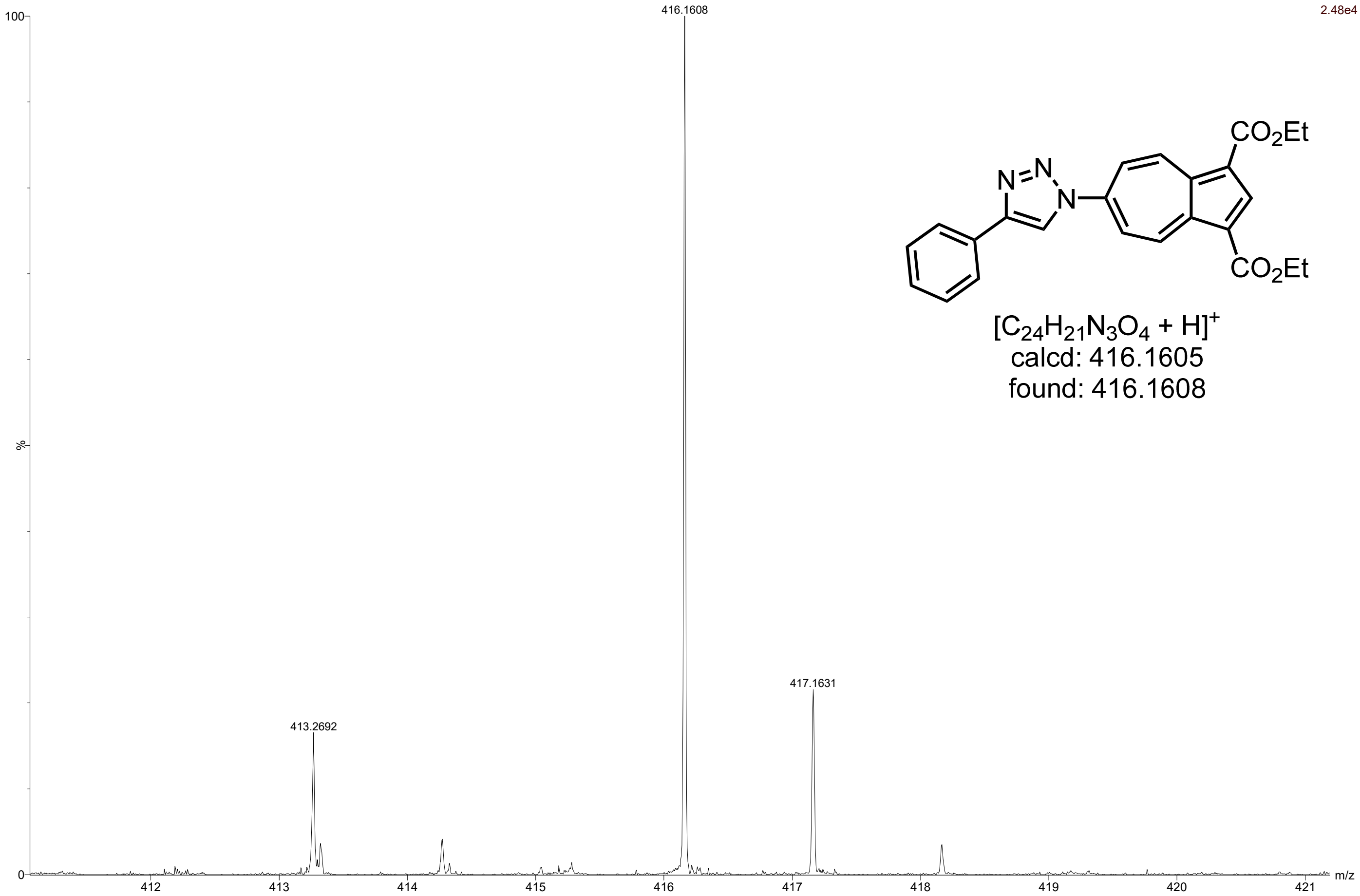

Figure S32. HRMS (ESI-TOF, positive) of **6a**.

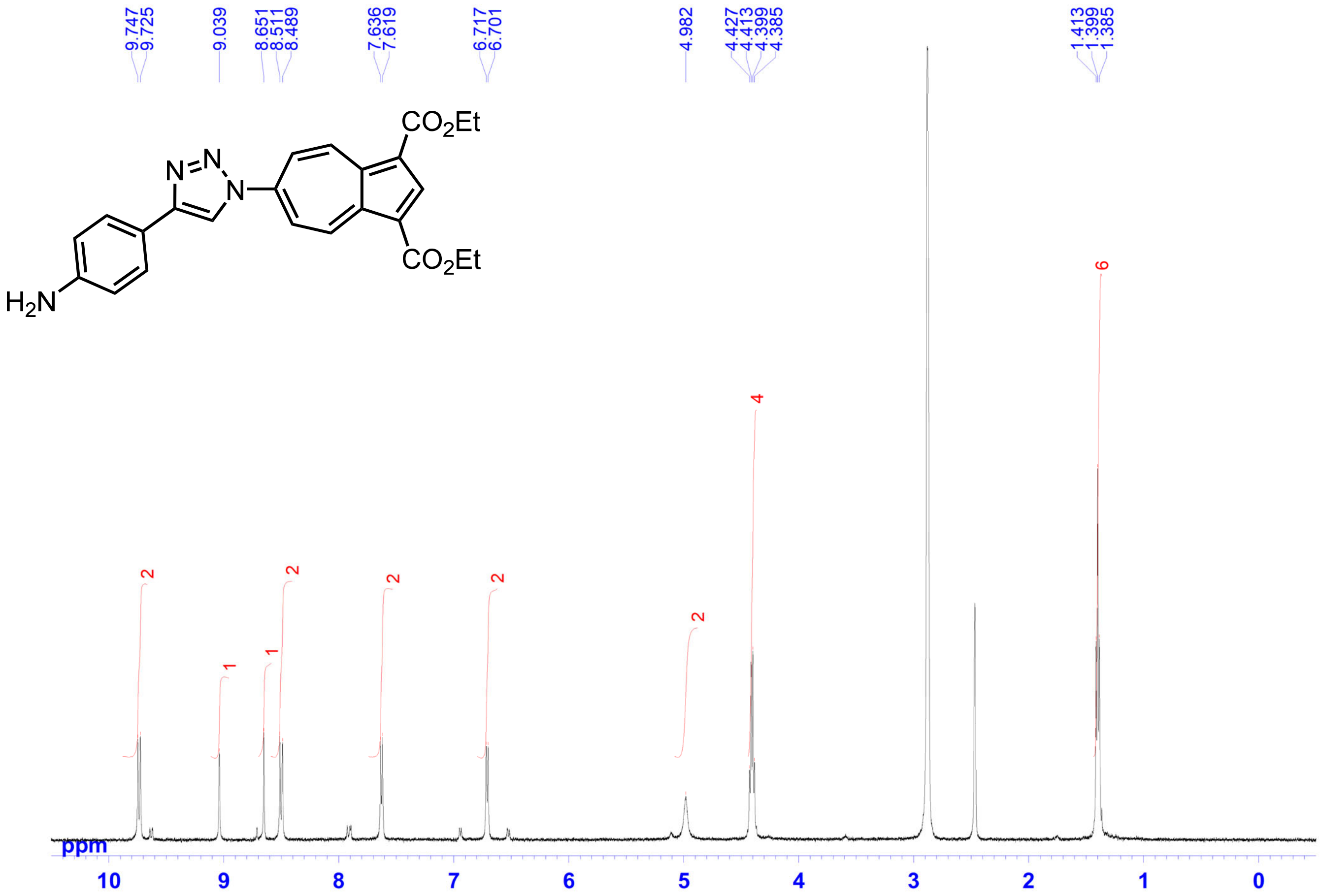

Figure S33. ^{1}H NMR spectrum of **6b** in DMSO-d_6 (500 MHz).

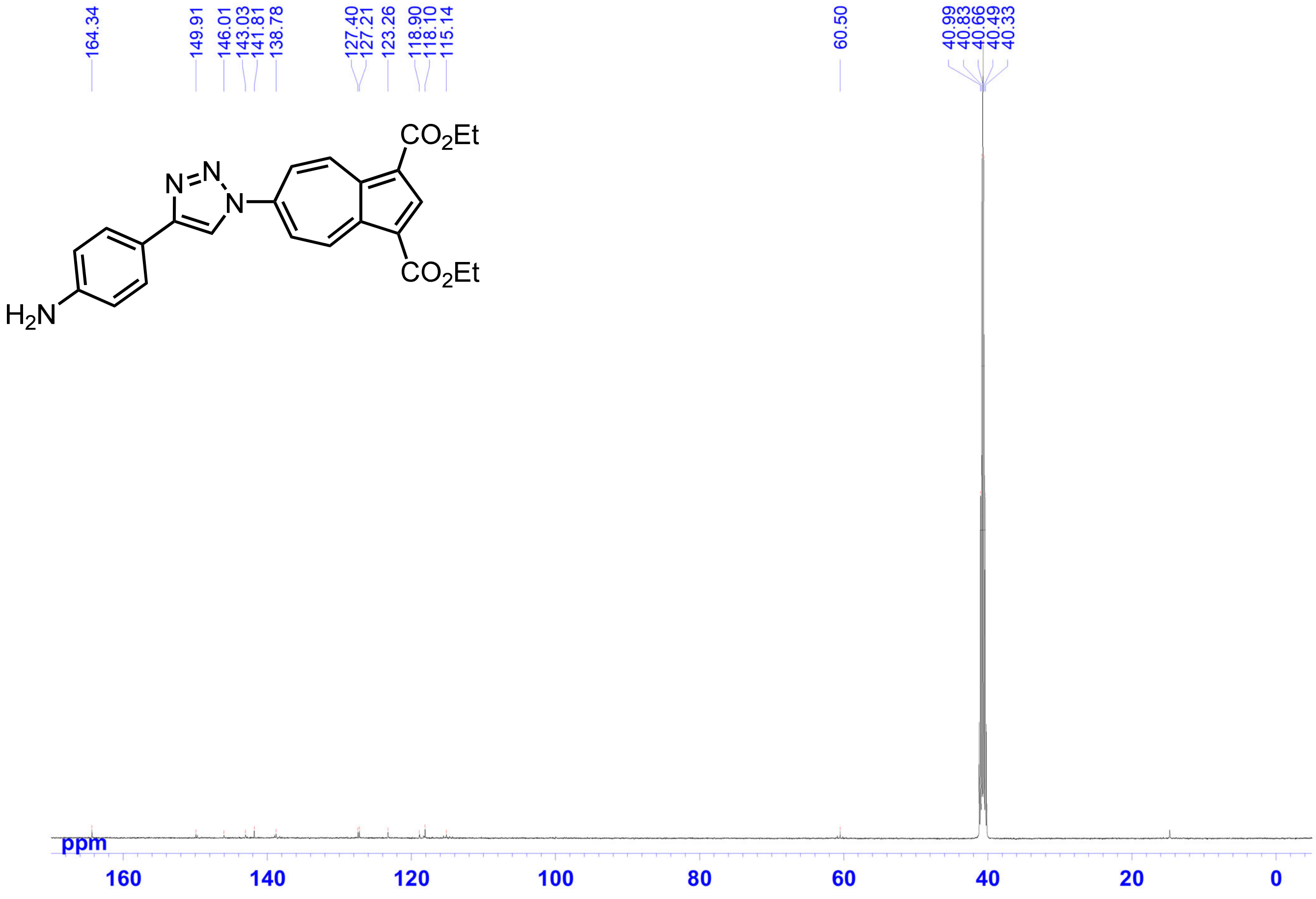

Figure S34. $^{13}C\{^{1}H\}$ NMR spectrum of **6b** in DMSO-d_6 (126 MHz).

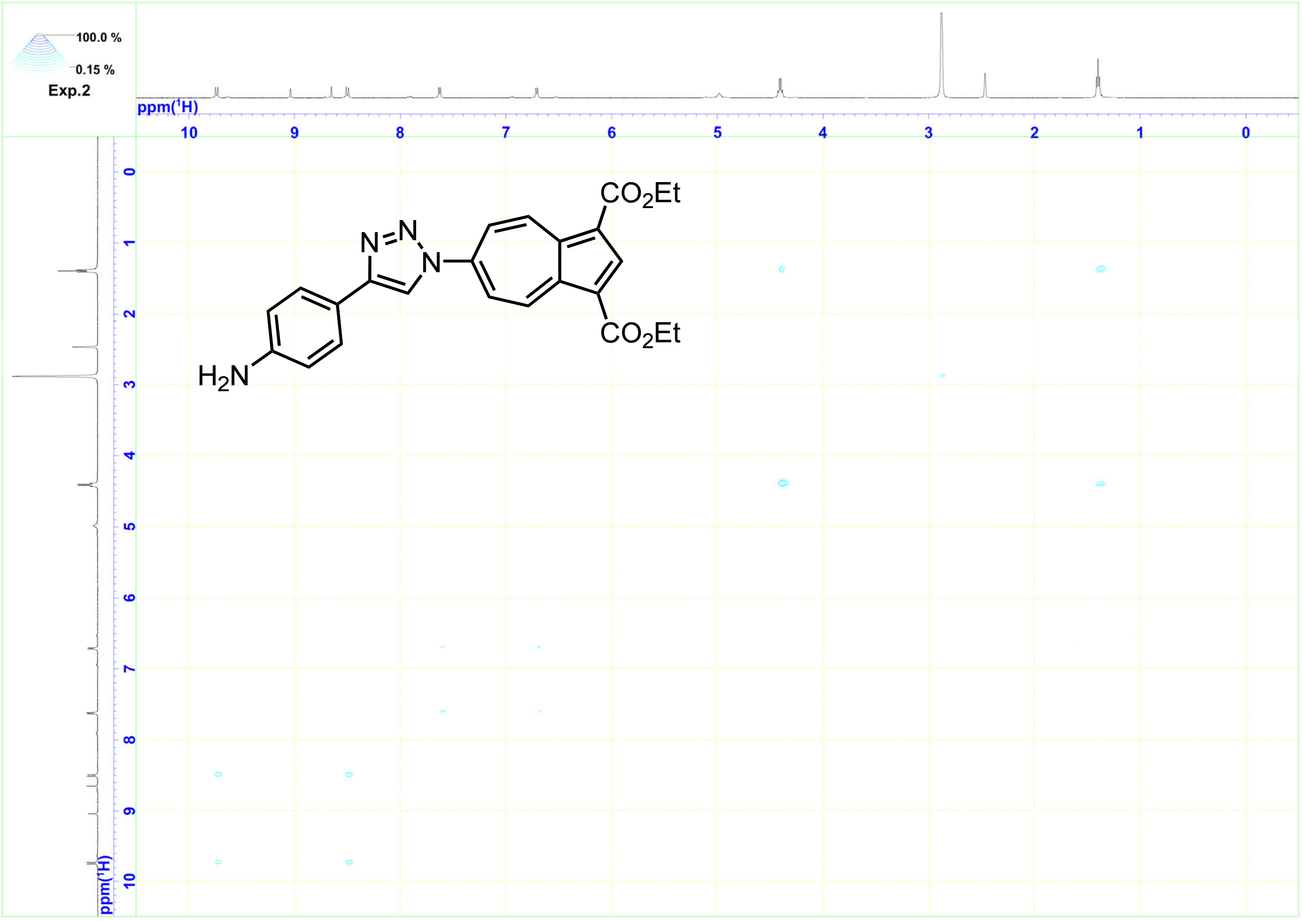

Figure S35. COSY spectrum of **6b** in DMSO-d_6 (500 MHz).

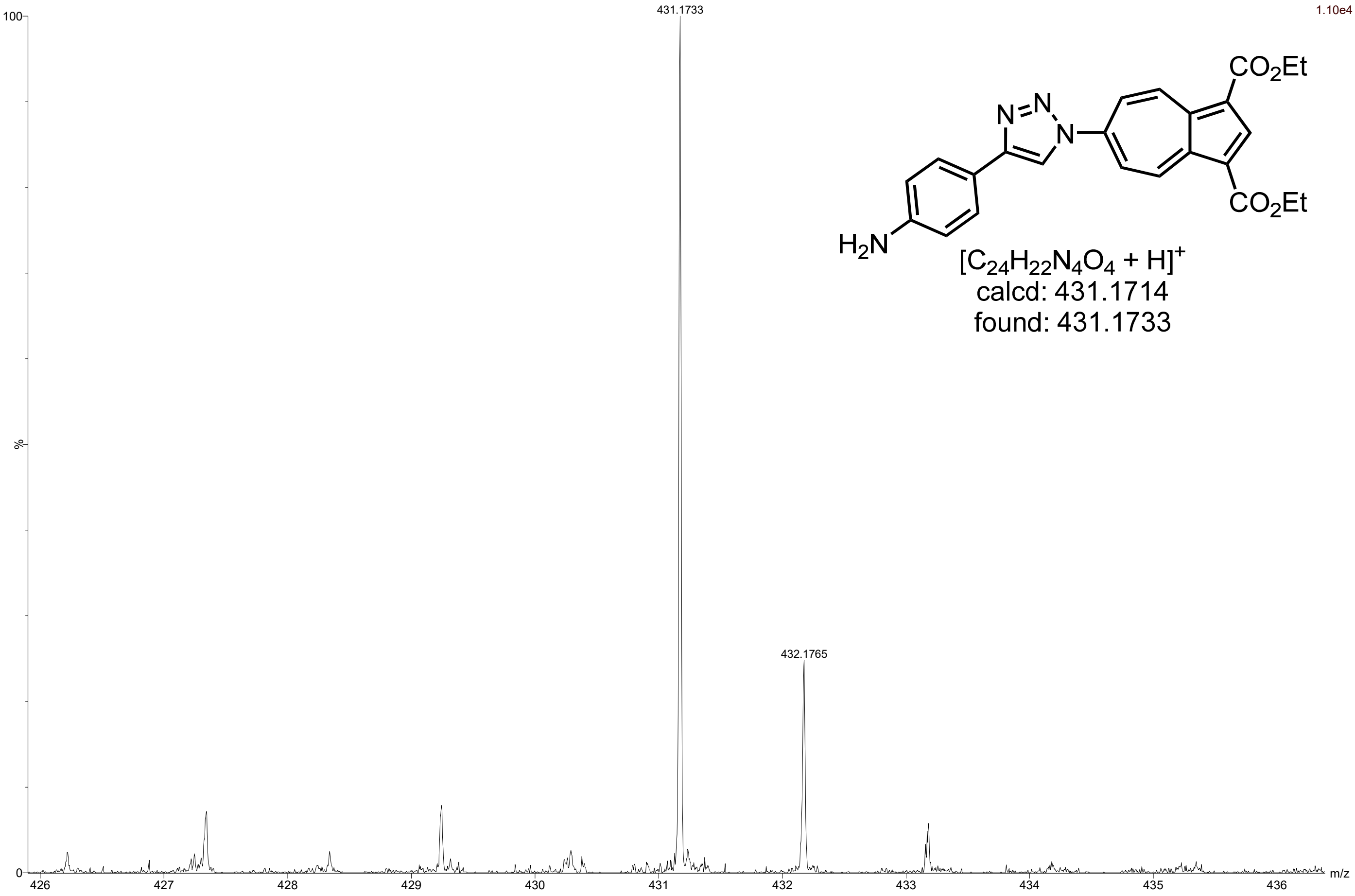

Figure S36. HRMS (ESI-TOF, positive) of **6b**.

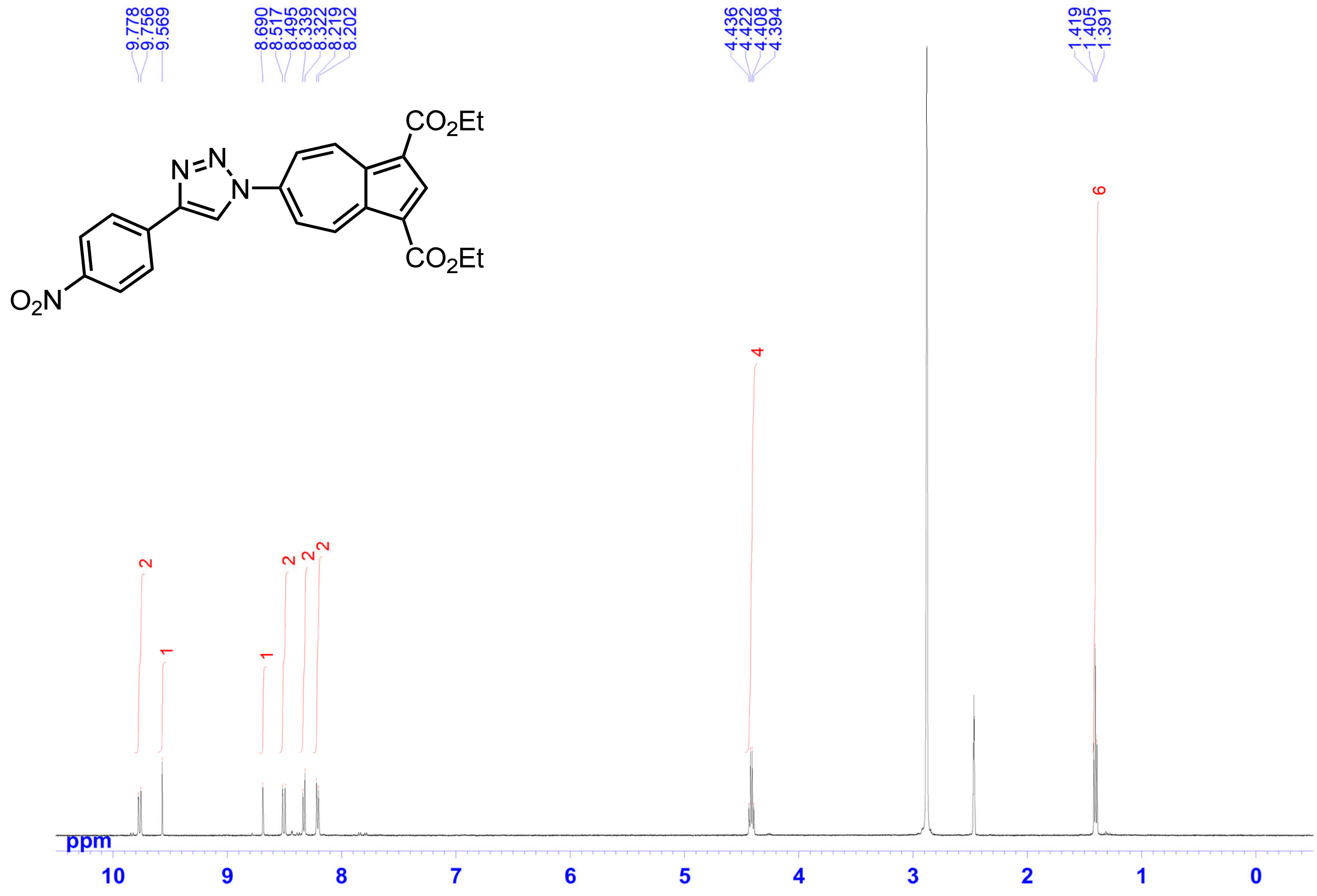

Figure S37. ^{1}H NMR spectrum of **6c** in DMSO-*d*6 (500 MHz).

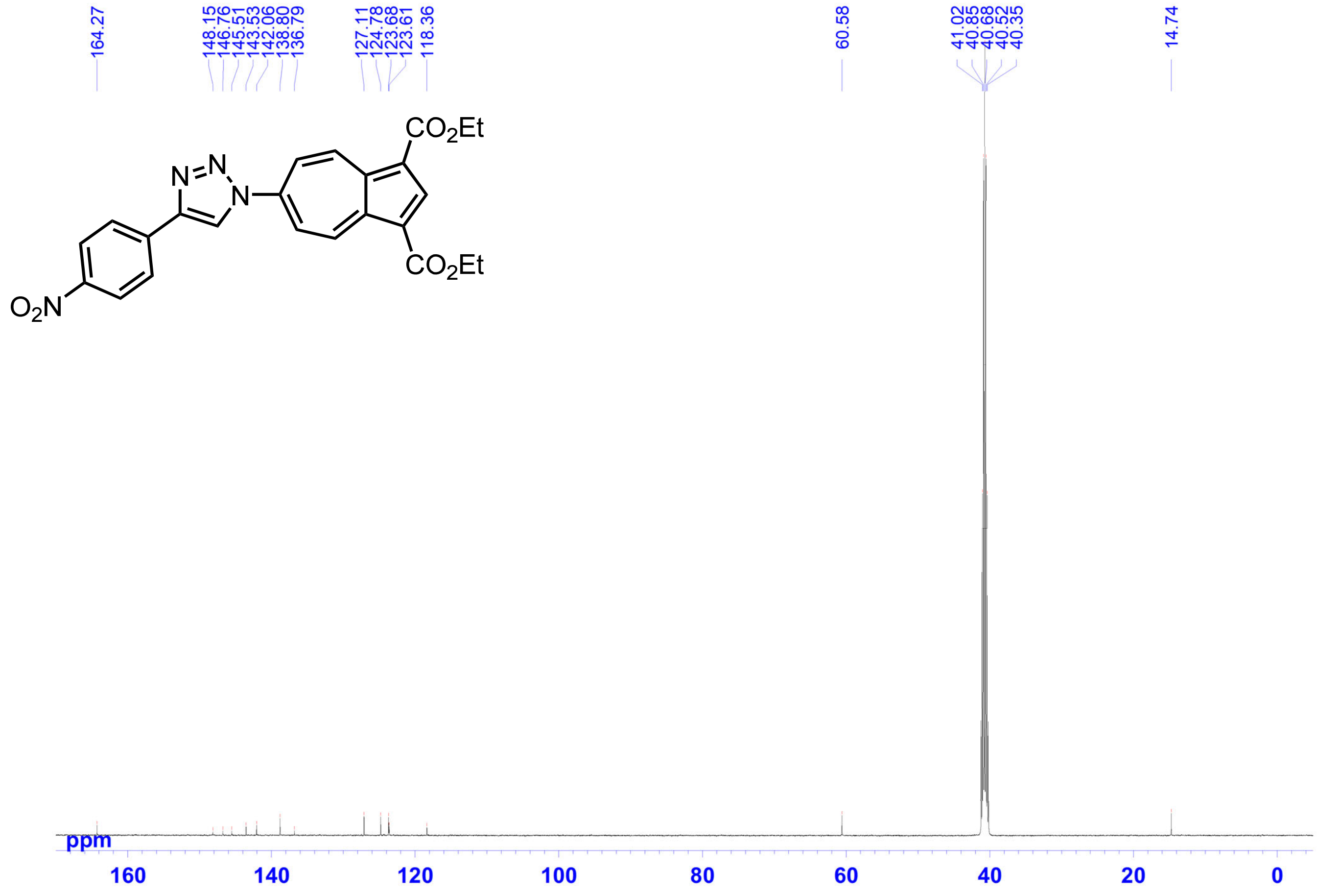

Figure S38. ^{13}C{^{1}H} NMR spectrum of **6c** in DMSO-*d*6 (126 MHz).

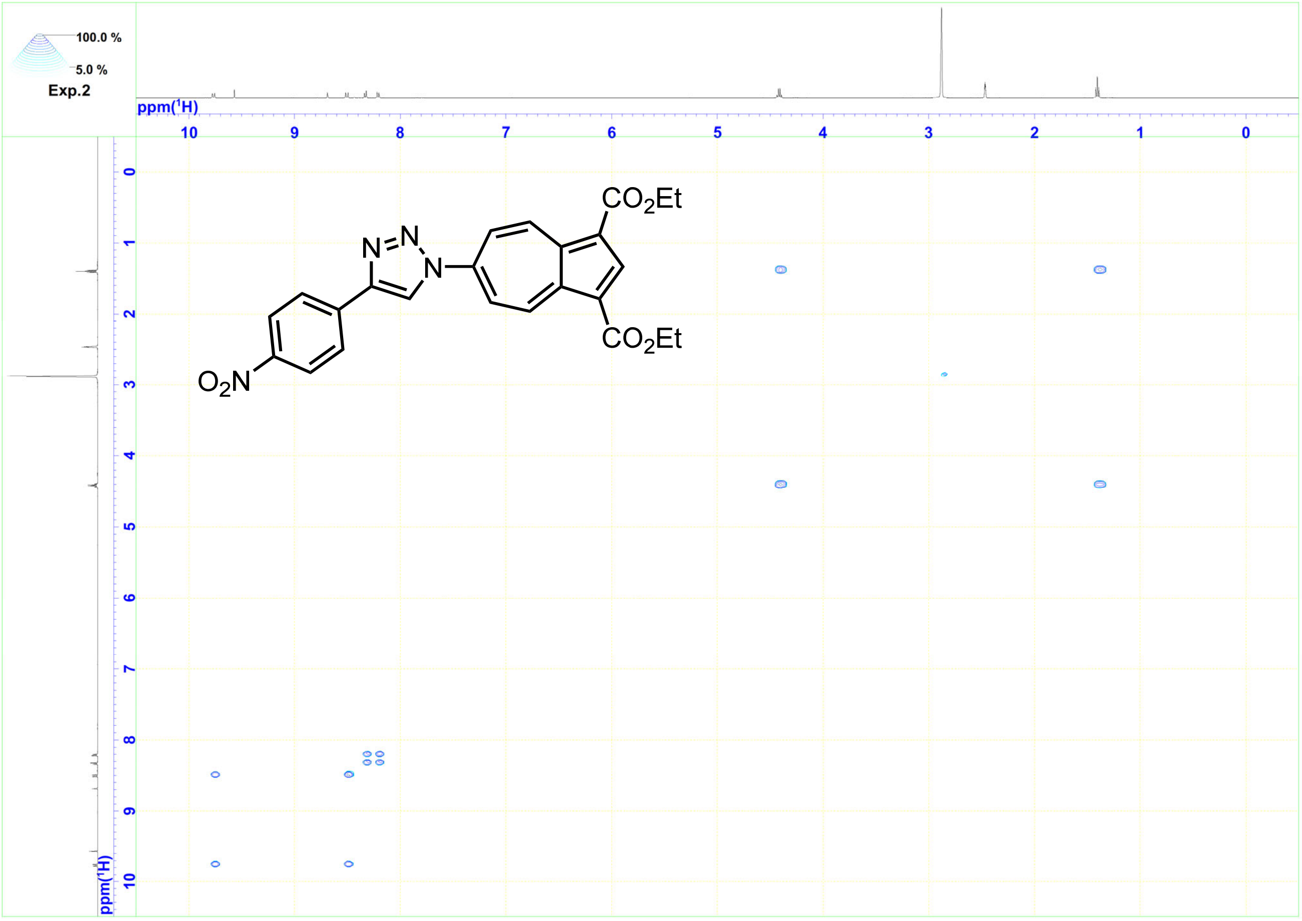

Figure S39. COSY spectrum of **6c** in DMSO-*d*6 (500 MHz).

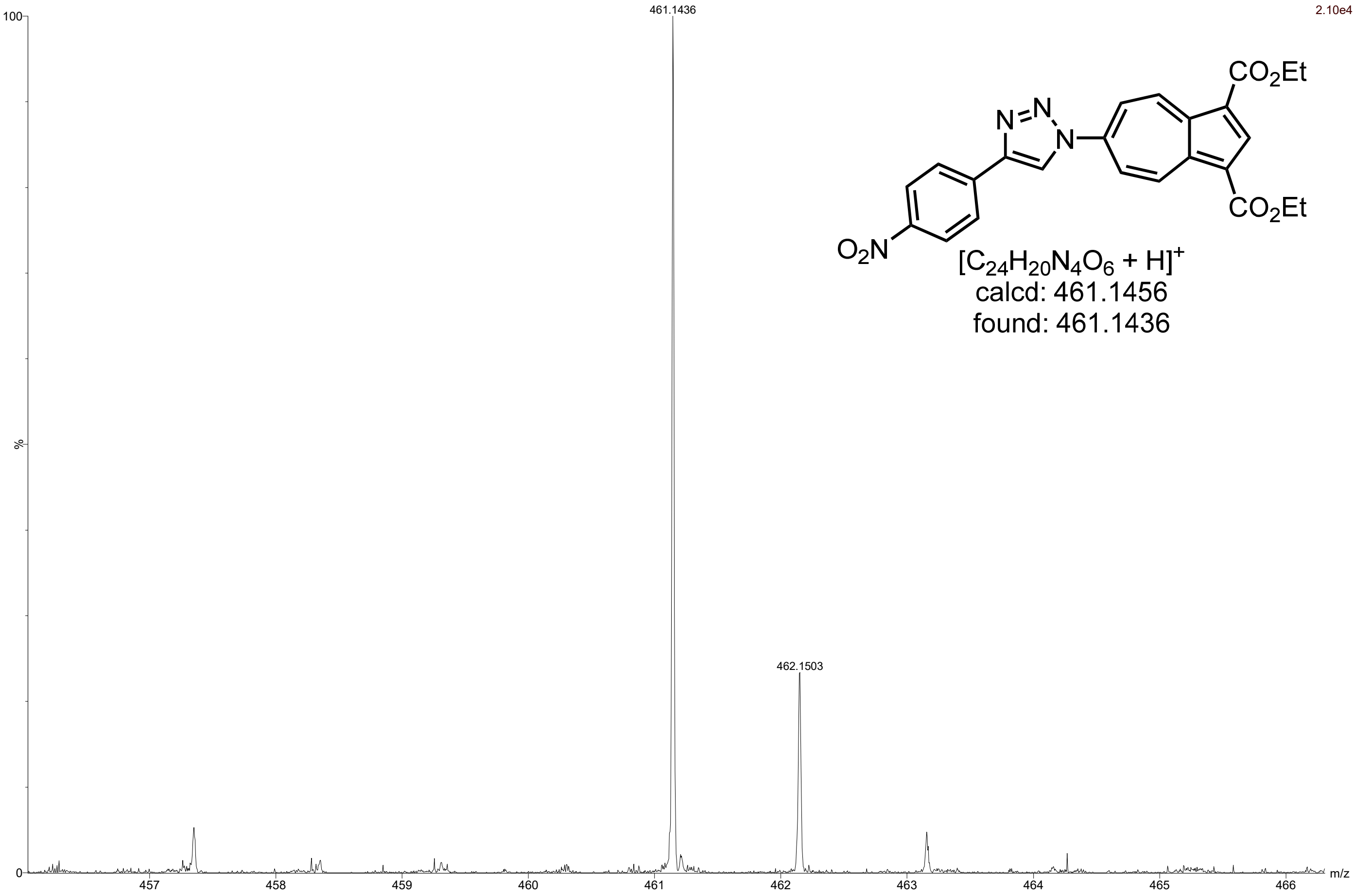

Figure S40. HRMS (ESI-TOF, positive) of **6c**.

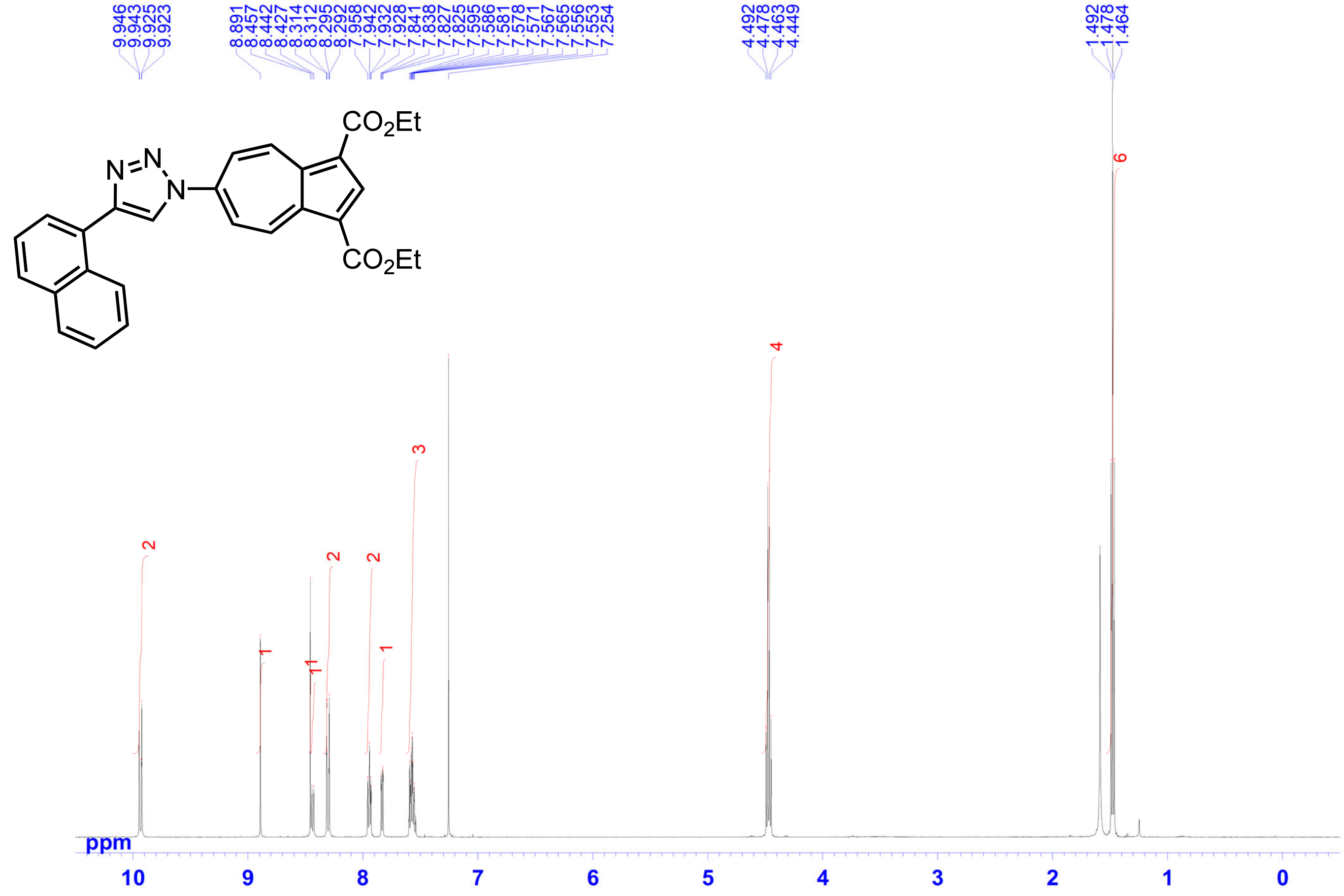

Figure S41. ^{1}H NMR spectrum of **6d** in $CDCl_3$ (500 MHz).

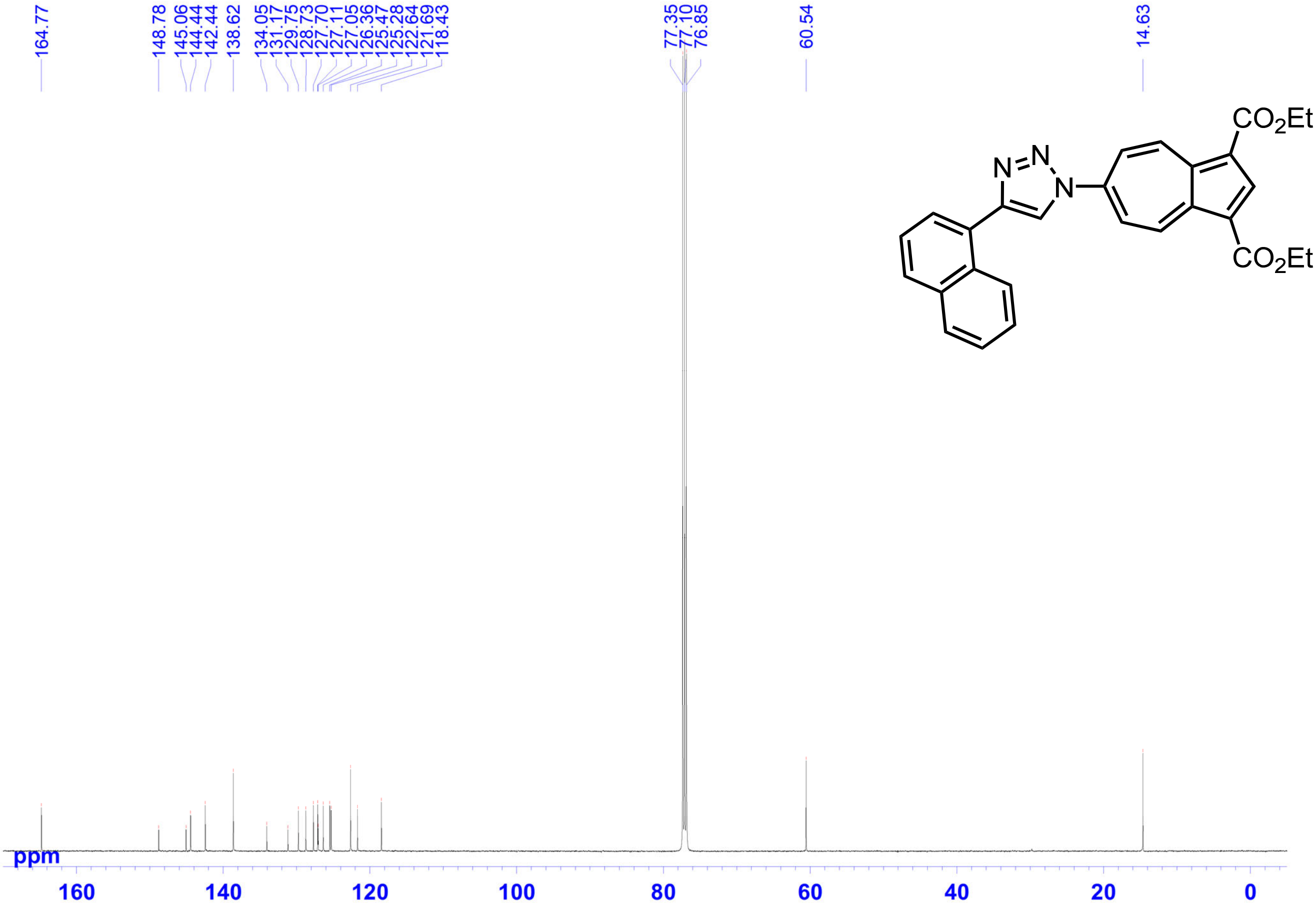

Figure S42. $^{13}C\{^{1}H\}$ NMR spectrum of **6d** in $CDCl_3$ (126 MHz).

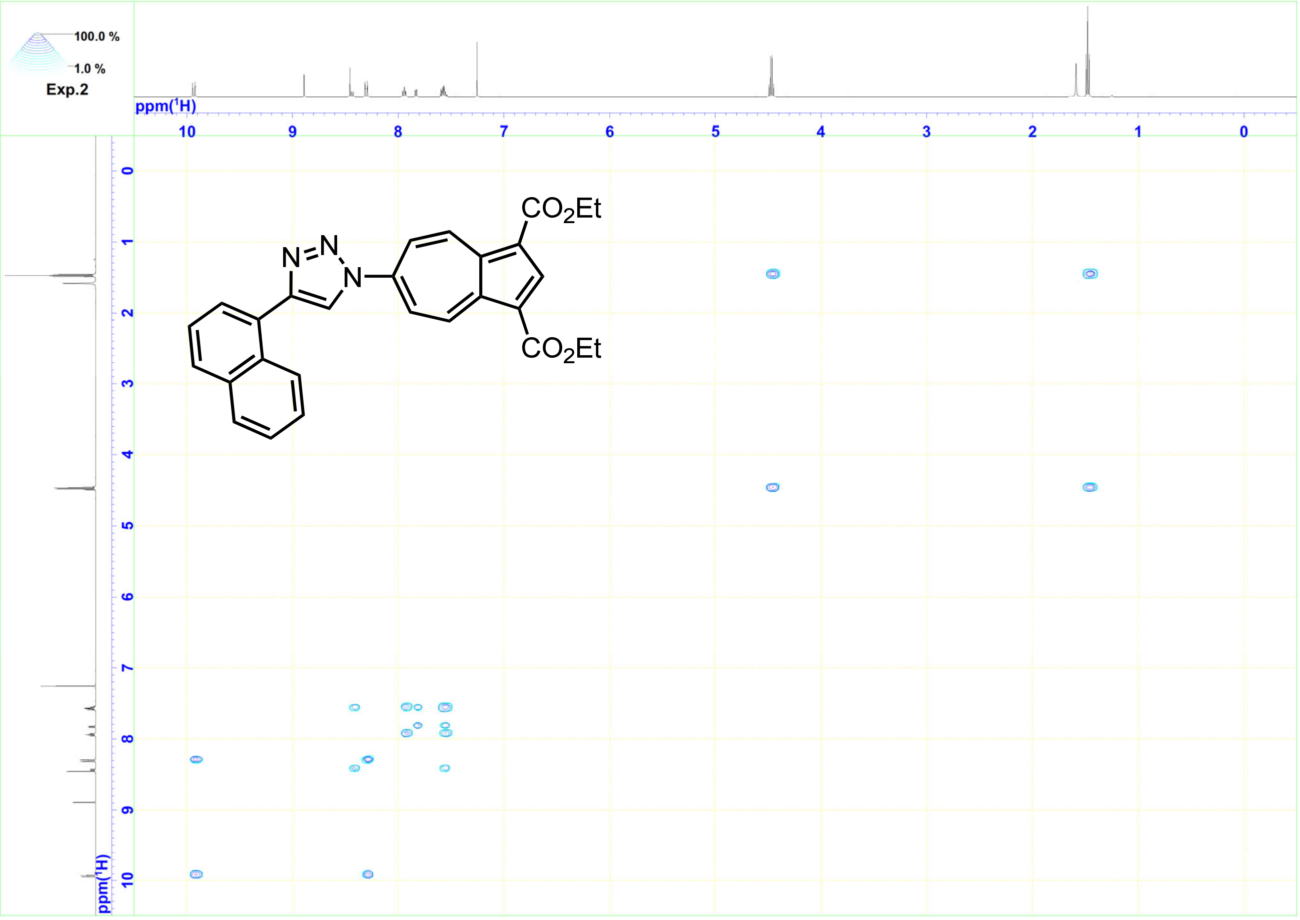

Figure S43. COSY spectrum of **6d** in $CDCl_3$ (500 MHz).

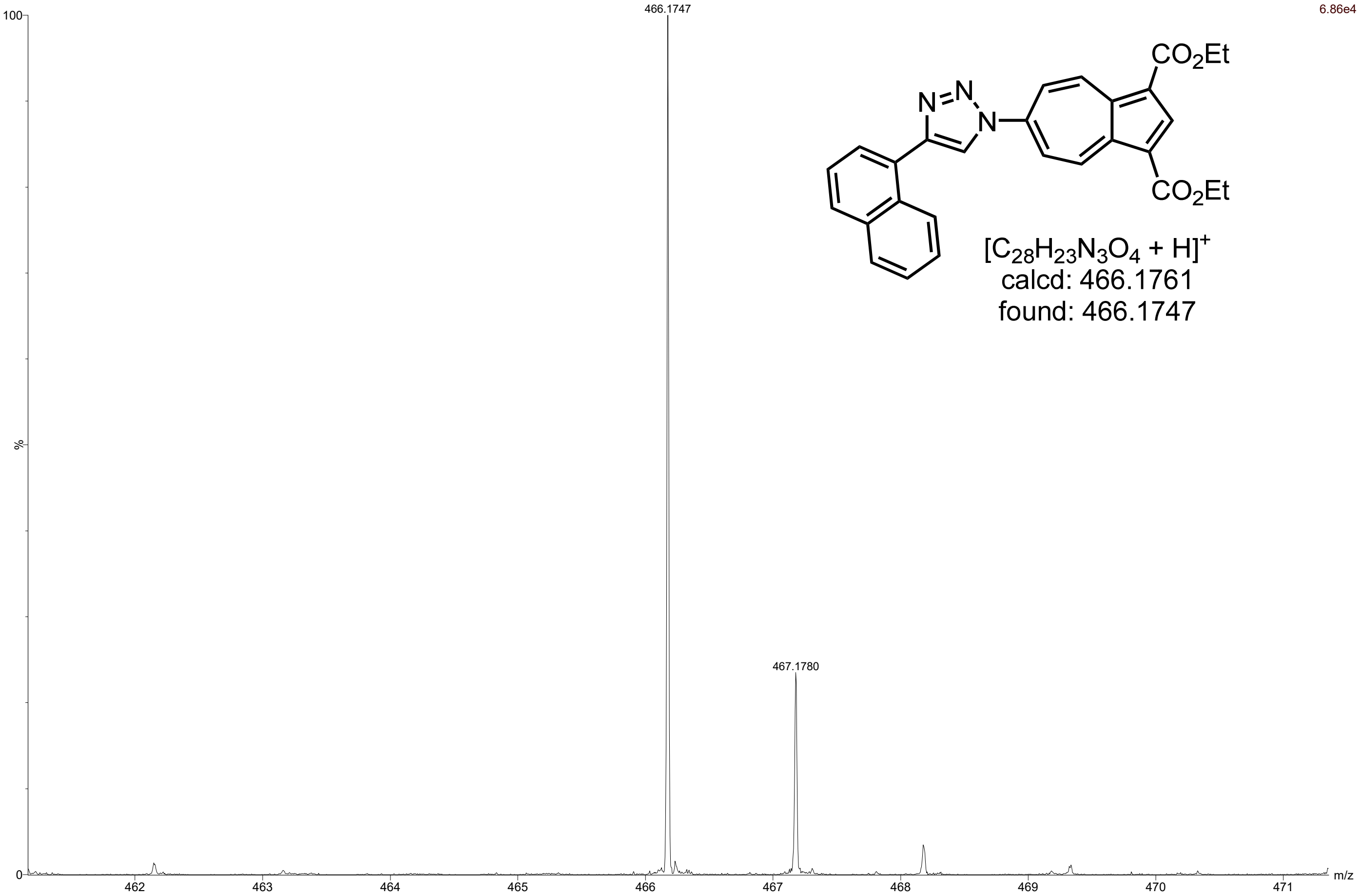

Figure S44. HRMS (ESI-TOF, positive) of **6d**.

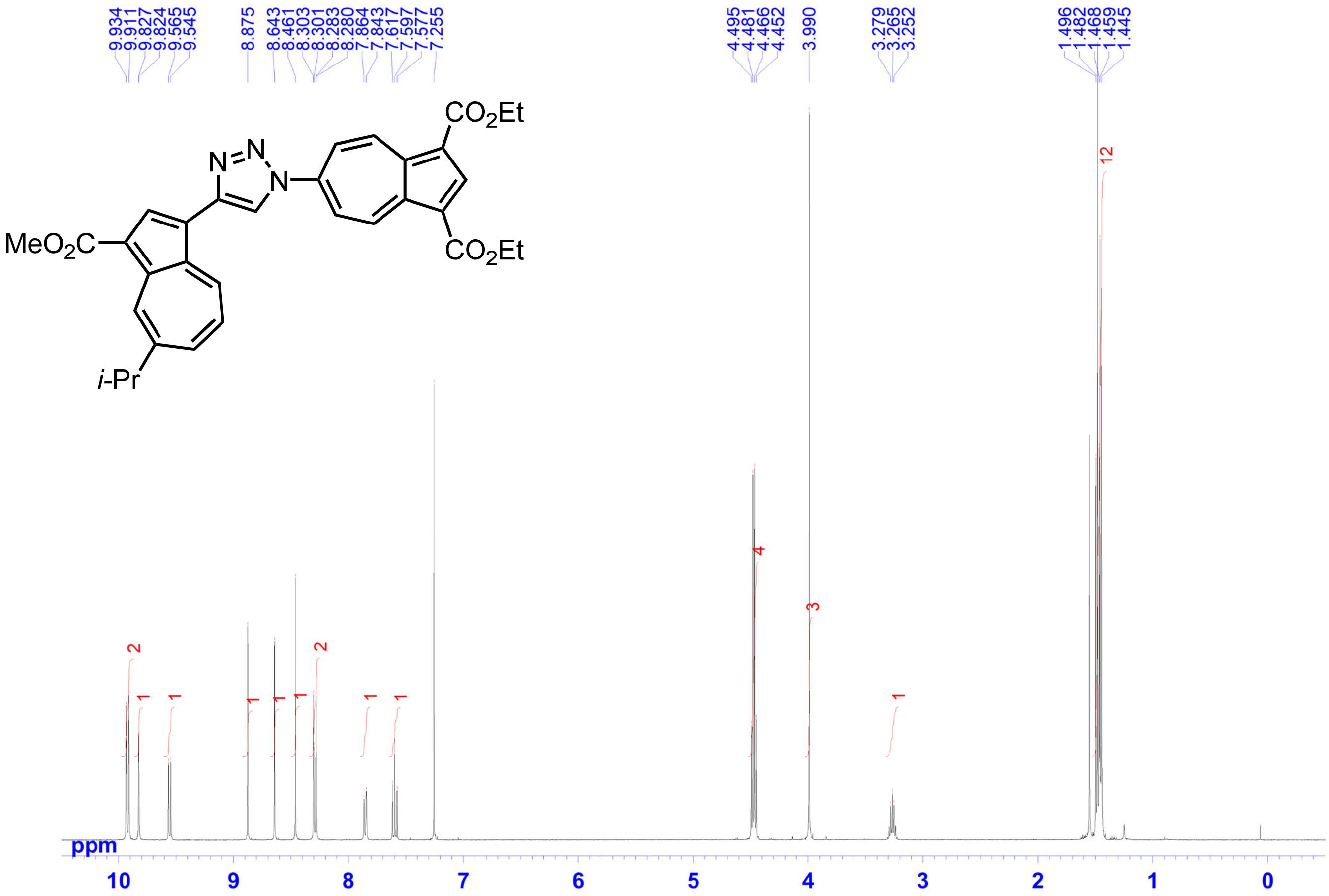

Figure S45. ^{1}H NMR spectrum of **6e** in $CDCl_3$ (500 MHz).

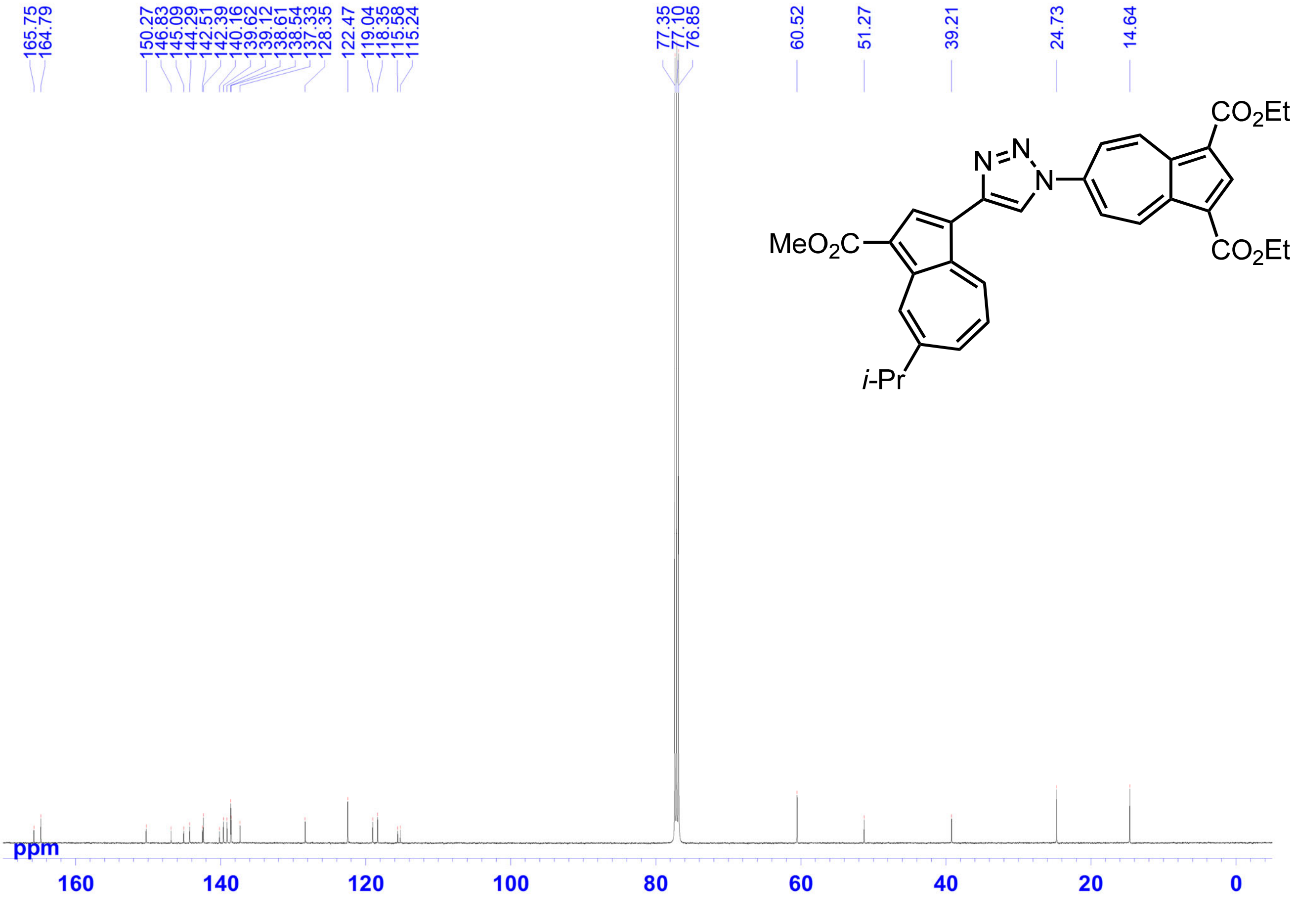

Figure S46. $^{13}C\{^{1}H\}$ NMR spectrum of **6e** in $CDCl_3$ (126 MHz).

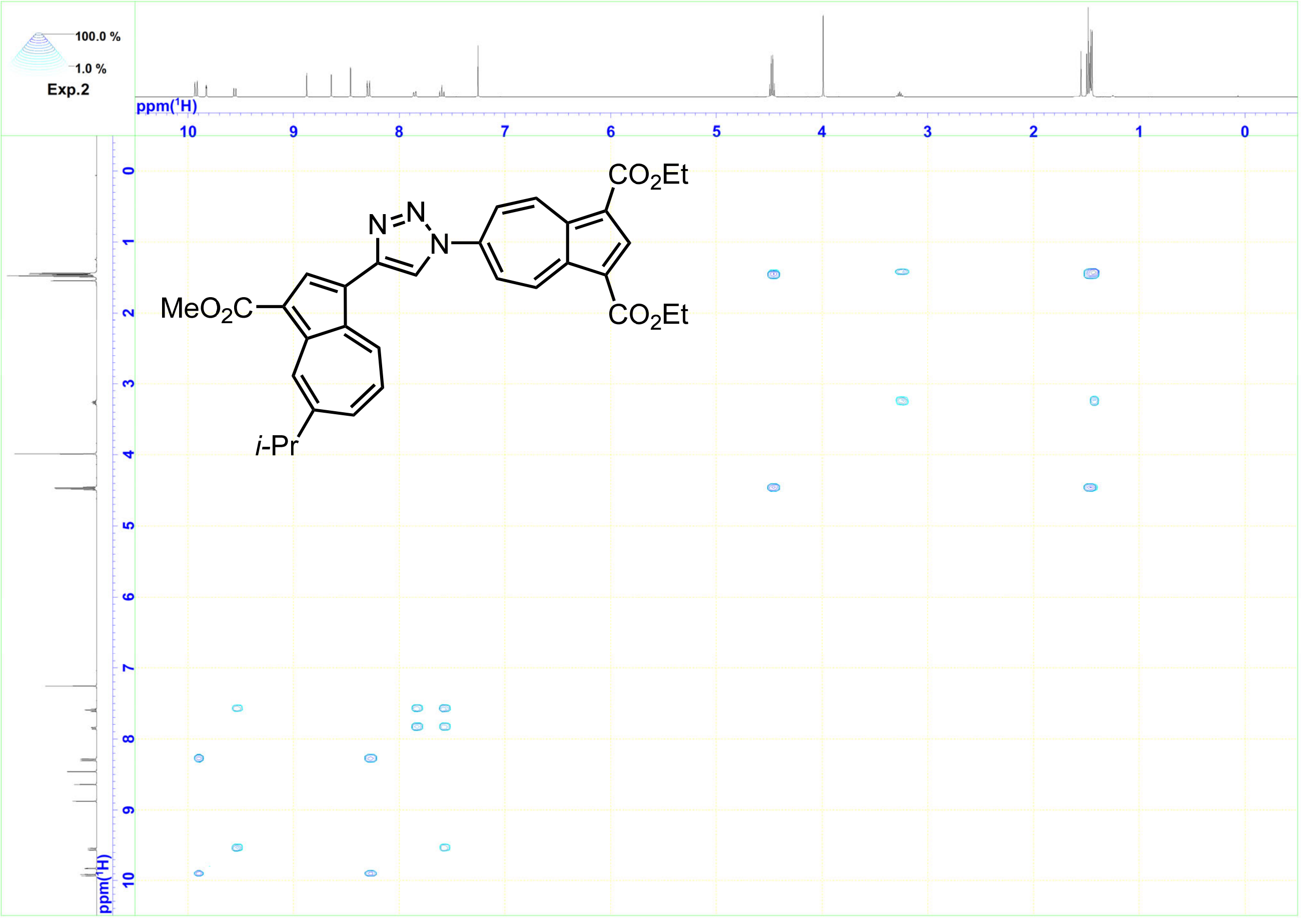

Figure S47. COSY spectrum of **6e** in CDCl3 (500 MHz).

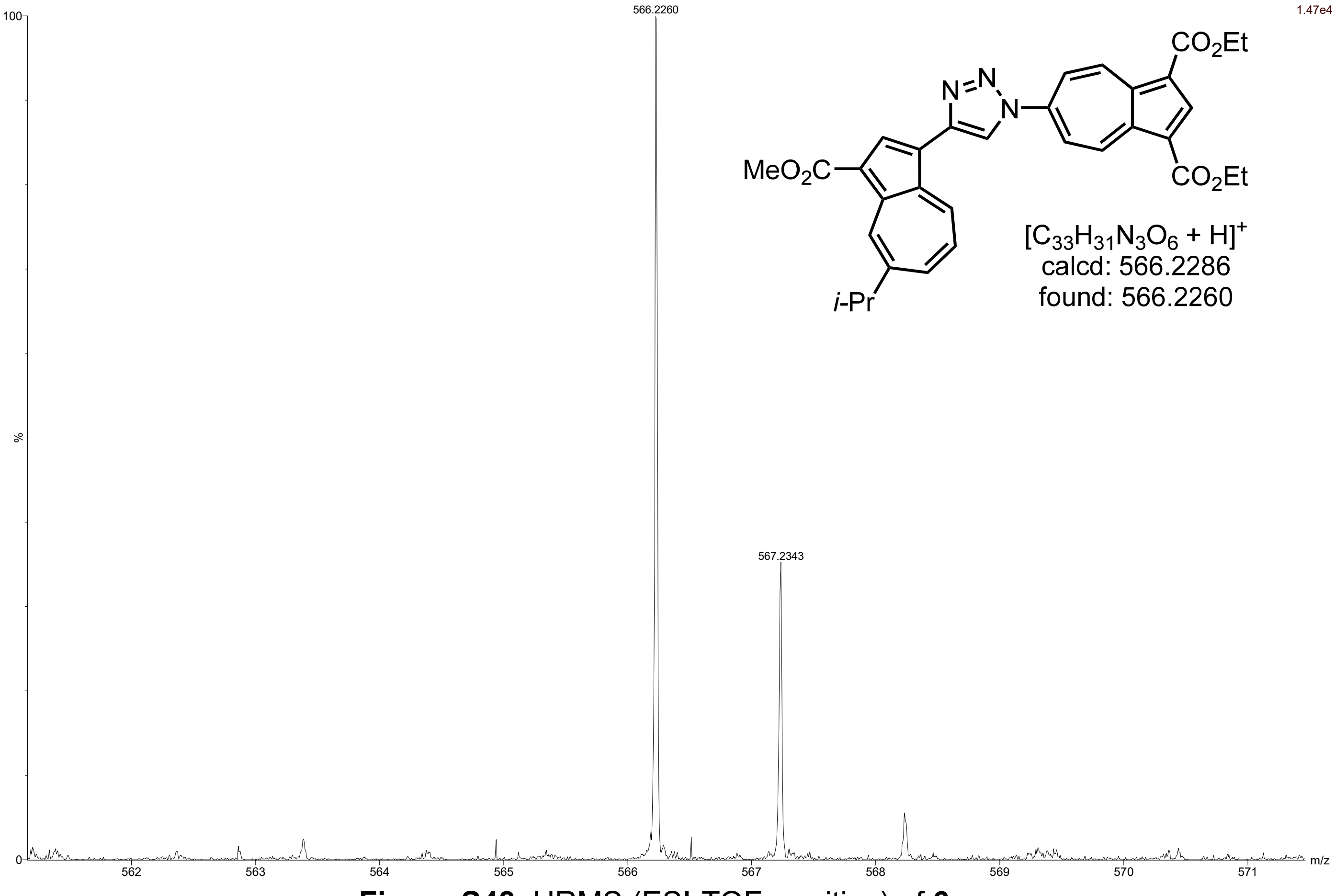

Figure S48. HRMS (ESI-TOF, positive) of **6e**.

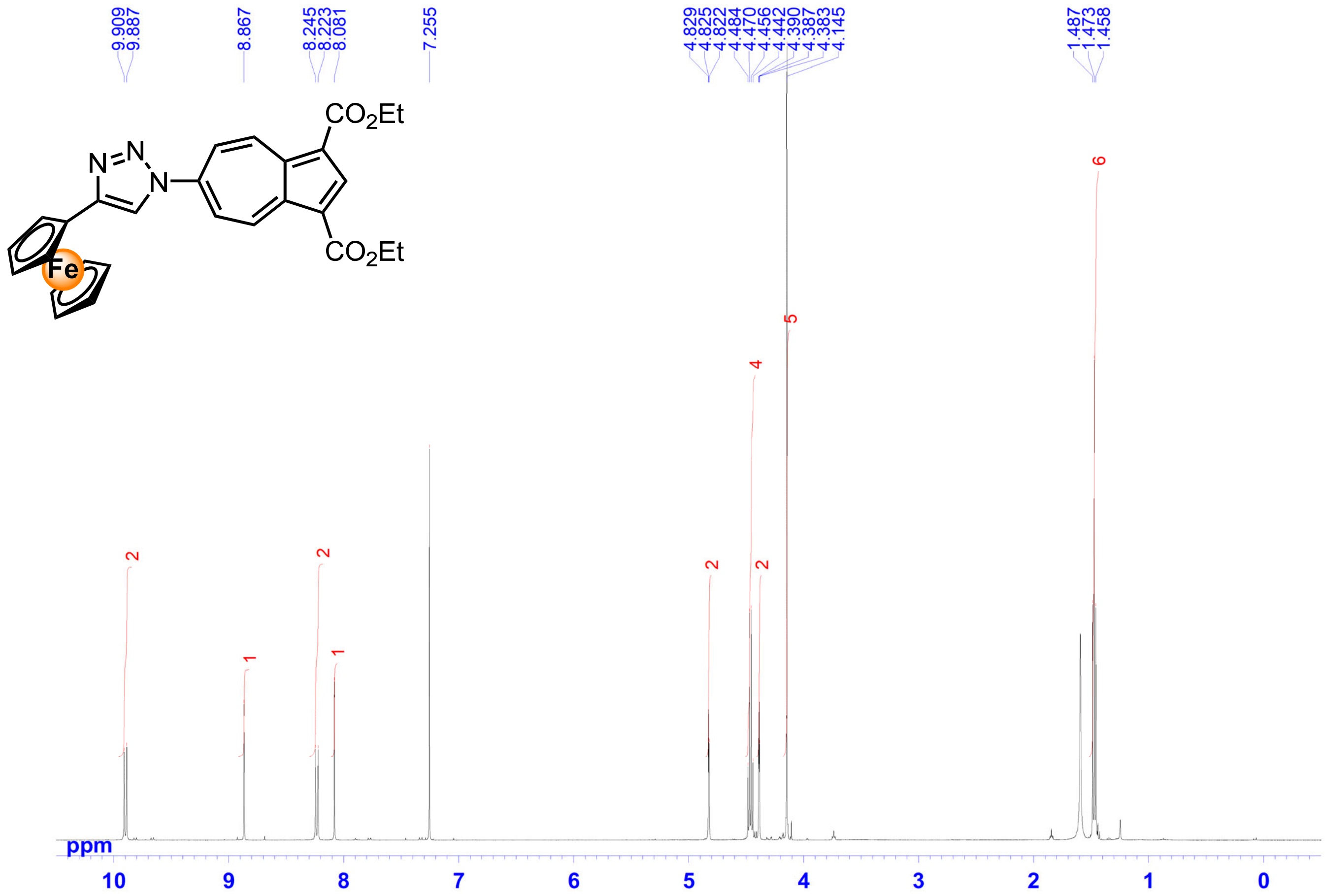

Figure S49. ^{1}H NMR spectrum of **6f** in $CDCl_3$ (500 MHz).

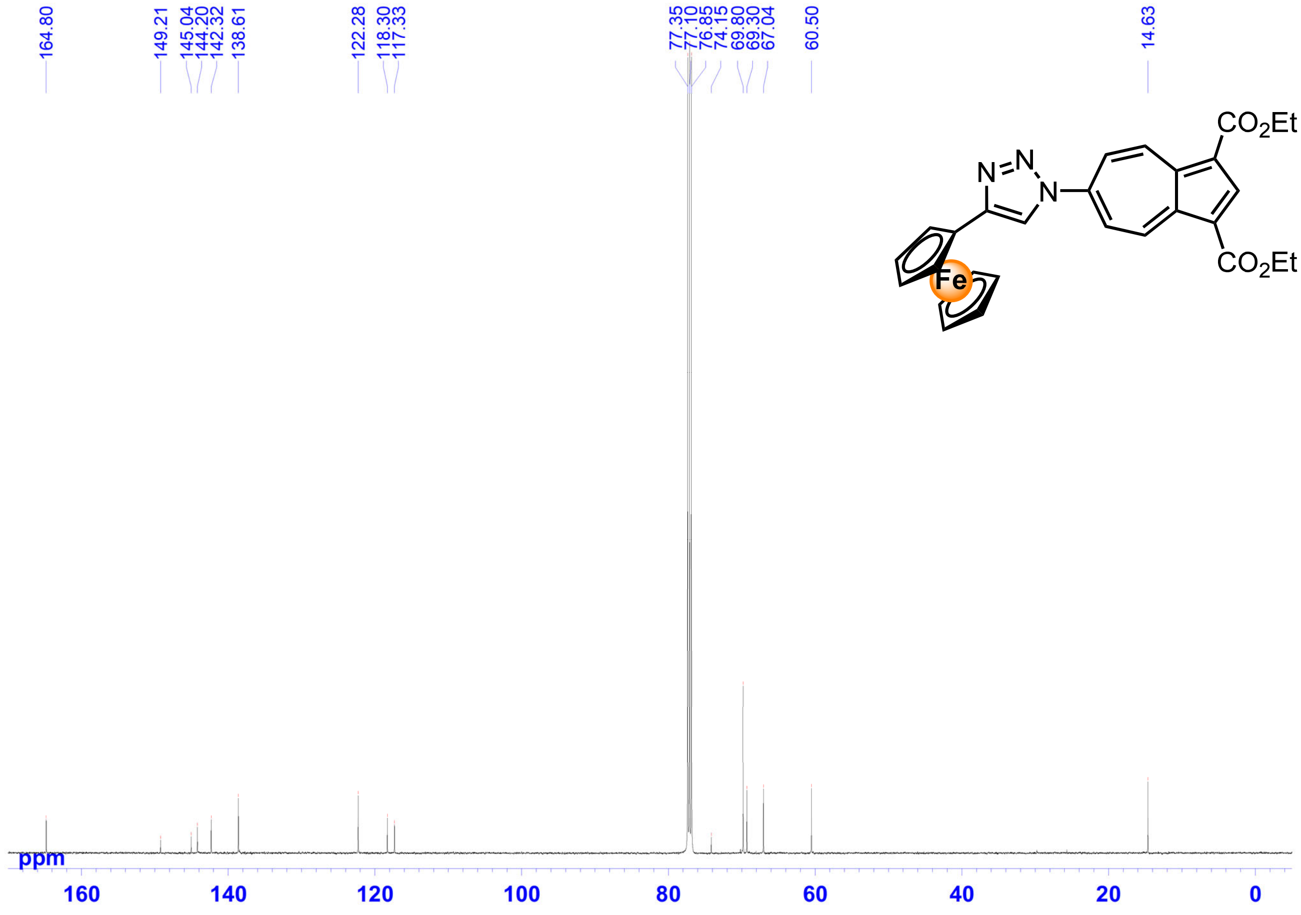

Figure S50. $^{13}C\{^{1}H\}$ NMR spectrum of **6f** in $CDCl_3$ (126 MHz).

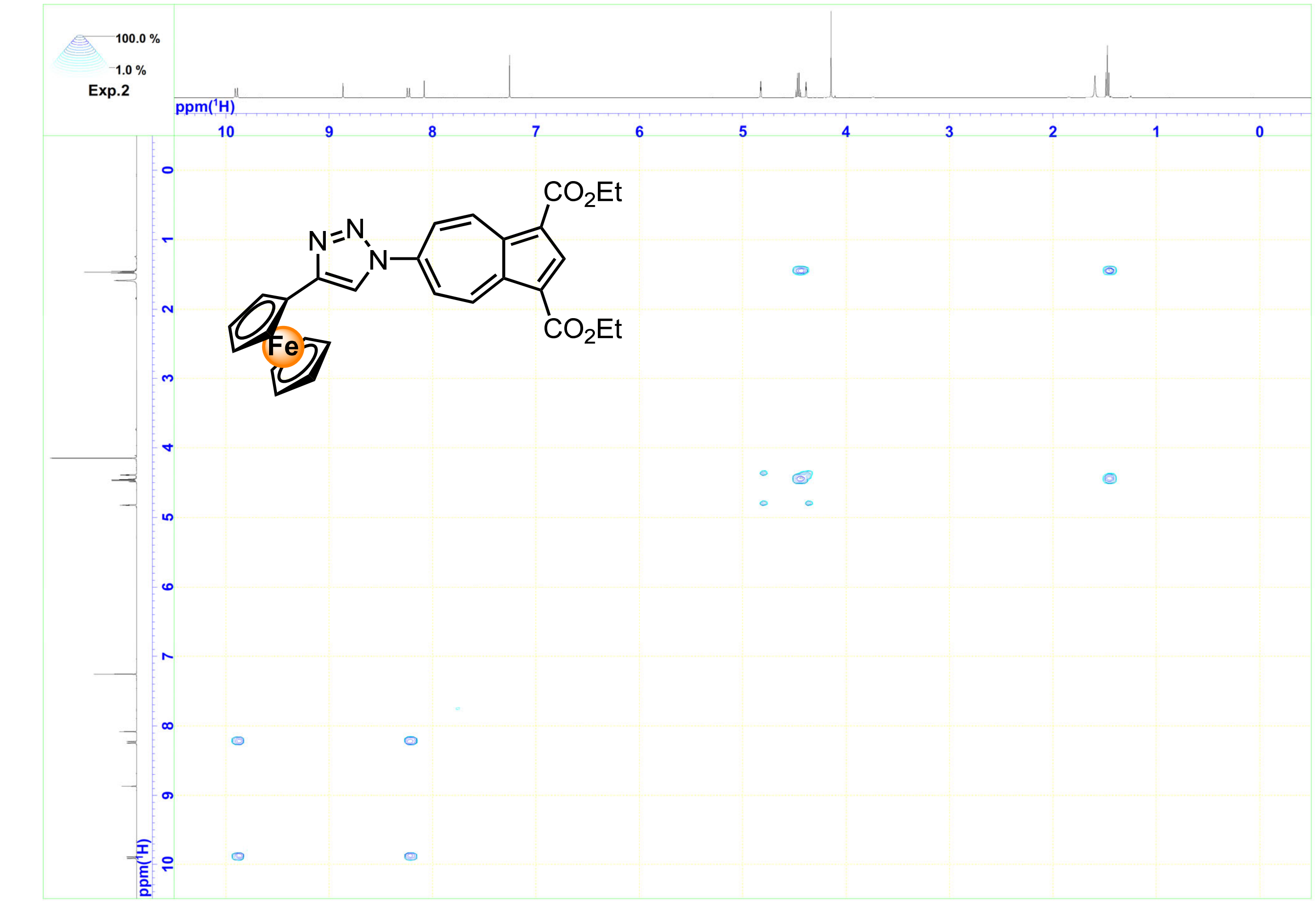

Figure S51. COSY spectrum of **6f** in $CDCl_3$ (500 MHz).

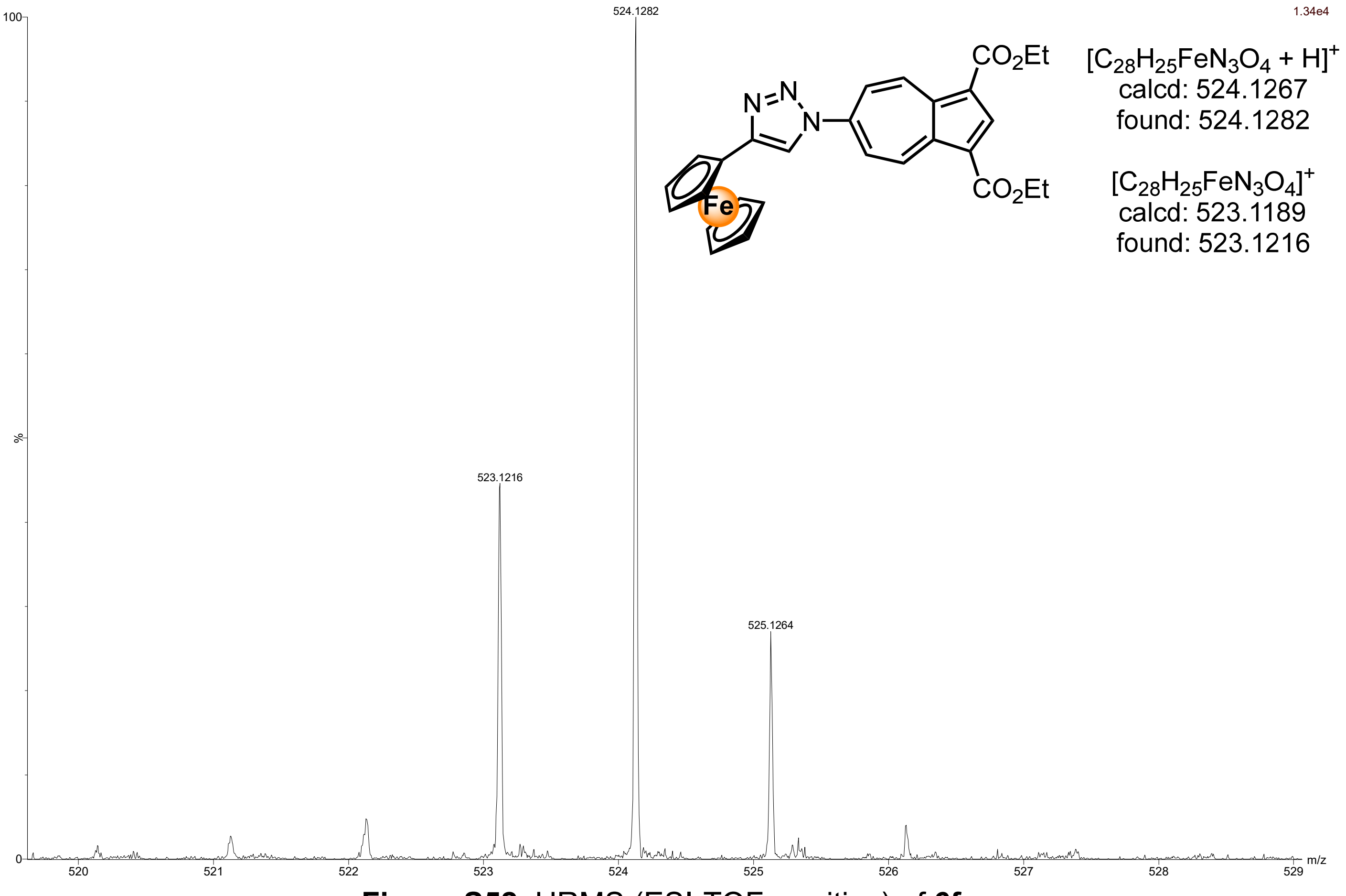

Figure S52. HRMS (ESI-TOF, positive) of **6f**.

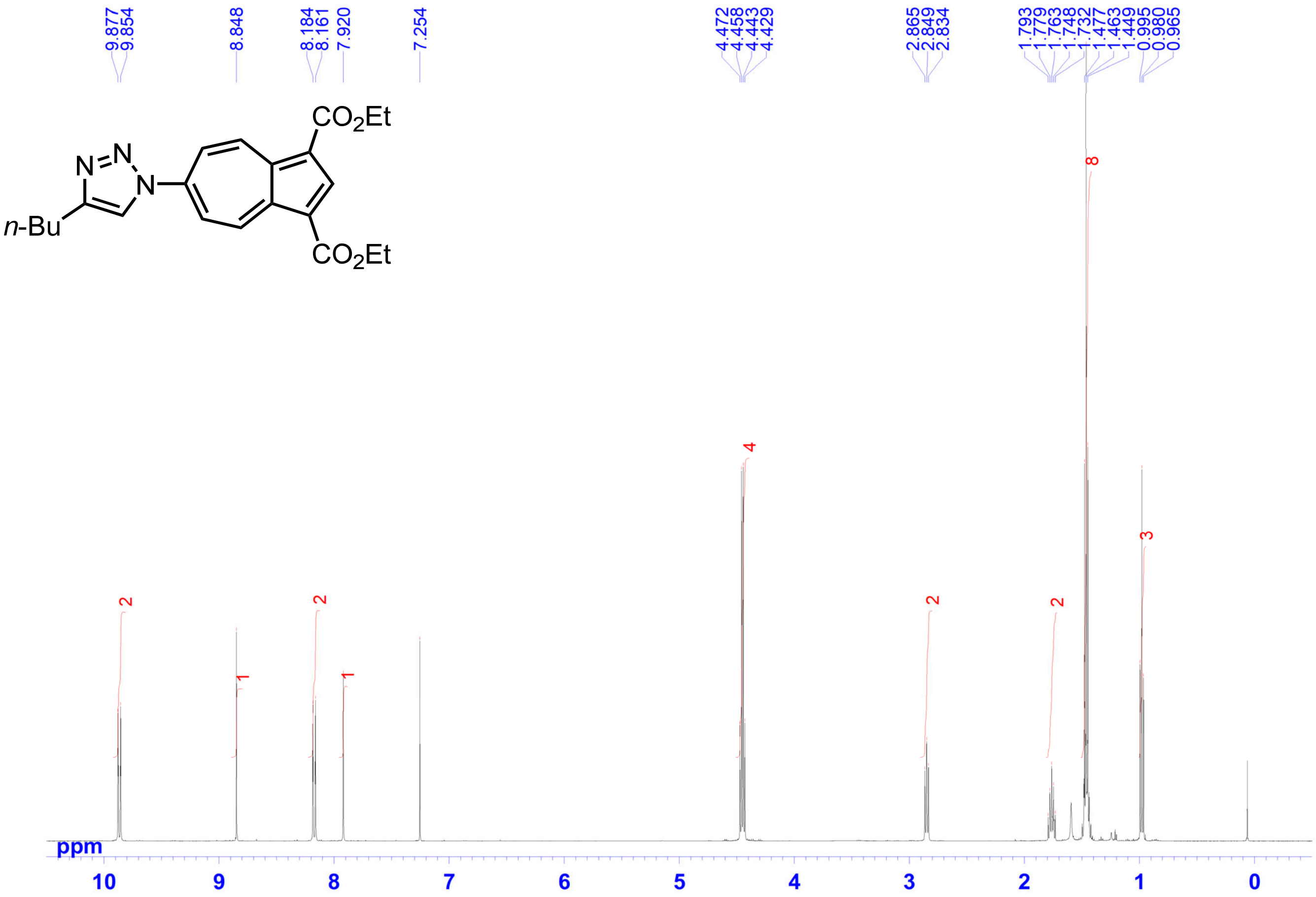

Figure S53. ^{1}H NMR spectrum of **6g** in $CDCl_3$ (500 MHz).

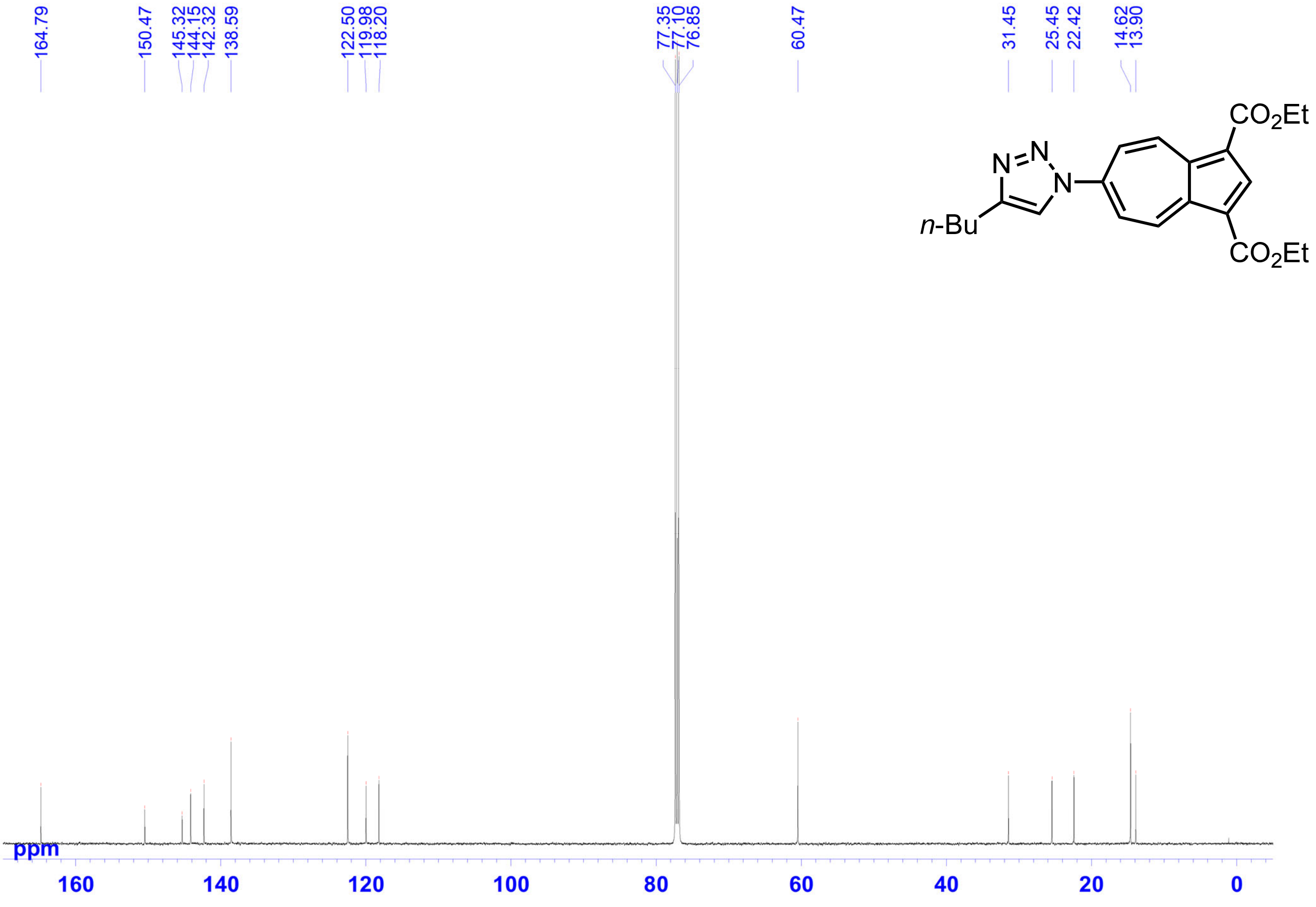

Figure S54. $^{13}C\{^{1}H\}$ NMR spectrum of **6g** in $CDCl_3$ (126 MHz).

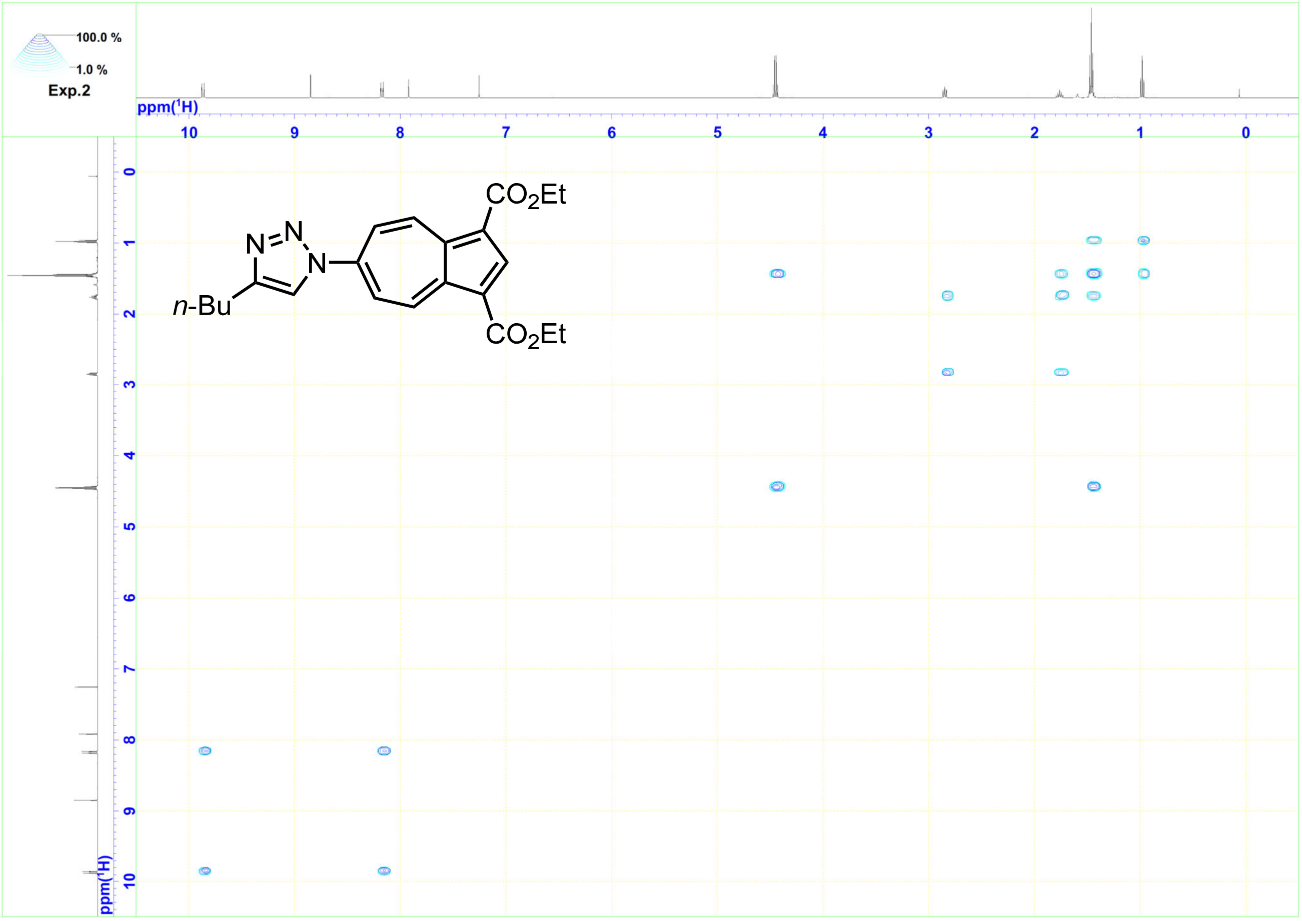

Figure S55. COSY spectrum of **6g** in $CDCl_3$ (500 MHz).

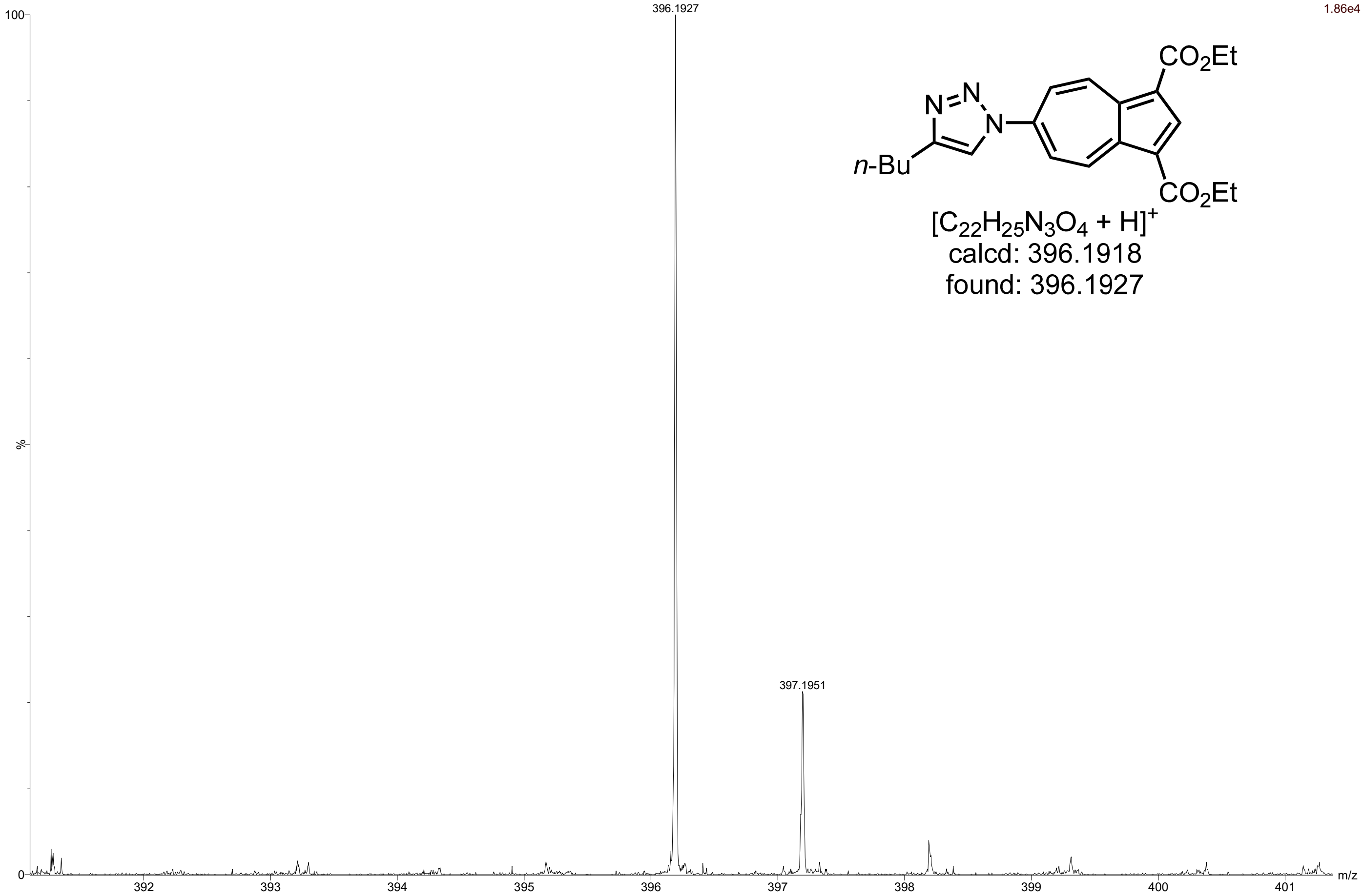

Figure S56. HRMS (ESI-TOF, positive) of **6g**.

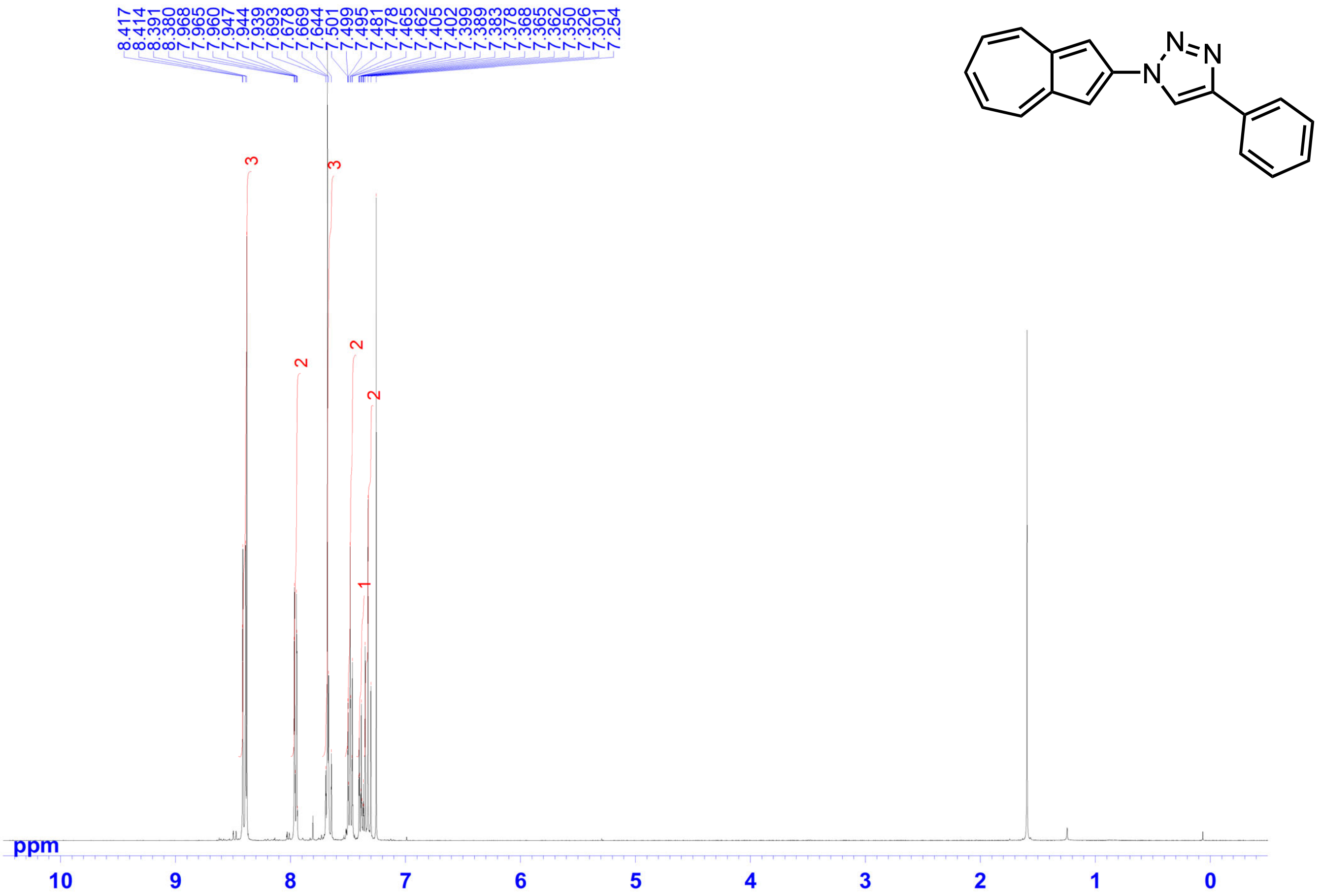

Figure S57. ^{1}H NMR spectrum of **7a** in $CDCl_3$ (400 MHz).

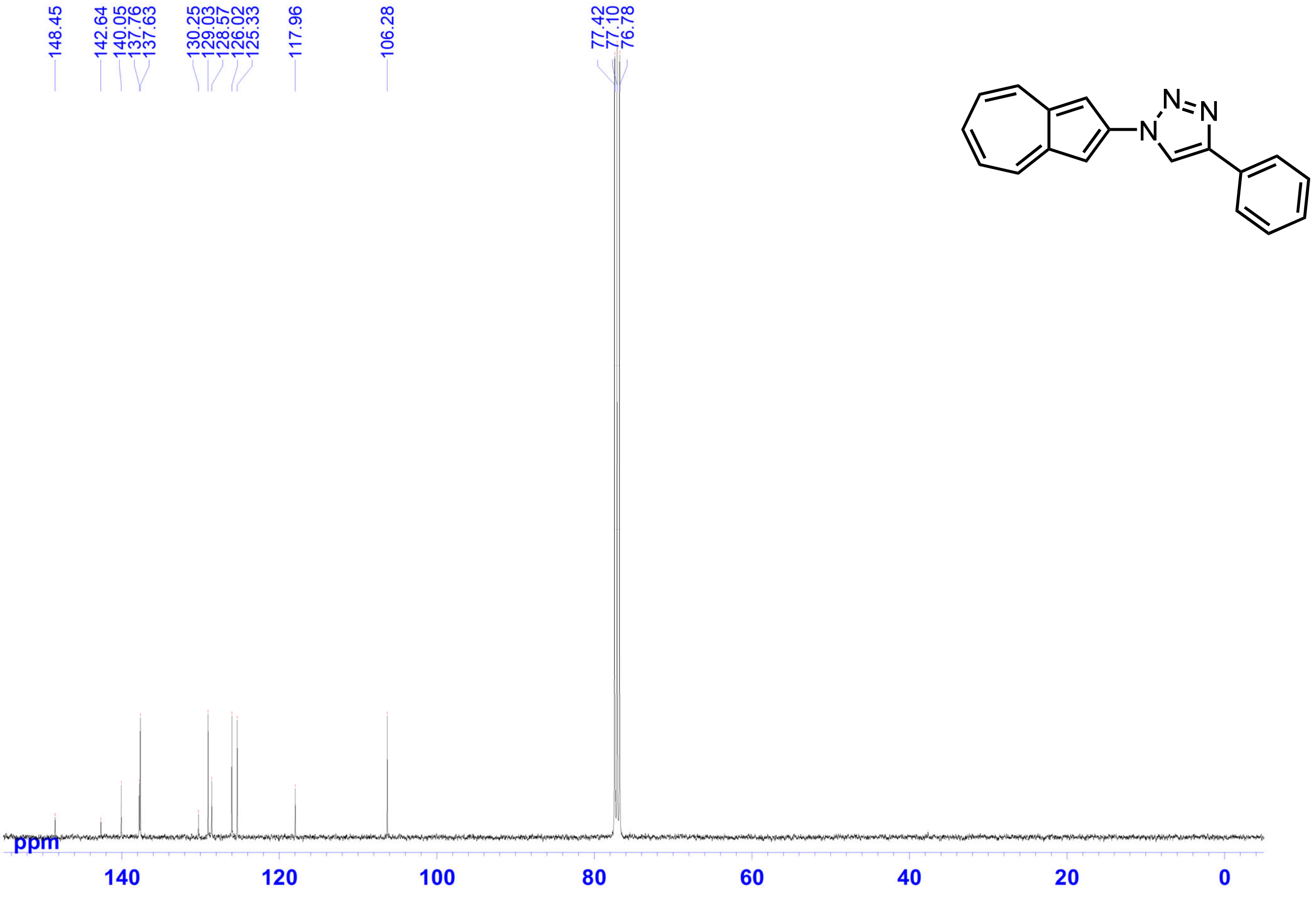

Figure S58. $^{13}C\{^1H\}$ NMR spectrum of **7a** in $CDCl_3$ (100 MHz).

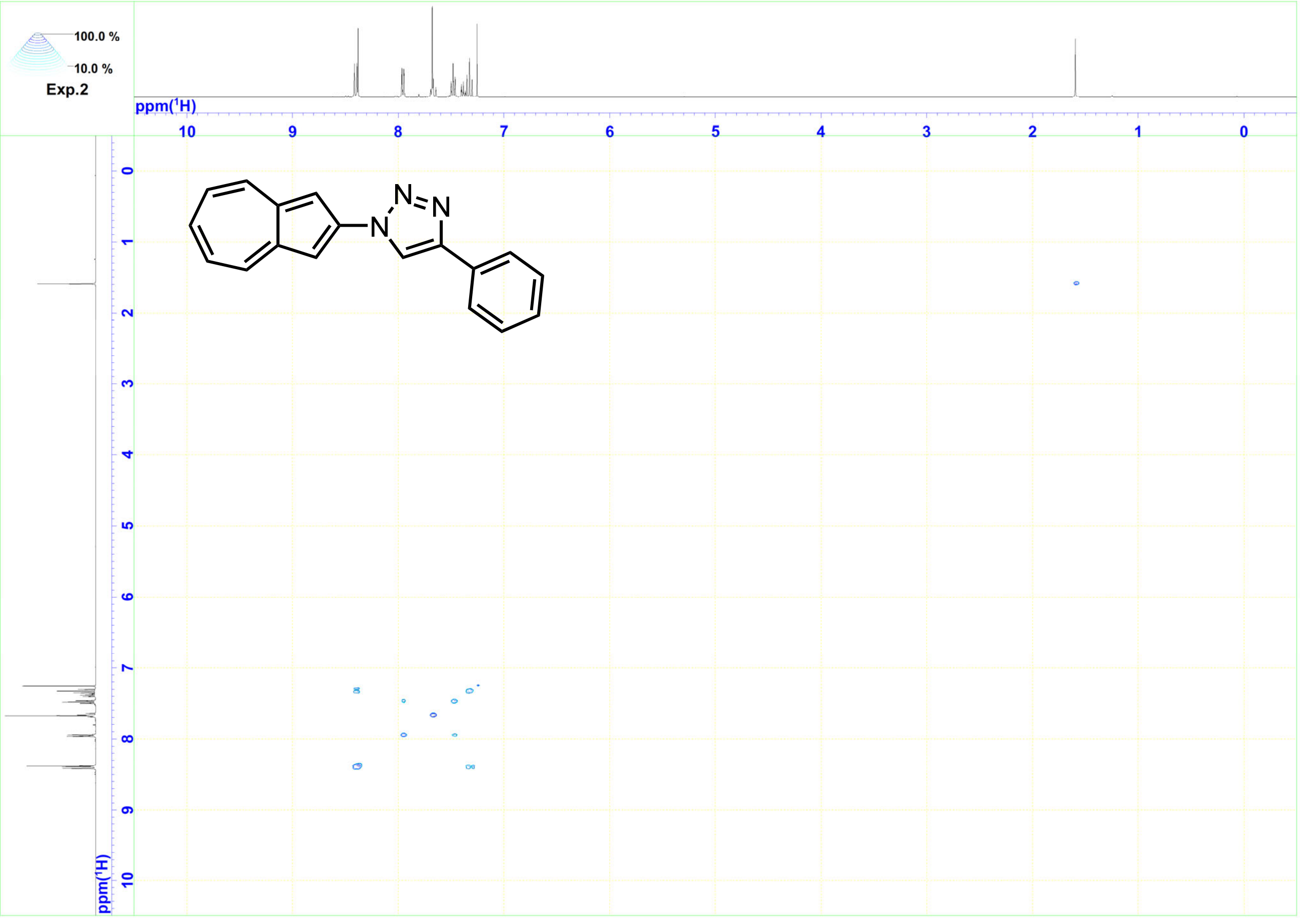

Figure S59. COSY spectrum of **7a** in $CDCl_3$ (400 MHz).

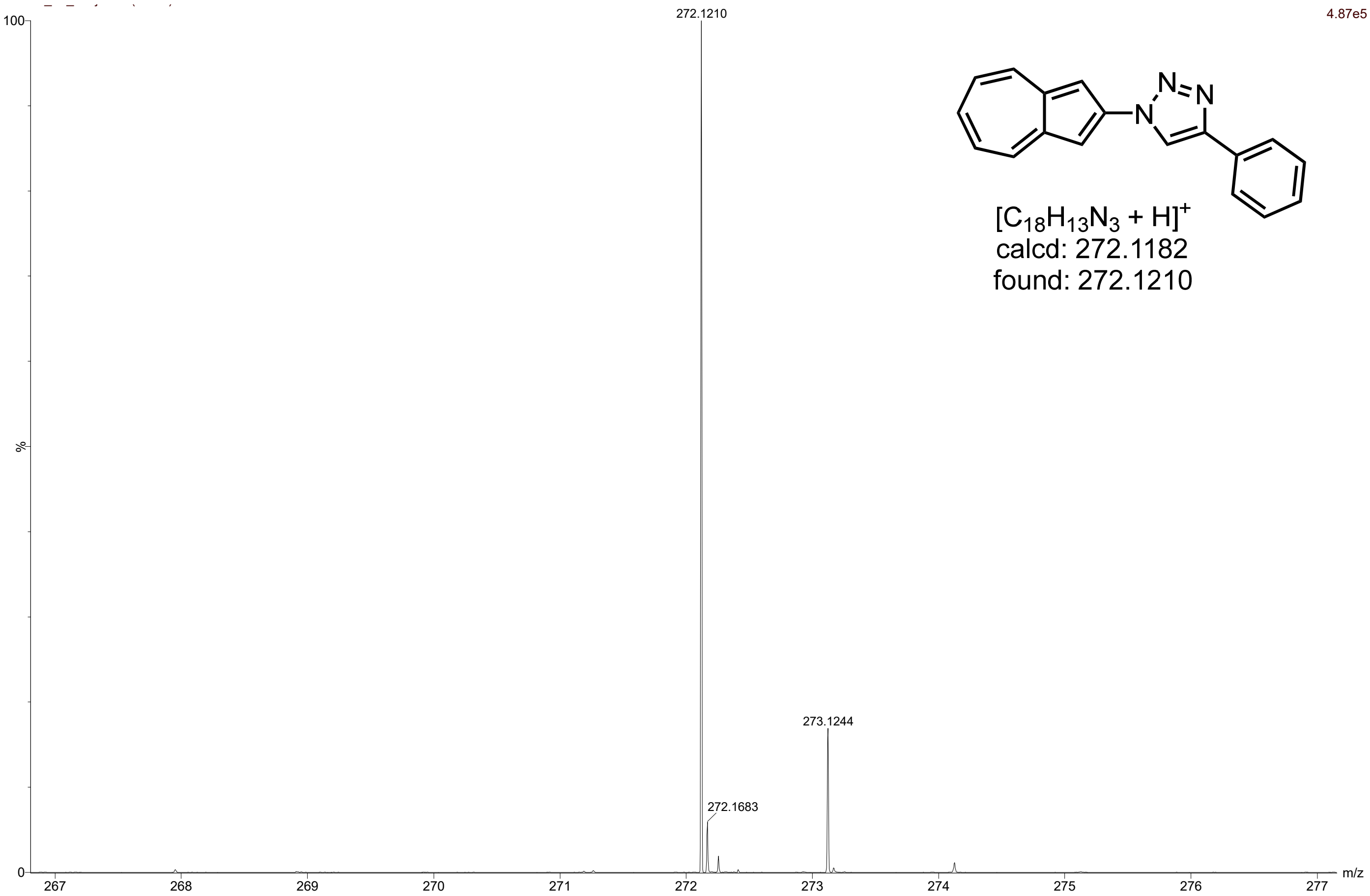

Figure S60. HRMS (ESI-TOF, positive) of **7a**.

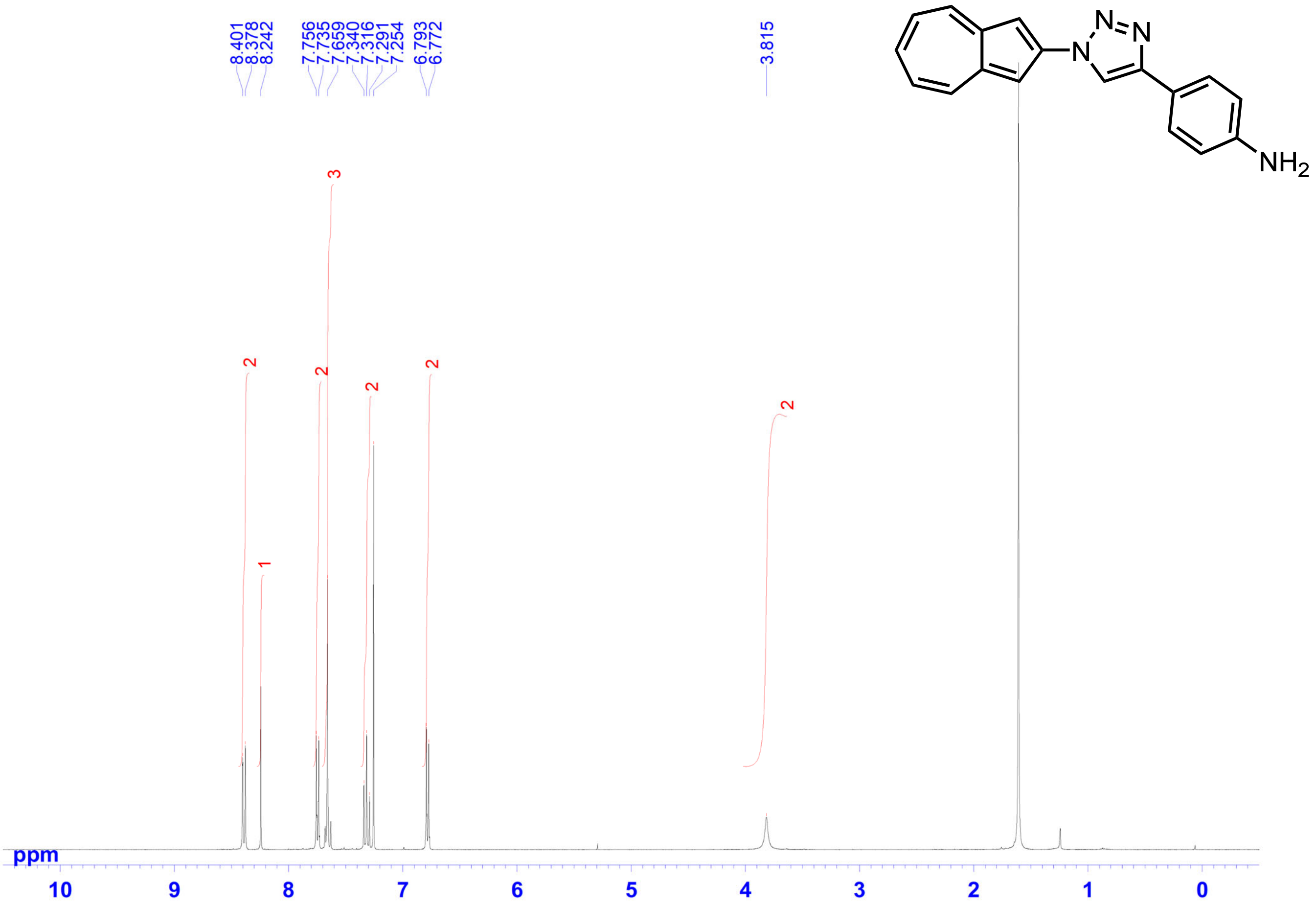

Figure S61. ^{1}H NMR spectrum of **7b** in $CDCl_3$ (400 MHz).

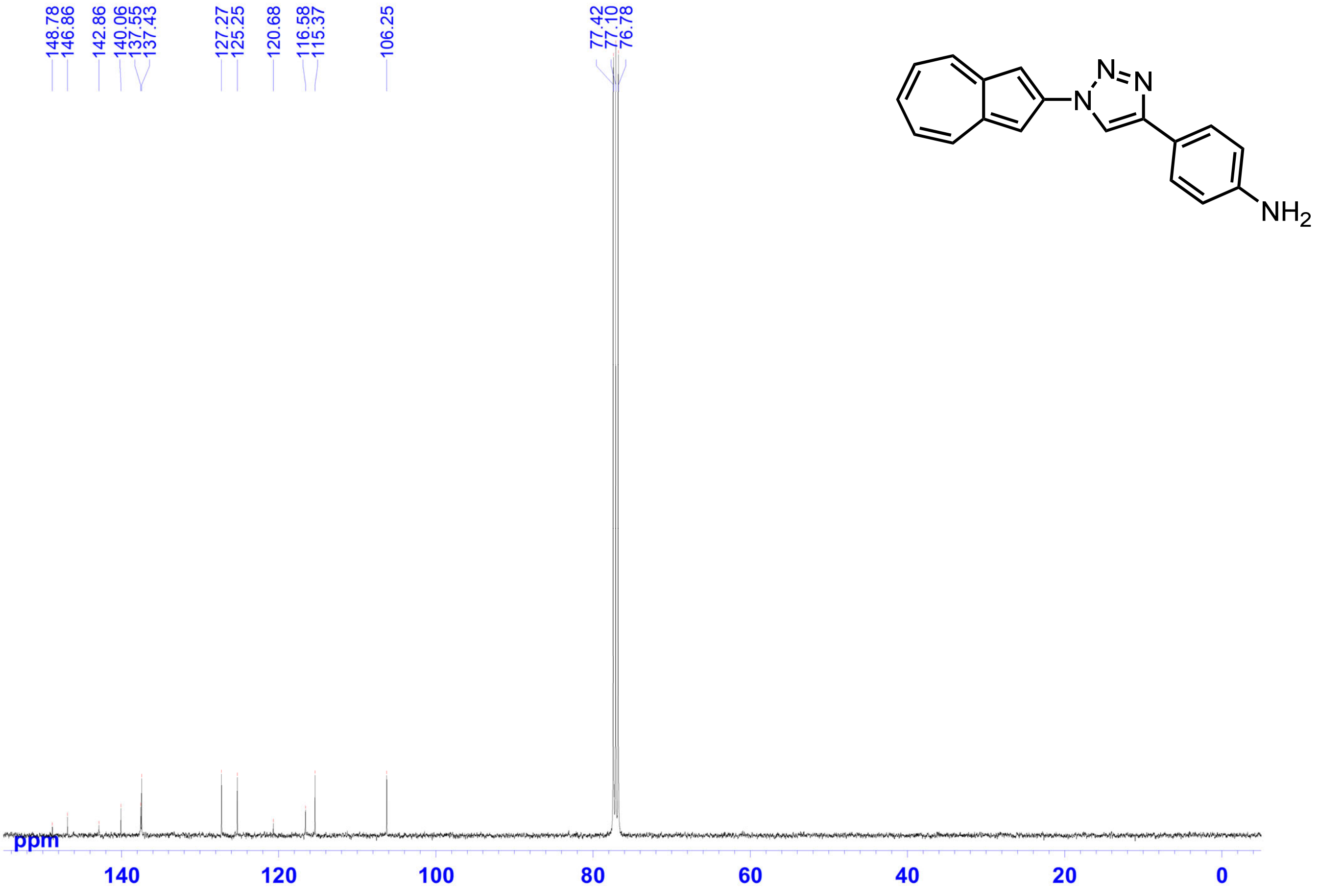

Figure S62. $^{13}C\{^{1}H\}$ NMR spectrum of **7b** in $CDCl_3$ (100 MHz).

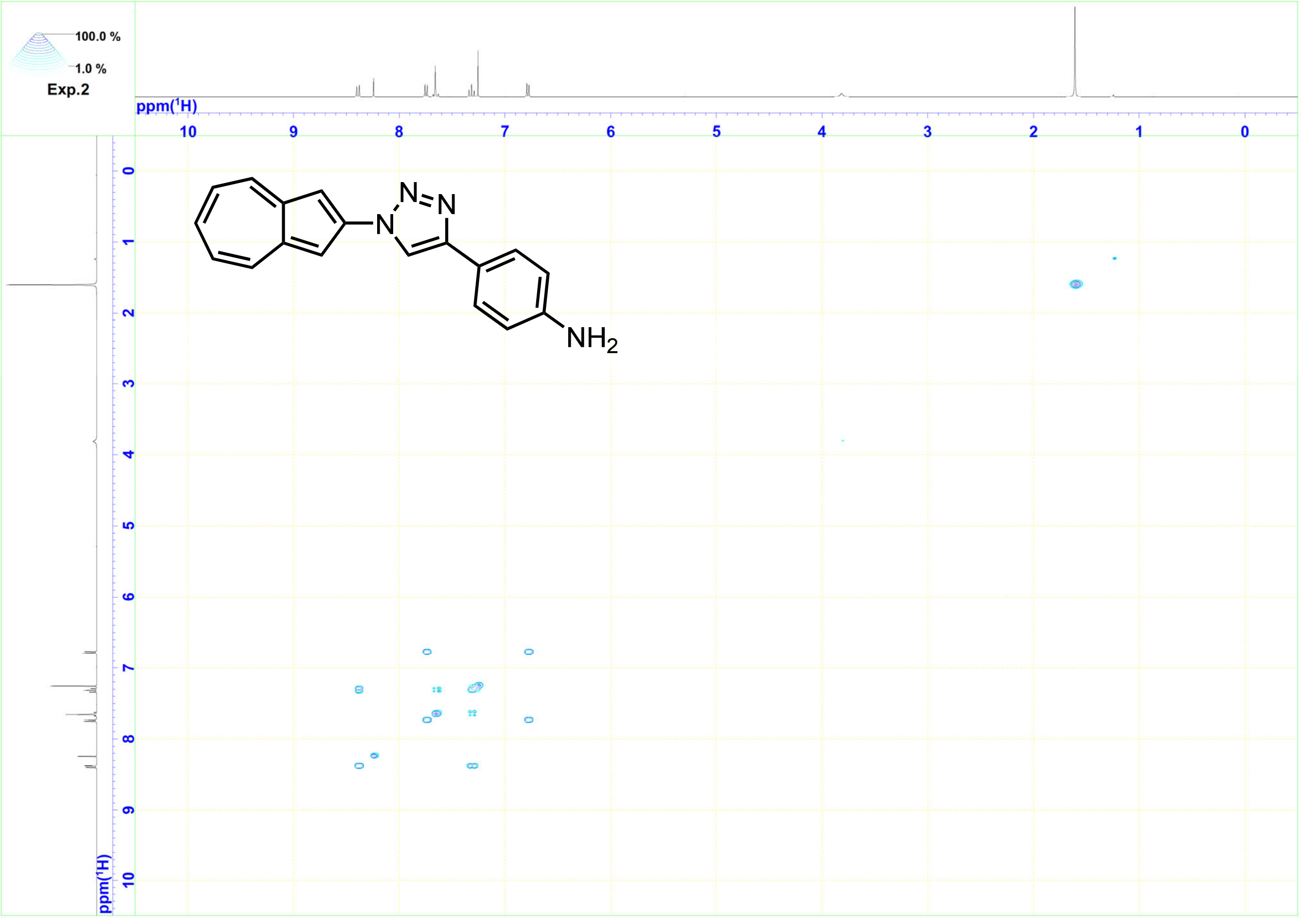

Figure S63. COSY spectrum of **7b** in $CDCl_3$ (400 MHz).

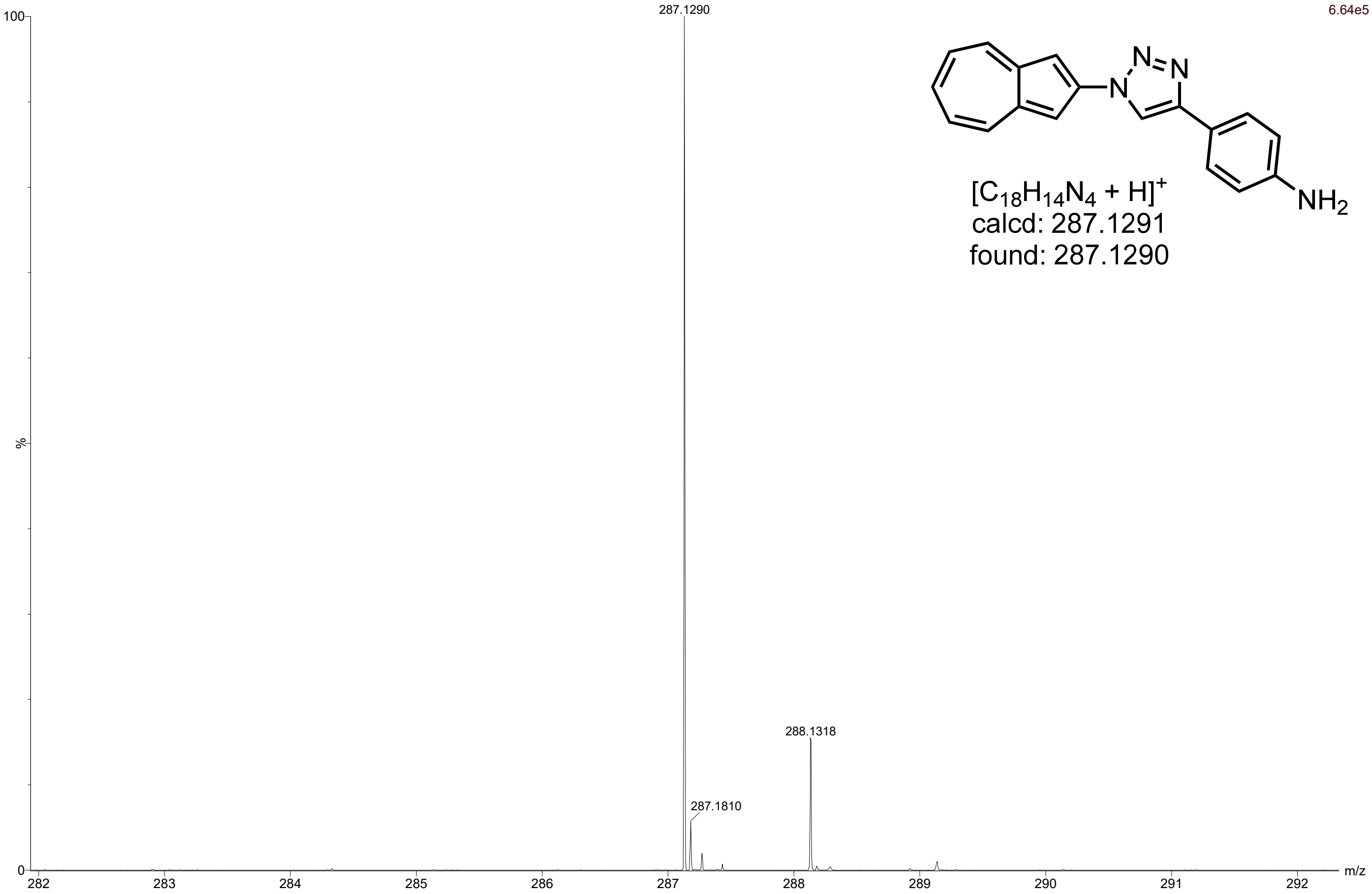

Figure S64. HRMS (ESI-TOF, positive) of **7b**.

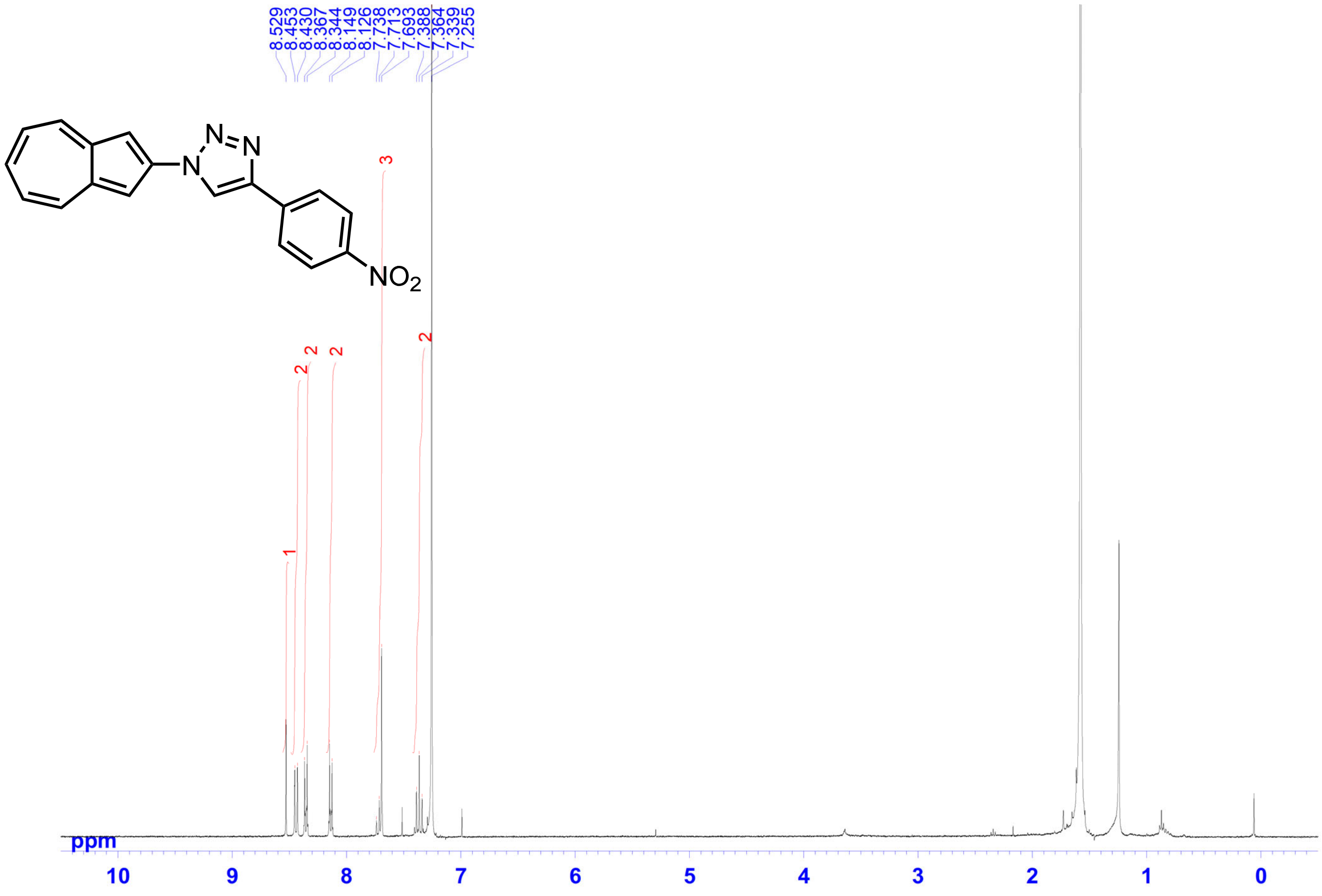

Figure S65. ¹H NMR spectrum of **7c** in $CDCl_3$ (400 MHz).

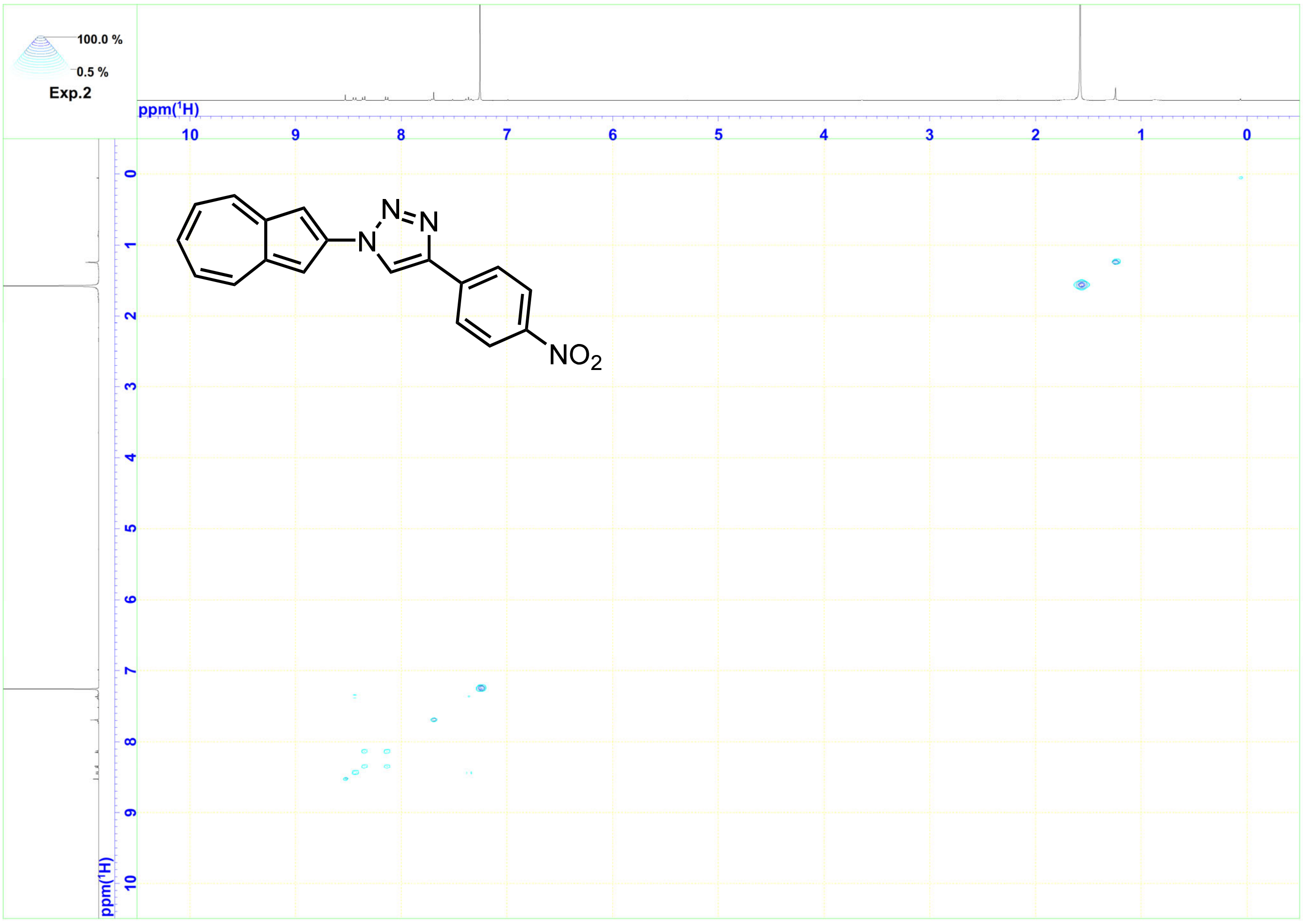

Figure S66. COSY spectrum of **7c** in $CDCl_3$ (400 MHz).

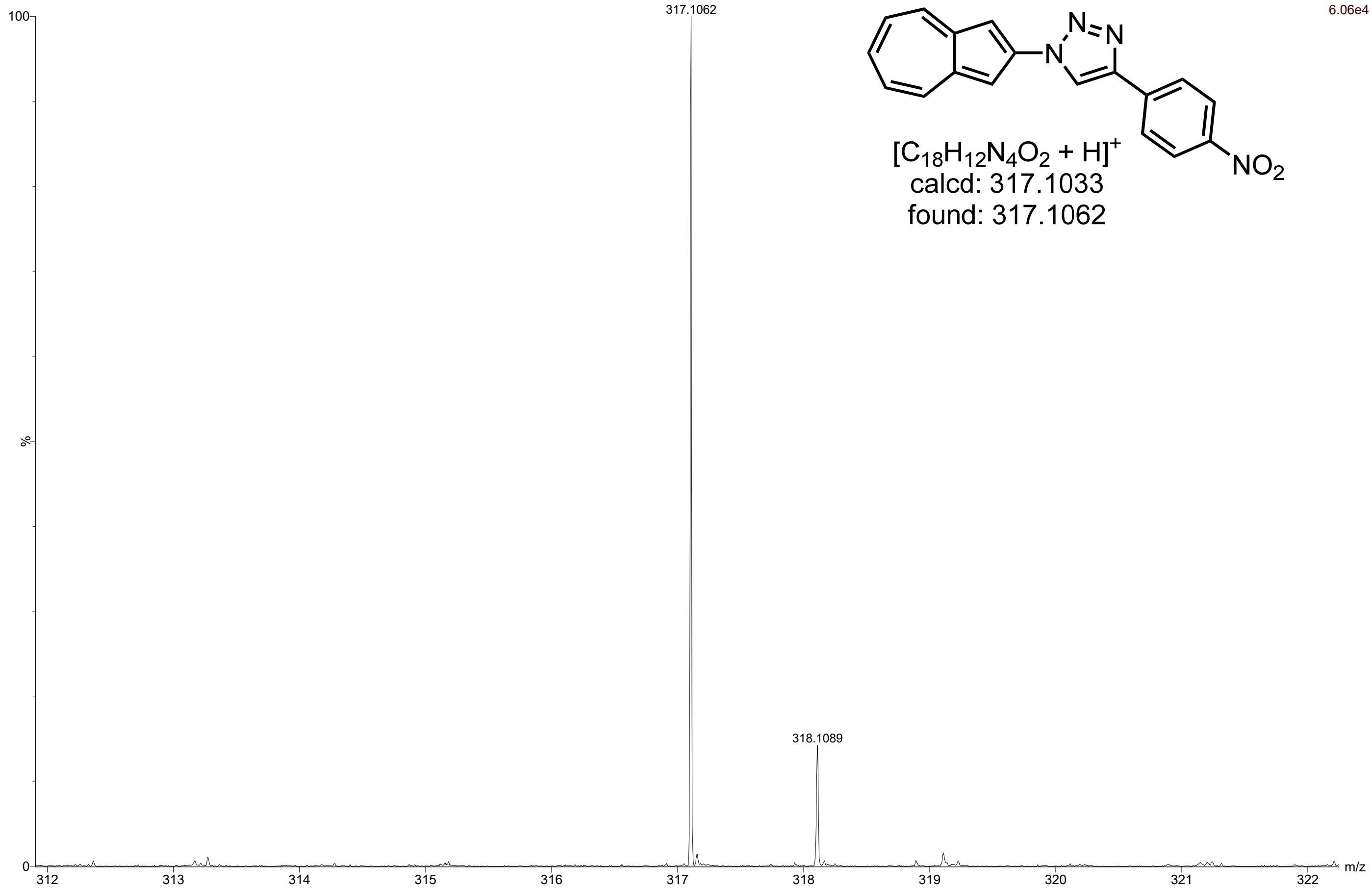

Figure S67. HRMS (ESI-TOF, positive) of **7c**.

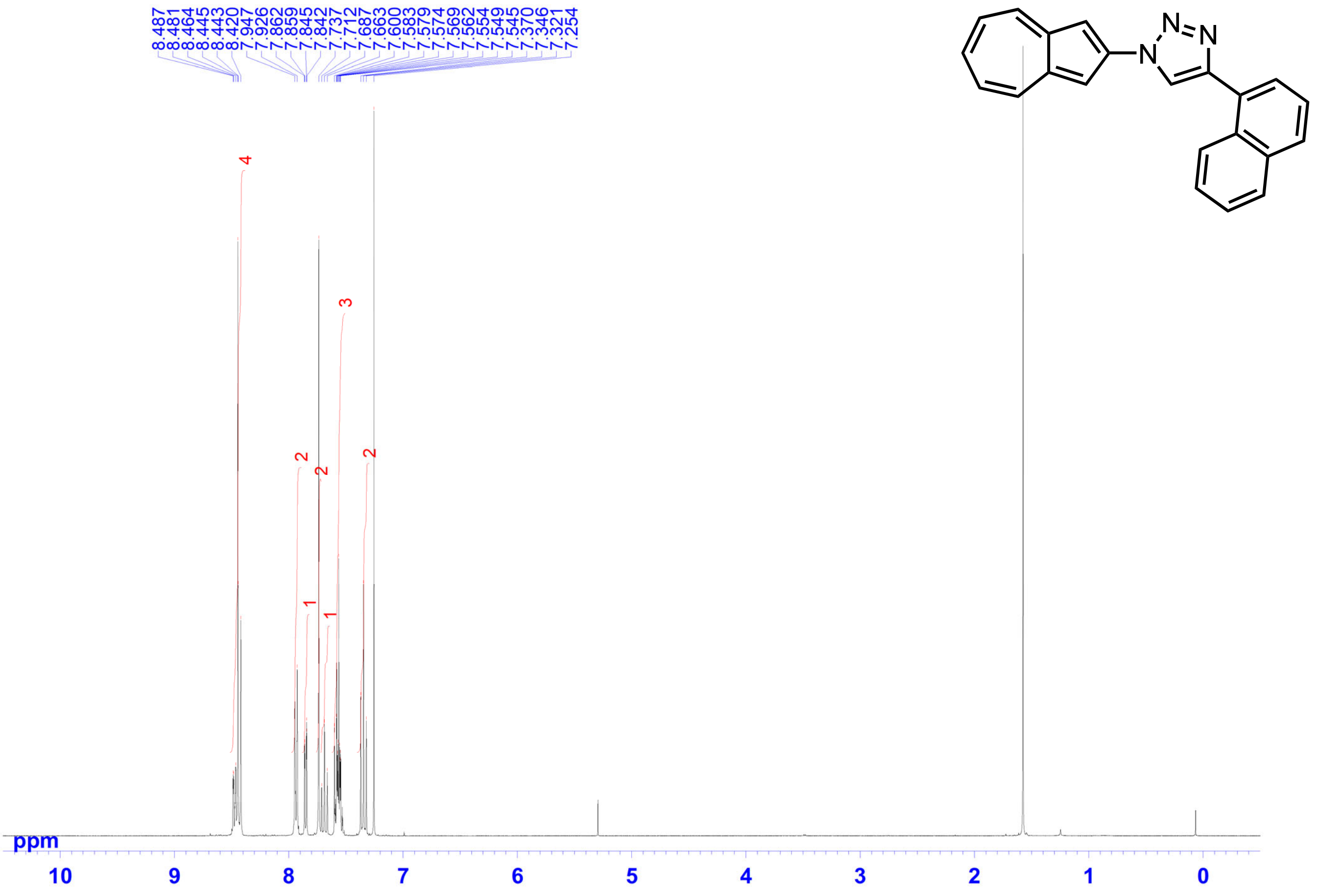

Figure S68. ^{1}H NMR spectrum of **7d** in $CDCl_3$ (400 MHz).

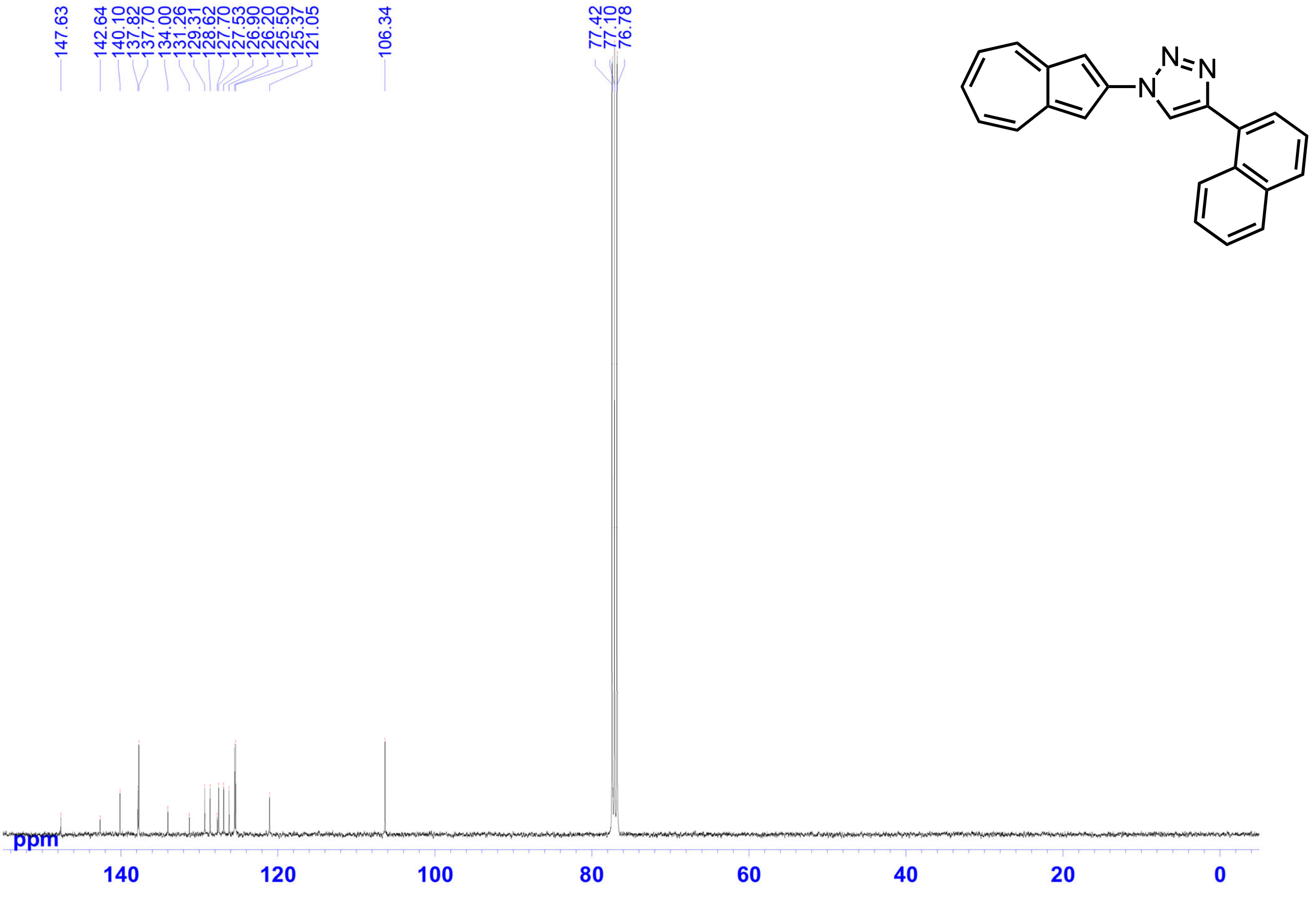

Figure S69. ^{13}C{^{1}H} NMR spectrum of **7d** in $CDCl_3$ (100 MHz).

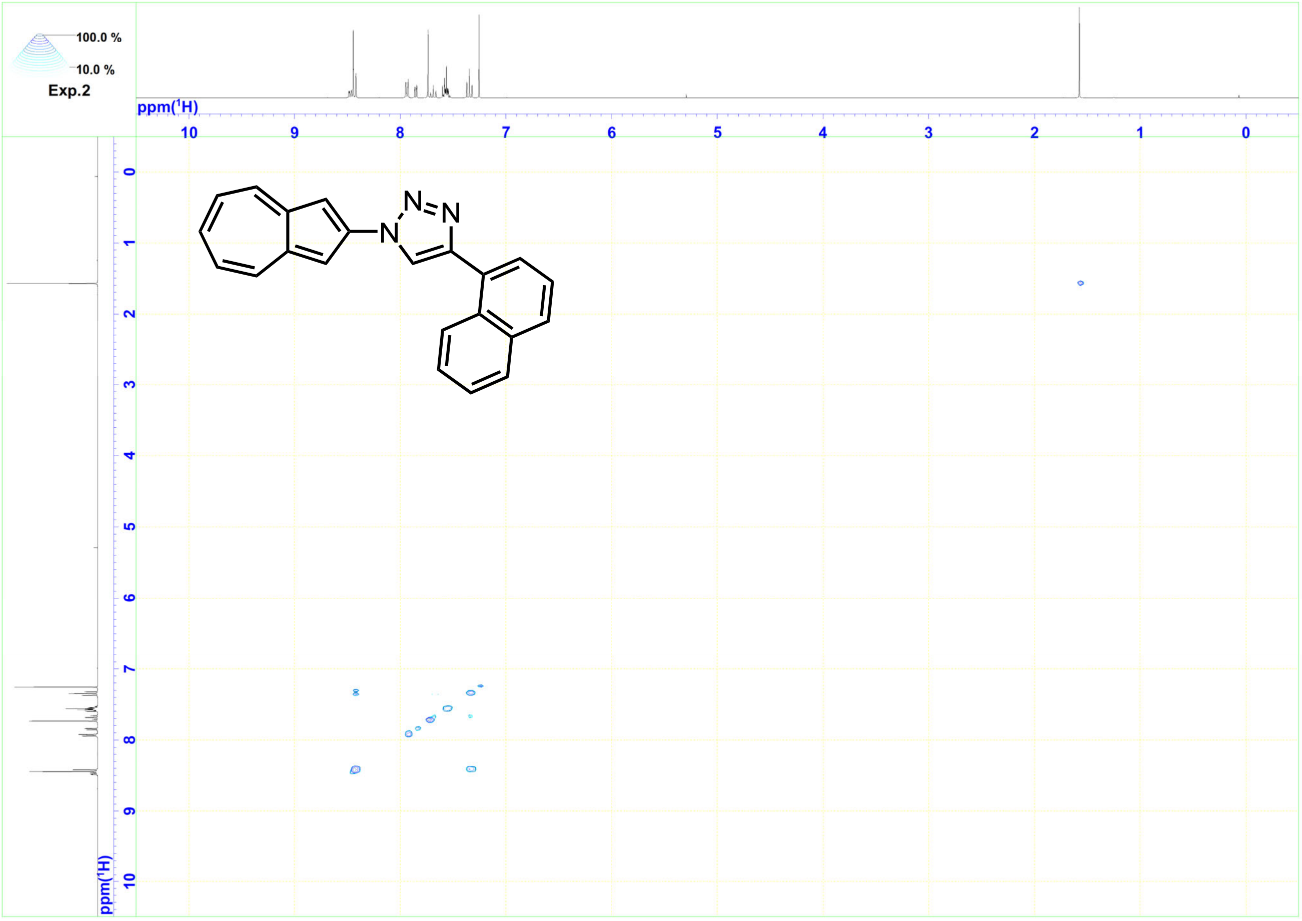

Figure S70. COSY spectrum of **7d** in $CDCl_3$ (400 MHz).

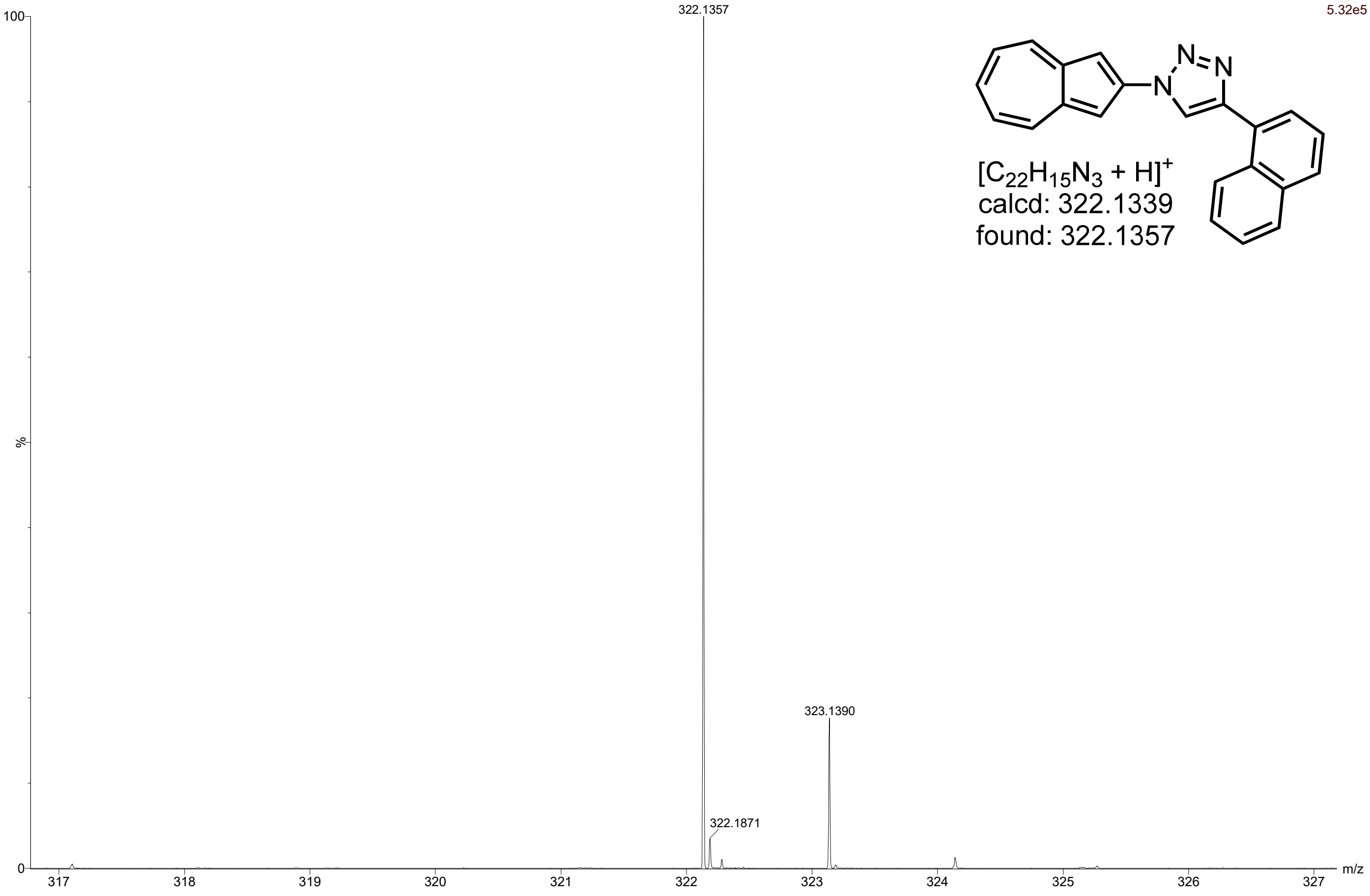

Figure S71. HRMS (ESI-TOF, positive) of **7d**.

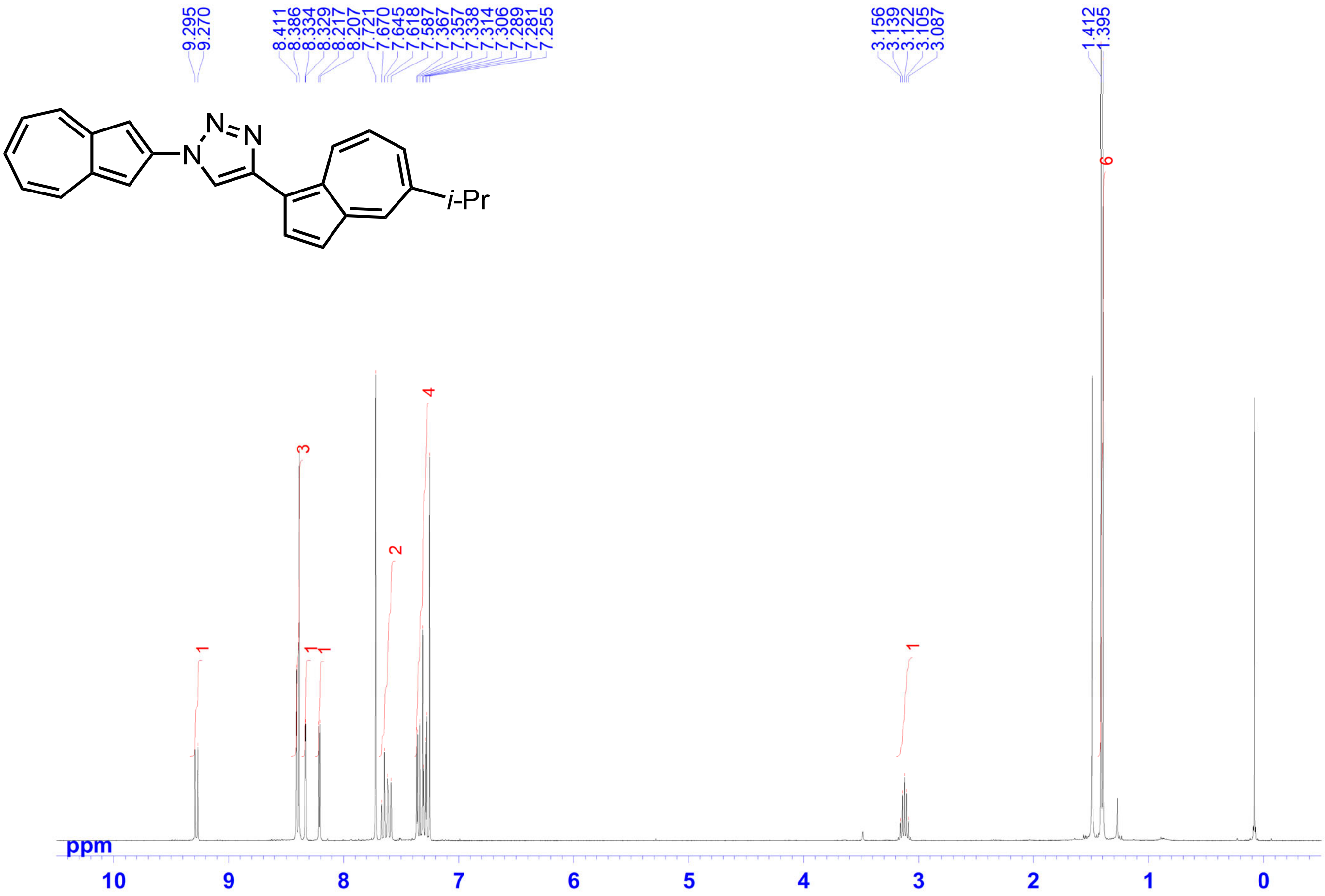

Figure S72. ^{1}H NMR spectrum of **7e** in $CDCl_3$ (400 MHz).

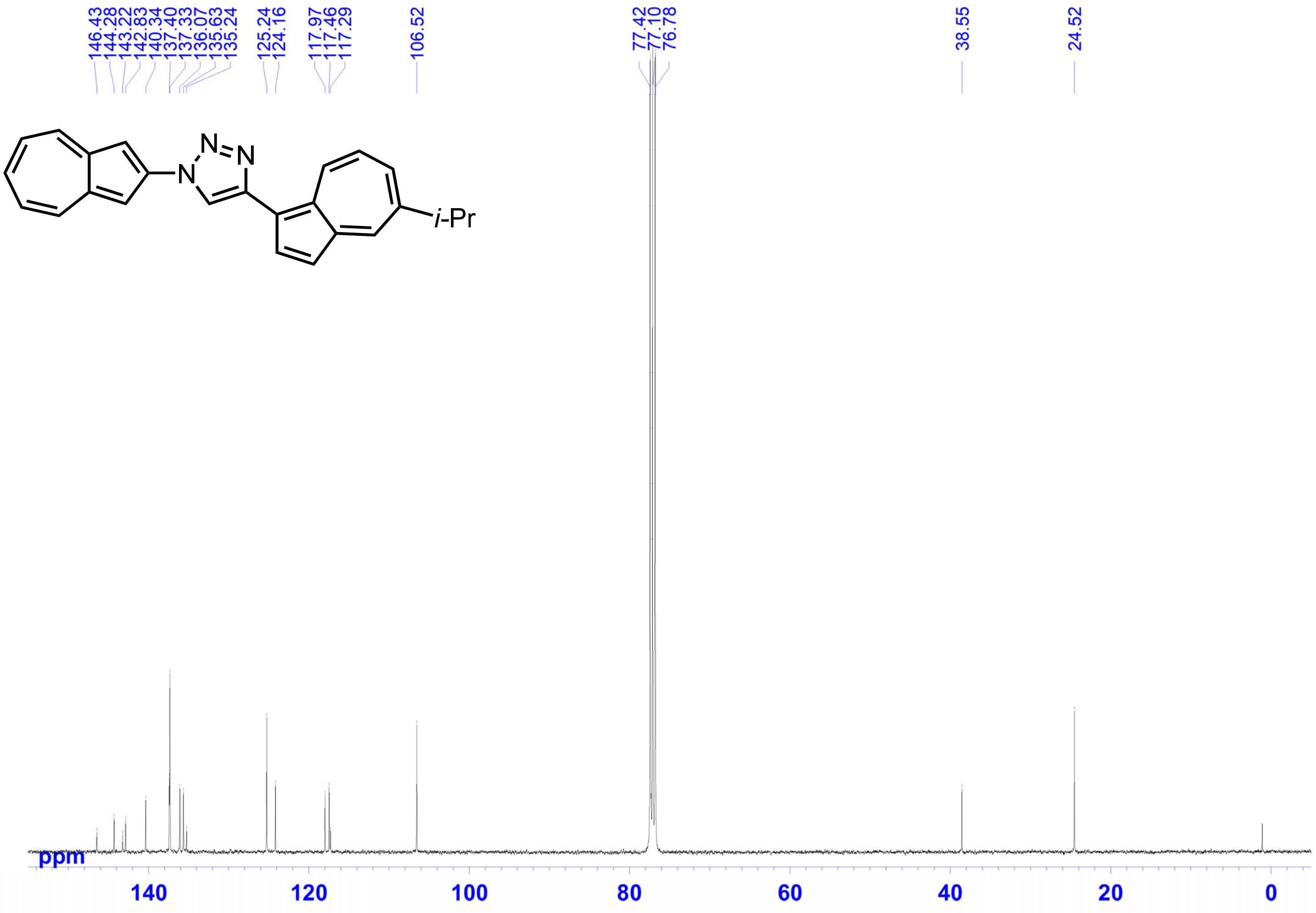

Figure S73. $^{13}C\{^1H\}$ NMR spectrum of **7e** in $CDCl_3$ (100 MHz).

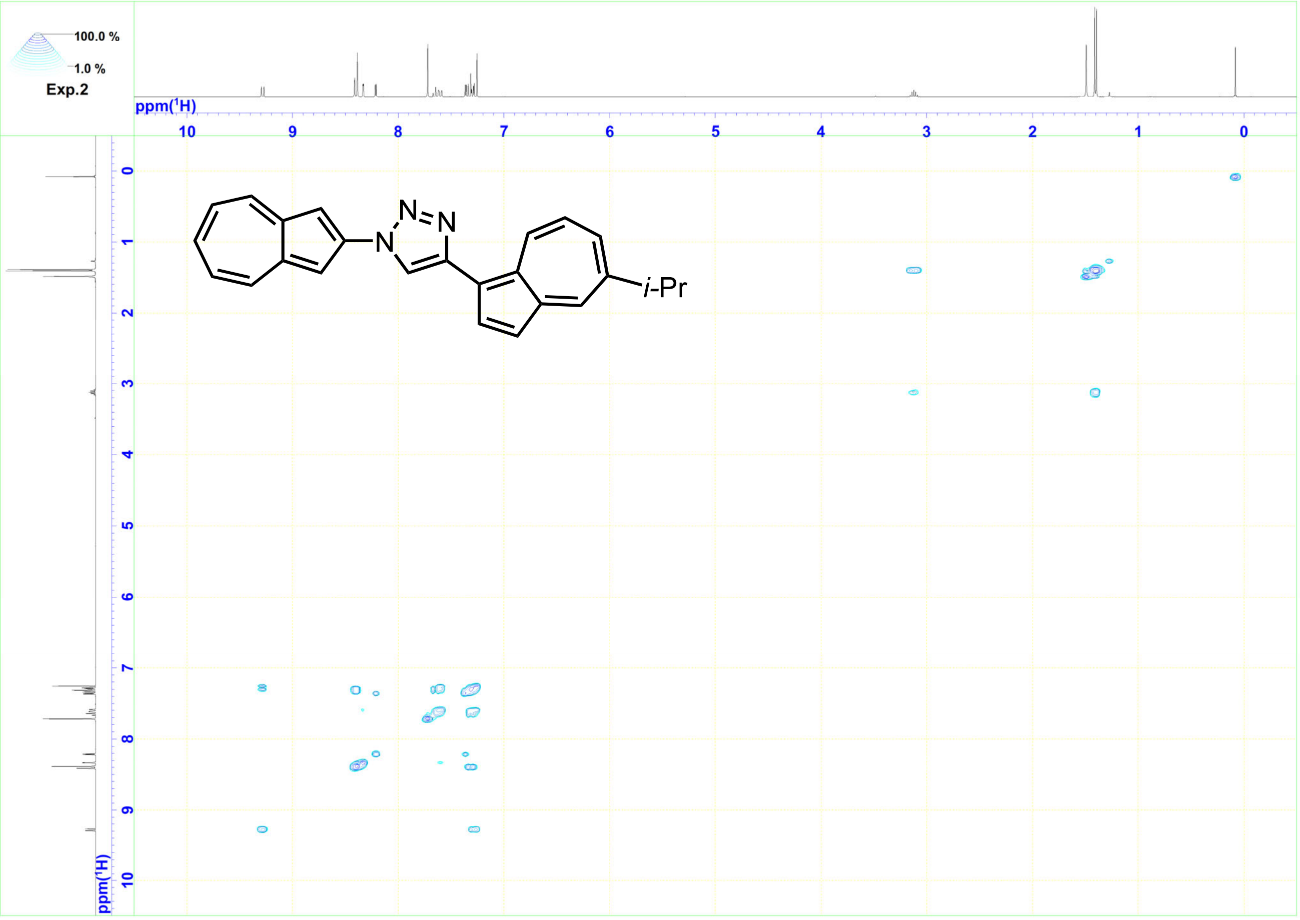

Figure S74. COSY spectrum of **7e** in $CDCl_3$ (400 MHz).

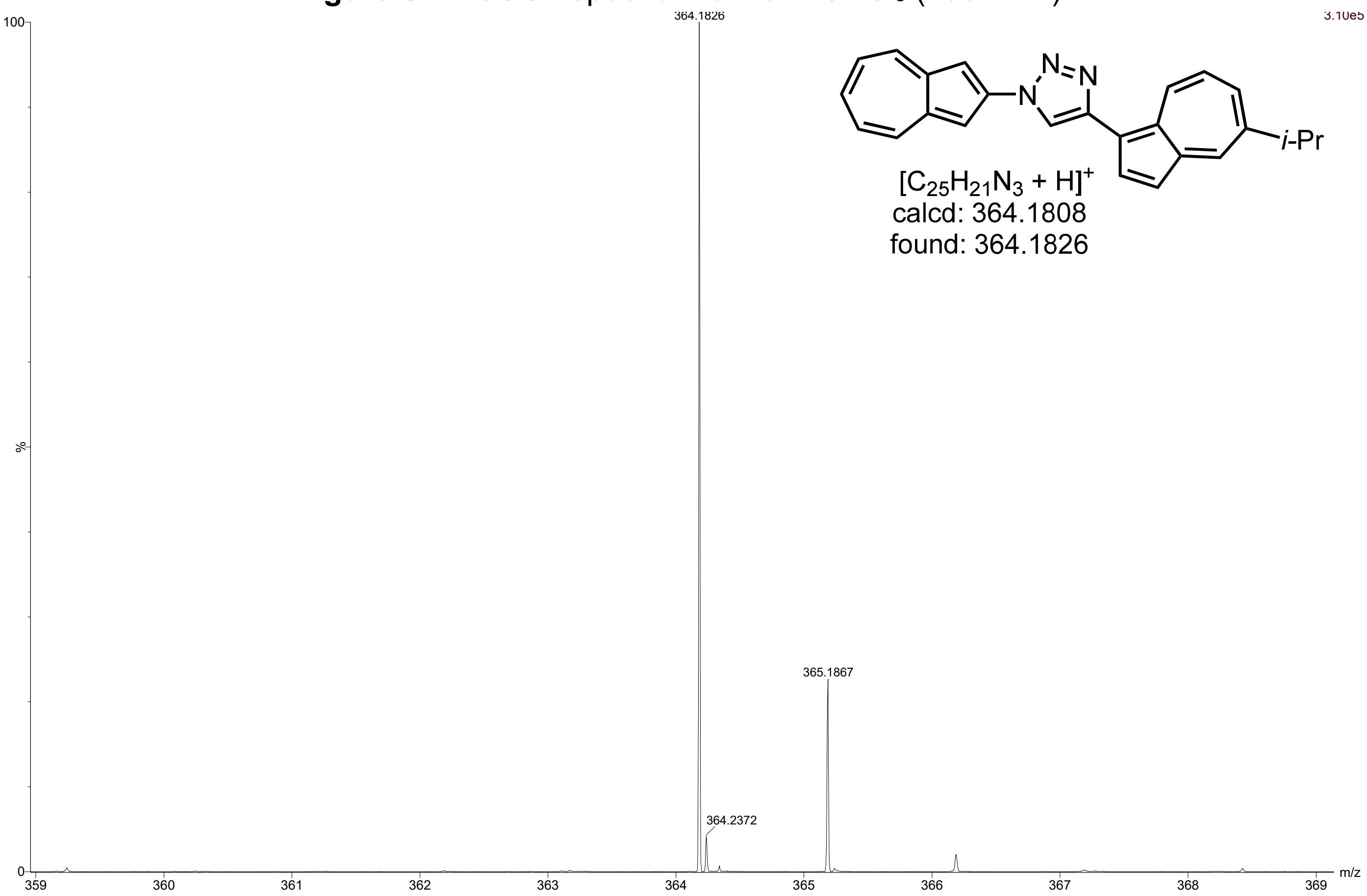

Figure S75. HRMS (ESI-TOF, positive) of **7e**.

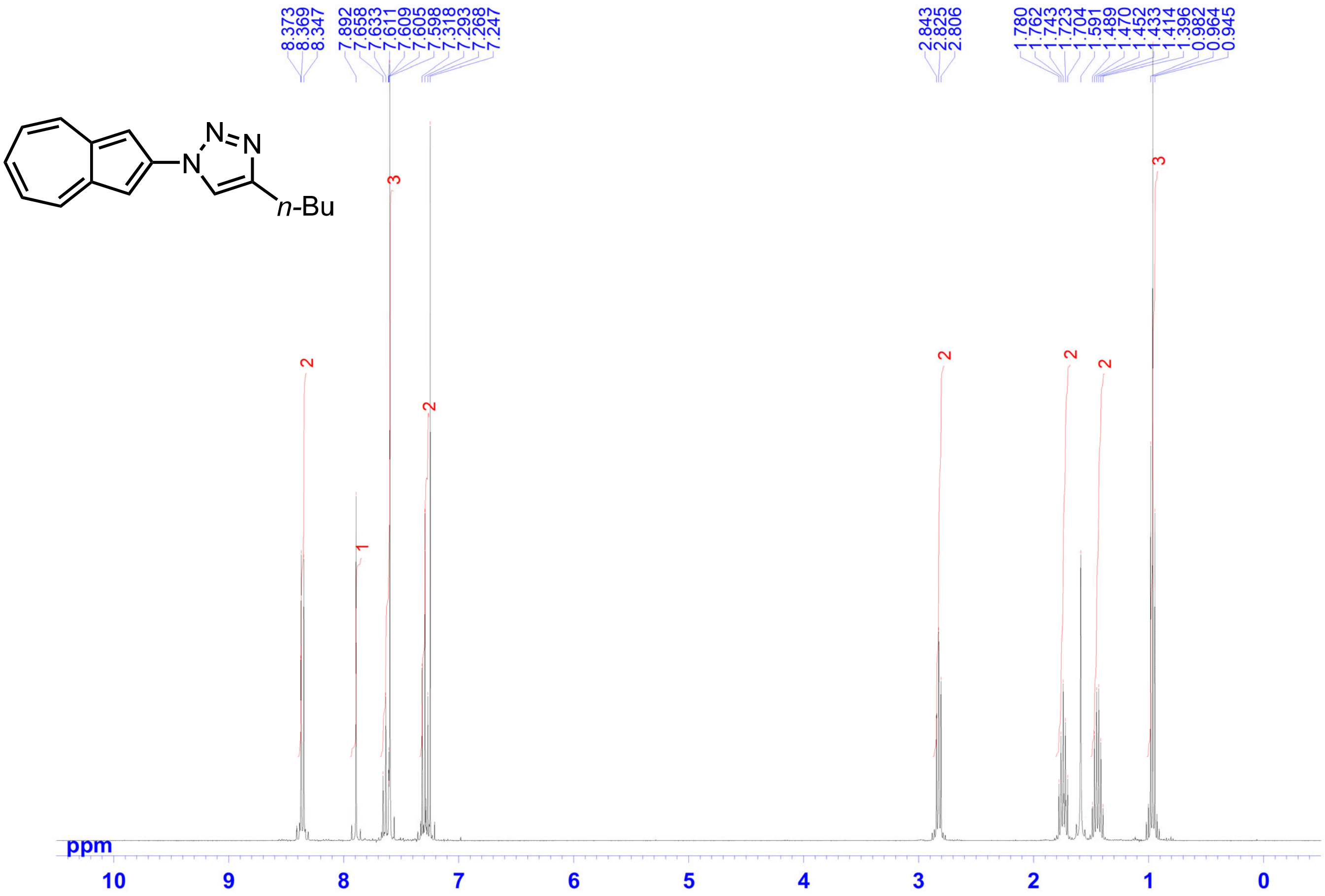

Figure S76. ^{1}H NMR spectrum of **7g** in $CDCl_3$ (400 MHz).

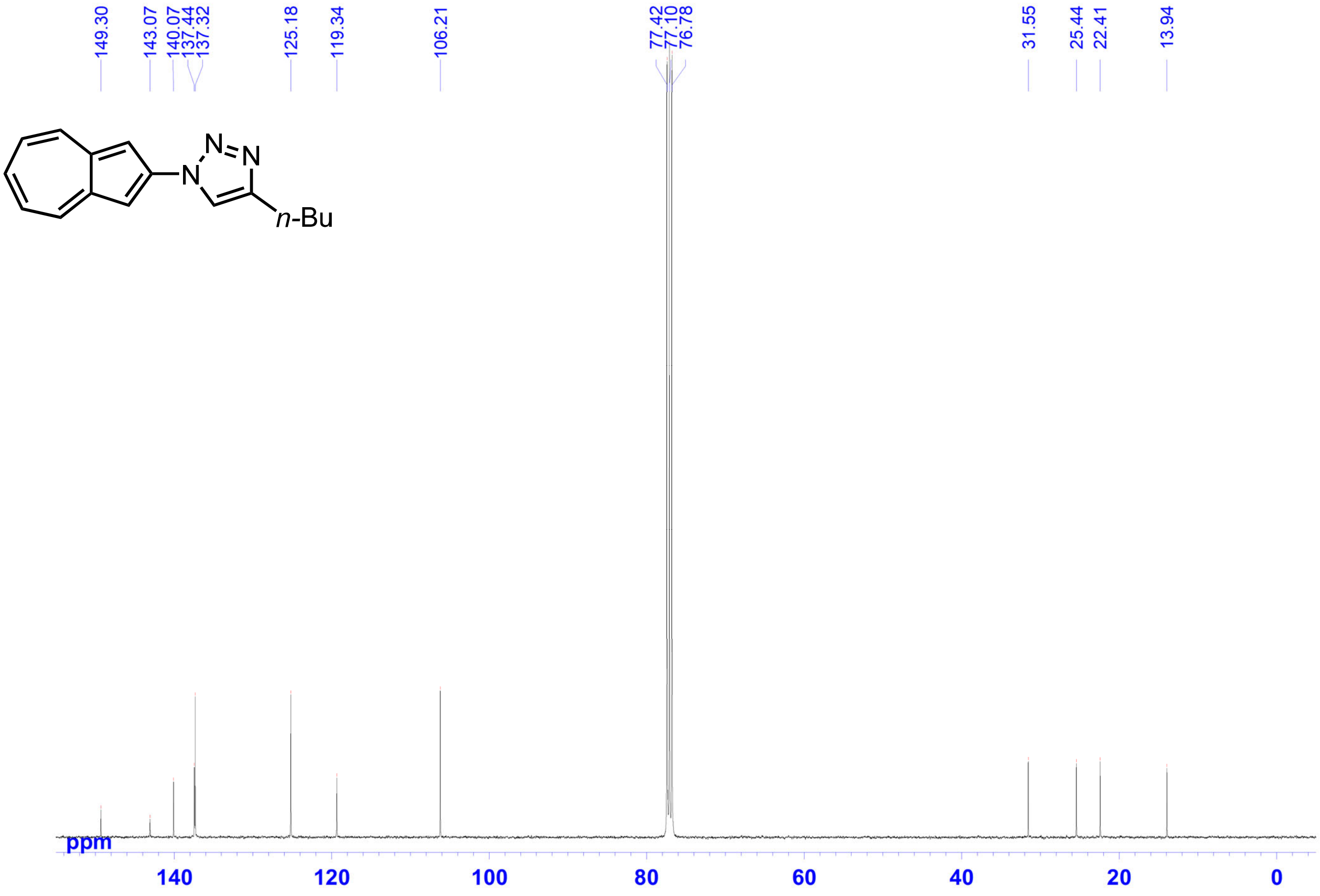

Figure S77. $^{13}C\{^{1}H\}$ NMR spectrum of **7g** in $CDCl_3$ (100 MHz).

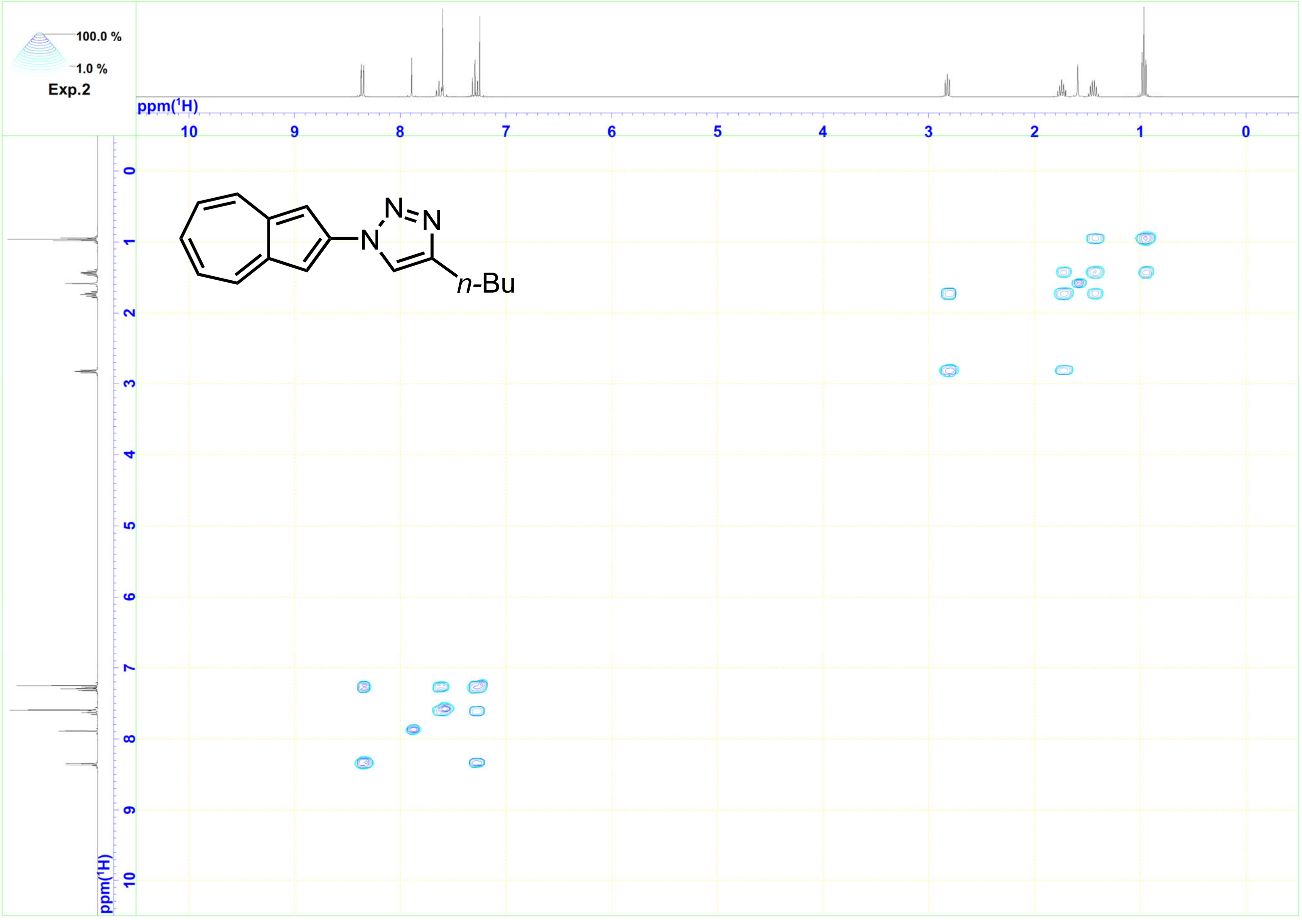

Figure S78. COSY spectrum of **7g** in $CDCl_3$ (400 MHz).

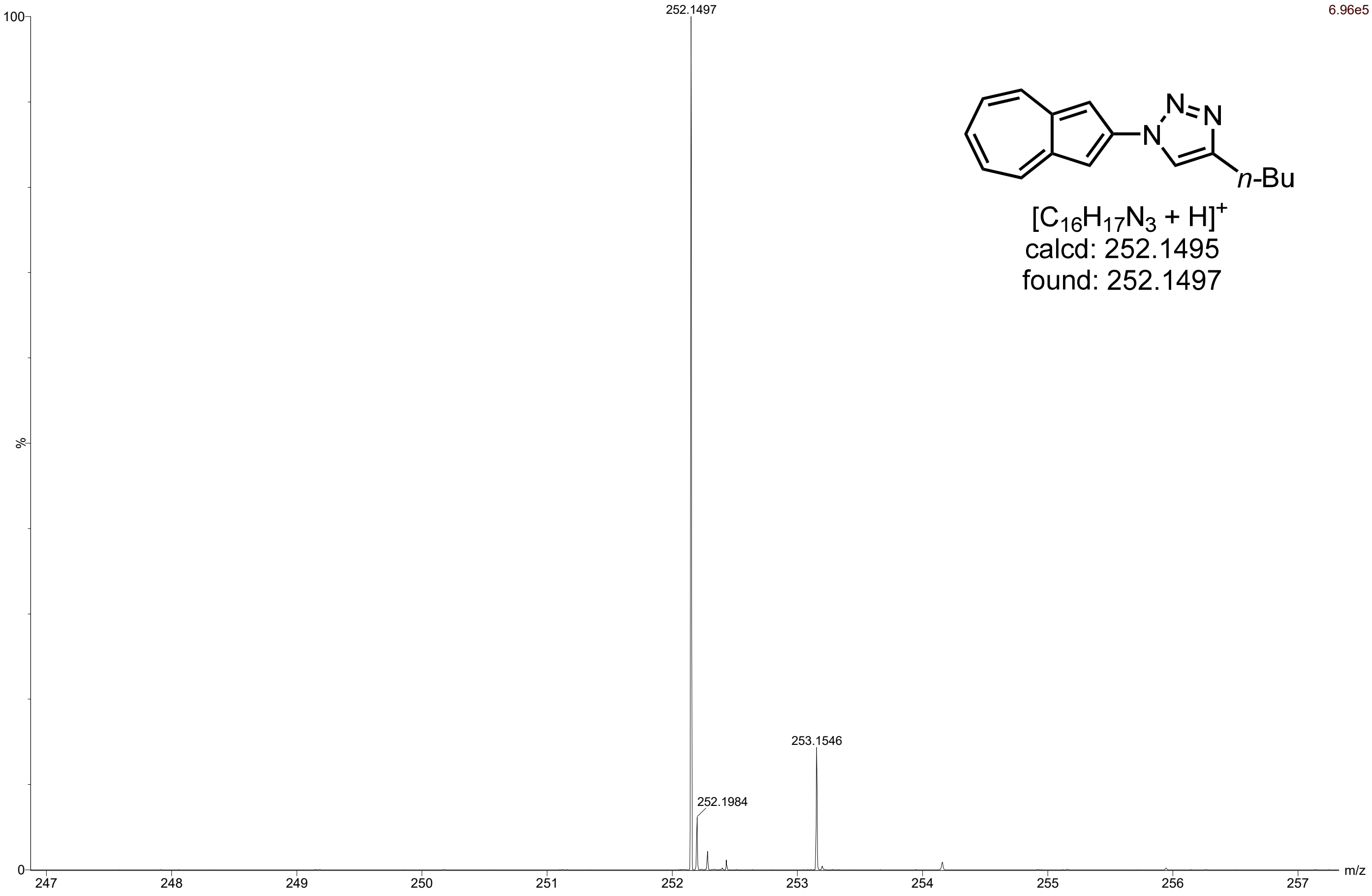

Figure S79. HRMS (ESI-TOF, positive) of **7g**.

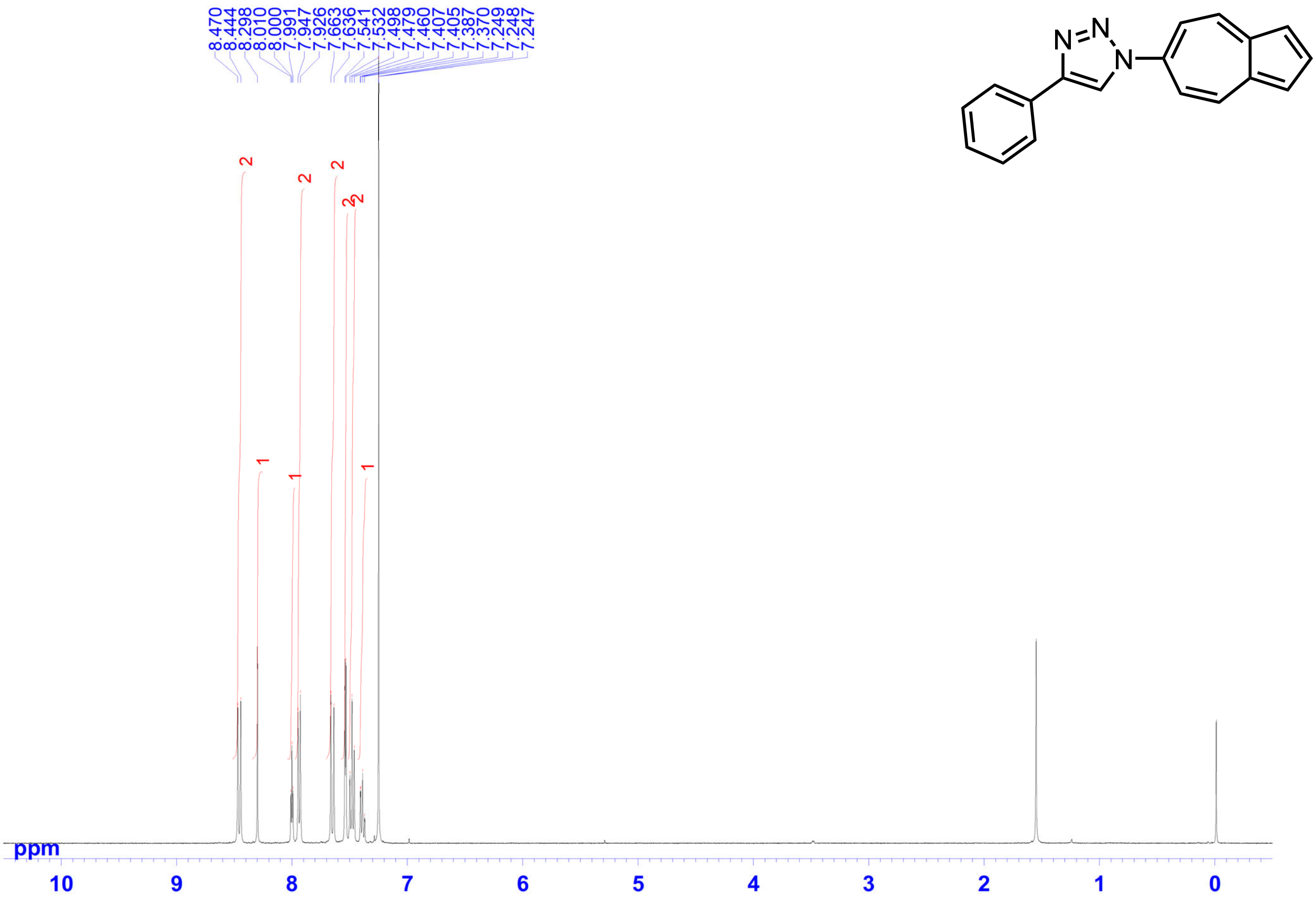

Figure S80. ^{1}H NMR spectrum of **8** in $CDCl_3$ (400 MHz).

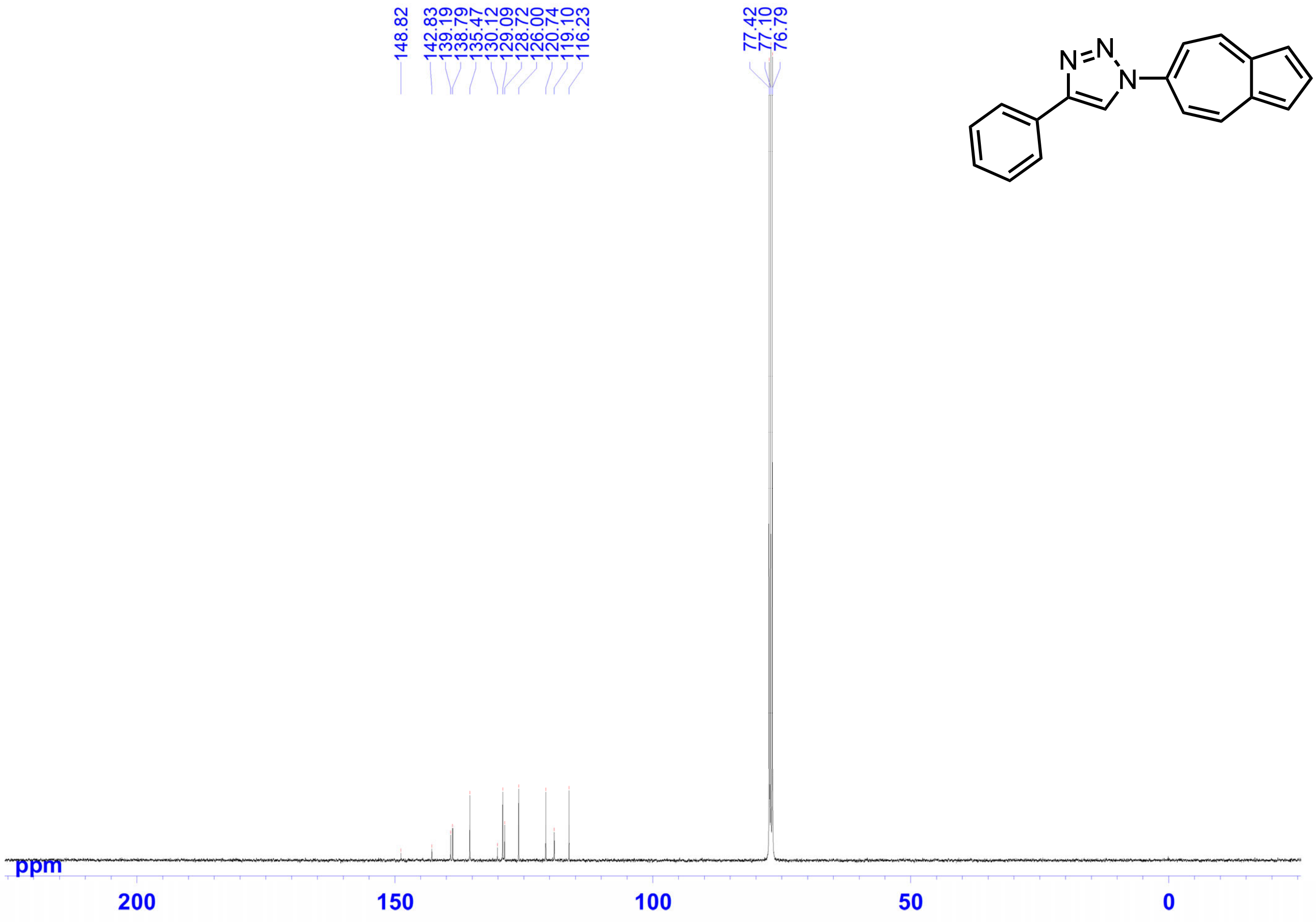

Figure S81. $^{13}C\{^{1}H\}$ NMR spectrum of **8** in $CDCl_3$ (100 MHz).

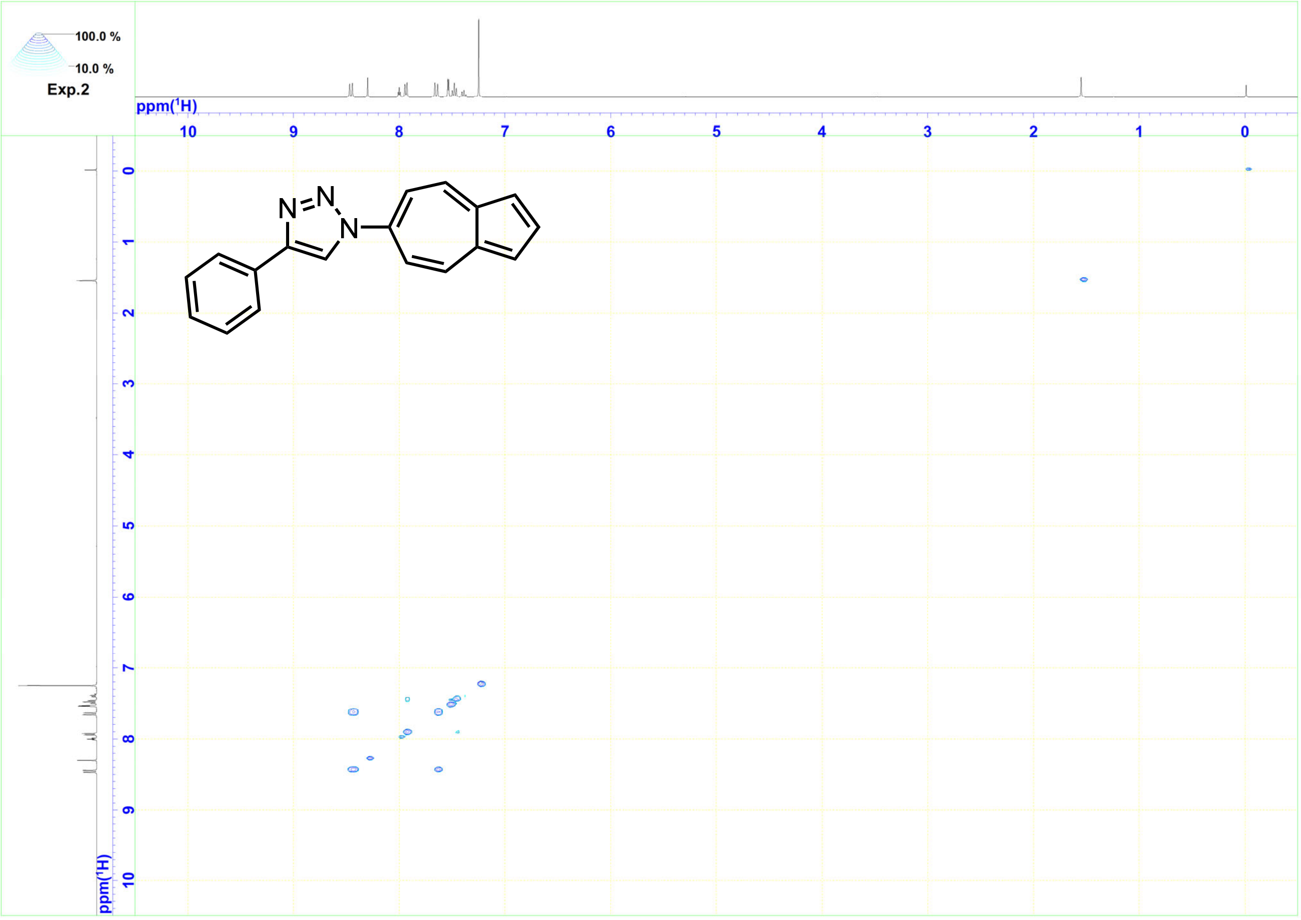

Figure S82. COSY spectrum of **8** in CDCl3 (400 MHz).

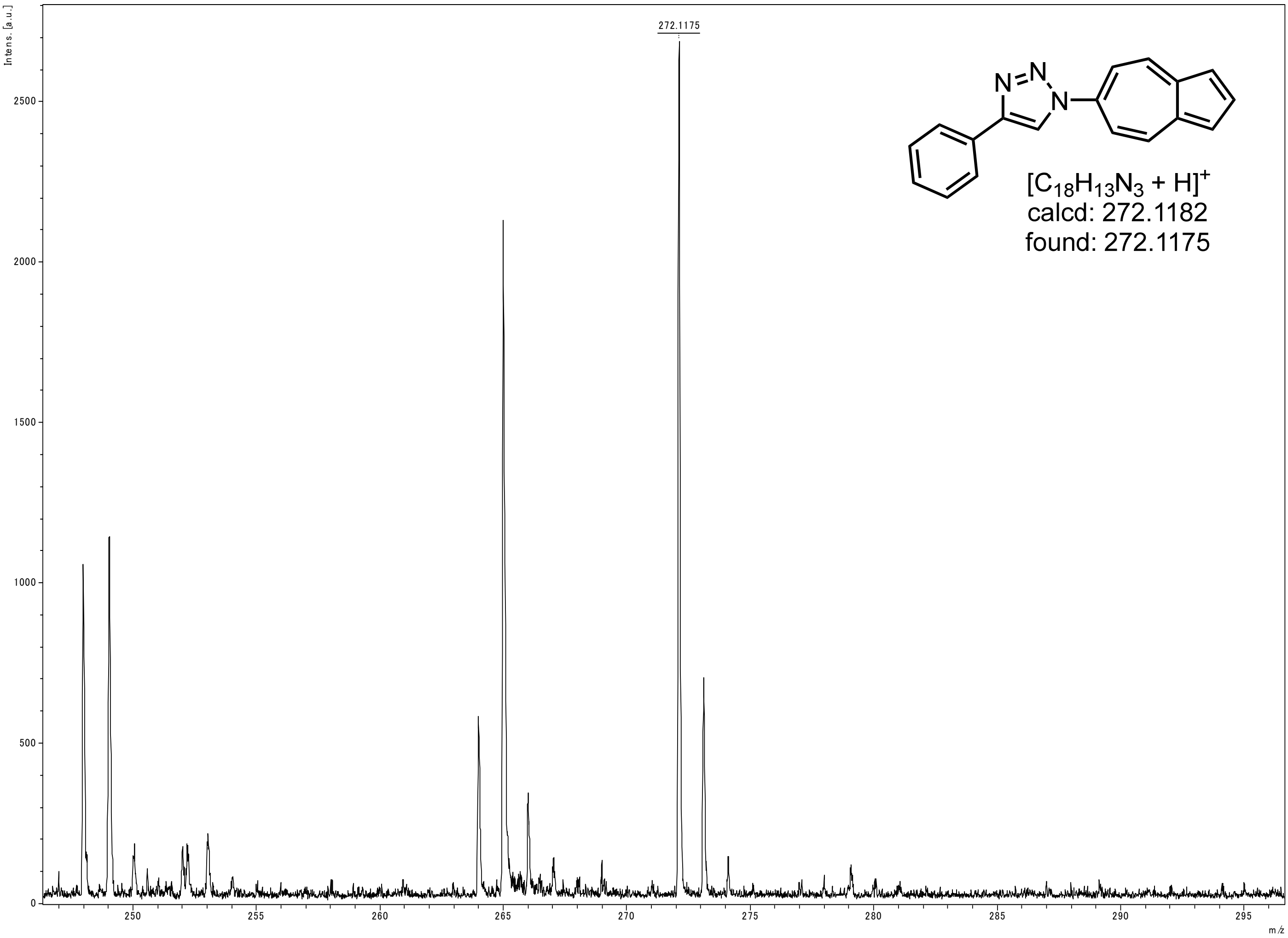

Figure S83. HRMS (ESI-TOF, positive) of **8**.

2. UV/Vis and fluorescent spectra of reported compounds

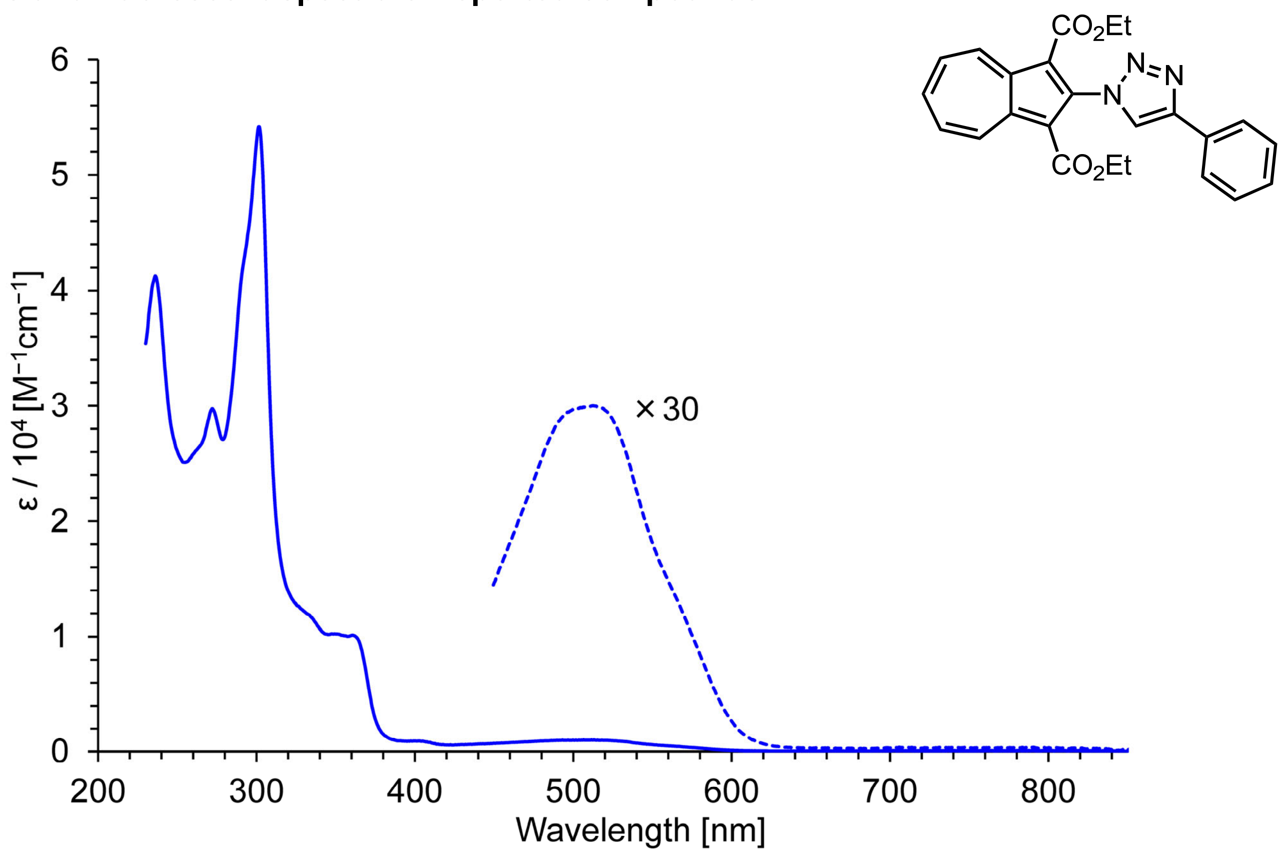

Figure S84. UV/Vis spectra of **5a** (3.29×10^{-5} M) in CH_2Cl_2. The spectrum in the visible region is magnified × 30 (dotted lines).

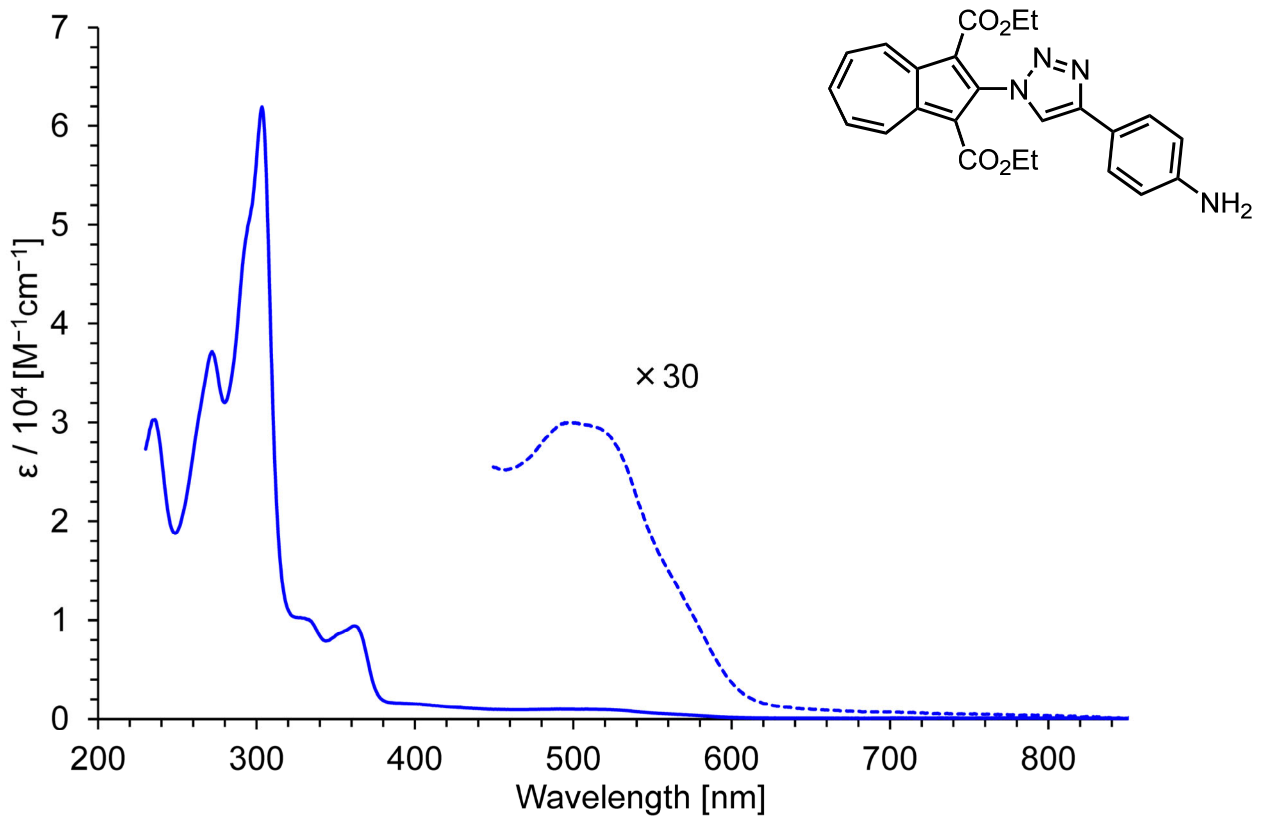

Figure S85. UV/Vis spectra of **5b** (2.30×10^{-5} M) in CH_2Cl_2. The spectrum in the visible region is magnified × 30 (dotted lines).

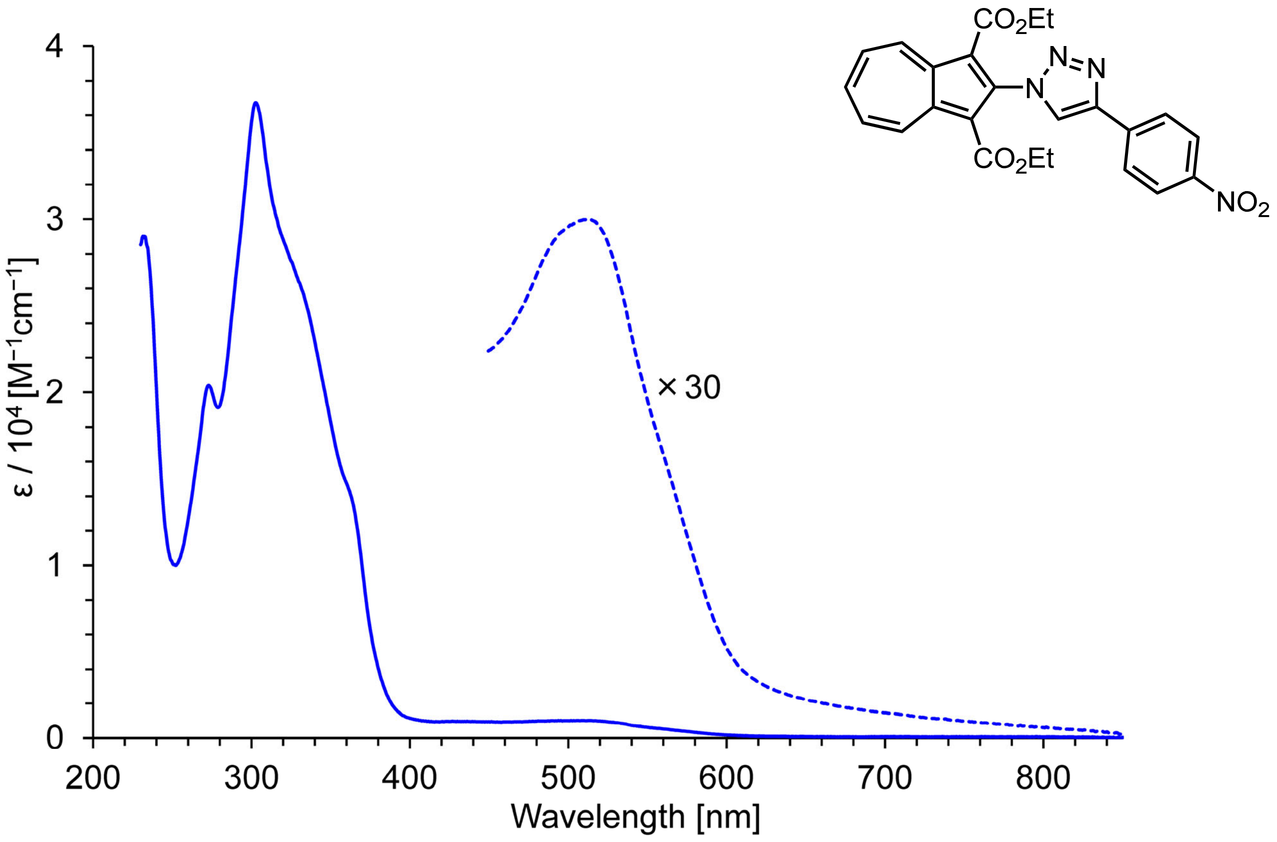

Figure S86. UV/Vis spectra of **5c** (2.73×10^{-5} M) in CH_2Cl_2. The spectrum in the visible region is magnified × 30 (dotted lines).

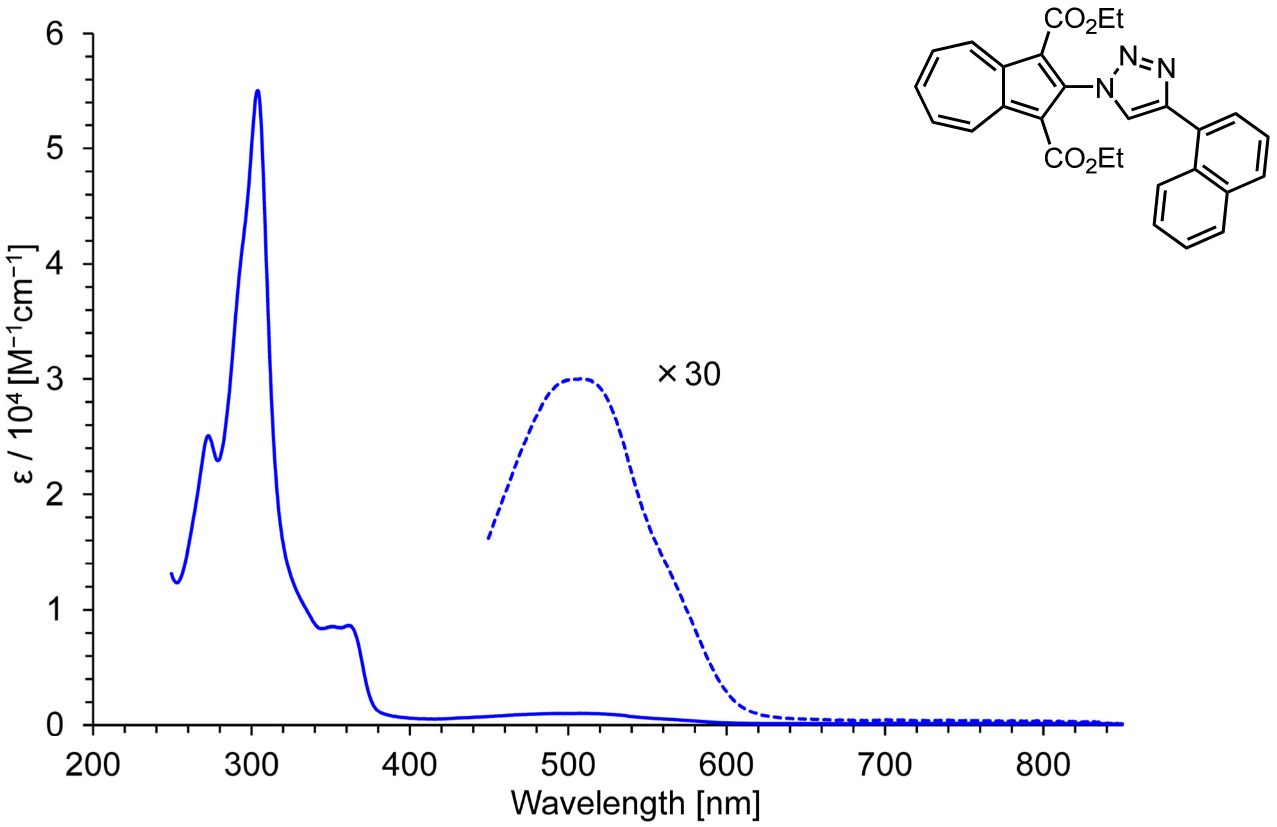

Figure S87. UV/Vis spectra of **5d** (2.71×10^{-5} M) in CH_2Cl_2. The spectrum in the visible region is magnified × 30 (dotted lines).

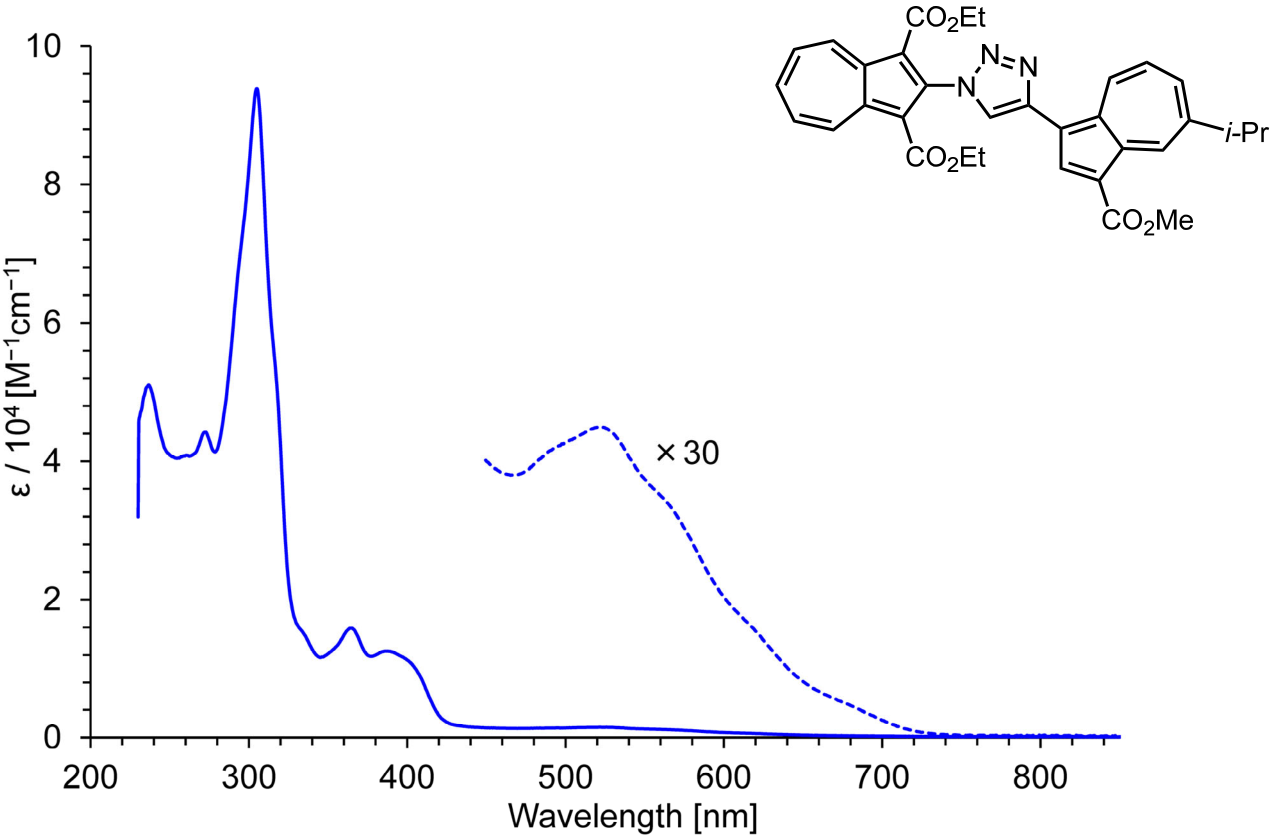

Figure S88. UV/Vis spectra of **5e** (2.41×10^{-5} M) in CH_2Cl_2. The spectrum in the visible region is magnified × 30 (dotted lines).

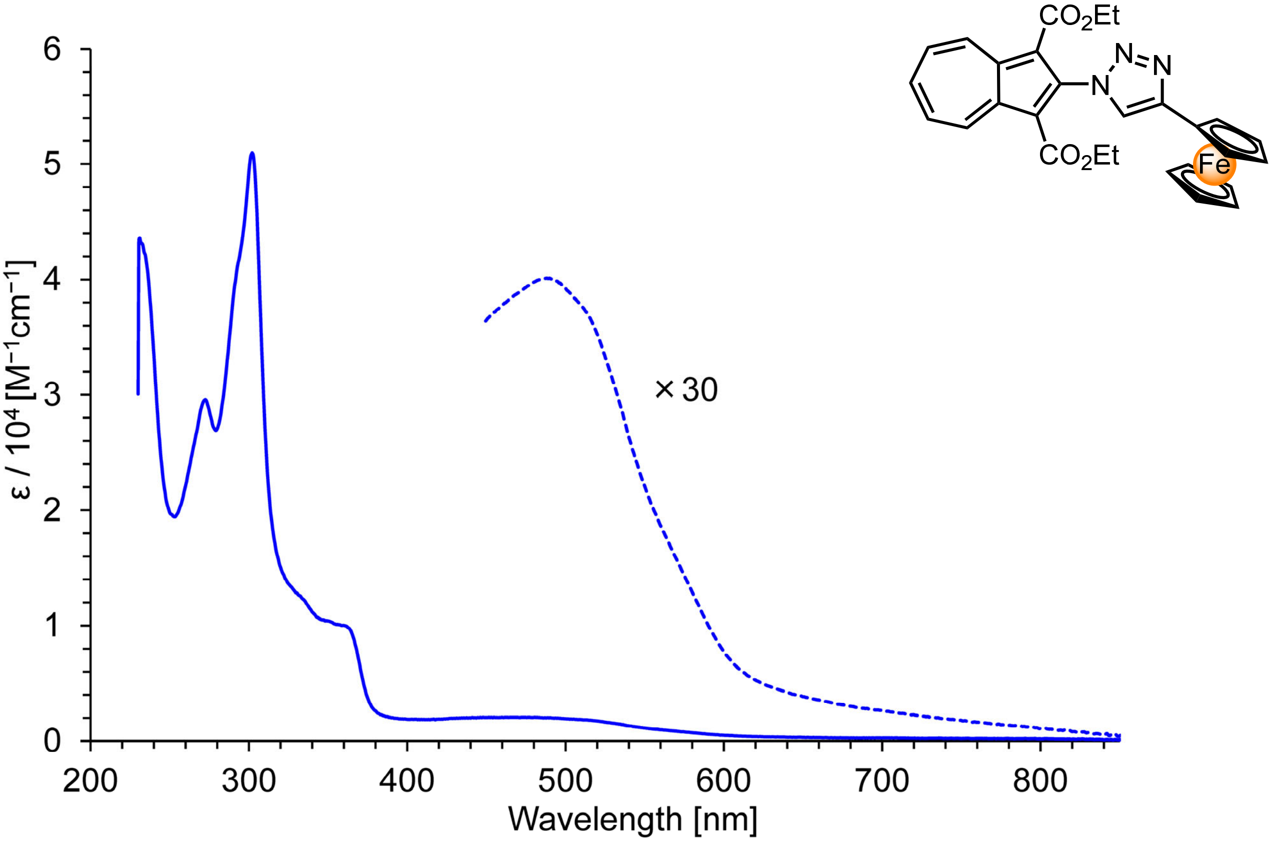

Figure S89. UV/Vis spectra of **5f** (2.55×10^{-5} M) in CH_2Cl_2. The spectrum in the visible region is magnified × 30 (dotted lines).

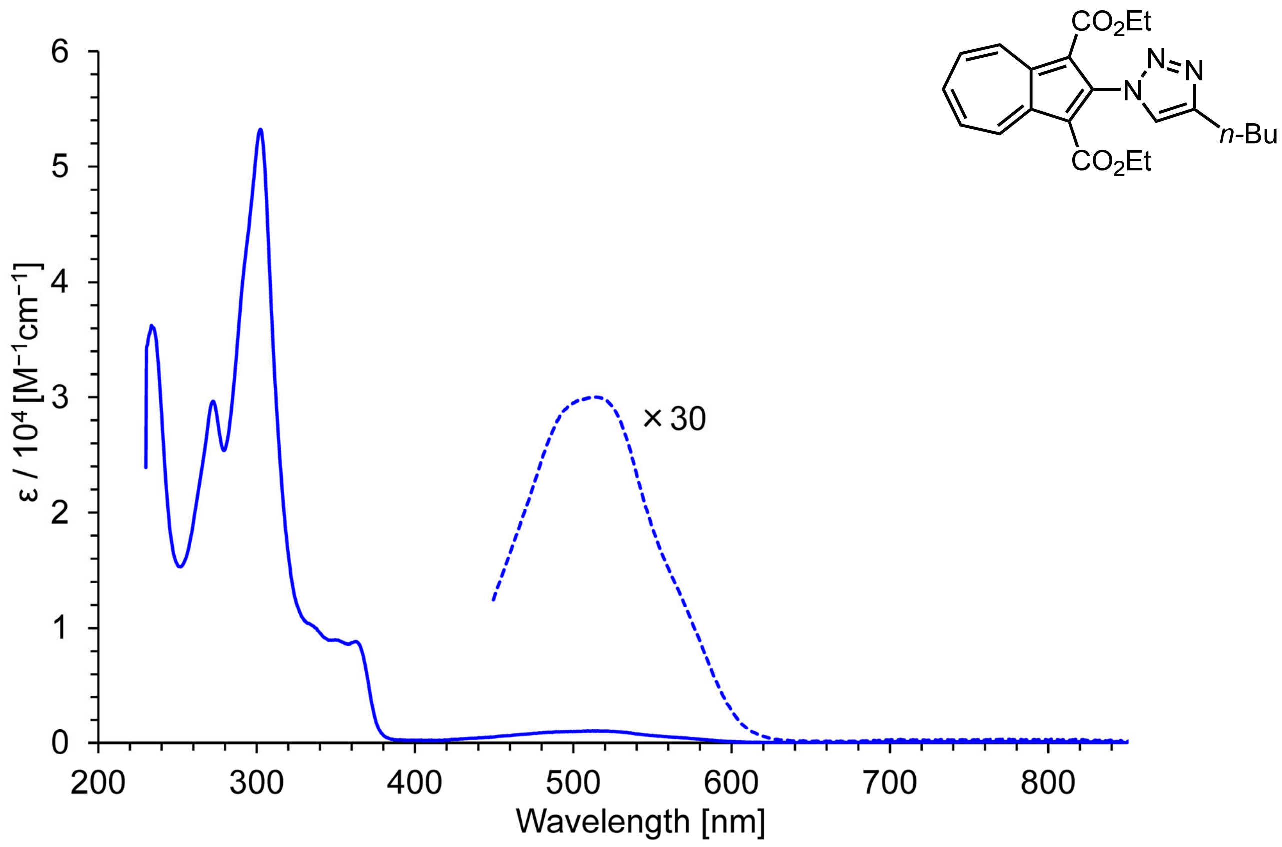

Figure S90. UV/Vis spectra of **5g** (2.33×10^{-5} M) in CH_2Cl_2. The spectrum in the visible region is magnified × 30 (dotted lines).

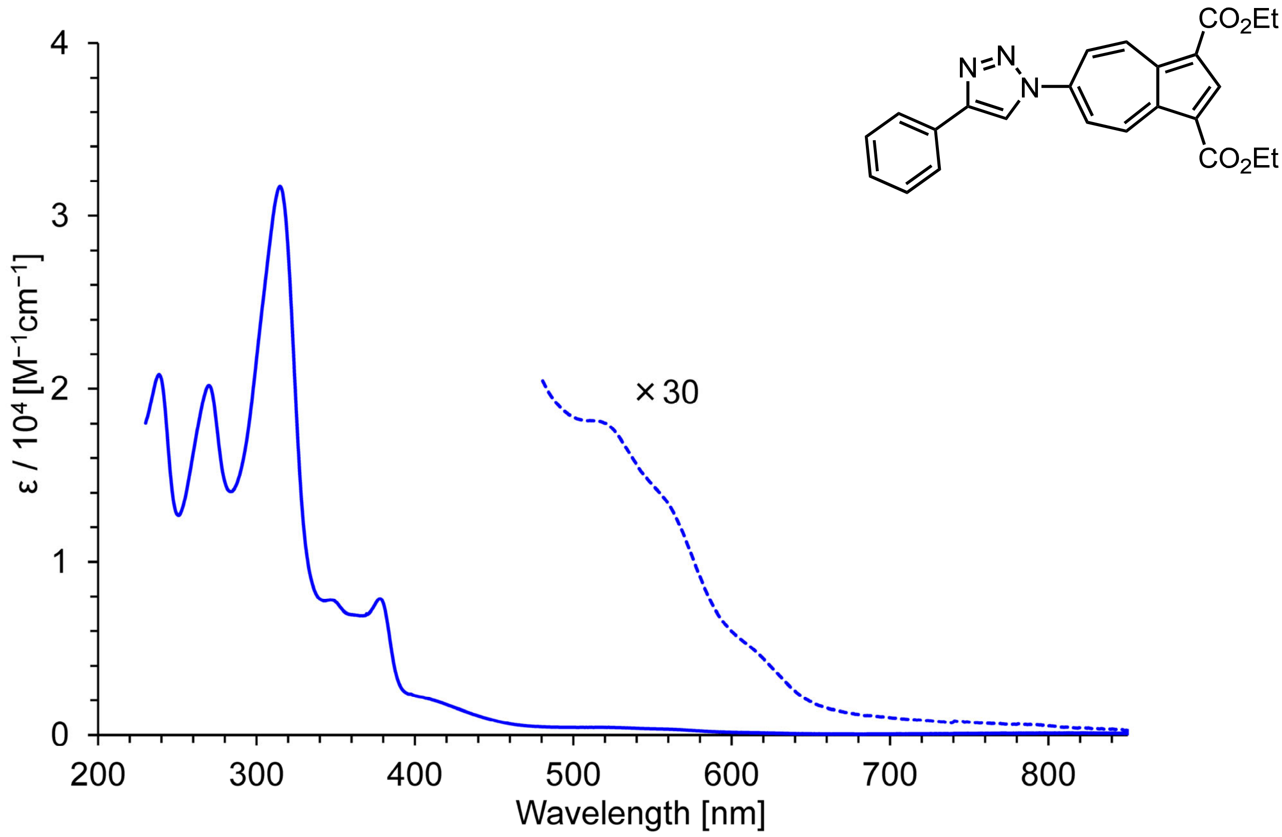

Figure S91. UV/Vis spectra of **6a** (3.01×10^{-5} M) in CH_2Cl_2. The spectrum in the visible region is magnified × 30 (dotted lines).

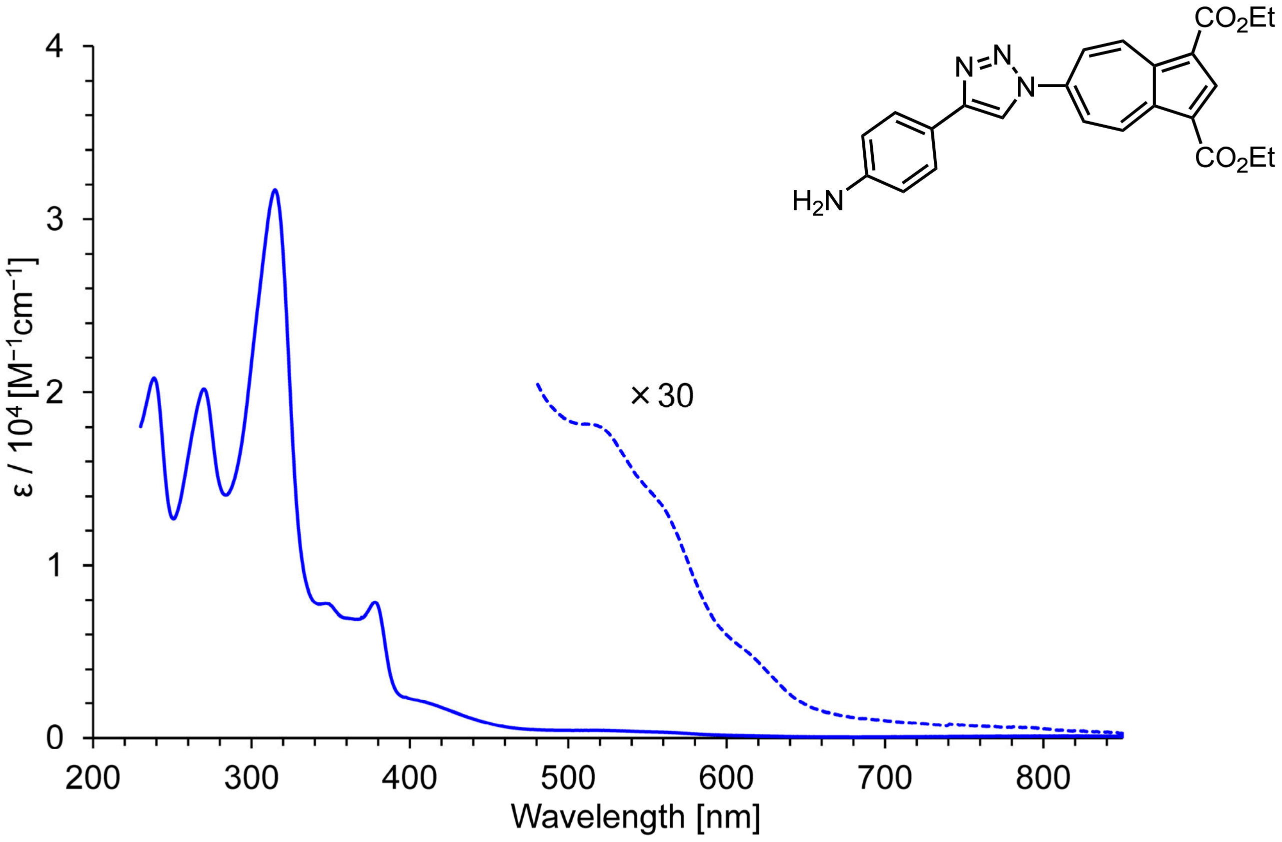

Figure S92. UV/Vis spectra of **6b** (2.65×10^{-5} M) in CH_2Cl_2. The spectrum in the visible region is magnified × 30 (dotted lines).

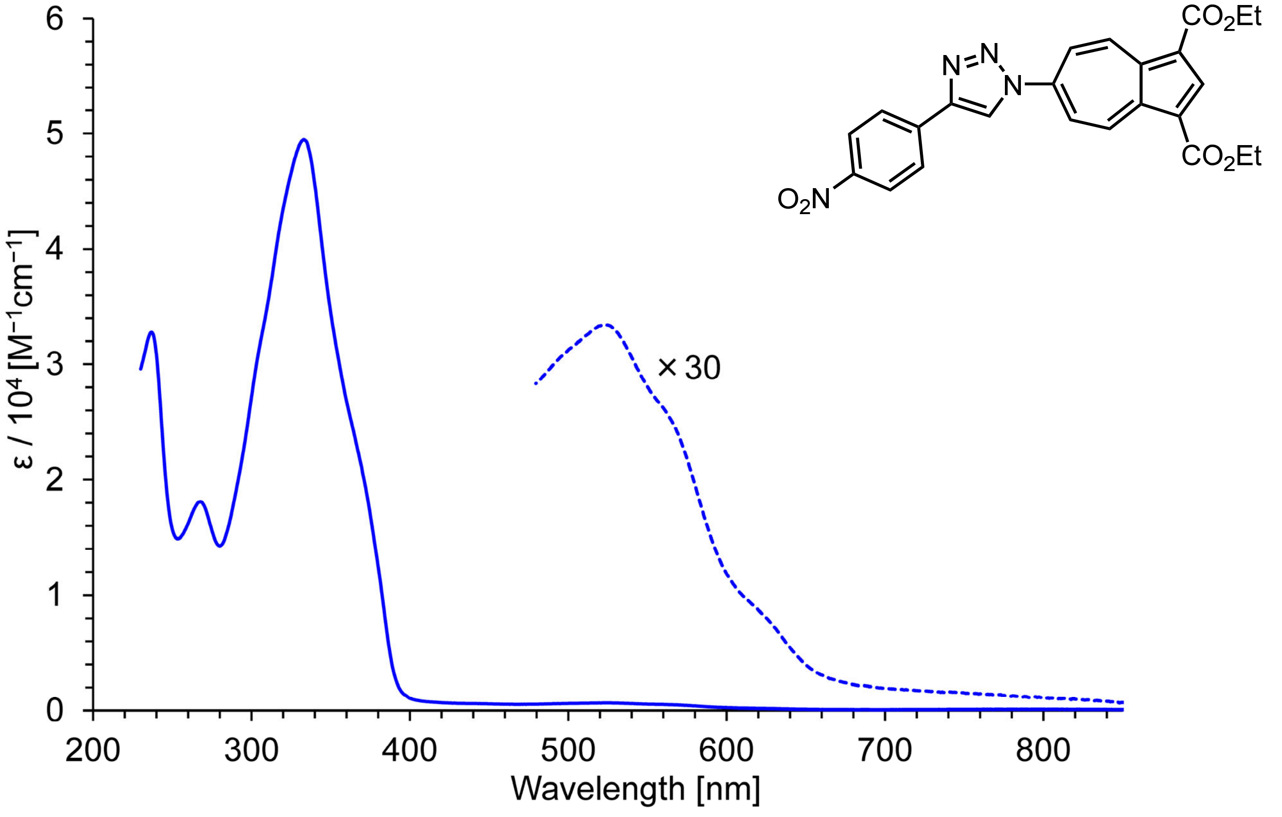

Figure S93. UV/Vis spectra of **6c** (2.13×10^{-5} M) in CH_2Cl_2. The spectrum in the visible region is magnified × 30 (dotted lines).

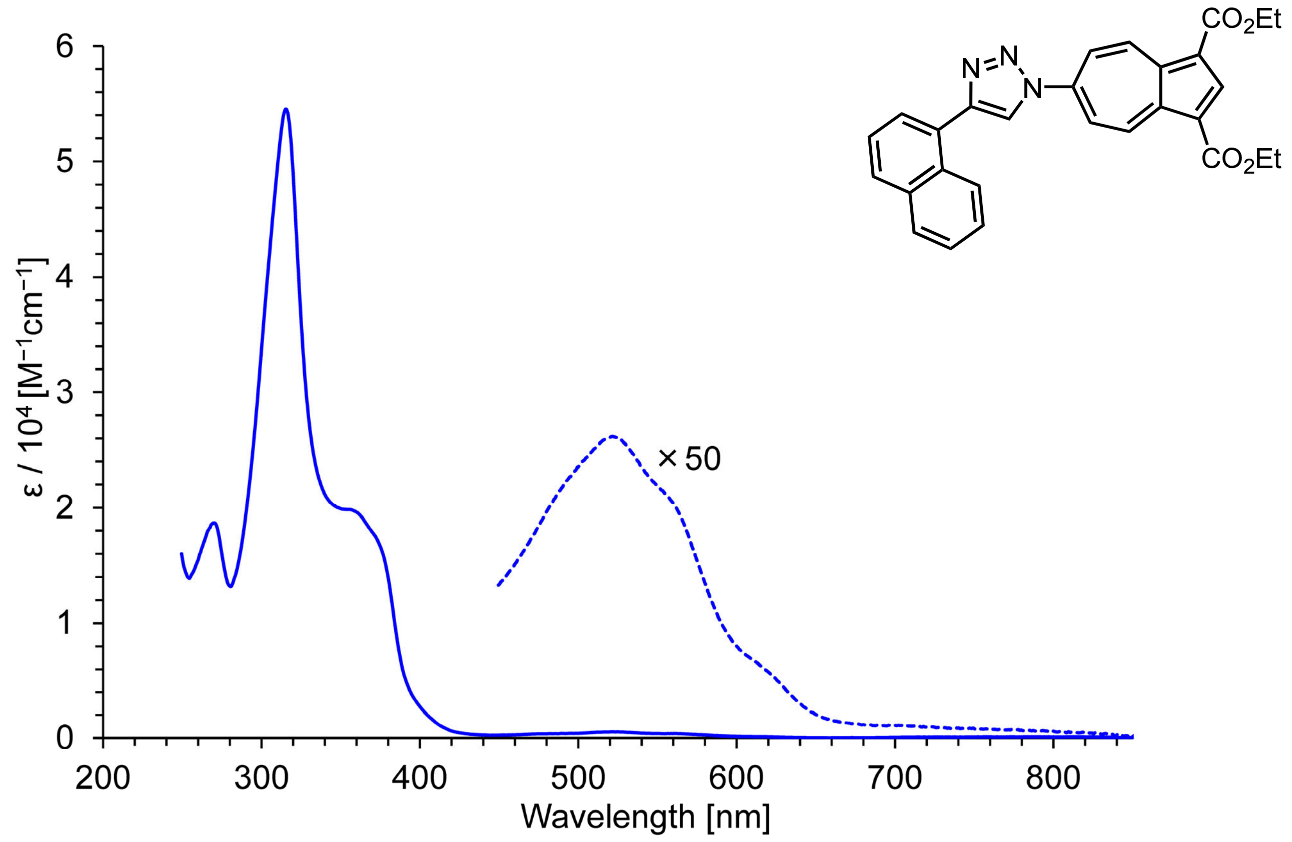

Figure S94. UV/Vis spectra of **6d** (2.65×10^{-5} M) in CH_2Cl_2. The spectrum in the visible region is magnified × 30 (dotted lines).

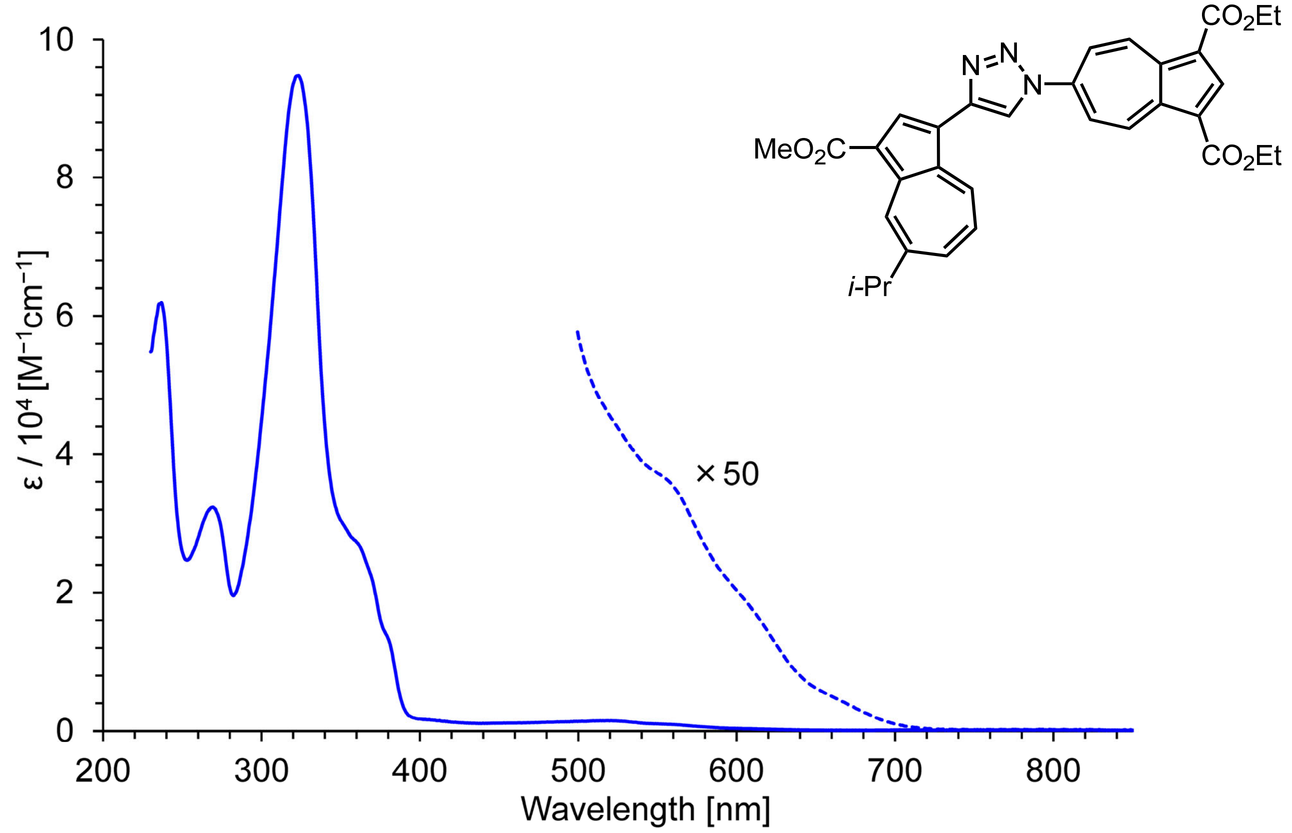

Figure S95. UV/Vis spectra of **6e** (2.29×10^{-5} M) in CH_2Cl_2. The spectrum in the visible region is magnified × 30 (dotted lines).

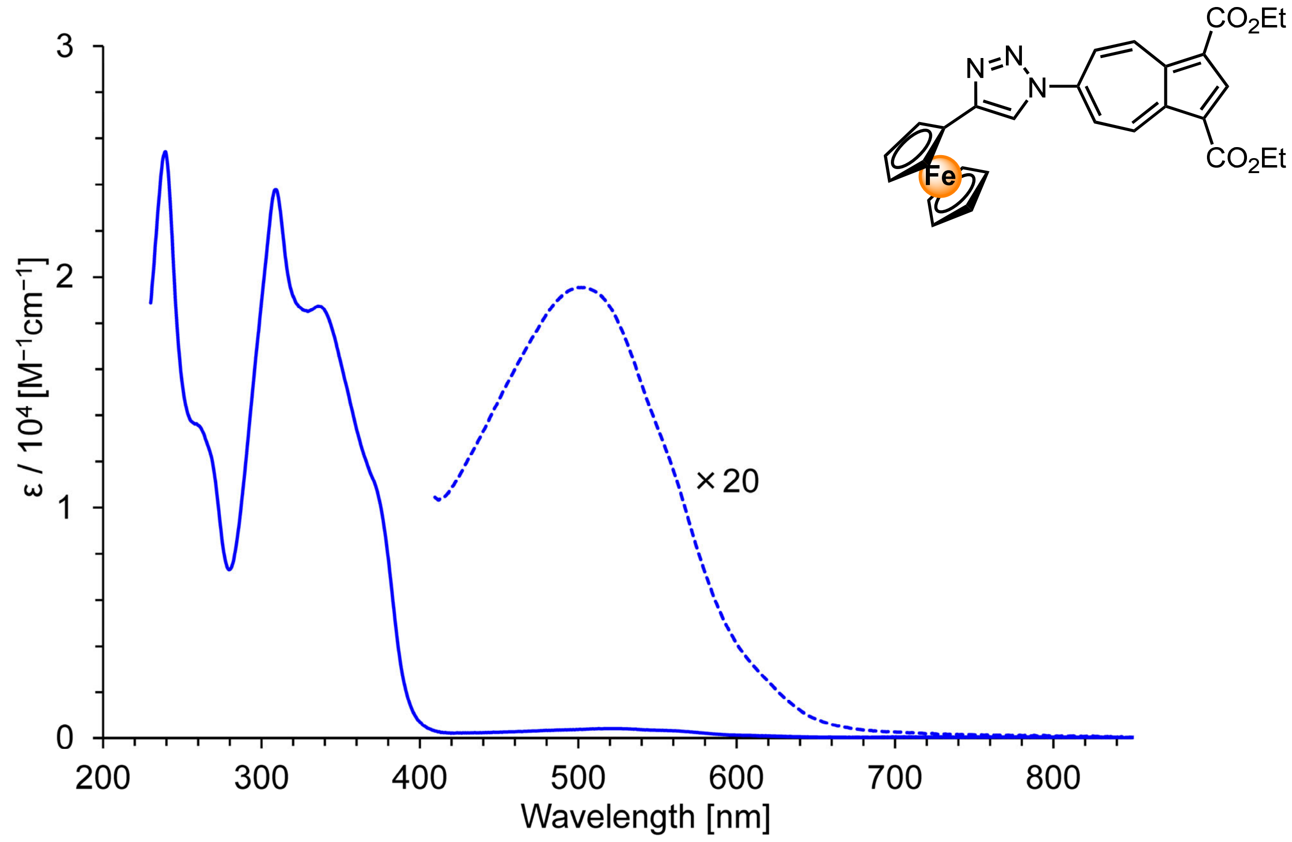

Figure S96. UV/Vis spectra of **6f** (4.13×10^{-5} M) in CH_2Cl_2. The spectrum in the visible region is magnified × 30 (dotted lines).

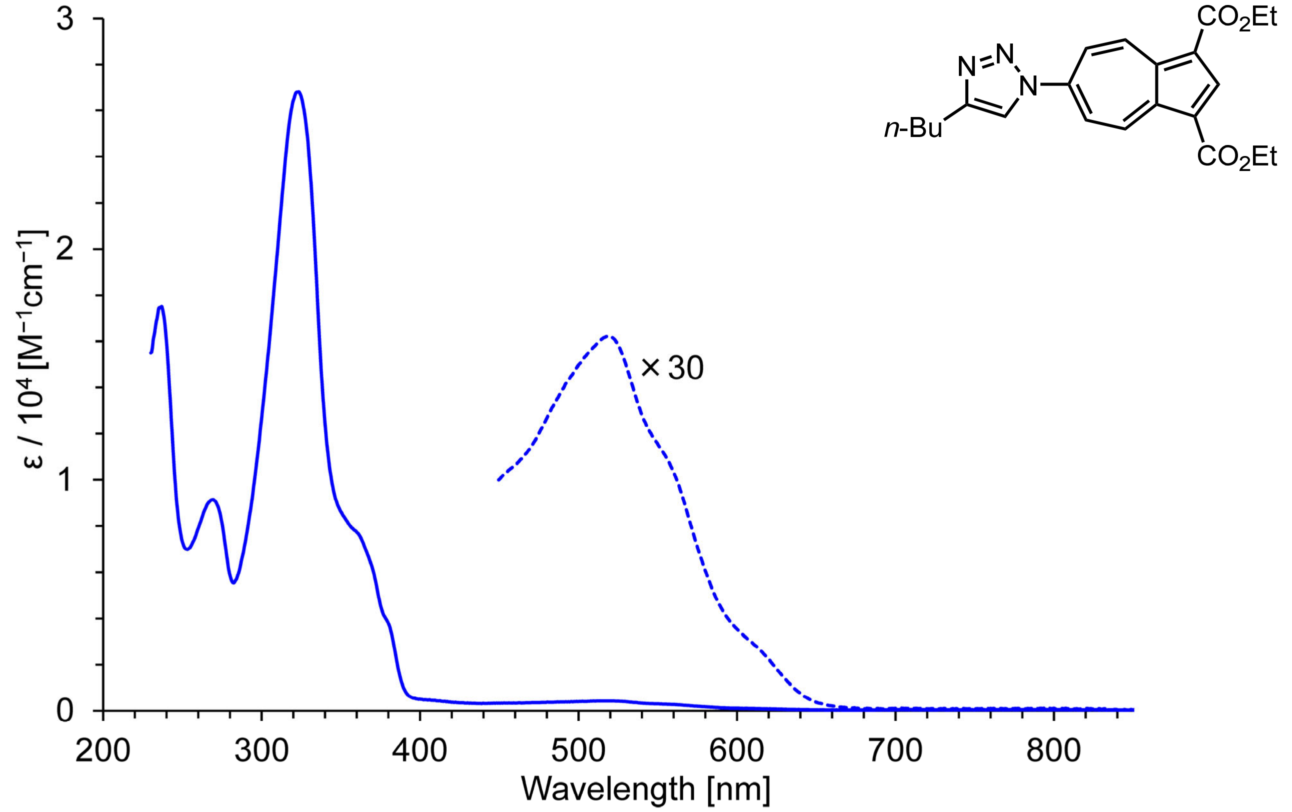

Figure S97. UV/Vis spectra of **6g** (3.12×10^{-5} M) in CH_2Cl_2. The spectrum in the visible region is magnified × 30 (dotted lines).

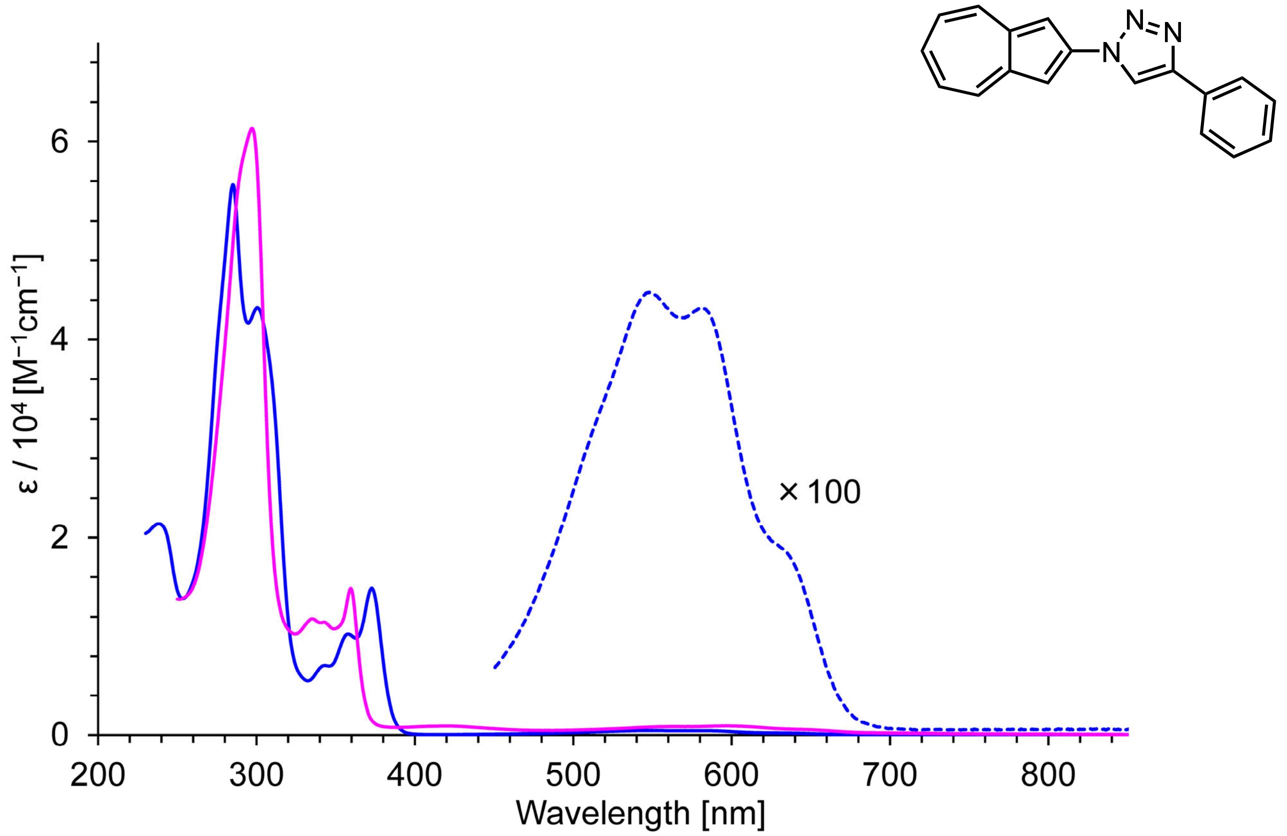

Figure S98. UV/Vis spectra of **7a** (3.78×10^{-5} M) in CH_2Cl_2 (blue line) and in 10% TFA/CH_2Cl_2 (pink line). The spectrum in the visible region is magnified × 100 (dotted lines).

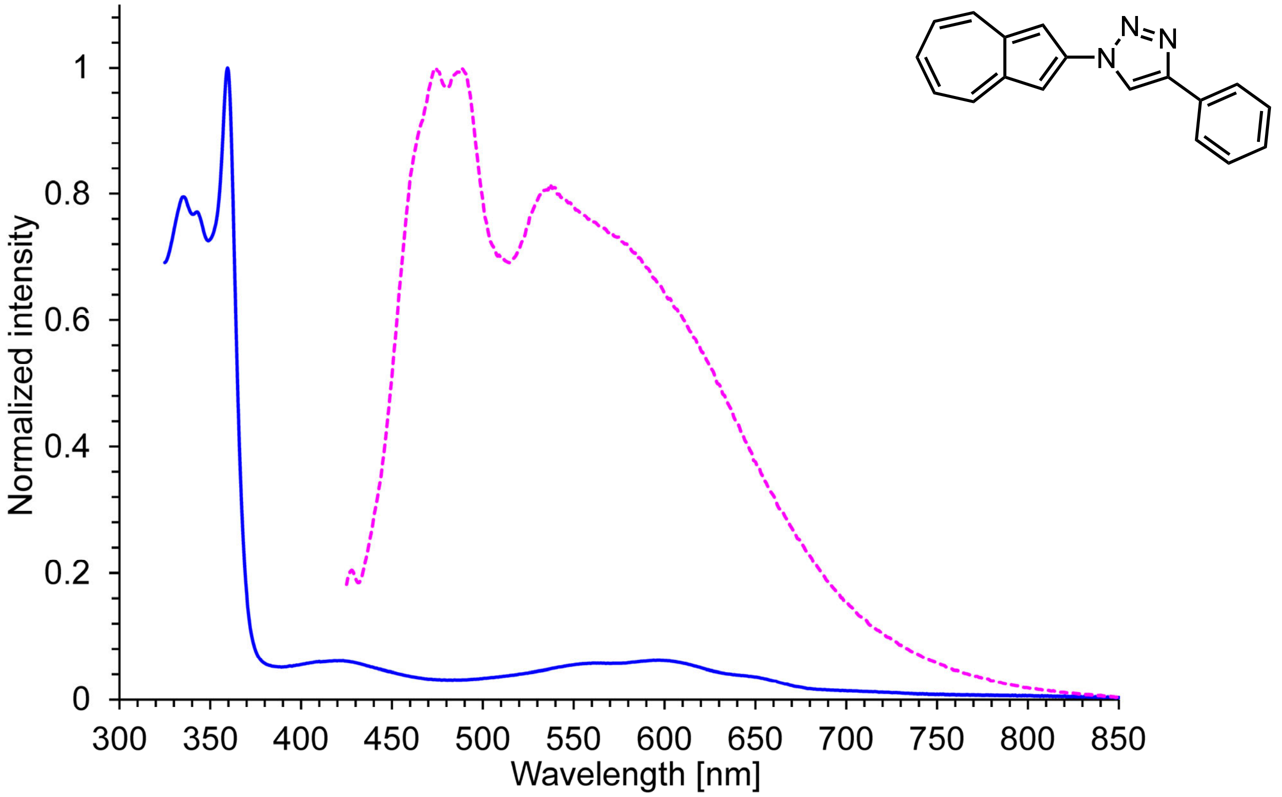

Figure S99. UV/Vis (blue line) and fluorescence spectra exited at λ_{EX} = 415 nm (pink-dotted line) of **7a** (3.78×10^{-5} M) in 10% TFA/CH_2Cl_2.

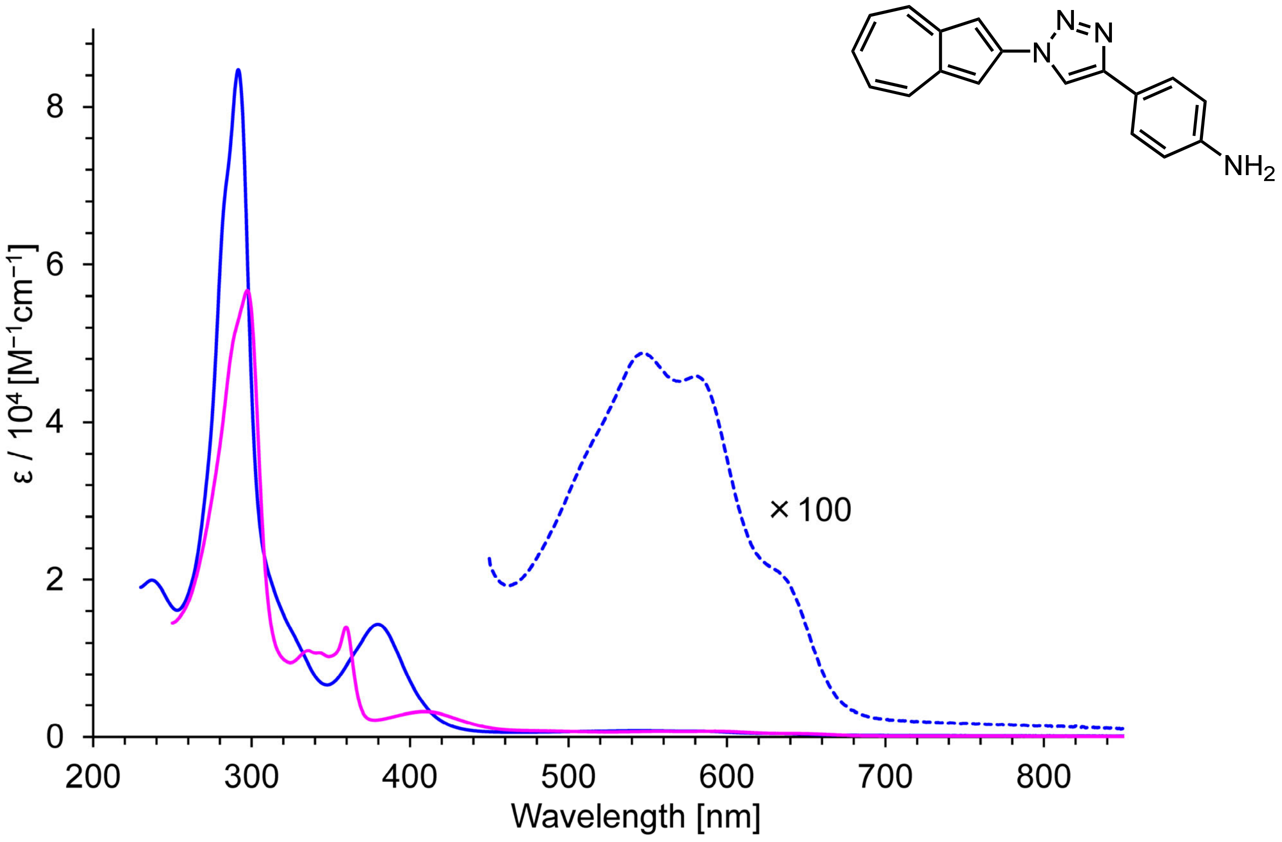

Figure S100. UV/Vis spectra of **7b** (3.53 × 10^{-5} M) in CH_2Cl_2 (blue line) and in 10% TFA/CH_2Cl_2 (pink line). The spectrum in the visible region is magnified × 100 (dotted lines).

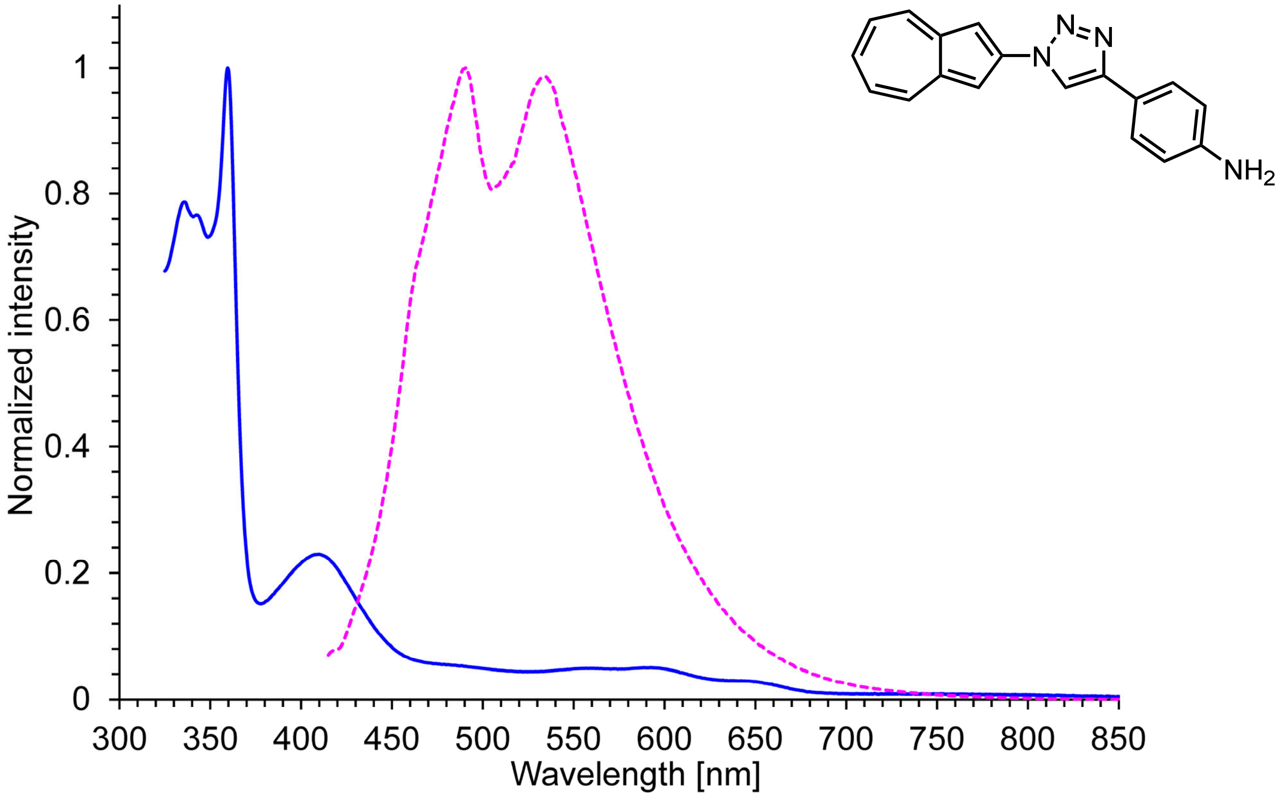

Figure S101. UV/Vis (blue line) and fluorescence spectra exited at λ_{EX} = 405 nm (pink-dotted line) of **7b** (3.53 × 10^{-5} M) in 10% TFA/CH_2Cl_2.

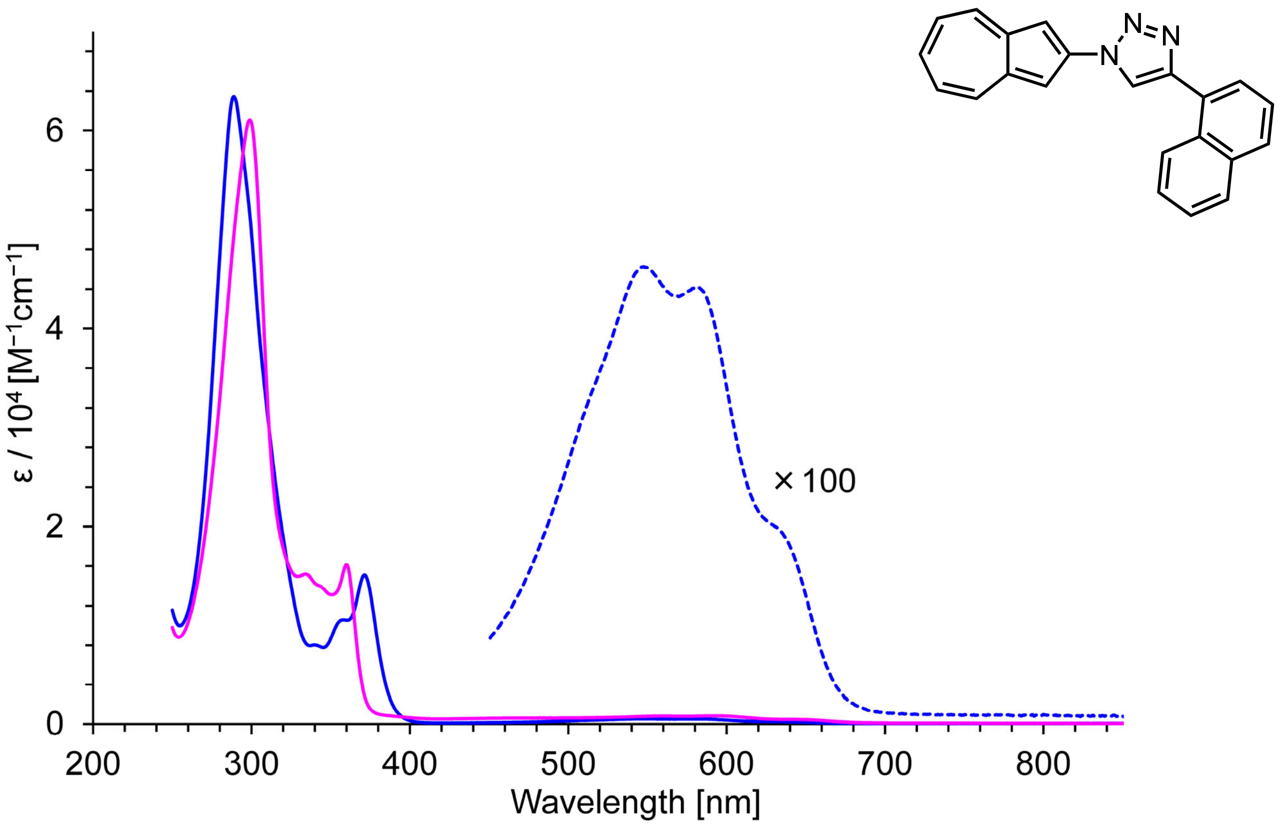

Figure S102. UV/Vis spectra of **7d** (3.05×10^{-5} M) in CH_2Cl_2 (blue line) and in 10% TFA/CH_2Cl_2 (pink line). The spectrum in the visible region is magnified × 100 (dotted lines).

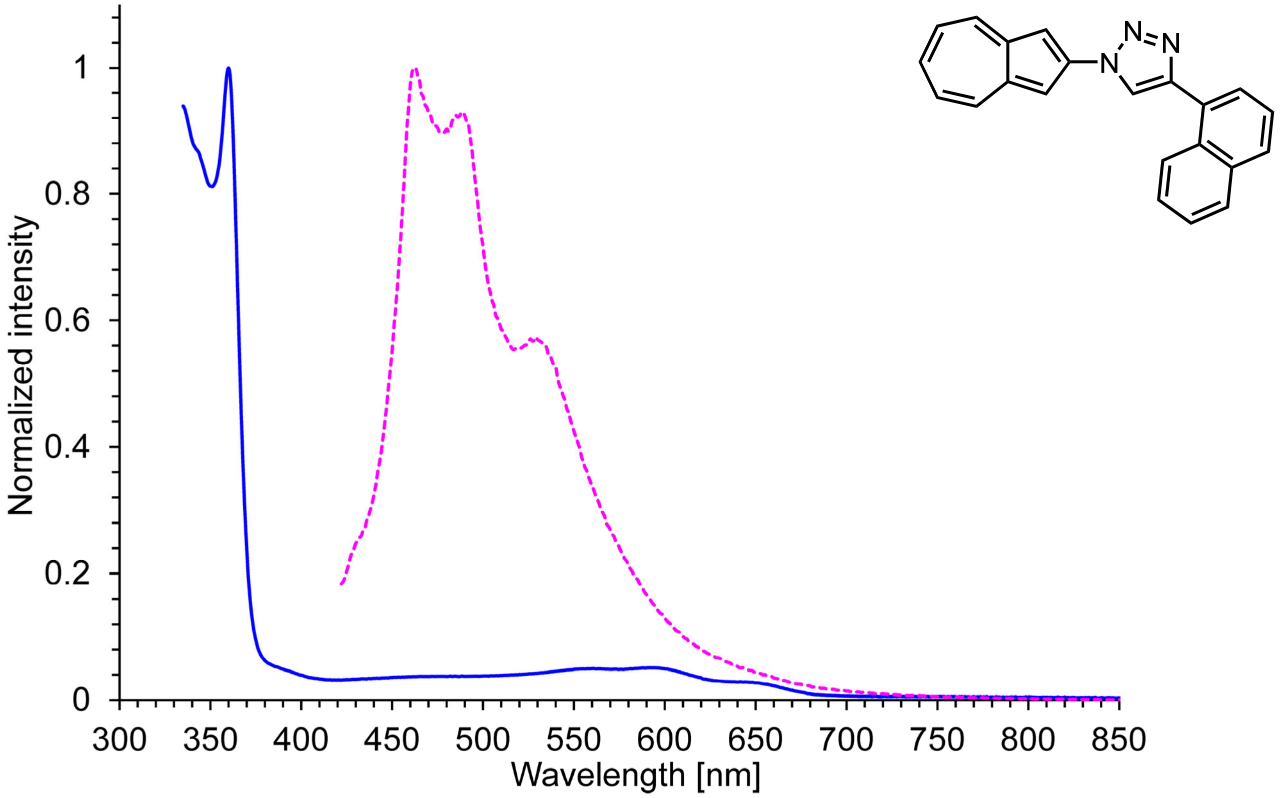

Figure S103. UV/Vis (blue line) and fluorescence spectra exited at λ_{EX} = 405 nm (pink-dotted line) of **7d** (3.05×10^{-5} M) in 10% TFA/CH_2Cl_2.

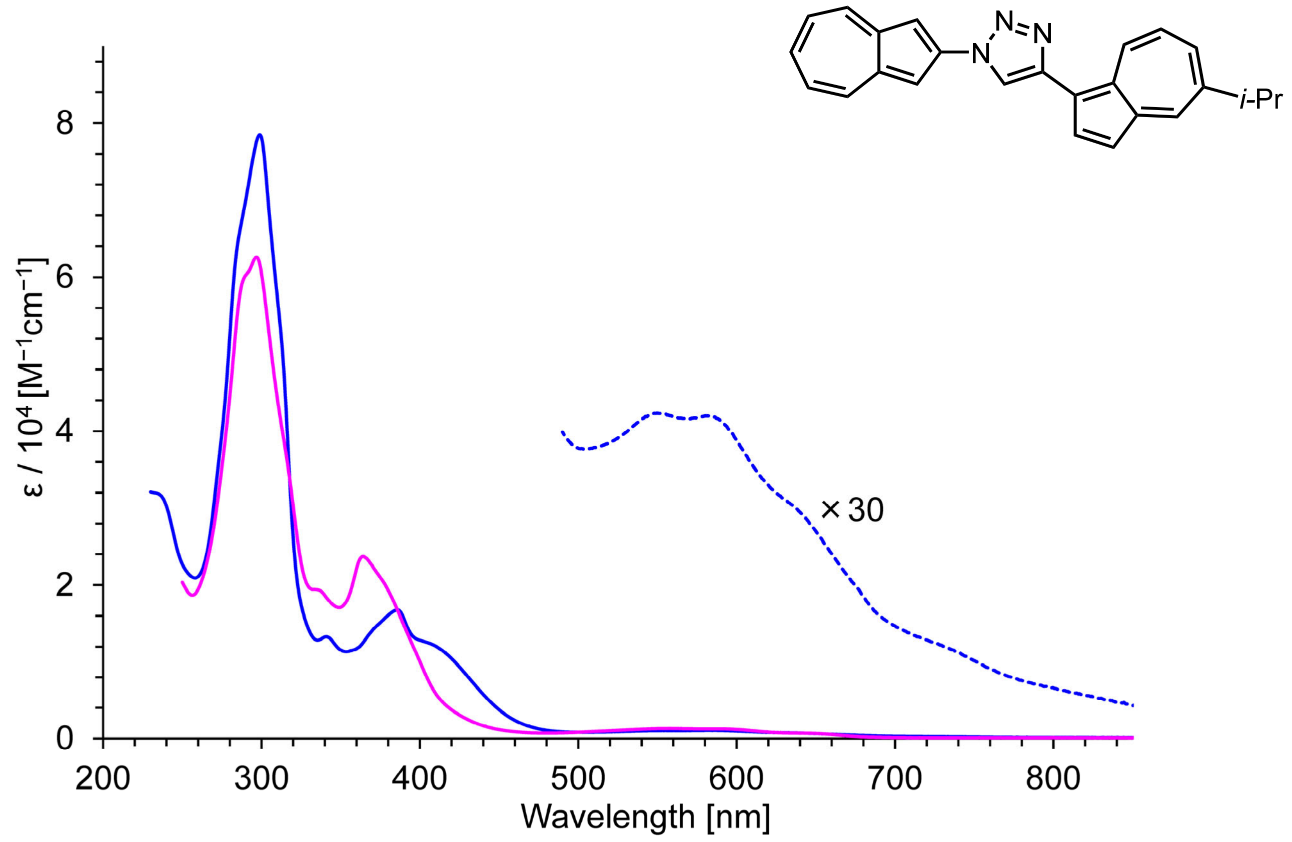

Figure S104. UV/Vis spectra of **7e** (2.06×10^{-5} M) in CH_2Cl_2 (blue line) and in 10% TFA/CH_2Cl_2 (pink line). The spectrum in the visible region is magnified × 100 (dotted lines).

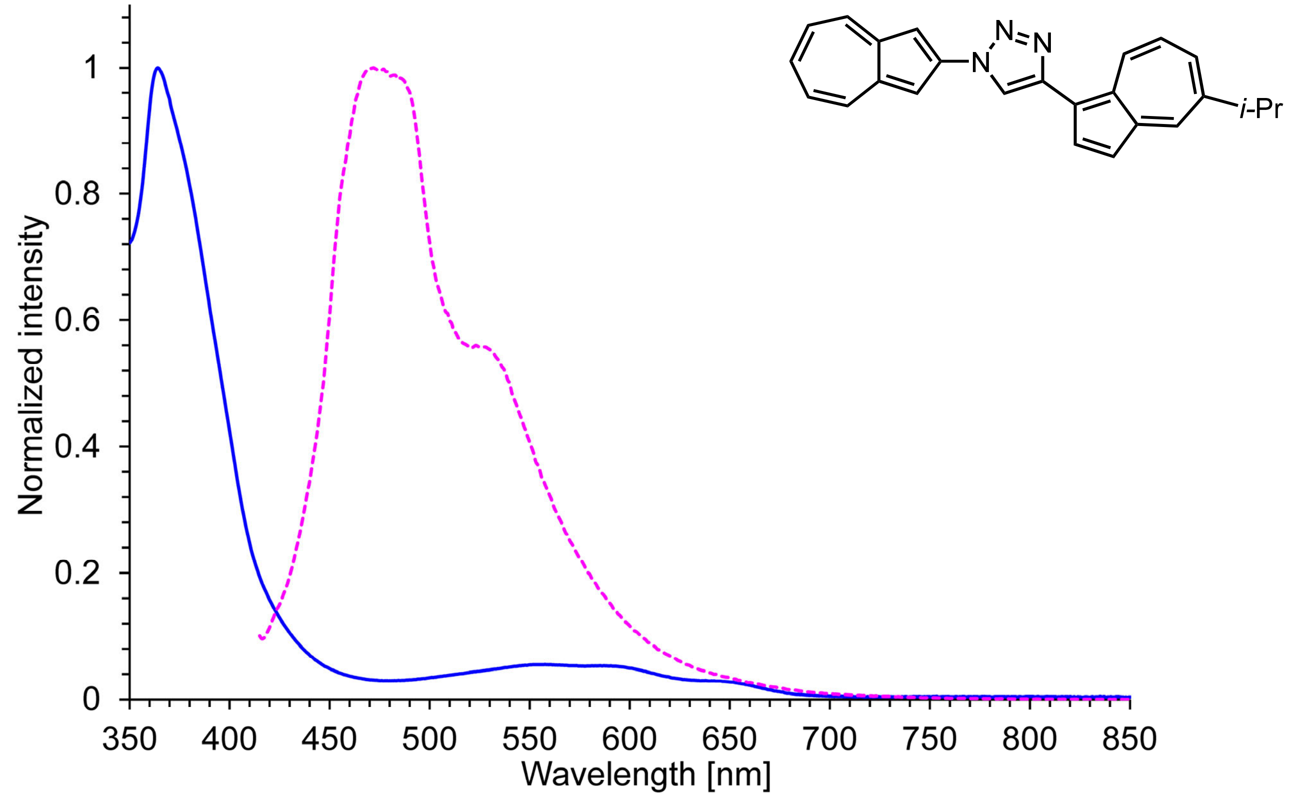

Figure S105. UV/Vis (blue line) and fluorescence spectra exited at λ_{EX} = 400 nm (pink-dotted line) of **7e** (2.06×10^{-5} M) in 10% TFA/CH_2Cl_2.

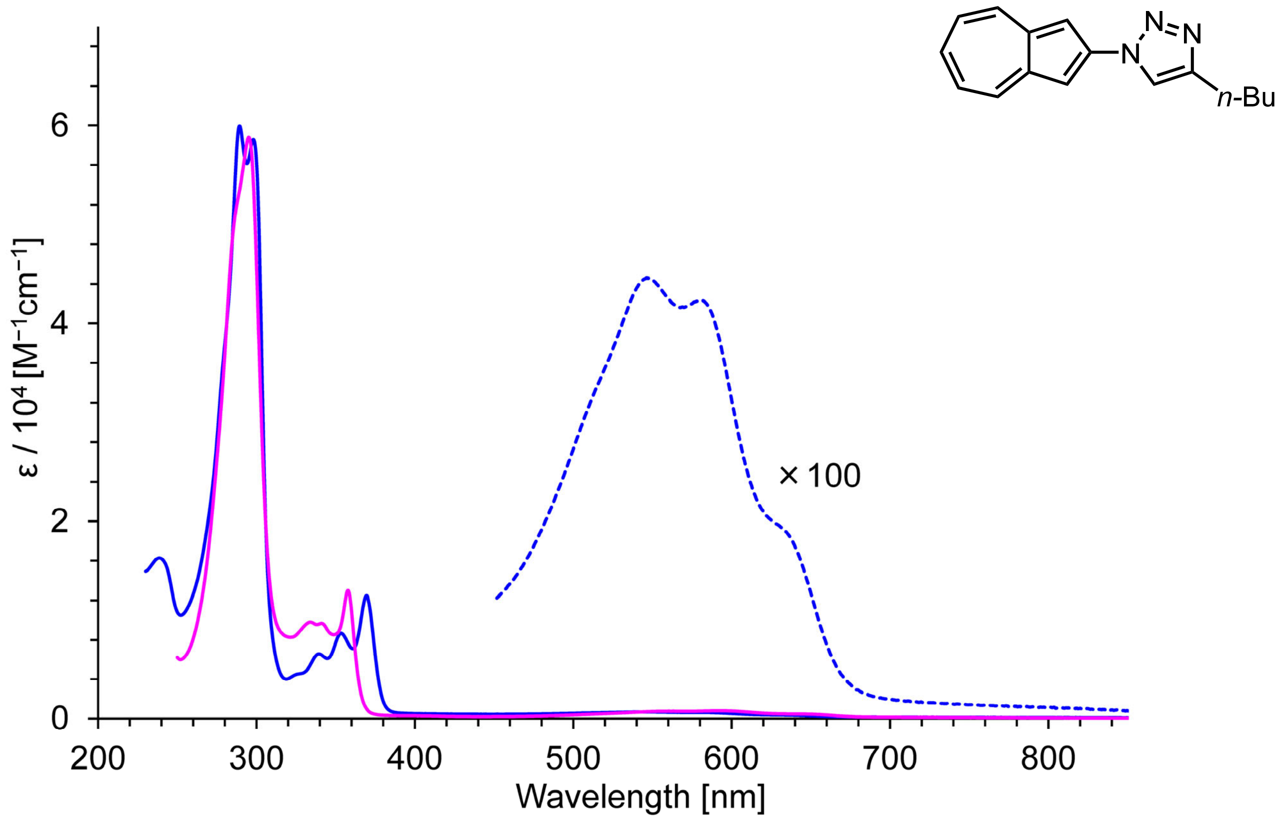

Figure S106. UV/Vis spectra of **7g** (4.53×10^{-5} M) in CH_2Cl_2 (blue line) and in 10% TFA/CH_2Cl_2 (pink line). The spectrum in the visible region is magnified × 100 (dotted lines).

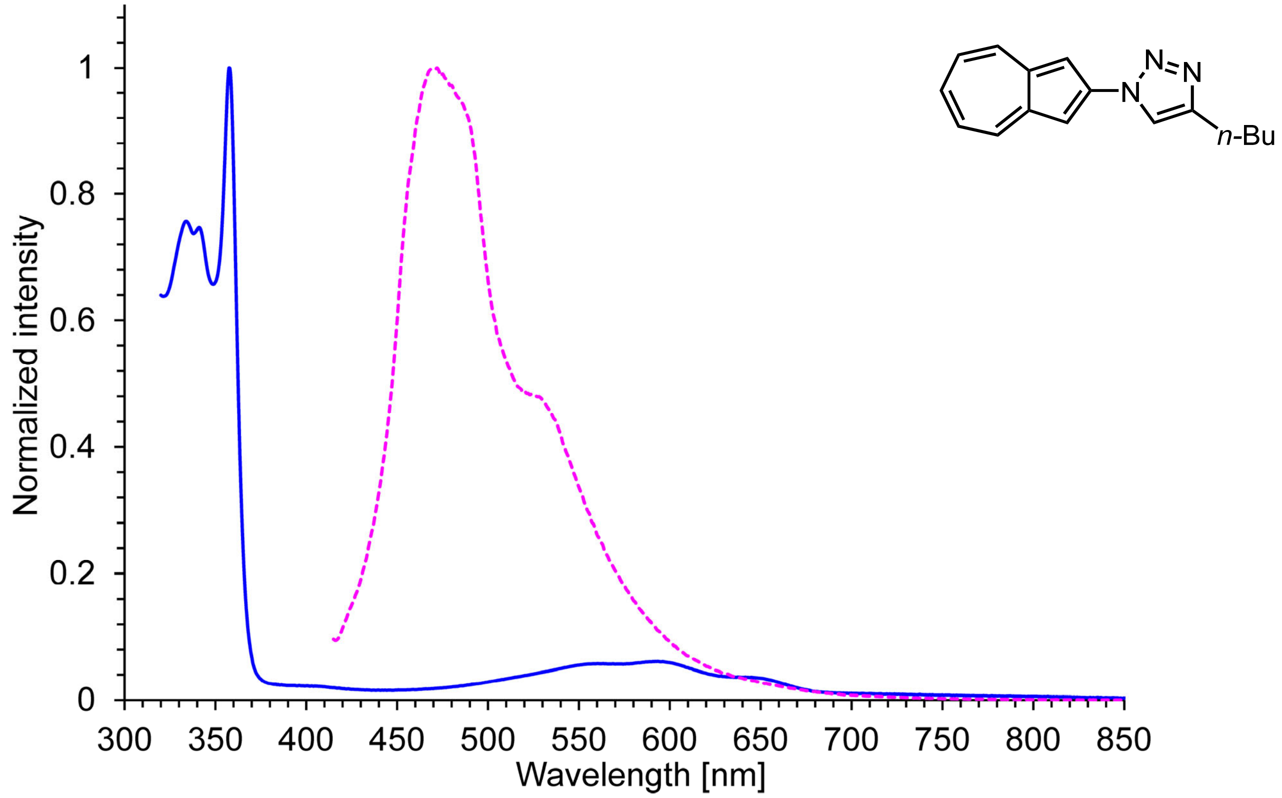

Figure S107. UV/Vis (blue line) and fluorescence spectra exited at λ_{EX} = 400 nm (pink-dotted line) of **7g** (4.53×10^{-5} M) in 10% TFA/CH_2Cl_2.

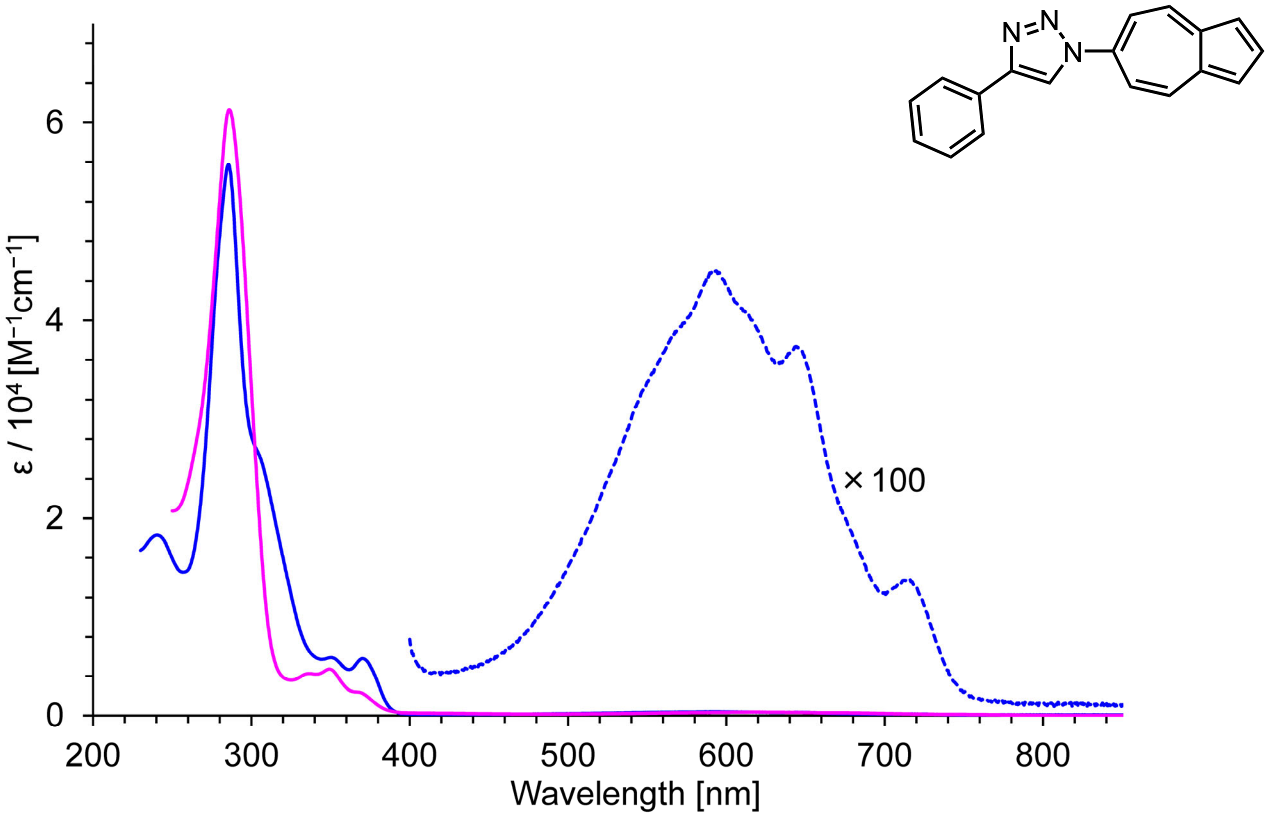

Figure S108. UV/Vis spectra of **8** (1.89×10^{-5} M) in CH_2Cl_2 (blue line) and in 10% TFA/CH_2Cl_2 (pink line). The spectrum in the visible region is magnified × 100 (dotted lines).

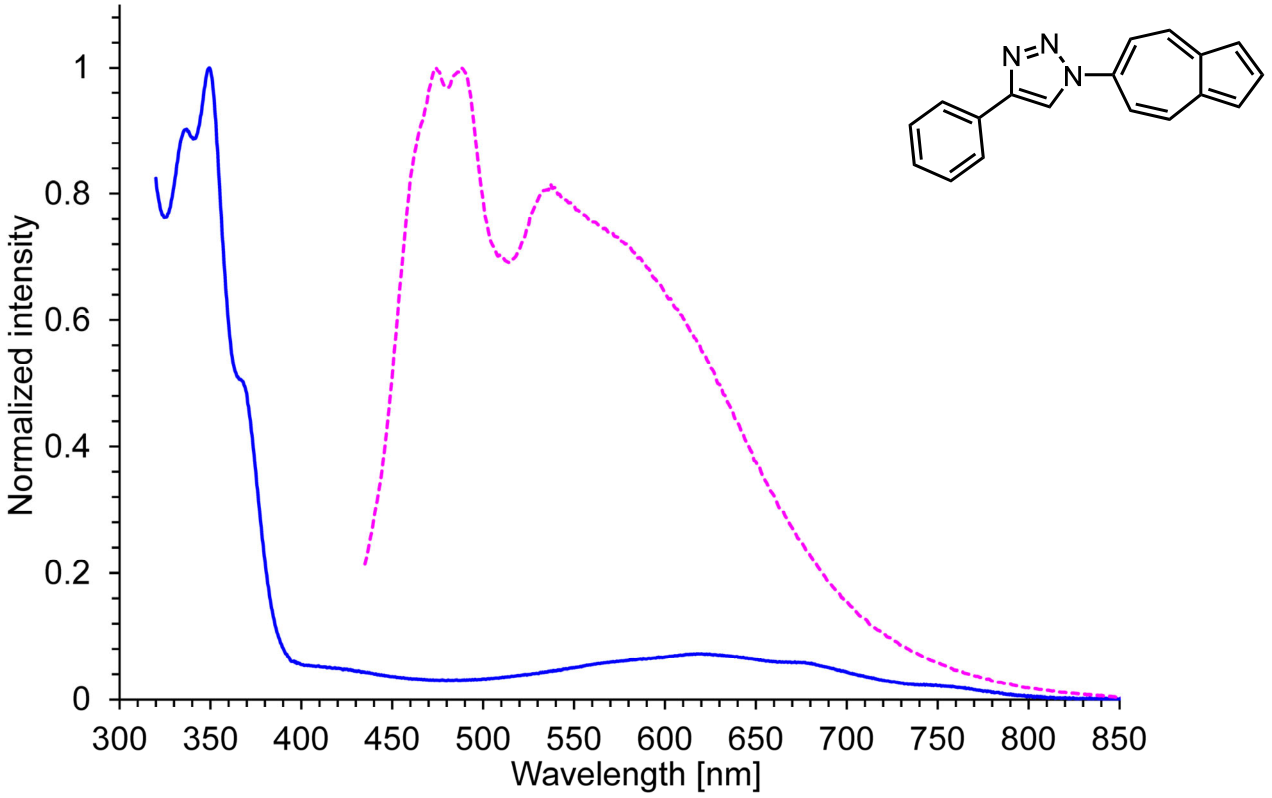

Figure S109. UV/Vis (blue line) and fluorescence spectra exited at λ_{EX} = 415 nm (pink-dotted line) of **8** (1.89×10^{-5} M) in 10% TFA/CH_2Cl_2.

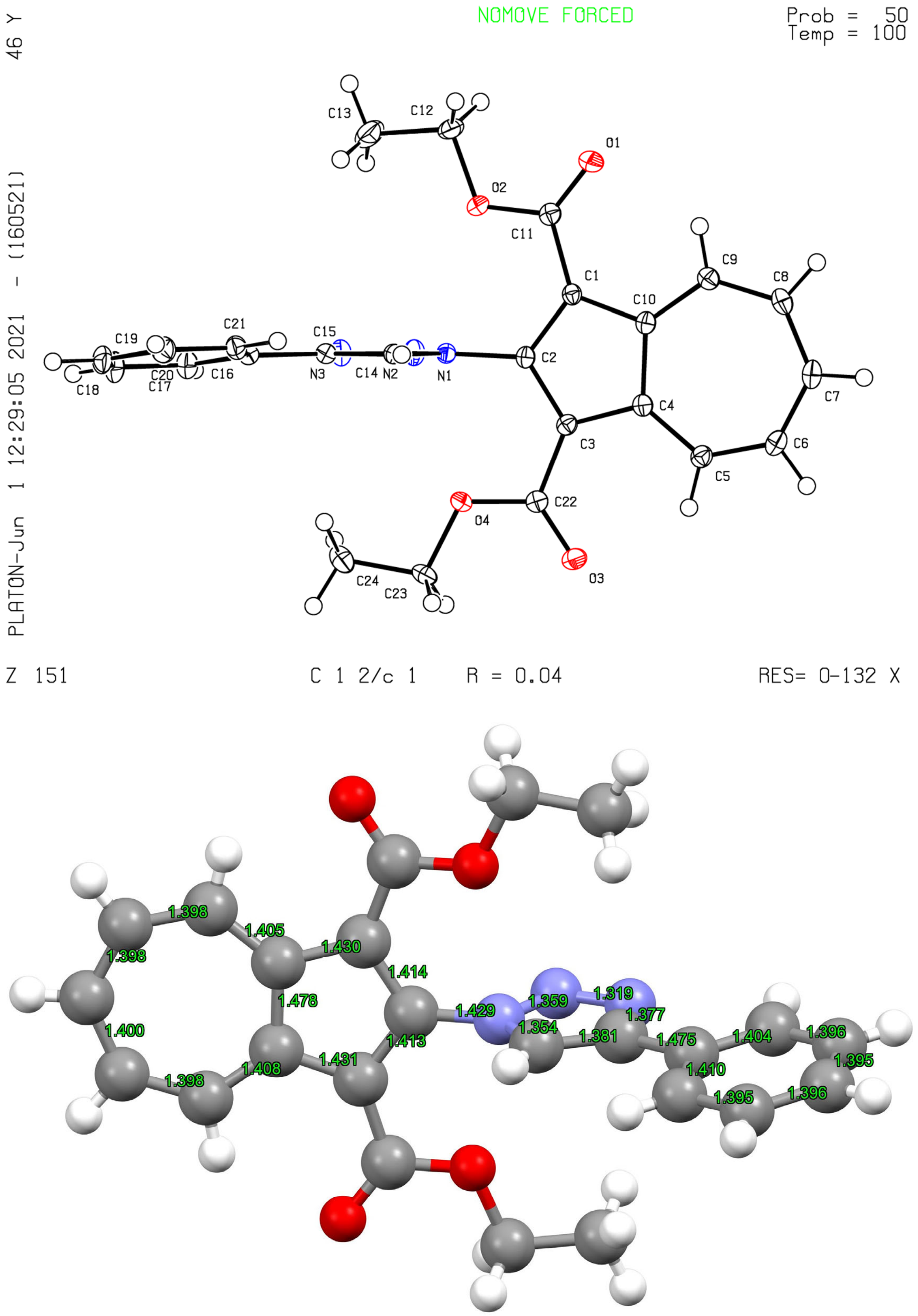

Figure S110. Top: ORTEP diagrams of **5a (CCDC 2087438)**; ellipsoids are drawn at the 50% probability level; recrystallized from CH_2Cl_2/MeOH. Bottom: Bond lengths of azulene and triazole moieties in **5a.**

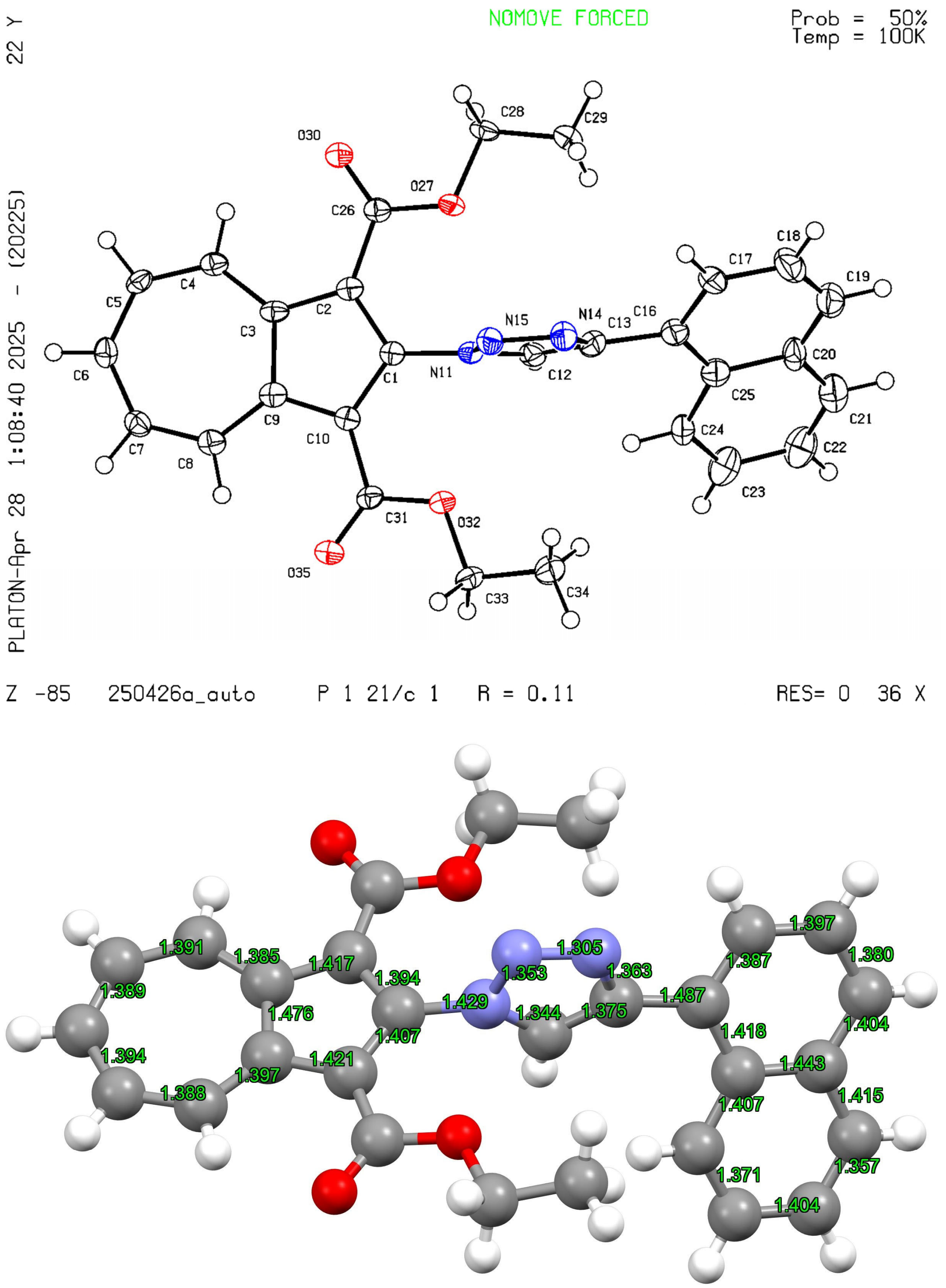

Figure S111. Top: ORTEP diagrams of **5d (CCDC 2447293)**; ellipsoids are drawn at the 50% probability level; recrystallized from CH_2Cl_2/MeOH. Bottom: Bond lengths of azulene and triazole moieties in **5d.**

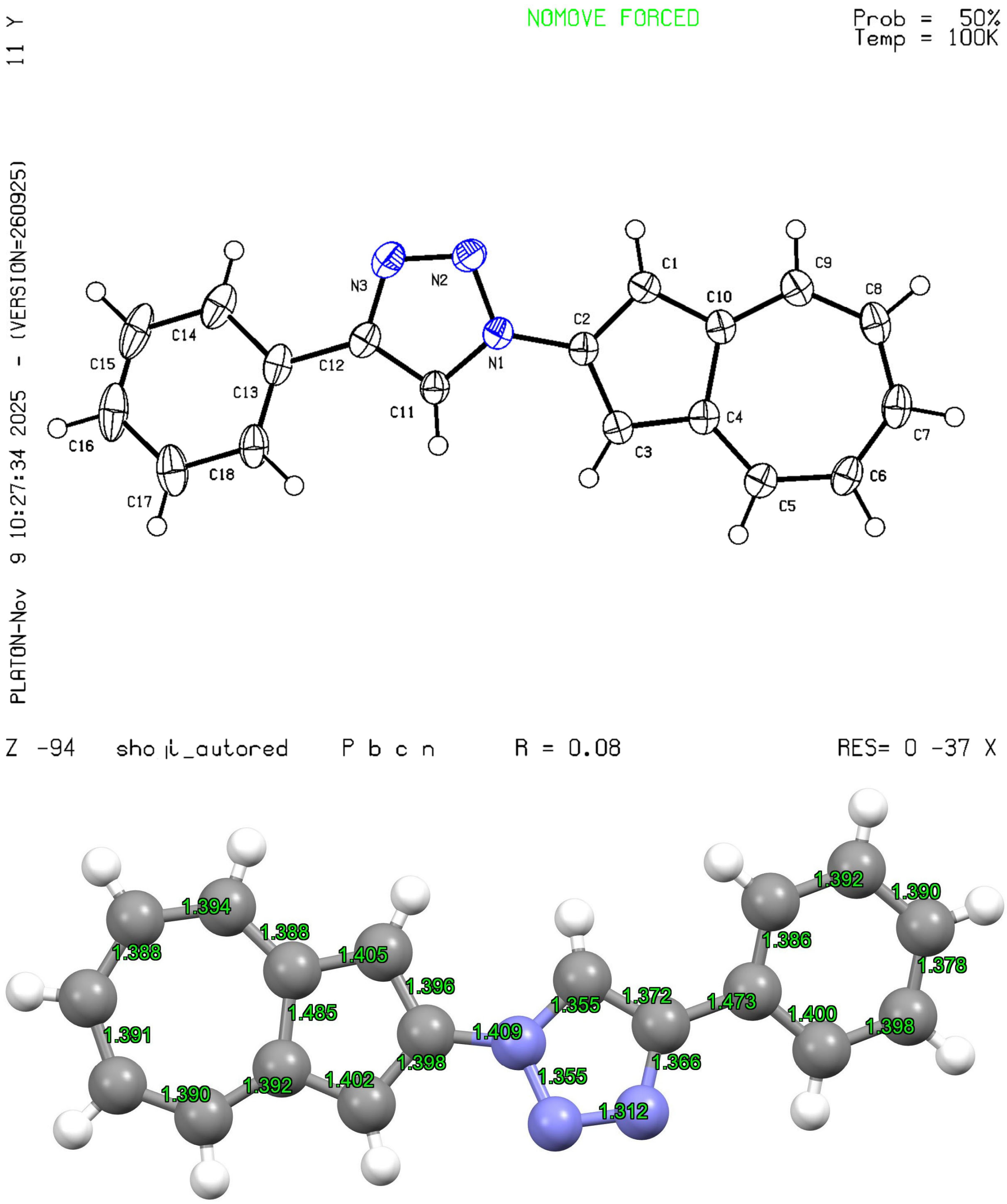

Figure S112. Top: ORTEP diagrams of **7a (CCDC 2502616)**; ellipsoids are drawn at the 50% probability level; recrystallized from CH_2Cl_2/MeOH. Bottom: Bond lengths of azulene and triazole moieties in **7a.**

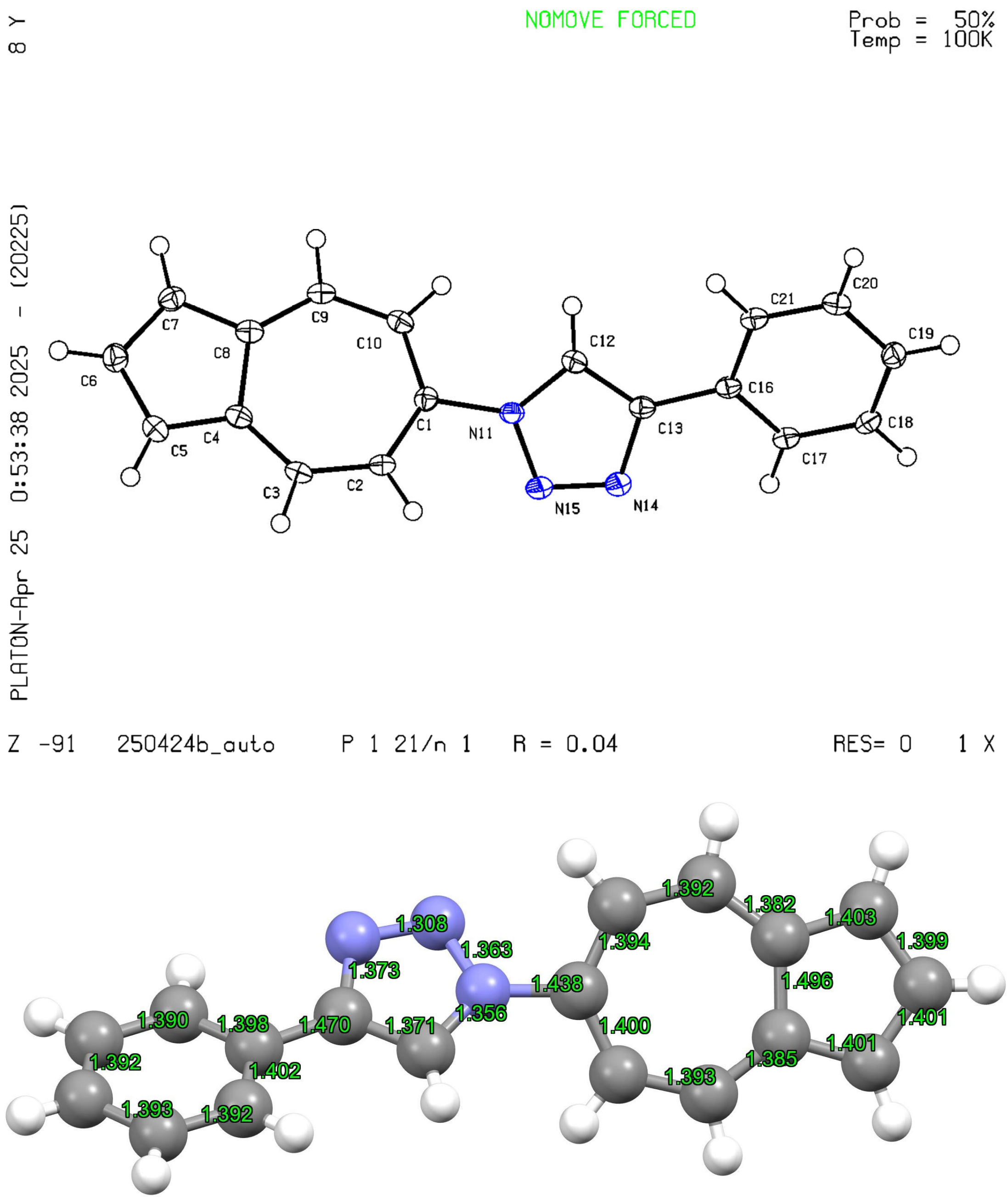

Figure S113. ORTEP diagrams of **8 (CCDC 2447292)**; ellipsoids are drawn at the 50% probability level; recrystallized from CH_2Cl_2/MeOH. Bottom: Bond lengths of azulene and triazole moieties in **8.**

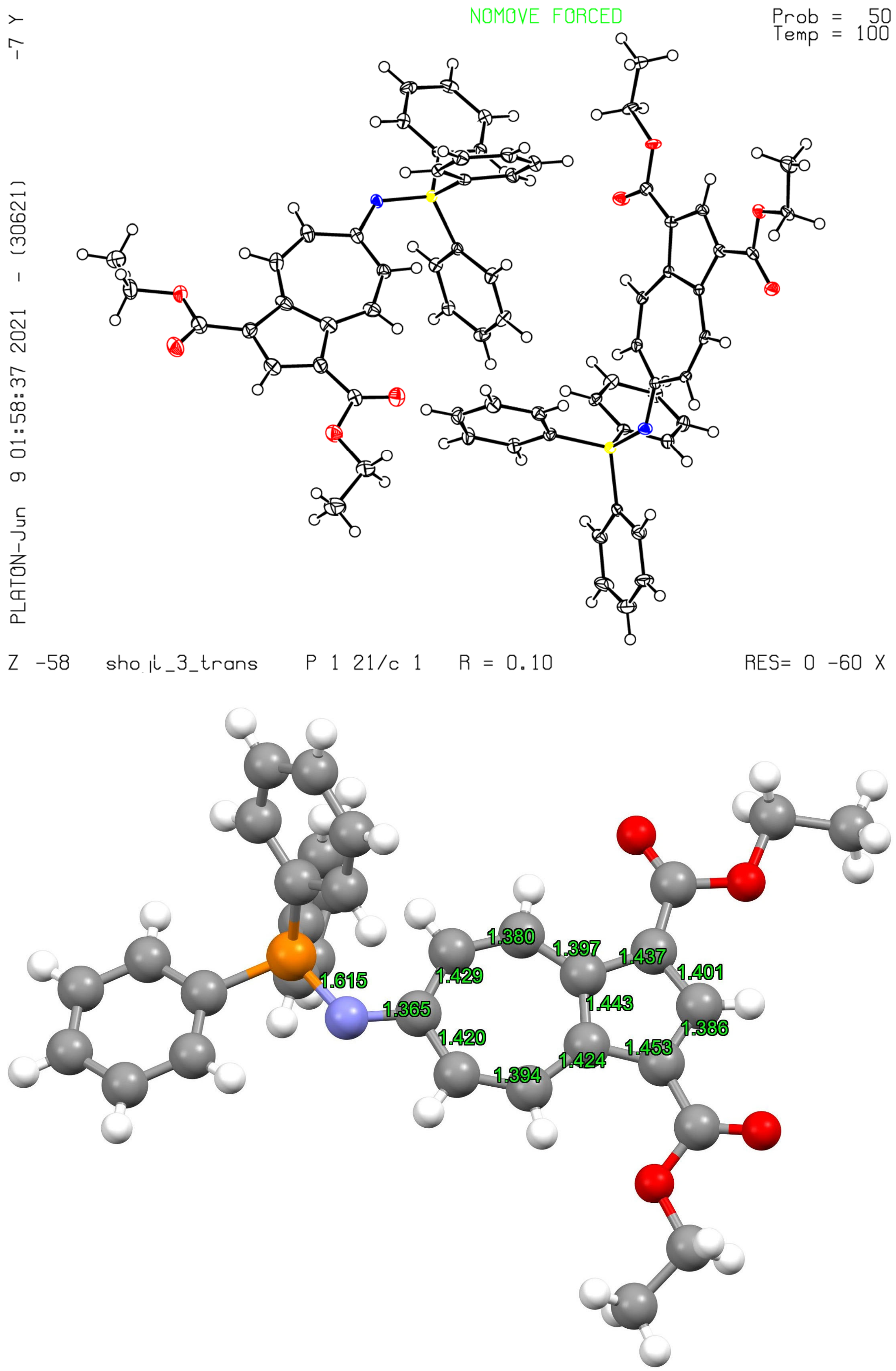

Figure S114. ORTEP diagrams of **9 (CCDC 2088856)**; ellipsoids are drawn at the 50% probability level; recrystallized from CH_2Cl_2/MeOH. Bottom: Bond lengths of azulene and iminophosphorane moieties in **8.**

Table S1. Selected crystal data of **5a**, **5d**, **7a**, **8**, and **9**.

CCDC number	**5a** 2087438	**5d** 2447293	**7a** 2502616	**8** 2447292	**9** 2088856
Crystal system	Monoclinic	Monoclinic	Orthorhombic	Monoclinic	Monoclinic
Space group	C2/c	$P2_1/c$	Pbcn	$P2_1/n$	$P2_1/c$
a (Å)	20.5744(7)	7.6094(4)	11.0837(5)	5.7519(3)	28.370(12)
b (Å)	7.3366(3)	30.2587(17)	12.2468(5)	31.3515(18)	8.343(4)
c (Å)	27.4326(8)	10.0680(5)	19.8090(7)	7.2953(3)	23.459(10)
α (°)	90	90	90	90	90
β (°)	97.492(3)	104.291(5)	90	94.133(4)	95.659(8)
γ (°)	90	90	90	90	90
V (Å^3)	4105.5(3)	2246.4(2)	2688.87(19)	1312.15(12)	5526(4)
Z	8	4	8	4	8
Temp (K)	100	100	100	100	100
μ (Mo Kα) (mm^{-1})	0.093	0.094	0.081	0.084	0.140
D_{calc} (g/cm^3)	1.344	1.376	1.34	1.373	1.316
R_1 (I ≥ 2σ)	0.0412	0.1053	0.078	0.0425	0.0970
wR_2 (all data)	0.1081	0.1783	0.1849	0.1037	0.1993
